# Access to α‑Functionalized Amines via Calcium-Catalyzed Deacetylations

**DOI:** 10.1021/acs.joc.5c03185

**Published:** 2026-03-23

**Authors:** Michael P. Cameron, Mark G. McLaughlin

**Affiliations:** School of Chemistry and Chemical Engineering, 1596 Queen’s University Belfast, Belfast BT9 5AG, U.K.

## Abstract

A calcium-catalyzed approach to accessing α-functionalized amines via deacetylation has been developed. Using Ca­(NTf_2_)_2_/*n*BuNPF_6_ as a mild Lewis acid system, a range of α-acetoxy substrates undergo smooth deacetylation and functionalization. The reaction displays excellent substrate tolerance and accommodates diverse nucleophiles, including sulfur, indole, amide, and cyanide derivatives. Furthermore, telescoped deprotection–reprotection protocols enable facile access to a variety of *N*-protected amines in high yields, providing a simple and practical route to valuable α-functionalized amine scaffolds.

## Graphical abstract

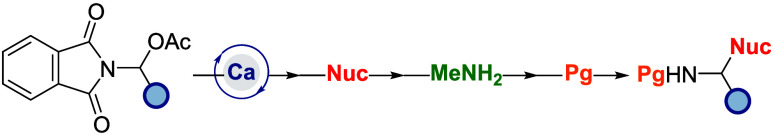

## Introduction

Amines are ubiquitous in chemistry, with an almost innumerable array of uses across many sectors including pharmaceutical, agrochemical, bulk, and fine chemicals.^1,2^ Of particular interest are amines with α-functionalization, as this often plays a key role in determining their behavior such as biological activity.^3^ As such, the development of new methods for the construction of α-functionalized amines remains a cornerstone of modern synthetic organic chemistry,^4^ with advances in organocatalysis,^5^ transition metal catalysis,^6^ photoredox catalysis,^7–9^ and electrosynthesis.^10,11^ Although these methods all produce the desired product, they each have limitations based on catalyst availability (commercial vs bespoke), complicated and costly reaction setups, or simple functional group tolerance. Furthermore, the requirement for strictly anhydrous and oxygen-free conditions in many of these novel methods somewhat limits their uptake in medicinal chemistry.^12^

As part of an ongoing medicinal chemistry program focused on the development of new covalent modalities, we required ready access to a wide range of α-functionalized amines, with a particular emphasis on installing groups that provide a functional handle for further derivatization or inherent hydrogen bonding ability. Given our continuing interest in the use of calcium as a Lewis acid catalyst,^13–15^ we reasoned that we could use deoxyfunctionalization to install the aforementioned functionality. As such and based on our previous experience, we began by surveying a range of potential ether functionalities in the α-position; however, many of these were difficult to access reproducibly. Interestingly, many of the published methods installed an α-OAc group before further functionalization,^16–18^ and we wondered if this would be amenable to our system, Scheme 1.

**Scheme 1:**
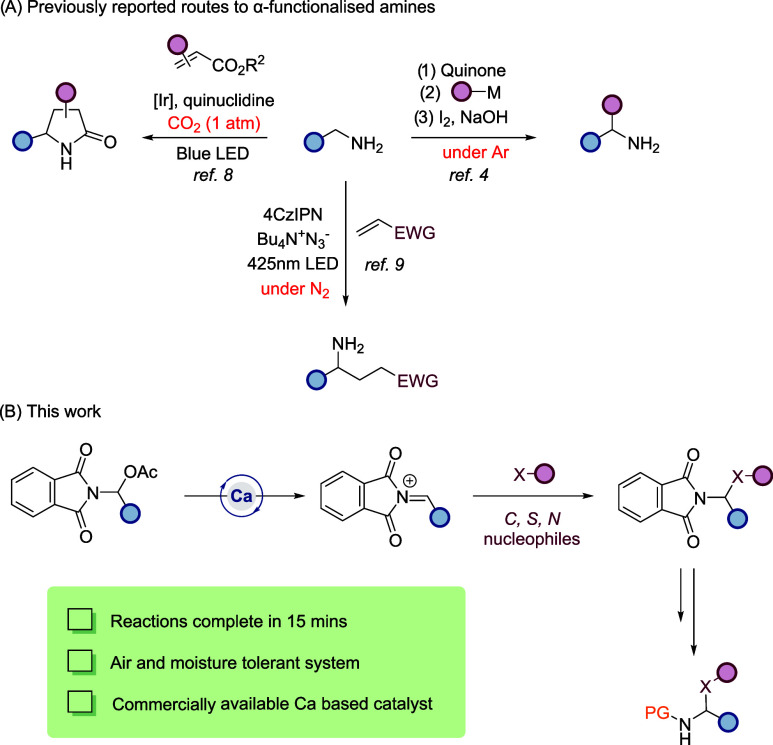
Strategies toward α-Functionalized Amines

## Results and Discussion

Subjecting **1a** to allyl-TMS under the catalytic Ca­(NTf_2_)_2_/*n*BuNPF_6_ system in DCM at room temperature afforded the desired allylated product in 11%, with the remaining mass balance of unreacted starting material (Entry **1**). Increasing the reaction temperature to 40 °C gave a marginal increase in yield, although conversion of the starting material remained low (Entry **2**). Switching to higher boiling solvents to facilitate reactions at 80 °C, no improvement in yield was observed in EtOAc (Entry **3**), whereas MeCN did give a marked increase in yield (Entry **4**). Switching to dimethylcarbonate (DMC), a reported green solvent alternative for MeCN,^19^ saw a yield drop again (Entry **5**). Trialing the reaction in HFIP, which becomes highly Lewis acidic in the presence of Ca­(NTf_2_)_2_,^20^ did give full conversion of the starting material but gave a poor yield of the desired product. Finally, 1,2-DCE was screened as a reaction solvent, reproducibly providing the desired product **2a** in excellent yield (Entries **7** and **10**). Lowering the catalyst loading was found to be detrimental to the yield, with no reaction occurring in its absence (Table 1).

**Table 1:**
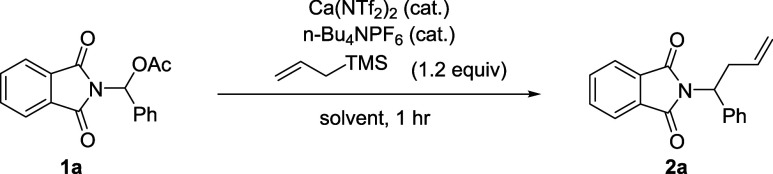
Optimization Study

Entry	Solvent	Cat. loading (mol %)	Temp (°C)	Yield^a^
1	DCM	5	r.t.	11%
2	DCM	5	40	18%
3	EtOAc	5	80	13%
4	MeCN	5	80	58%
5	DMC	5	80	22%
6	HFIP	5	80	19%
7	1,2-DCE	5	80	91%
8	1,2-DCE	0	80	n.r.
9	1,2-DCE	2	80	45%
**10**	**1,2-DCE**	**5**	**80**	**84%** ^b^

a NMR yields using nitromethane as an internal standard.

b Isolated yield.

With these conditions in hand, we set about characterizing a substrate scope, initially focusing on allylation reactions (Table 2). The reaction was tolerant to the majority of substrates that we subjected it to. Aryl rings bearing electron-donating and -withdrawing groups in various substitution patterns all worked well, with the notable exception of strongly electron-donating groups (**2e**, **2j**, **2k**), which returned the desired products in moderate yields. Furan was only moderately tolerated (**2m**), possibly due to undesirable interactions between the oxygen-containing heterocycle and oxophilic Ca catalyst; however, the thiophene and cyclohexyl motifs worked in excellent yields (**2n**, **2o**).

**Table 2:**
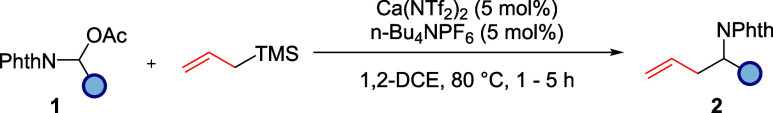

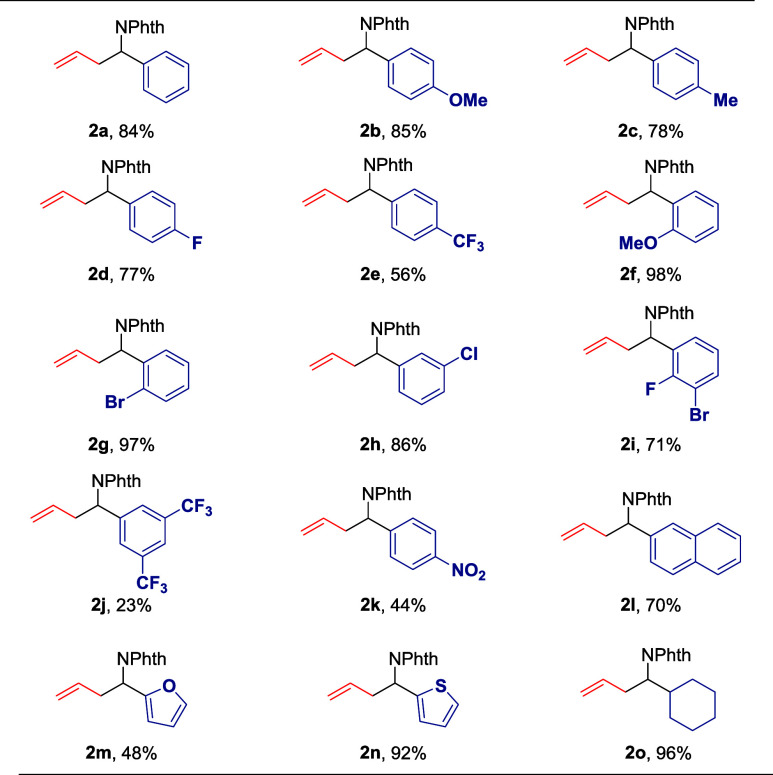
Allylation Substrate Scope^a^

a Isolated yields.

We next moved onto establishing how heteroatomic nucleophiles behaved in the system (Table 3). As shown, sulfur (**3a**–**3f**) and indole (**3g**–**k**) provided the deacetylated products in high yields (64–99%), with differing electronics and substitution patterns being well tolerated across the board. Electron-poor substrates that had lower yields with allyl-TMS (**2j**, **2k**) gave much higher yields with sulfur (**3c)** and indole (**3j**) nucleophiles. Amines were much more challenging, with simple aliphatic/aromatic amines all failing to provide the product, most likely due to premature deprotection of the Phth group by the nucleophilic amines. Moving onto amides proved more successful, with a range of amides synthesized in moderate but synthetically useful yields. Cbz-NH_2_ was an excellent nucleophile, providing an orthogonally protected bisamine (**3o**) in an effectively quantitative yield.

**Table 3:**
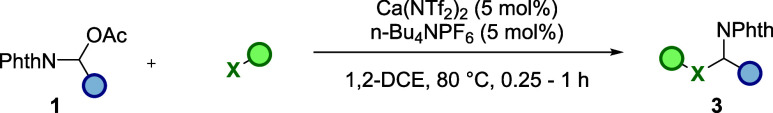

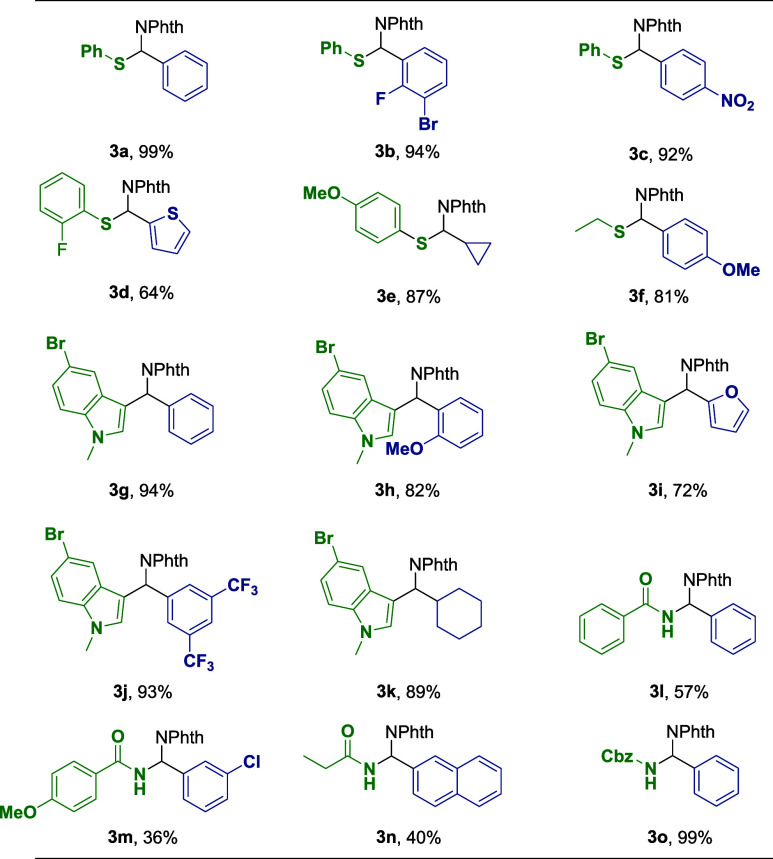
Further Nucleophile Screen^a^

a Isolated yields.

A final survey of nucleophilic partners was conducted using TMSCN, given the usefulness of the products toward further functionalization.^21^ As such, a range of our protected *O*-acyl-*N*,*O-*acetals were subjected to the calcium system in the presence of 2 equiv of TMSCN, which resulted in clean conversion to the product with concomitant high yields in most cases (Table 4). Again, the reaction was generally tolerant to electron-withdrawing (**4b**–**4e**) and -donating groups (**4f**–**4h**), heterocyclic scaffolds (**4i**, **4j**), and aliphatic motifs (**4l**, **4m**)

**Table 4:**
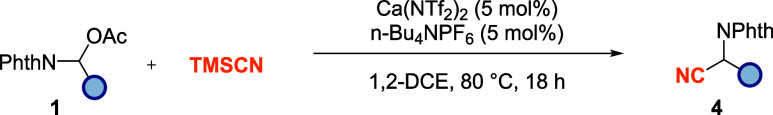

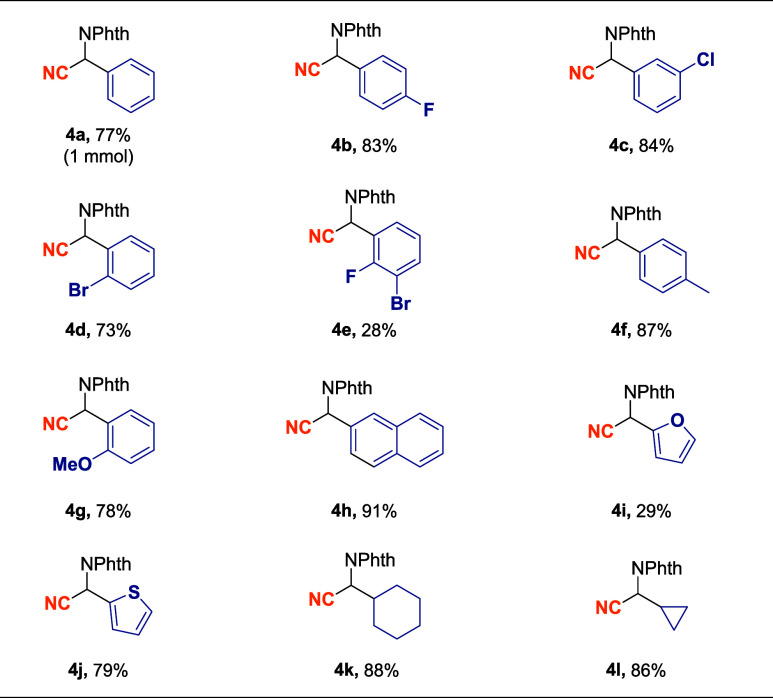
TMSCN as a Nucleophile^a^

a Isolated yields.

Although phthalimide is a useful protecting group,^22,23^ we reasoned that installation of a more well-known and encountered protecting group on nitrogen would render our methodology more valuable to the community. A three-step deoxyfunctionalization–deprotection–reprotection strategy was therefore envisaged. Additionally, we decided that telescoping this series of steps would render the process amenable to a range of amines. As such, after surveying traditional phthalimide deprotections, MeNH_2_ in EtOH proved to be the most robust. Taking this into account, the three-step procedure was carried out, producing the Boc protecting amines in good to excellent yields (over three steps) throughout (Table 5).

**Table 5:**
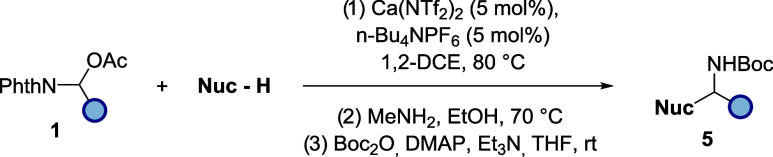

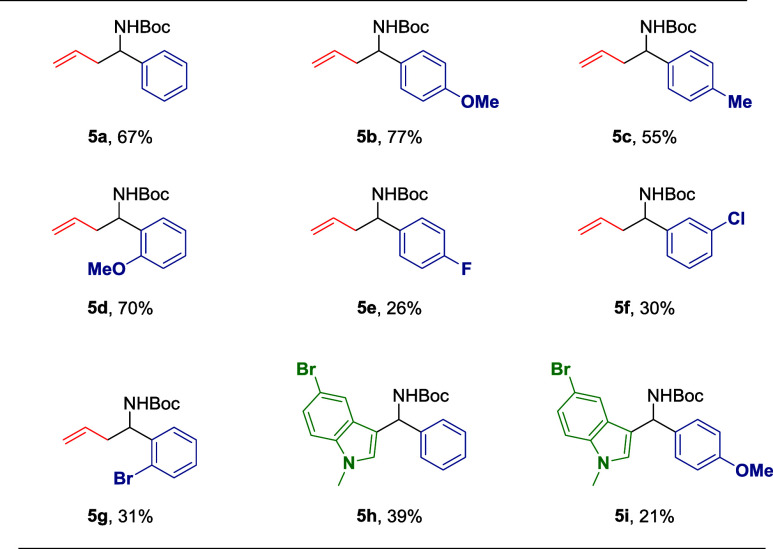
N-Boc Substrate Scope^a^

a Yield is combined yield over three steps of the isolated N-Boc product.

Given the success of this strategy, we then wanted to install other well-known amine protecting groups using the same telescoped process (Table 6). As such, Fmoc (**6a**), Bz (**6b, 6h**), Alloc (**6c, 6i**), Ac (**6d, 6g**), and Cbz (**6e, 6f**) protecting groups were all successfully deployed in excellent yields (over three steps) to once again provide a small library of useful protected amines.

**Table 6:**
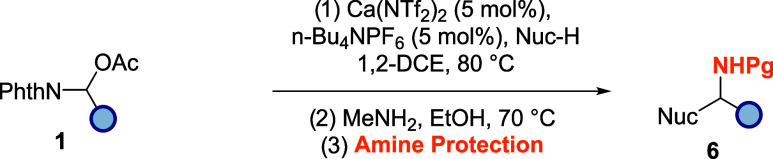

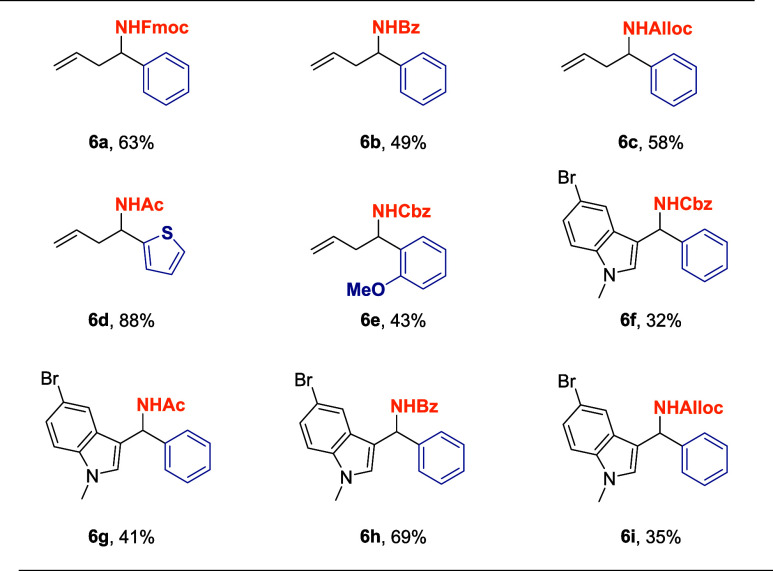
N-Protecting Group Scope^a^

a Yield is combined yield over three steps of the isolated N-protected product.

We hypothesize that the reaction proceeds via the mechanism presented in Scheme 2. There is evidence to suggest that in a 1:1 mixture of Ca­(NTf_2_)_2_ and *n*Bu_4_NPF_6_, an anion metathesis occurs to give **A**,^24^ which we postulate is the active catalyst species. The oxophilic Ca^2+^ cation of **A** then initiates the deacetylation of **1**, generating *N*-acyliminium ion **2**, which is subsequently attacked by the desired nucleophile, forming adduct **3**. The AcO anion of species **B** then deprotonates/desilylates **3**, yielding the α-functionalized Phth-N amine **4** and regenerating the active catalyst **A**, with AcOH or AcOTMS as a byproduct.

**Scheme 2:**
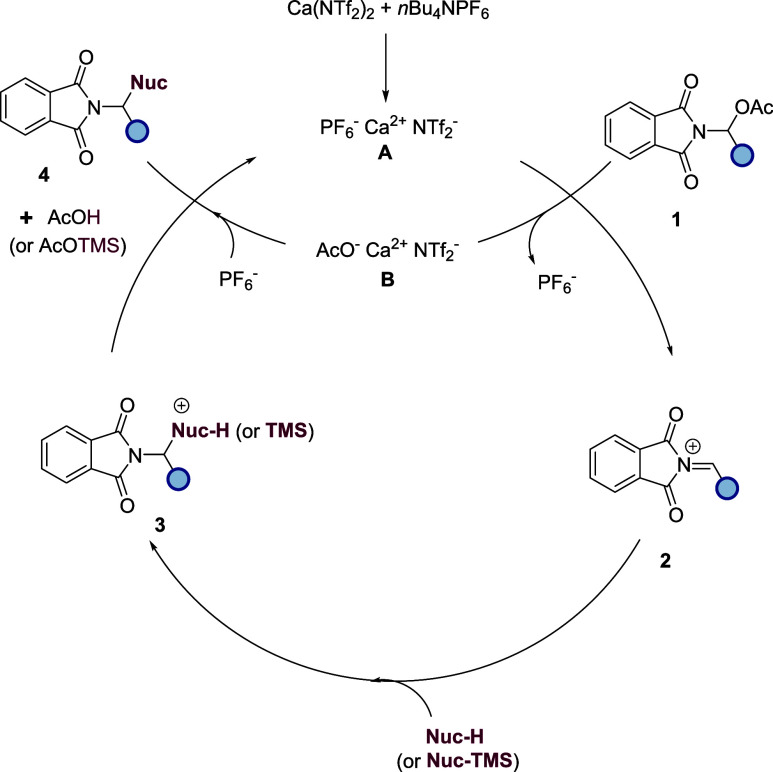
Proposed Mechanism

## Conclusions

In summary, we have demonstrated a general and efficient calcium-catalyzed methodology for the preparation of α-functionalized amines through deacetylation. This operationally straightforward process proceeds under mild, moisture-tolerant conditions, offering a broad substrate scope and compatibility with multiple nucleophiles. The telescoped deprotection–reprotection sequence further enhances the synthetic versatility of the approach, enabling the preparation of diverse *N*-protected amine libraries in excellent overall yields. Given the abundance and low toxicity of calcium catalysts, this strategy provides a practical and sustainable alternative to traditional transition metal-mediated α-functionalization methods with significant potential for applications in medicinal and synthetic chemistry.

## Experimental Section

### Solvents and Reagents

Solvents and reagents were purchased in the highest purity available from Acros Organics, Alfa Aesar, Fluorochem, TCI, Fisher Scientific, or Merck. All solvents were purchased from commercial sources and used without purification (reagent grade). Metal salts and ligands were stored in a desiccator when not in use. The anhydrous solvent was prepared by storing the solvent over activated 4Å MS for 72 h. Standard vacuum line techniques were used, and glassware was oven-dried prior to use. Organic solvents were dried during workup by using anhydrous MgSO_4_. All reactions were performed using DrySyn heating mantles and pressure-regulated vials or round-bottom flasks.

### Purification and Chromatography

Thin layer chromatography (TLC) was carried out using aluminum plates coated with 60 F_254_ silica gel. Plates were visualized using UV light (254 or 365 nm) and developed with iodine, basic permanganate solution, or ninhydrin. Flash column chromatography (FCC) was performed on Fluorochem Silica gel 60, 40–63 μm RE as the stationary phase, and the solvents employed were reagent grade.

### Characterization

^1^H NMR spectroscopic data were obtained at 400 MHz (Bruker Ultrashield 400 Plus) or 600 MHz (Bruker Ultrashield 600 Plus), and ^13^C NMR data were obtained at 100 MHz (Bruker Ultrashield 400 Plus) or 151 MHz (Bruker Ultrashield 600 Plus) at 298 K. Infrared spectra were recorded on an Agilent Technologies Cary 630 FTIR spectrometer. High-resolution mass spectrometry data were recorded using electron spray ionization (ESI) on an Agilent 6560 Ion Mobility LC/Q-TOF mass spectrometer.

#### 2-(1-Phenylbut-3-en-1-yl)­isoindoline-1,3-dione (**2a**)

To a 22 mL screw top vial were added (1,3-dioxoisoindolin-2-yl)­(phenyl)­methyl acetate (100 mg, 0.339 mmol), Ca­(NTf_2_)_2_ (10 mg, 0.017 mmol), *n*Bu_4_NPF_6_ (6.5 mg, 0.017 mmol), and allyl-TMS (46 μL, 0.406 mmol) in 1,2-DCE (1.69 mL), reacting for 1 h and then being isolated by FCC (1:12 EtOAc:Hex) as a colorless viscous oil (79 mg, 84%). RF (1:3 EtOAc:Hex): 0.74. ^1^H NMR (400 MHz, CDCl_3_) δ 7.81–7.75 (m, 2H), 7.68–7.63 (m, 2H), 7.57–7.52 (m, 2H), 7.37–7.30 (m, 2H), 7.29–7.23 (m, 1H), 5.77 (dddd, *J* = 17.1, 10.2, 8.3, 5.6 Hz, 1H), 5.45 (dd, *J* = 10.6, 5.9 Hz, 1H), 5.14 (ddd, *J* = 17.1, 2.9, 1.7 Hz, 1H), 5.01 (d, *J* = 10.1 Hz, 1H), 3.48–3.36 (m, 1H), 3.02–2.91 (m, 1H). ^13^C­{H} NMR (101 MHz, CDCl_3_) δ: 168.3, 139.2, 134.4, 133.9, 131.8, 128.6, 128.1, 127.9, 123.2, 118.3, 54.4, 35.3. Data in accordance with literature.^25^

#### 2-(1-(4-Methoxyphenyl)­but-3-en-1-yl)­isoindoline-1,3-dione (**2b**)

To a 22 mL screw top vial were added (1,3-dioxoisoindolin-2-yl)­(4-methoxyphenyl)­methyl acetate (75 mg, 0.231 mmol), Ca­(NTf_2_)_2_ (6.9 mg, 0.012 mmol), *n*Bu_4_NPF_6_ (4.5 mg, 0.012 mmol), and allyl-TMS (44 μL, 0.277 mmol) in 1,2-DCE (1.2 mL), reacting for 1 h and then being isolated by FCC (1:9 EtOAc:Hex) as a colorless viscous oil (60 mg, 85%). RF (1:4 EtOAc:Hex): 0.56. ^1^H NMR (400 MHz, CDCl_3_) δ 7.82–7.74 (m, 2H), 7.71–7.62 (m, 2H), 7.55–7.45 (m, 2H), 6.92–6.82 (m, 2H), 5.76 (dddd, *J* = 17.0, 10.2, 8.2, 5.7 Hz, 1H), 5.39 (dt, *J* = 17.1, 8.5 Hz, 1H), 5.13 (dd, *J* = 17.1, 1.2 Hz, 1H), 5.00 (d, *J* = 10.1 Hz, 1H), 3.77 (s, 3H), 3.44–3.31 (m, 1H), 3.01–2.89 (m, 1H).^13^C­{H} NMR (101 MHz, CDCl_3_) δ 168.3, 159.2, 134.5, 133.9, 131.9, 131.4, 129.4, 123.2, 118.1, 113.8, 55.2, 53.9, 35.5. Data in accordance with literature.^25^

#### 2-(1-(P-tolyl)­but-3-en-1-yl)­isoindoline-1,3-dione (**2c**)

To a 22 mL screw top vial were added (1,3-dioxoisoindolin-2-yl)­(p-tolyl)­methyl acetate (75 mg, 0.242 mmol), Ca­(NTf_2_)_2_ (7.3 mg, 0.012 mmol), *n*Bu_4_NPF_6_ (4.7 mg, 0.012 mmol), and allyl-TMS (46 μL, 0.290 mmol) in 1,2-DCE (1.2 mL), reacting for 1 h and then being isolated by FCC (1:12 EtOAc:Hex) as a colorless viscous oil (55 mg, 78%). RF (1:4 EtOAc:Hex): 0.68. ^1^H NMR (400 MHz, CDCl_3_) δ 7.82–7.75 (m, 2H), 7.69–7.63 (m, 2H), 7.43 (d, *J* = 8.1 Hz, 2H), 7.13 (d, *J* = 8.0 Hz, 2H), 5.77 (dddd, *J* = 17.0, 10.2, 8.3, 5.7 Hz, 1H), 5.41 (dd, *J* = 10.5, 5.9 Hz, 1H), 5.13 (dd, *J* = 17.1, 1.2 Hz, 1H), 5.00 (d, *J* = 10.1 Hz, 1H), 3.45–3.33 (m, 1H), 3.01–2.91 (m, 1H), 2.30 (s, 3H). ^13^C­{H} NMR (101 MHz, CDCl_3_) δ: 168.3, 137.6, 136.2, 134.5, 133.9, 131.9, 129.2, 128.0, 123.2, 118.1, 54.1, 35.4, 21.1. IR ν_max_ (cm^–1^): 2961, 2918, 1766, 1705, 1382, 1351, 1326, 1071, 713. HRMS (ESI) *m*/*z*: [M + H]+ Calcd for C_19_H_18_NO_2_ 292.1338; Found 292.1333.

#### 2-(1-(4-Fluorophenyl)­but-3-en-1-yl)­isoindoline-1,3-dione (**2d**)

To a 22 mL screw top vial were added (1,3-dioxoisoindolin-2-yl)­(4-fluorophenyl)­methyl acetate (75 mg, 0.239 mmol), Ca­(NTf_2_)_2_ (7.2 mg, 0.012 mmol), *n*Bu_4_NPF_6_ (4.6 mg, 0.012 mmol), and allyl-TMS (46 μL, 0.287 mmol) in 1,2-DCE (1.2 mL), reacting for 1 h and then being isolated by FCC (1:12 EtOAc:Hex) as a colorless viscous oil (54 mg, 77%). RF (1:4 EtOAc:Hex): 0.67. ^1^H NMR (400 MHz, CDCl_3_) δ 7.82–7.77 (m, 2H), 7.71–7.65 (m, 2H), 7.56–7.49 (m, 2H), 7.06–6.96 (m, 2H), 5.75 (dddd, *J* = 17.1, 10.2, 8.2, 5.7 Hz, 1H), 5.41 (dd, *J* = 10.4, 6.1 Hz, 1H), 5.13 (ddd, *J* = 17.1, 2.9, 1.6 Hz, 1H), 5.01 (d, *J* = 10.0 Hz, 1H), 3.46–3.30 (m, 1H), 3.02–2.89 (m, 1H). ^13^C­{H} NMR (101 MHz, CDCl_3_) δ 168.6 (s), 162.6 (d, *J* = 246.6 Hz), 135.3 (d, *J* = 3.3 Hz), 134.4 (s), 134.3 (s), 132.1 (s), 130.2 (d, *J* = 8.1 Hz), 123.6 (s), 118.7 (s), 115.7 (d, *J* = 21.4 Hz), 54.0 (s), 35.8 (s). ^19^F NMR (376 MHz, CDCl_3_) δ −114.22. IR ν_max_ (cm^–1^): 3075, 2922, 1768, 1705, 1507, 1384, 1351, 1328, 1223, 1071, 836, 713. HRMS (ESI) *m*/*z*: [M + H]+ Calcd for C_18_H_15_FNO_2_ 296.1087; Found 296.1078.

#### 2-(1-(4-(Trifluoromethyl)­phenyl)­but-3-en-1-yl)­isoindoline-1,3-dione (**2e**)

To a 22 mL screw top vial were added (1,3-dioxoisoindolin-2-yl)­(4-(trifluoromethyl)­phenyl)­methyl acetate (49 mg, 0.135 mmol), Ca­(NTf_2_)_2_ (4.1 mg, 0.007 mmol), *n*Bu_4_NPF_6_ (2.6 mg, 0.007 mmol), and allyl-TMS (26 μL, 0.162 mmol) in 1,2-DCE (0.68 mL), reacting overnight and then being isolated by FCC (1:12 EtOAc:Hex) as a colorless viscous oil (26 mg, 56%). RF (1:4 EtOAc:Hex): 0.68. ^1^H NMR (400 MHz, CDCl_3_) δ 7.85–7.78 (m, 2H), 7.74–7.69 (m, 2H), 7.66 (d, *J* = 8.4 Hz, 2H), 7.59 (d, *J* = 8.3 Hz, 2H), 5.76 (dddd, *J* = 17.0, 10.1, 8.2, 5.7 Hz, 1H), 5.49 (dd, *J* = 10.5, 6.0 Hz, 1H), 5.15 (dd, *J* = 17.1, 1.2 Hz, 1H), 5.04 (d, *J* = 10.1 Hz, 1H), 3.49–3.32 (m, 1H), 3.06–2.92 (m, 1H). ^13^C­{H} NMR (101 MHz, CDCl_3_) δ 168.2 (s), 143.0 (d, *J* = 1.2 Hz), 134.1 (s), 133.7 (s), 131.6 (s), 130.1 (q, *J* = 32.4 Hz), 128.5 (s), 125.6 (q, *J* = 3.8 Hz), 124.0 (q, *J* = 272.2 Hz), 123.4 (s), 118.8 (s), 53.8 (s), 35.1 (s). ^19^F NMR (376 MHz, CDCl_3_) δ −62.64. IR ν_max_ (cm^–1^): 3047, 1769, 1705, 1383, 1323, 1167, 1120, 1067, 922, 716. HRMS (ESI) *m*/*z*: [M + H]^+^ Calcd for C_19_H_15_F_3_NO_2_ 346.1055; Found 346.1046

#### 2-(1-(2-Methoxyphenyl)­but-3-en-1-yl)­isoindoline-1,3-dione (**2f**)

To a 22 mL screw top vial were added (1,3-dioxoisoindolin-2-yl)­(2-methoxyphenyl)­methyl acetate (78 mg, 0.240 mmol), Ca­(NTf_2_)_2_ (7.2 mg, 0.012 mmol), *n*Bu_4_NPF_6_ (4.6 mg, 0.012 mmol), and allyl-TMS (46 μL, 0.277 mmol) in 1,2-DCE (1.15 mL), reacting for 1 h and then being isolated by FCC (1:12 EtOAc:Hex) as a colorless viscous oil (72 mg, 98%). RF (1:4 EtOAc:Hex): 0.54. ^1^H NMR (400 MHz, CDCl_3_) δ 7.83–7.74 (m, 2H), 7.71–7.62 (m, 3H), 7.33–7.20 (m, 1H), 6.97 (td, *J* = 7.6, 0.8 Hz, 1H), 6.84 (d, *J* = 8.2 Hz, 1H), 5.11 (dd, *J* = 17.1, 1.1 Hz, 1H), 4.99 (d, *J* = 10.2 Hz, 1H), 3.78 (s, 3H), 3.39–3.25 (m, 1H), 2.93–2.81 (m, 1H). ^13^C­{H} NMR (101 MHz, CDCl_3_) δ: 168.3, 156.9, 134.7, 133.7, 131.9, 128.9, 128.9, 126.9, 123.1, 120.2, 118.0, 110.4, 55.5, 48.0, 35.2. IR ν_max_ (cm^–1^): 2978, 2946, 1763, 1699, 1494, 1466, 1436, 1390, 1358, 1254, 1120, 911, 721. HRMS (ESI) *m*/*z*: [M + H]+ Calcd for C_19_H_18_NO_3_ 308.1287; Found 308.1281.

#### 2-(1-(2-Bromophenyl)­but-3-en-1-yl)­isoindoline-1,3-dione (**2g**)

To a 22 mL screw top vial were added (2-bromophenyl)­(1,3-dioxoisoindolin-2-yl)­methyl acetate (75 mg, 0.200 mmol), Ca­(NTf_2_)_2_ (6.0 mg, 0.010 mmol), *n*Bu_4_NPF_6_ (3.9 mg, 0.010 mmol), and allyl-TMS (38 μL, 0.24 mmol) in 1,2-DCE (1.00 mL), reacting for 5 h and and then being isolated by FCC (1:12 EtOAc:Hex) as a colorless viscous oil (69 mg, 97%). RF (1:4 EtOAc:Hex): 0.56. ^1^H NMR (400 MHz, CDCl_3_) δ 7.86–7.77 (m, 3H), 7.72–7.66 (m, 2H), 7.55 (dd, *J* = 8.0, 1.3 Hz, 1H), 7.33 (td, *J* = 7.7, 1.2 Hz, 1H), 7.14 (td, *J* = 7.7, 1.6 Hz, 1H), 5.91–5.74 (m, 2H), 5.13 (ddd, *J* = 17.1, 2.7, 1.5 Hz, 1H), 5.02 (d, *J* = 10.1 Hz, 1H), 3.39–3.23 (m, 1H), 2.97–2.84 (m, 1H). ^13^C­{H} NMR (101 MHz, CDCl_3_) δ: 168.2, 137.9, 134.0, 133.1, 131.7, 130.2, 129.4, 127.4, 124.0, 123.3, 118.6, 53.8, 35.9. IR ν_max_ (cm^–1^): 2916, 1770, 1705, 1381, 1347, 1079, 1008, 915, 880, 714. HRMS (ESI) *m*/*z*: [M + H]^+^ Calcd for C_18_H_15_BrNO_4_ 356.0281; Found 356.0275.

#### 2-(1-(3-Chlorophenyl)­but-3-en-1-yl)­isoindoline-1,3-dione (**2h**)

To a 22 mL screw top vial were added (3-chlorophenyl)­(1,3-dioxoisoindolin-2-yl)­methyl acetate (75 mg, 0.227 mmol), Ca­(NTf_2_)_2_ (6.6 mg, 0.011 mmol), *n*Bu_4_NPF_6_ (4.3 mg, 0.011 mmol), and allyl-TMS (43 μL, 0.272 mmol) in 1,2-DCE (1.10 mL), reacting for 5 h and then being isolated by FCC (1:12 EtOAc:Hex) as a colorless viscous oil (61 mg, 86%). RF (1:4 EtOAc:Hex): 0.56 ^1^H NMR (400 MHz, CDCl_3_) δ 7.84–7.77 (m, 2H), 7.73–7.66 (m, 2H), 7.55–7.51 (m, 1H), 7.46–7.40 (m, 1H), 7.30–7.22 (m, 2H), 5.75 (dddd, *J* = 17.1, 10.2, 8.2, 5.7 Hz, 1H), 5.40 (dd, *J* = 10.6, 5.9 Hz, 1H), 5.14 (ddd, *J* = 17.1, 2.8, 1.6 Hz, 1H), 5.02 (d, *J* = 9.8 Hz, 1H), 3.44–3.31 (m, 1H), 3.00–2.89 (m, 1H). ^13^C­{H} NMR (101 MHz, CDCl_3_) δ: 168.1, 141.1, 134.4, 134.1, 133.9, 131.7, 129.8, 128.3, 128.1, 126.2, 123.3, 118.6, 53.8, 35.2. IR ν_max_ (cm^–1^): 3065, 2920, 1770, 1705, 1381, 1349, 1071, 915, 714. HRMS (ESI) *m*/*z*: [M + H]^+^ Calcd for C_18_H_14_BrFNO_2_ 312.0786; Found 312.0782.

#### 2-(1-(3-Bromo-2-fluorophenyl)­but-3-en-1-yl)­isoindoline-1,3-dione (**2i**)

To a 22 mL screw top vial were added (3-bromo-2-fluorophenyl)­(1,3-dioxoisoindolin-2-yl)­methyl acetate (75 mg, 0.191 mmol), Ca­(NTf_2_)_2_ (5.7 mg, 0.010 mmol), *n*Bu_4_NPF_6_ (3.7 mg, 0.010 mmol), and allyl-TMS (36 μL, 0.23 mmol) in 1,2-DCE (0.96 mL), reacting overnight and then being isolated by FCC (1:9 EtOAc:Hex) as a white solid (51 mg, 71%). RF (1:4 EtOAc:Hex): 0.61. ^1^H NMR (400 MHz, CDCl_3_) δ 7.84–7.78 (m, 2H), 7.74–7.65 (m, 3H), 7.52–7.44 (m, 1H), 7.05 (t, *J* = 7.9 Hz, 1H), 5.84–5.71 (m, 2H), 5.14 (dd, *J* = 17.1, 1.0 Hz, 1H), 5.04 (d, *J* = 10.1 Hz, 1H), 3.39–3.24 (m, 1H), 2.97–2.85 (m, 1H). ^13^C­{H} NMR (101 MHz, CDCl_3_) δ 167.9 (s), 156.8 (d, *J* = 248.7 Hz), 134.1 (s), 133.5 (s), 133.1 (s), 131.6 (s), 128.8 (d, *J* = 2.9 Hz), 127.5 (d, *J* = 14.7 Hz), 124.9 (d, *J* = 4.5 Hz), 123.4 (s), 118.8 (d, *J* = 21.0 Hz), 109.3 (d, *J* = 21.7 Hz), 47.2 (d, *J* = 2.6 Hz), 35.0 (s). ^19^F NMR (376 MHz, CDCl_3_) δ −109.65. IR ν_max_ (cm^–1^): 3082, 2924, 1770, 1709, 1450, 1385, 1347, 1064, 928, 714. HRMS (ESI) *m*/*z*: [M + H]+ Calcd for C_18_H_14_BrFNO_2_ 374.0186; Found 374.0180.

#### 2-(1-(3,5-Bis­(trifluoromethyl)­phenyl)­but-3-en-1-yl)­isoindoline-1,3-dione (**2j**)

To a 22 mL screw top vial were added (3,5-bis­(trifluoromethyl)­phenyl)­(1,3-dioxoisoindolin-2-yl)­methyl acetate (100 mg, 0.232 mmol), Ca­(NTf_2_)_2_ (7.0 mg, 0.012 mmol), *n*Bu_4_NPF_6_ (4.5 mg, 0.012 mmol), and allyl-TMS (44 μL, 0.278 mmol) in 1,2-DCE (1.16 mL), reacting overnight and then being isolated by FCC (1:12 EtOAc:Hex) as a colorless viscous oil (22 mg, 23%). RF (1:4 EtOAc:Hex): 0.61. ^1^H NMR (400 MHz, CDCl_3_) δ 8.01 (s, 2H), 7.87–7.79 (m, 3H), 7.77–7.70 (m, 2H), 5.81–5.68 (m, 1H), 5.53 (dd, *J* = 10.4, 6.0 Hz, 1H), 5.17 (d, *J* = 17.0 Hz, 1H), 5.06 (d, *J* = 10.2 Hz, 1H), 3.47–3.36 (m, 1H), 3.05–2.94 (m, 1H). ^13^C­{H} NMR (101 MHz, CDCl_3_) δ 168.0 (s), 141.5 (s), 134.3 (s), 133.1 (s), 131.9 (q, *J* = 33.4 Hz), 131.5 (s), 128.6 (d, *J* = 2.6 Hz), 123.6 (s), 127.7–118.6 (m), 122.2–121.9 (m), 119.3 (s), 53.3 (s), 35.2 (s). ^19^F NMR (376 MHz, CDCl_3_) δ −62.80. IR ν_max_ (cm^–1^): 3080, 1765, 1703, 1381, 1351, 1274, 1128, 924, 893, 721, 699. HRMS (ESI) *m*/*z*: [M + H]+ Calcd for C_20_H_14_F_6_NO_2_ 414.0915; Found 414.092.

#### 2-(1-(4-Nitrophenyl)­but-3-en-1-yl)­isoindoline-1,3-dione (**2k**)

To a 22 mL screw top vial were added (1,3-dioxoisoindolin-2-yl)­(4-nitrophenyl)­methyl acetate (75 mg, 0.22 mmol), Ca­(NTf_2_)_2_ (6.6 mg, 0.011 mmol), *n*Bu_4_NPF_6_ (4.3 mg, 0.011 mmol), and allyl-TMS (30 μL, 0.264 mmol) in 1,2-DCE (1.10 mL), reacting overnight and then being isolated by FCC (1:6 EtOAc:Hex) as a colorless viscous oil (31 mg, 44%).**b**RF (1:4 EtOAc:Hex): 0.56. ^1^H NMR (400 MHz, CDCl_3_) δ 8.19 (d, *J* = 8.8 Hz, 2H), 7.88–7.77 (m, 2H), 7.78–7.66 (m, 4H), 5.83–5.69 (m, 1H), 5.52 (dd, *J* = 10.4, 6.1 Hz, 1H), 5.16 (dd, *J* = 17.1, 0.9 Hz, 1H), 5.05 (d, *J* = 10.1 Hz, 1H), 3.45–3.32 (m, 1H), 3.07–2.95 (m, 1H). ^13^C­{H} NMR (101 MHz, CDCl_3_) δ: 168.1, 147.5, 146.1, 134.3, 133.3, 131.5, 129.1, 123.8, 123.5, 119.1, 53.5, 35.1. IR ν_max_ (cm^–1^): 3460, 3080, 1770, 1705, 1599, 1509, 1382, 1345, 1077, 993, 851, 721. HRMS (ESI) *m*/*z*: [M + H]+ Calcd for C_18_H_15_N_2_O_4_ 323.1026; Found 323.1022

#### 2-(1-(Naphthalen-2-yl)­but-3-en-1-yl)­isoindoline-1,3-dione (**2l**)

To a 22 mL screw top vial were added (1,3-dioxoisoindolin-2-yl)­(naphthalen-2-yl)­methyl acetate (100 mg, 0.290 mmol), Ca­(NTf_2_)_2_ (9.0 mg, 0.015 mmol), *n*Bu_4_NPF_6_ (5.8 mg, 0.015 mmol), and allyl-TMS (55 μL, 0.35 mmol) in 1,2-DCE (1.50 mL), reacting for 1 h and then being isolated by FCC (1:12 EtOAc:Hex) as a colorless viscous oil (66 mg, 70%). RF (1:4 EtOAc:Hex): 0.60. ^1^H NMR (400 MHz, CDCl_3_) δ 8.01 (s, 1H), 7.88–7.77 (m, 5H), 7.73–7.62 (m, 3H), 7.50–7.42 (m, 2H), 5.84 (dddd, *J* = 17.1, 10.2, 8.2, 5.7 Hz, 1H), 5.63 (dd, *J* = 10.5, 6.0 Hz, 1H), 5.20 (dd, *J* = 17.1, 1.2 Hz, 1H), 5.05 (d, *J* = 10.1 Hz, 1H), 3.60–3.46 (m, 1H), 3.19–3.05 (m, 1H). ^13^C­{H} NMR (101 MHz, CDCl_3_) δ: 168.6, 136.8, 134.6, 134.1, 133.4, 133.1, 132.0, 128.6, 128.4, 127.8, 127.3, 126.4, 126.4, 126.2, 123.4, 118.5, 54.8, 35.6. IR ν_max_ (cm^–1^): 3047, 2955, 2909, 1768, 1701, 1382, 1351, 1328, 1077, 923, 713. HRMS (ESI) *m*/*z*: [M + H]+ Calcd for C_22_H_18_NO_2_ 328.1338; Found 328.1328

#### 2-(1-(Furan-2-yl)­but-3-en-1-yl)­isoindoline-1,3-dione (**2m**)

To a 22 mL screw top vial were added (1,3-dioxoisoindolin-2-yl)­(furan-2-yl)­methyl acetate (75 mg, 0.263 mmol), Ca­(NTf_2_)_2_ (7.8 mg, 0.013 mmol), *n*Bu_4_NPF_6_ (5.0 mg, 0.013 mmol), and allyl-TMS (50 μL, 0.32 mmol) in 1,2-DCE (1.30 mL), reacting for 1 h and then being isolated by FCC (1:9 EtOAc:Hex) as a colorless viscous oil (34 mg, 48%). RF (1:4 EtOAc:Hex): 0.60. ^1^H NMR (400 MHz, CDCl_3_) δ 7.85–7.79 (m, 2H), 7.73–7.67 (m, 2H), 7.32 (d, *J* = 1.1 Hz, 1H), 6.40 (d, *J* = 3.3 Hz, 1H), 6.33 (dd, *J* = 3.3, 1.8 Hz, 1H), 5.77 (dddd, *J* = 17.0, 10.2, 8.3, 5.7 Hz, 1H), 5.50 (dd, *J* = 10.3, 5.8 Hz, 1H), 5.14 (ddd, *J* = 17.1, 2.8, 1.6 Hz, 1H), 5.03 (d, *J* = 10.0 Hz, 1H), 3.28–3.17 (m, 1H), 3.03–2.93 (m, 1H). ^13^C­{H} NMR (101 MHz, CDCl_3_) δ: 167.8, 151.9, 141.9, 134.0, 133.5, 131.8, 123.3, 118.7, 110.4, 107.7, 47.7, 34.4. IR ν_max_ (cm^–1^): 3118, 2924, 1772, 1707, 1377, 1355, 1135, 1084, 1008, 934, 876, 713. HRMS (ESI) *m*/*z*: [M + H]+ Calcd for C_16_H_14_NO_3_ 268.0968; Found 268.0962

#### 2-(1-(Thiophen-2-yl)­but-3-en-1-yl)­isoindoline-1,3-dione (**2n**)

To a 22 mL screw top vial were added (1,3-dioxoisoindolin-2-yl)­(thiophen-2-yl)­methyl acetate (75 mg, 0.249 mmol), Ca­(NTf_2_)_2_ (7.5 mg, 0.013 mmol), *n*Bu_4_NPF_6_ (4.8 mg, 0.013 mmol), and allyl-TMS (47 μL, 0.30 mmol) in 1,2-DCE (1.2 mL), reacting for 1 h and then being isolated by FCC (1:9 EtOAc:Hex) as a colorless viscous oil (65 mg, 92%). RF (1:4 EtOAc:Hex): 0.59. ^1^H NMR (400 MHz, CDCl_3_) δ 7.86–7.77 (m, 2H), 7.73–7.65 (m, 2H), 7.22 (dd, *J* = 5.1, 1.0 Hz, 1H), 7.16 (d, *J* = 3.5 Hz, 1H), 6.94 (dd, *J* = 5.1, 3.6 Hz, 1H), 5.81–5.71 (m, 1H), 5.68 (dd, *J* = 10.4, 6.1 Hz, 1H), 5.14 (dd, *J* = 17.1, 1.2 Hz, 1H), 5.02 (d, *J* = 10.1 Hz, 1H), 3.46–3.31 (m, 1H), 3.09–2.93 (m, 1H). ^13^C­{H} NMR (101 MHz, CDCl_3_) δ: 167.8, 142.0, 134.0, 133.8, 131.7, 126.6, 126.3, 125.3, 123.3, 118.6, 49.6, 37.1. IR ν_max_ (cm^–1^): 3076, 1768, 1701, 1466, 1371, 1347, 1325, 1232, 1067, 916, 708. HRMS (ESI) *m*/*z*: [M + H]^+^ Calcd for C_16_H_14_NO_2_S 284.0740; Found 284.0735

#### 2-(1-Cyclohexylbut-3-en-1-yl)­isoindoline-1,3-dione (**2o**)

To a 22 mL screw top vial were added cyclohexyl­(1,3-dioxoisoindolin-2-yl)­methyl acetate (75 mg, 0.250 mmol), Ca­(NTf_2_)_2_ (7.5 mg, 0.013 mmol), *n*Bu_4_NPF_6_ (4.8 mg, 0.013 mmol), and allyl-TMS (47 μL, 0.30 mmol) in 1,2-DCE (1.2 mL), reacting overnight and then being isolated by FCC (1:15 EtOAc:Hex) as a colorless viscous oil (68 mg, 96%). RF (1:4 EtOAc:Hex): 0.72. ^1^H NMR (400 MHz, CDCl_3_) δ 7.85–7.77 (m, 2H), 7.74–7.66 (m, 2H), 5.65 (dtd, *J* = 16.9, 9.6, 5.2 Hz, 1H), 4.98 (d, *J* = 17.0 Hz, 1H), 4.88 (d, *J* = 10.1 Hz, 1H), 4.07–3.94 (m, 1H), 2.90–2.74 (m, 1H), 2.66–2.52 (m, 1H), 2.16–2.03 (m, 1H), 1.99–1.89 (m, 1H), 1.82–1.72 (m, 1H), 1.70–1.60 (m, 2H), 1.59–1.51 (m, 1H), 1.36–0.86 (m, 5H). ^13^C­{H} NMR (101 MHz, CDCl_3_) δ: 168.8, 135.1, 133.8, 131.7, 123.1, 117.6, 56.9, 39.2, 33.8, 30.7, 30.3, 26.2, 25.8, 25.7. Data in accordance with literature.^25^

#### 2-(Phenyl­(phenylthio)­methyl)­isoindoline-1,3-dione (**3a**)

To a 22 mL screw top vial were added (1,3-dioxoisoindolin-2-yl)­(phenyl)­methyl acetate (50 mg, 0.169 mmol), Ca­(NTf_2_)_2_ (5.1 mg, 0.009 mmol), *n*Bu_4_NPF_6_ (3.3 mg, 0.009 mmol), and thiophenol (21 μL, 0.203 mmol) in 1,2-DCE (0.85 mL), reacting for 15 min and then being isolated by FCC (1:6 EtOAc:Hex) as a colorless viscous oil (58 mg, 99%). RF (1:4 EtOAc:Hex): 0.46. ^1^H NMR (400 MHz, CDCl_3_) δ 7.80–7.74 (m, 2H), 7.73–7.64 (m, 4H), 7.50–7.43 (m, 2H), 7.39–7.28 (m, 3H), 7.24–7.19 (m, 3H), 6.74 (s, 1H). ^13^C­{H} NMR (101 MHz, CDCl_3_) δ: 166.8, 136.8, 134.2, 133.5, 133.1, 131.5, 129.2, 128.6, 128.3, 128.1, 123.5, 61.0. IR ν_max_ (cm^–1^): 3058, 1764, 1712, 1375, 1343, 1310, 1068, 889, 695. HRMS (ESI) *m*/*z*: [M–PhS]^+^ Calcd for C_15_H_10_N_2_O_4_ 236.0706; Found 236.0711.

#### 2-((3-Bromo-2-fluorophenyl)­(phenylthio)­methyl)­isoindoline-1,3-dione (**3b**)

To a 22 mL screw top vial were added (3-bromo-2-fluorophenyl)­(1,3-dioxoisoindolin-2-yl)­methyl acetate (75 mg, 0.191 mmol), Ca­(NTf_2_)_2_ (5.7 mg, 0.010 mmol), *n*Bu_4_NPF_6_ (3.7 mg, 0.010 mmol), and thiophenol (23 μL, 0.229 mmol) in 1,2-DCE (0.96 mL), reacting for 15 min and then being isolated by FCC (1:6 EtOAc:Hex) as a colorless viscous oil (79 mg, 94%). RF (1:4 EtOAc:Hex): 0.63. ^1^H NMR (400 MHz, CDCl_3_) δ 8.17–8.11 (m, 1H), 7.81–7.75 (m, 2H), 7.72–7.66 (m, 2H), 7.56–7.46 (m, 3H), 7.27–7.22 (m, 3H), 7.11 (td, *J* = 8.0, 1.0 Hz, 1H), 6.96 (s, 1H). ^13^C­{H} NMR (101 MHz, CDCl_3_) δ 166.3 (s), 155.9 (d, *J* = 249.3 Hz), 134.3 (s), 134.0 (s), 133.7 (s), 132.3 (s), 131.4 (s), 130.4 (d, *J* = 1.8 Hz), 129.3 (s), 128.8 (s), 125.5 (d, *J* = 14.1 Hz), 125.0 (d, *J* = 4.6 Hz), 123.6 (s), 109.1 (d, *J* = 21.4 Hz), 54.2 (d, *J* = 3.2 Hz). ^19^F NMR (376 MHz, CDCl_3_) δ −108.95 (t, *J* = 6.7 Hz). IR ν_max_ (cm^–1^): 3058, 1763, 1716, 1449, 1373, 1321, 1224, 1067, 889, 713. HRMS (ESI) *m*/*z*: [M–SPh]^+^ Calcd for C_15_H_8_NO_2_FBr 331.9717; 331.9714

#### 2-((4-Nitrophenyl)­(phenylthio)­methyl)­isoindoline-1,3-dione (**3c**)

To a 22 mL screw top vial were added (1,3-dioxoisoindolin-2-yl)­(4-nitrophenyl)­methyl acetate (75 mg, 0.220 mmol), Ca­(NTf_2_)_2_ (6.6 mg, 0.011 mmol), *n*Bu_4_NPF_6_ (4.3 mg, 0.011 mmol), and thiophenol (27 μL, 0.264 mmol) in 1,2-DCE (1.10 mL), reacting for 15 min and then being isolated by FCC (1:4 EtOAc:Hex) as an off-white solid obtained (79 mg, 92%). RF (1:3 EtOAc:Hex): 0.23. ^1^H NMR (400 MHz, CDCl_3_) δ 8.25–8.18 (m, 2H), 7.90–7.84 (m, 2H), 7.85–7.79 (m, 2H), 7.78–7.70 (m, 2H), 7.51–7.42 (m, 2H), 7.31–7.20 (m, 3H), 6.75 (s, 1H). ^13^C­{H} NMR (101 MHz, CDCl_3_) δ: 166.6, 147.8, 143.8, 134.6, 133.5, 132.5, 131.3, 129.5, 129.2, 128.9, 123.8, 123.8, 60.3. IR ν_max_ (cm^–1^): 3106, 3080, 1772, 1714, 1597, 1515, 1343, 1319, 1079, 892, 713. HRMS (ESI) *m*/*z*: [M–H]^−^ Calcd for C_21_H_13_N_2_O_4_S 389.0602; Found 389.0592.

#### 2-(((2-Fluorophenyl)­thio)­(thiophen-2-yl)­methyl)­isoindoline-1,3-dione (**3d**)

To a 22 mL screw top vial were added (1,3-dioxoisoindolin-2-yl)­(thiophen-2-yl)­methyl acetate (75 mg, 0.249 mmol), Ca­(NTf_2_)_2_ (7.5 mg, 0.012 mmol), *n*Bu_4_NPF_6_ (4.8 mg, 0.012 mmol), and 2-fluorothiophenol (32 μL, 0.299 mmol) in 1,2-DCE (1.25 mL), reacting for 15 min and then being isolated by FCC (1:6 EtOAc:Hex) as a white solid obtained (59 mg, 64%). RF (1:4 EtOAc:Hex): 0.43. ^1^H NMR (400 MHz, CDCl_3_) δ 7.84–7.78 (m, 2H), 7.73–7.68 (m, 2H), 7.46 (td, *J* = 7.5, 1.7 Hz, 1H), 7.32–7.26 (m, 3H), 7.08–7.01 (m, 1H), 7.00–6.94 (m, 2H), 6.90 (d, *J* = 0.6 Hz, 1H). ^13^C­{H} NMR (101 MHz, CDCl_3_) δ 166.3 (s), 162.8 (d, *J* = 248.0 Hz), 139.1 (s), 136.5 (s), 134.3 (s), 131.6 (d, *J* = 8.1 Hz), 131.5 (s), 127.7 (s), 126.8 (s), 126.63 (s), 124.7 (d, *J* = 3.9 Hz), 123.6 (s), 119.8 (d, *J* = 18.4 Hz), 116.1 (d, *J* = 23.0 Hz), 55.4 (d, *J* = 2.3 Hz). ^19^F NMR (376 MHz, CDCl_3_) δ −106.62 (ddd, *J* = 9.1, 7.3, and 5.1 Hz). IR ν_max_ (cm^–1^): 3067, 2924, 1764, 1714, 1466, 1353, 1325, 1218, 1081, 885, 762, 702. HRMS (ESI) *m*/*z*: [M–S­(C_6_H_5_F)]^+^ Calcd for C_13_H_8_NO_2_S 242.0270; Found 242.0270.

#### 2-(Cyclopropyl­((4-methoxyphenyl)­thio)­methyl)­isoindoline-1,3-dione (**3e**)

To a 22 mL screw top vial were added cyclopropyl­(1,3-dioxoisoindolin-2-yl)­methyl acetate (75 mg, 0.289 mmol), Ca­(NTf_2_)_2_ (8.7 mg, 0.015 mmol), *n*Bu_4_NPF_6_ (5.6 mg, 0.015 mmol), and 4-methoxythiophenol (43 μL, 0.347 mmol) in 1,2-DCE (1.45 mL), reacting for 15 min and then being isolated by FCC (1:8 EtOAc:Hex) as a pale-yellow solid obtained (85 mg, 87%). RF (1:4 EtOAc:Hex): 0.47. ^1^H NMR (400 MHz, CDCl_3_) δ 7.86–7.74 (m, 2H), 7.73–7.66 (m, 2H), 7.36–7.31 (m, 2H), 6.75–6.65 (m, 2H), 4.61 (d, *J* = 10.5 Hz, 1H), 3.71 (s, 3H), 2.11–1.99 (m, 1H), 0.90–0.78 (m, 1H), 0.64–0.52 (m, 2H), 0.39–0.30 (m, 1H). ^13^C­{H} NMR (101 MHz, CDCl_3_) δ: 167.0, 160.1, 136.6, 134.0, 131.6, 131.6, 123.3, 114.5, 64.5, 55.2, 14.2, 6.6, 5.7. IR ν_max_ (cm^–1^): 2996, 2927, 2834, 1764, 1712, 1589, 1494, 1371, 1246, 1079, 1030, 837, 710. HRMS (ESI) *m*/*z*: [M–S­(C_6_H_5_OCH_3_)]^+^ Calcd for C_12_H_10_NO_2_ 200.0706; Found 200.0706.

#### 2-((Ethylthio)­(4-methoxyphenyl)­methyl)­isoindoline-1,3-dione (**3f**)

To a 22 mL screw top vial were added (1,3-dioxoisoindolin-2-yl)­(4-methoxyphenyl)­methyl acetate (50 mg, 0.154 mmol), Ca­(NTf_2_)_2_ (4.6 mg, 0.008 mmol), *n*Bu_4_NPF_6_ (3.0 mg, 0.008 mmol), and ethanethiol (13 μL, 0.185 mmol) in 1,2-DCE (0.77 mL), reacting for 15 min and then being isolated by FCC (1:6 EtOAc:Hex) as a colorless oil obtained (41 mg, 81%). RF (1:4 EtOAc:Hex): 0.45. ^1^H NMR (400 MHz, CDCl_3_) δ 7.86–7.81 (m, 2H), 7.74–7.68 (m, 2H), 7.61–7.55 (m, 2H), 6.89–6.83 (m, 2H), 6.46 (s, 1H), 3.78 (s, 3H), 2.73–2.54 (m, 2H), 1.30 (t, *J* = 7.4 Hz, 3H). ^13^C­{H} NMR (101 MHz, CDCl_3_) δ: 167.1, 159.5, 134.2, 131.7, 129.4, 129.3, 123.5, 113.7, 56.6, 55.3, 26.6, 14.5. IR ν_max_ (cm^–1^): 2929, 2840, 1754, 1709, 1608, 1511, 1373, 1312, 1250, 1189, 1098, 1021, 891, 829, 710. HRMS (ESI) *m*/*z*: [M–SEt]^+^ Calcd for C_16_H_12_NO_3_ 266.0812; Found 266.0806.

#### 2-((5-Bromo-1-methyl-1H-indol-3-yl)­(phenyl)­methyl)­isoindoline-1,3-dione (**3g**)

To a 22 mL screw top vial were added (1,3-dioxoisoindolin-2-yl)­(phenyl)­methyl acetate (50 mg, 0.169 mmol), Ca­(NTf_2_)_2_ (5.1 mg, 0.008 mmol), *n*Bu_4_NPF_6_ (3.3 mg, 0.008 mmol), and 5-bromo-*N*-methylindole (43 mg, 0.203 mmol) in 1,2-DCE (0.85 mL), reacting for 15 min and then being isolated by FCC (1:8 to 1:6 EtOAc:Hex) as an off-white solid obtained (71 mg, 94%). RF (1:4 EtOAc:Hex): 0.31. ^1^H NMR (400 MHz, CDCl_3_) δ 7.83–7.78 (m, 2H), 7.71–7.66 (m, 2H), 7.64 (d, *J* = 1.7 Hz, 1H), 7.50–7.45 (m, 2H), 7.36–7.25 (m, 4H), 7.17–7.10 (m, 2H), 6.95 (s, 1H), 3.72 (s, 3H). ^13^C­{H} NMR (101 MHz, CDCl_3_) δ: 166.9, 137.6, 134.3, 133.0, 130.8, 130.2, 127.8, 127.4, 126.8, 126.6, 123.7, 122.4, 120.3, 112.0, 110.5, 109.9, 48.9, 32.1. IR ν_max_ (cm^–1^): 3058, 3030, 2920, 165, 1701, 1474, 1382, 1353, 1321, 1070, 894, 792, 714. HRMS (ESI) *m*/*z*: [M–C_9_H_7_NBr]^+^ Calcd for C_15_H_10_NO_2_ 236.0706 Found 236.0703

#### 2-((5-Bromo-1-methyl-1H-indol-3-yl)­(2-methoxyphenyl)­methyl)­isoindoline-1,3-dione (**3h**)

To a 22 mL screw top vial were added (1,3-dioxoisoindolin-2-yl)­(2-methoxyphenyl)­methyl acetate (75 mg, 0.231 mmol), Ca­(NTf_2_)_2_ (6.9 mg, 0.012 mmol), *n*Bu_4_NPF_6_ (4.5 mg, 0.012 mmol), and 5-bromo-*N*-methylindole (58 mg, 0.277 mmol) in 1,2-DCE (1.16 mL), reacting for 15 min and then being isolated by FCC (1:4 EtOAc:Hex) as an off-white solid obtained (90 mg, 82%). RF (1:4 EtOAc:Hex): 0.24. ^1^H NMR (400 MHz, CDCl_3_) δ 7.83–7.76 (m, 2H), 7.70–7.63 (m, 2H), 7.58 (d, *J* = 1.7 Hz, 1H), 7.47 (dd, *J* = 7.6, 1.4 Hz, 1H), 7.30–7.24 (m, 2H), 7.21 (s, 1H), 7.13 (d, *J* = 8.7 Hz, 1H), 7.09 (s, 1H), 6.88 (td, *J* = 8.3, 2.0 Hz, 2H), 3.76 (s, 3H), 3.70 (s, 3H). ^13^C­{H} NMR (101 MHz, CDCl_3_) δ: 168.1, 156.9, 135.6, 133.9, 132.0, 130.4, 130.3, 129.2, 128.6, 126.4, 124.7, 123.2, 121.6, 120.2, 112.9, 111.6, 110.9, 110.4, 55.7, 45.1, 33.0. IR ν_max_ (cm^–1^): 3052, 2961, 2920, 1772, 1705, 1476, 1354, 1239, 1107, 869, 874, 756, 728, 712. HRMS (ESI) *m*/*z*: [M–C_9_H_7_NBr]^+^ Calcd for C_16_H_12_NO_3_ 266.0812; Found 266.0811.

#### 2-((5-Bromo-1-methyl-1H-indol-3-yl)­(furan-2-yl)­methyl)­isoindoline-1,3-dione (**3i**)

To a 22 mL screw top vial were added (1,3-dioxoisoindolin-2-yl)­(furan-2-yl)­methyl acetate (75 mg, 0.263 mmol), Ca­(NTf_2_)_2_ (7.9 mg, 0.013 mmol), *n*Bu_4_NPF_6_ (5.1 mg, 0.013 mmol), and 5-bromo-*N*-methylindole (66 mg, 0.316 mmol) in 1,2-DCE (1.32 mL), reacting for 15 min and then being isolated by FCC (1:6 to 1:4 EtOAc:Hex) as a brown solid. (82 mg, 72%). RF (1:4 EtOAc:Hex): 0.29. ^1^H NMR (400 MHz, CDCl_3_) δ 7.83 (t, *J* = 3.5 Hz, 1H), 7.78 (td, *J* = 5.3, 2.1 Hz, 2H), 7.69–7.62 (m, 2H), 7.43 (s, 1H), 7.37 (t, *J* = 1.1 Hz, 1H), 7.27 (dd, *J* = 8.7, 1.8 Hz, 1H), 7.14 (d, *J* = 8.7 Hz, 1H), 6.92 (s, 1H), 6.35 (t, *J* = 2.3 Hz, 2H), 3.74 (s, 3H). ^13^C­{H} NMR (101 MHz, CDCl_3_) δ: 167.5, 151.2, 142.2, 135.2, 134.0, 131.9, 131.4, 128.4, 124.9, 123.4, 121.5, 113.3, 111.0, 110.6, 109.4, 108.9, 43.7, 33.2. IR ν_max_ (cm^–1^): 3119, 2920, 1763, 1705, 1476, 1343, 1317, 1142, 1107, 1008, 790, 728. HRMS (ESI) *m*/*z*: [M–C_9_H_7_NBr]^+^ Calcd for C_13_H_8_NO_3_ 226.0499; Found 226.0495.

#### 2-((3,5-Bis­(trifluoromethyl)­phenyl)­(5-bromo-1-methyl-1H-indol-3-yl)­methyl) isoindoline-1,3-dione (**3j**)

To a 22 mL screw top vial were added (3,5-bis­(trifluoromethyl)­phenyl)­(1,3-dioxoisoindolin-2-yl)­methyl acetate (75 mg, 0.174 mmol), Ca­(NTf_2_)_2_ (5.2 mg, 0.009 mmol), *n*Bu_4_NPF_6_ (3.4 mg, 0.009 mmol), and 5-bromo-*N*-methylindole (44 mg, 0.209 mmol) in 1,2-DCE (0.87 mL), reacting for 15 min and then being isolated by FCC (1:6 EtOAc:Hex) as a white solid. (94 mg, 93%). RF (1:4 EtOAc:Hex): 0.45. ^1^H NMR (400 MHz, CDCl_3_) δ 7.93 (s, 2H), 7.88–7.81 (m, 3H), 7.76–7.70 (m, 2H), 7.67 (d, *J* = 1.6 Hz, 1H), 7.32 (dd, *J* = 8.7, 1.8 Hz, 1H), 7.19 (d, *J* = 8.7 Hz, 1H), 7.15 (s, 1H), 7.04 (s, 1H), 3.77 (s, 3H). ^13^C­{H} NMR (101 MHz, CDCl_3_) δ 167.7 (s), 141.5 (s), 135.4 (s), 134.5 (s), 132.4–131.3 (m), 131.6 (s), 131.1 (s), 128.4 (s), 128.1 (d, *J* = 2.7 Hz), 125.3 (s), 123.7 (s), 123.2 (q, *J* = 272.8 Hz), 121.9–121.7 (m), 121.1 (s), 113.5 (s), 111.2 (s), 110.0 (s), 49.1 (s), 33.3 (s). ^19^F NMR (376 MHz, CDCl_3_) δ −62.67. IR ν_max_ (cm^–1^): 3062, 2922, 1774, 1705, 1477, 1354, 1280, 1164, 1108, 921, 794, 712. HRMS (ESI) *m*/*z*: [M + H]^+^ Calcd for C_26_H_16_ F_6_N_2_O_2_ 581.0294; Found 581.0283.

#### 2-((5-Bromo-1-methyl-1H-indol-3-yl)­(cyclohexyl)­methyl)­isoindoline-1,3-dione (**3k**)

To a 22 mL screw top vial were added cyclohexyl­(1,3-dioxoisoindolin-2-yl)­methyl acetate (75 mg, 0.242 mmol), Ca­(NTf_2_)_2_ (7.2 mg, 0.012 mmol), *n*Bu_4_NPF_6_ (4.6 mg, 0.012 mmol), and 5-bromo-*N*-methylindole (61 mg, 0.290 mmol) in 1,2-DCE (1.21 mL), reacting for 15 min and then being isolated by FCC (1:6 EtOAc:Hex) as a white solid. (97 mg, 89%). RF (1:4 EtOAc:Hex): 0.47. ^1^H NMR (400 MHz, CDCl_3_) δ 7.93 (d, *J* = 1.7 Hz, 1H), 7.78–7.72 (m, 2H), 7.65–7.59 (m, 2H), 7.40 (s, 1H), 7.25 (dd, *J* = 8.6, 1.8 Hz, 1H), 7.10 (d, *J* = 8.6 Hz, 1H), 5.26 (d, *J* = 11.4 Hz, 1H), 3.74 (s, 3H), 2.76 (qt, *J* = 11.2, 3.2 Hz, 1H), 1.77–1.61 (m, 5H), 1.36–1.14 (m, 3H), 1.13–1.00 (m, 1H), 0.98–0.85 (m, 1H). ^13^C­{H} NMR (101 MHz, CDCl_3_) δ: 168.4, 134.9, 133.8, 131.9, 130.2, 129.6, 124.6, 123.1, 121.6, 113.0, 111.8, 110.7, 51.7, 38.5, 33.1, 31.3, 30.5, 26.3, 25.76, 25.67. IR ν_max_ (cm^–1^): 2926, 2845, 1761, 1697, 1474, 1377, 1326, 1071, 793, 731. HRMS (ESI) *m*/*z*: [M + H]^+^ Calcd for C_24_H_24_N_2_O_2_Br 451.1016; Found 451.1007

#### *N*-((1,3-Dioxoisoindolin-2-yl)­(phenyl)­methyl)­benzamide (**3l**)

To a 22 mL screw top vial were added (1,3-dioxoisoindolin-2-yl)­(phenyl)­methyl acetate (75 mg, 0.254 mmol), Ca­(NTf_2_)_2_ (7.6 mg, 0.013 mmol), *n*Bu_4_NPF_6_ (4.9 mg, 0.013 mmol), and benzamide (37 mg, 0.305 mmol) in 1,2-DCE (1.27 mL), reacting for 1 h and then being isolated by FCC (1:3 EtOAc:Hex) as a white solid (52 mg, 57%). RF (1:1 EtOAc:Hex): 0.71. ^1^H NMR (400 MHz, CDCl_3_) δ 7.93 (d, *J* = 9.6 Hz, 1H), 7.89–7.83 (m, 4H), 7.76–7.70 (m, 2H), 7.62 (d, *J* = 9.6 Hz, 1H), 7.55–7.47 (m, 3H), 7.47–7.42 (m, 2H), 7.41–7.30 (m, 3H). ^13^C­{H} NMR (101 MHz, CDCl_3_) δ: 167.7, 166.3, 137.1, 134.4, 133.3, 132.2, 131.7, 129.0, 128.7, 127.3, 126.1, 123.7, 58.8. IR ν_max_ (cm^–1^): 3369, 3050, 1776, 1710, 1664, 1511, 1312, 1258, 1123, 878, 718. HRMS (ESI) *m*/*z*:: [M + H]^+^ Calcd for C_22_H_17_N_2_O_3_ 357.1234; Found 357.1236.

#### *N*-((3-Chlorophenyl)­(1,3-dioxoisoindolin-2-yl)­methyl)-4-methoxybenzamide (**3m**)

To a 22 mL screw top vial were added (3-chlorophenyl)­(1,3-dioxoisoindolin-2-yl)­methyl acetate (75 mg, 0.227 mmol), Ca­(NTf_2_)_2_ (6.8 mg, 0.011 mmol), *n*Bu_4_NPF_6_ (4.4 mg, 0.011 mmol), and 4-methoxybenzamide (41 mg, 0.272 mmol) in 1,2-DCE (1.14 mL), reacting for 1 h and then being isolated by FCC (1:3 EtOAc:Hex) as a white solid. (34 mg, 36%). RF (1:3 EtOAc:Hex): 0.20. ^1^H NMR (400 MHz, CDCl_3_) δ 7.90–7.86 (m, 2H), 7.86–7.80 (m, 3H), 7.78–7.72 (m, 2H), 7.59 (d, *J* = 9.7 Hz, 1H), 7.49–7.46 (m, 1H), 7.38–7.33 (m, 1H), 7.33–7.29 (m, 2H), 6.97–6.91 (m, 2H), 3.85 (s, 3H). ^13^C­{H} NMR (101 MHz, CDCl_3_) δ: 167.6, 165.8, 162.9, 139.4, 135.0, 134.5, 131.6, 130.2, 129.3, 128.9, 126.4, 125.3, 124.4, 123.9, 114.0, 58.1, 55.5. IR ν_max_ (cm^–1^): 3367, 2922, 1774, 1707, 1604, 1489, 1313, 1246, 1172, 1026, 842, 721. HRMS (ESI) *m*/*z*: [M + H]^+^ Calcd for C_23_H_18_N_2_O_4_Cl 421.0950; Found 421.0952.

#### *N*-((1,3-Dioxoisoindolin-2-yl)­(naphthalen-2-yl)­methyl)­propionamide (**3n**)

To a 22 mL screw top vial were added (1,3-dioxoisoindolin-2-yl)­(naphthalen-2-yl)­methyl acetate (75 mg, 0.217 mmol), Ca­(NTf_2_)_2_ (6.5 mg, 0.011 mmol), *n*Bu_4_NPF_6_ (4.2 mg, 0.011 mmol), and propanamide (19 mg, 0.260 mmol) in 1,2-DCE (1.09 mL), reacting for 1 h and then being isolated by FCC (1:3 to 1:2 EtOAc:Hex) as a white solid. (31 mg, 40%). RF (1:3 EtOAc:Hex): 0.17. ^1^H NMR (400 MHz, CDCl_3_) δ 7.89–7.77 (m, 6H), 7.76–7.70 (m, 2H), 7.57 (d, *J* = 9.7 Hz, 1H), 7.53 (dd, *J* = 8.6, 1.9 Hz, 1H), 7.50–7.44 (m, 2H), 7.31 (d, *J* = 9.7 Hz, 1H), 2.44–2.28 (m, 2H), 1.20 (t, *J* = 7.6 Hz, 3H). ^13^C­{H} NMR (101 MHz, CDCl_3_) δ: 173.0, 167.6, 134.5, 134.4, 133.2, 133.0, 131.7, 129.0, 128.2, 127.6, 126.6, 126.6, 125.2, 123.7, 58.4, 29.5, 9.4. IR ν_max_ (cm^–1^): 3371, 2924, 1774, 1707, 1500, 1351, 1209, 1097, 715. HRMS (ESI) *m*/*z*: [M + H]^+^ Calcd for C_22_H_19_N_2_O_3_ 359.1390; Found 359.1393

#### Benzyl ((1,3-Dioxoisoindolin-2-yl)­(phenyl)­methyl)­carbamate (**3o**)

To a 22 mL screw top vial were added (1,3-dioxoisoindolin-2-yl)­(phenyl)­methyl acetate (75 mg, 0.254 mmol), Ca­(NTf_2_)_2_ (7.6 mg, 0.013 mmol), *n*Bu_4_NPF_6_ (4.9 mg, 0.013 mmol), and benzyl carbamate (46 mg, 0.304 mmol) in 1,2-DCE (1.27 mL), reacting for 1 h and then being isolated by FCC (1:4 EtOAc:Hex) as a colorless oil. (98 mg, 99%). RF (1:3 EtOAc:Hex): 0.34. ^1^H NMR (400 MHz, CDCl_3_) δ 7.86–7.79 (m, 2H), 7.74–7.67 (m, 2H), 7.46–7.39 (m, 2H), 7.36–7.27 (m, 7H), 7.22–7.12 (m, 1H), 6.61 (d, *J* = 7.5 Hz, 1H), 5.14 (dd, *J* = 39.6, 12.2 Hz, 2H). ^13^C­{H} NMR (101 MHz, CDCl_3_) δ: 167.4, 155.3, 136.9, 135.9, 134.4, 131.8, 128.9, 128.6, 128.6, 128.3, 126.0, 123.7, 67.5, 60.5. IR ν_max_ (cm^–1^): 3350, 3062, 3032, 2952, 1774, 1703, 1494, 1381, 1351, 1215, 1116, 1038, 883, 717, 693. HRMS (ESI) *m*/*z*: [M + Na]^+^ Calcd for C_23_H_18_N_2_O_4_Na 409.1159; Found 409.1181

#### 2-(1,3-Dioxoisoindolin-2-yl)-2-phenylacetonitrile (**4a**)

To a 22 mL screw top vial were added (1,3-dioxoisoindolin-2-yl)­(phenyl)­methyl acetate (295 mg, 1.00 mmol), Ca­(NTf_2_)_2_ (30 mg, 0.05 mmol), *n*Bu_4_NPF_6_ (19 mg, 0.05 mmol), and TMSCN (250 μL, 2.00 mmol) in 1,2-DCE (5.0 mL), reacting overnight and then being isolated by FCC (1:6 EtOAc:Hex) as a white solid (202 mg, 77%). RF (1:4 EtOAc:Hex): 0.42. ^1^H NMR (400 MHz, CDCl_3_) δ 7.93–7.86 (m, 2H), 7.81–7.74 (m, 2H), 7.66–7.60 (m, 2H), 7.45–7.38 (m, 3H), 6.39 (s, 1H). ^13^C­{H} NMR (101 MHz, CDCl_3_) δ: 165.7, 134.8, 131.7, 131.3, 129.7, 129.2, 127.8, 124.1, 114.7, 43.0. IR ν_max_ (cm^–1^): 2911, 1712, 1377, 1341, 1099, 885, 712. HRMS (ESI) *m*/*z*: [M + K]^+^ Calcd for C_16_H_10_N_2_O_2_K 301.0374; Found 301.0380

#### 2-(1,3-Dioxoisoindolin-2-yl)-2-(4-fluorophenyl)­acetonitrile (**4b**)

To a 22 mL screw top vial were added (1,3-dioxoisoindolin-2-yl)­(4-fluorophenyl)­methyl acetate (75 mg, 0.239 mmol), Ca­(NTf_2_)_2_ (7.2 mg, 0.012 mmol), *n*Bu_4_NPF_6_ (4.6 mg, 0.012 mmol), and TMSCN (60 μL, 0.478 mmol) in 1,2-DCE (1.20 mL), reacting overnight and then being isolated by FCC (1:6 EtOAc:Hex) as a white solid (56 mg, 83%). RF (1:4 EtOAc:Hex): 0.44. ^1^H NMR (400 MHz, CDCl_3_) δ 7.92–7.86 (m, 2H), 7.82–7.76 (m, 2H), 7.67–7.60 (m, 2H), 7.14–7.06 (m, 2H), 6.36 (s, 1H). ^13^C­{H} NMR (101 MHz, CDCl_3_) δ 165.6 (s), 163.3 (d, *J* = 250.1 Hz), 134.9 (s), 131.2 (s), 130.1 (d, *J* = 8.7 Hz), 127.8 (d, *J* = 3.4 Hz), 124.1 (s), 116.3 (d, *J* = 22.1 Hz), 114.6 (s), 42.4 (s). ^19^F NMR (376 MHz, CDCl_3_) δ −110.73. IR ν_max_ (cm^–1^): 2912, 1716, 1604, 1507, 1377, 1330, 1220, 1095, 840, 713

HRMS (ESI) *m*/*z*: [M–CN]^+^ Calcd for C_15_H_9_NO_2_F 254.0612; Found 254.0615.

#### 2-(3-Chlorophenyl)-2-(1,3-dioxoisoindolin-2-yl)­acetonitrile (**4c**)

To a 22 mL screw top vial were added (3-chlorophenyl)­(1,3-dioxoisoindolin-2-yl)­methyl acetate (50 mg, 0.152 mmol), Ca­(NTf_2_)_2_ (4.6 mg, 0.0076 mmol), *n*Bu_4_NPF_6_ (2.9 mg, 0.0076 mmol), and TMSCN (38 μL, 0.303 mmol) in 1,2-DCE (0.76 mL), reacting overnight and then being isolated by FCC (1:6 EtOAc:Hex) as a white solid (38 mg, 84%). RF (1:4 EtOAc:Hex): 0.32. ^1^H NMR (400 MHz, CDCl_3_) δ 7.94–7.87 (m, 2H), 7.83–7.77 (m, 2H), 7.61–7.57 (m, 1H), 7.56–7.51 (m, 1H), 7.40–7.35 (m, 2H), 6.36 (s, 1H). ^13^C­{H} NMR (101 MHz, CDCl_3_) δ: 165.5, 135.2, 135.0, 133.5, 131.2, 130.5, 130.1, 127.9, 126.0, 124.2, 114.2, 42.4. IR ν_max_ (cm^–1^): 2933, 1776, 1722, 1470, 1433, 1381, 1325, 1079, 788, 721, 684. HRMS (ESI) *m*/*z*: [M–H]^−^ Calcd for C_16_H_8_N_2_O_2_Cl 295.0280; Found 295.0280

#### 2-(2-Bromophenyl)-2-(1,3-dioxoisoindolin-2-yl)­acetonitrile (**4d**)

To a 22 mL screw top vial were added (2-bromophenyl)­(1,3-dioxoisoindolin-2-yl)­methyl acetate (75 mg, 0.200 mmol), Ca­(NTf_2_)_2_ (6.0 mg, 0.010 mmol), *n*Bu_4_NPF_6_ (3.9 mg, 0.010 mmol), and TMSCN (50 μL, 0.400 mmol) in 1,2-DCE (1.00 mL), reacting overnight and then being isolated by FCC (1:6 EtOAc:Hex) as a white solid (50 mg, 73%). RF (1:4 EtOAc:Hex): 0.47. ^1^H NMR (400 MHz, CDCl_3_) δ 8.15 (dd, *J* = 7.9, 1.6 Hz, 1H), 7.92–7.85 (m, 2H), 7.83–7.74 (m, 2H), 7.58 (dd, *J* = 8.0, 1.2 Hz, 1H), 7.47 (td, *J* = 7.7, 1.2 Hz, 1H), 7.30 (td, *J* = 7.7, 1.6 Hz, 1H), 6.66 (s, 1H). ^13^C­{H} NMR (101 MHz, CDCl_3_) δ: 165.5, 134.9, 133.5, 132.2, 131.5, 131.2, 130.0, 127.8, 124.1, 122.7, 114.8, 43.8. IR ν_max_ (cm^–1^): 2916, 1772, 1718, 1466, 1373, 1326, 1082, 1026, 890, 717. HRMS (ESI) *m*/*z*: [M + Na]^+^ Calcd for C_16_H_9_N_2_O_2_BrNa 362.9740; Found 362.9735.

#### 2-(3-Bromo-2-fluorophenyl)-2-(1,3-dioxoisoindolin-2-yl)­acetonitrile (**4e**)

To a 22 mL screw top vial were added (3-bromo-2-fluorophenyl)­(1,3-dioxoisoindolin-2-yl)­methyl acetate (75 mg, 0.191 mmol), Ca­(NTf_2_)_2_ (5.7 mg, 0.010 mmol), *n*Bu_4_NPF_6_ (3.7 mg, 0.010 mmol), and TMSCN (48 μL, 0.382 mmol) in 1,2-DCE (0.96 mL), reacting overnight and then being isolated by FCC (1:8 EtOAc:Hex) as a white solid (19 mg, 28%). RF (1:4 EtOAc:Hex): 0.43. ^1^H NMR (400 MHz, CDCl_3_) δ 8.02–7.97 (m, 1H), 7.93–7.87 (m, 2H), 7.82–7.76 (m, 2H), 7.63 (ddd, *J* = 8.1, 6.6, 1.5 Hz, 1H), 7.18 (td, *J* = 8.0, 1.0 Hz, 1H), 6.67 (s, 1H). ^13^C­{H} NMR (101 MHz, CDCl_3_) δ 165.2 (s), 156.2 (d, *J* = 251.4 Hz), 135.5 (s), 134.9 (s), 131.2 (s), 130.1 (d, *J* = 1.5 Hz), 125.5 (d, *J* = 4.7 Hz), 124.2 (s), 120.1 (d, *J* = 14.3 Hz), 113.8 (s), 109.7 (d, *J* = 20.4 Hz), 37.8 (d, *J* = 4.5 Hz). ^19^F NMR (376 MHz, CDCl_3_) δ −109.38. IR ν_max_ (cm^–1^): 2920, 1772, 1729, 1466, 1377, 1328, 1080, 894, 792, 743, 719. HRMS (ESI) *m*/*z*: [M–H]^−^ Calcd for C_16_H_7_N_2_O_2_BrF 356.9680; Found 356.9682

#### 2-(1,3-Dioxoisoindolin-2-yl)-2-(p-tolyl)­acetonitrile (**4f**)

To a 22 mL screw top vial were added (1,3-dioxoisoindolin-2-yl)­(p-tolyl)­methyl acetate (50 mg, 0.162 mmol), Ca­(NTf_2_)_2_ (4.9 mg, 0.008 mmol), *n*Bu_4_NPF_6_ (3.1 mg, 0.008 mmol), and TMSCN (41 μL, 0.324 mmol) in 1,2-DCE (0.81 mL), reacting overnight and then being isolated by FCC (1:6 EtOAc:Hex) as a white solid (39 mg, 87%). RF (1:4 EtOAc:Hex): 0.36. ^1^H NMR (400 MHz, CDCl_3_) δ 7.94–7.83 (m, 2H), 7.80–7.73 (m, 2H), 7.51 (d, *J* = 8.1 Hz, 2H), 7.20 (d, *J* = 8.0 Hz, 2H), 6.35 (s, 1H), 2.34 (s, 3H). ^13^C­{H} NMR (101 MHz, CDCl_3_) δ: 165.7, 139.9, 134.8, 131.3, 129.9, 128.8, 127.8, 124.0, 114.9, 42.8, 21.2. IR ν_max_ (cm^–1^): 3069, 2909, 1787, 1716, 1513, 1466, 1369, 1332, 1097, 1082, 939, 885, 820, 713. HRMS (ESI) *m*/*z*: [M + H]^+^ Calcd for C_17_H_13_N_2_O_2_ 277.0972; Found 277.0974.

#### 2-(1,3-Dioxoisoindolin-2-yl)-2-(2-methoxyphenyl)­acetonitrile (**4g**)

To a 22 mL screw top vial were added (1,3-dioxoisoindolin-2-yl)­(2-methoxyphenyl)­methyl acetate (50 mg, 0.154 mmol), Ca­(NTf_2_)_2_ (4.6 mg, 0.008 mmol), *n*Bu_4_NPF_6_ (3.0 mg, 0.008 mmol), and TMSCN (39 μL, 0.308 mmol) in 1,2-DCE (0.77 mL), reacting overnight and then being isolated by FCC (1:6 EtOAc:Hex) as a white solid (35 mg, 78%). RF (1:4 EtOAc:Hex): 0.30. ^1^H NMR (400 MHz, CDCl_3_) δ 7.97–7.90 (m, 1H), 7.91–7.83 (m, 2H), 7.79–7.72 (m, 2H), 7.38 (td, *J* = 8.1, 1.6 Hz, 1H), 7.05 (td, *J* = 7.6, 1.0 Hz, 1H), 6.91–6.84 (m, 1H), 6.71 (s, 1H), 3.80 (s, 3H). ^13^C­{H} NMR (101 MHz, CDCl_3_) δ: 165.6, 156.3, 134.6, 131.4, 131.3, 130.5, 123.9, 120.7, 119.1, 115.3, 110.7, 55.7, 38.7. IR ν_max_ (cm^–1^): 2914, 2845, 1774, 1720, 1492, 1466, 1375, 1332, 1250, 1108, 1025, 754, 715. HRMS (ESI) *m*/*z*: [M + H]^+^ Calcd for C_17_H_13_N_2_O_3_ 293.0921; Found 293.0924

#### 2-(1,3-Dioxoisoindolin-2-yl)-2-(naphthalen-2-yl)­acetonitrile (**4h**)

To a 22 mL screw top vial were added (1,3-dioxoisoindolin-2-yl)­(naphthalen-2-yl)­methyl acetate (50 mg, 0.145 mmol), Ca­(NTf_2_)_2_ (4.3 mg, 0.007 mmol), *n*Bu_4_NPF_6_ (2.8 mg, 0.007 mmol), and TMSCN (36 μL, 0.290 mmol) in 1,2-DCE (0.73 mL), reacting overnight and then being isolated by FCC (1:6 EtOAc:Hex) as a white solid (41 mg, 91%). RF (1:4 EtOAc:Hex): 0.23. ^1^H NMR (400 MHz, CDCl_3_) δ 8.17 (d, *J* = 0.9 Hz, 1H), 7.91–7.78 (m, 5H), 7.77–7.71 (m, 2H), 7.60 (dd, *J* = 8.6, 1.9 Hz, 1H), 7.55–7.48 (m, 2H), 6.55 (s, 1H). ^13^C­{H} NMR (101 MHz, CDCl_3_) δ: 165.7, 134.8, 133.5, 132.9, 131.3, 129.4, 128.9, 128.4, 127.8, 127.7, 127.3, 127.0, 124.4, 124.1, 114.8, 43.2. IR ν_max_ (cm^–1^): 2901, 1787, 1716, 1602, 1466, 1369, 1328, 1097, 1084, 911, 833, 758, 711. HRMS (ESI) *m*/*z*: [M + NH_4_]^+^ Calcd for C_20_H_16_N_3_O_2_ 330.1237; Found 330.1243

#### 2-(1,3-Dioxoisoindolin-2-yl)-2-(furan-2-yl)­acetonitrile (**4i**)

To a 22 mL screw top vial were added (1,3-dioxoisoindolin-2-yl)­(furan-2-yl)­methyl acetate (50 mg, 0.175 mmol), Ca­(NTf_2_)_2_ (5.3 mg, 0.009 mmol), *n*Bu_4_NPF_6_ (3.4 mg, 0.009 mmol), and TMSCN (44 μL, 0.350 mmol) in 1,2-DCE (0.88 mL), reacting overnight and then being isolated by FCC (1:6 EtOAc:Hex) as a white solid (13 mg, 29%). RF (1:4 EtOAc:Hex): 0.28. ^1^H NMR (400 MHz, CDCl_3_) δ 7.95–7.89 (m, 2H), 7.82–7.77 (m, 2H), 7.39 (dd, *J* = 1.8, 0.7 Hz, 1H), 6.78 (dt, *J* = 3.4, 0.8 Hz, 1H), 6.45–6.40 (m, 2H). ^13^C­{H} NMR (101 MHz, CDCl_3_) δ: 165.3, 144.0, 143.6, 134.9, 131.2, 124.2, 113.2, 111.4, 111.2, 36.9. IR ν_max_ (cm^–1^): 2918, 2849, 1771, 1720, 1371, 1321, 1108, 1012, 922, 751, 713. HRMS (ESI) *m*/*z*: [M–CN]^+^ Calcd for C_13_H_8_NO_3_ 226.0499; Found 226.0501

#### 2-(1,3-Dioxoisoindolin-2-yl)-2-(thiophen-2-yl)­acetonitrile (**4j**)

To a 22 mL screw top vial were added (1,3-dioxoisoindolin-2-yl)­(thiophen-2-yl)­methyl acetate (75 mg, 0.249 mmol), Ca­(NTf_2_)_2_ (7.5 mg, 0.012 mmol), *n*Bu_4_NPF_6_ (4.8 mg, 0.012 mmol), and TMSCN (63 μL, 0.500 mmol) in 1,2-DCE (1.25 mL), reacting overnight and then being isolated by FCC (1:6 EtOAc:Hex) as a white solid (53 mg, 79%). RF (1:4 EtOAc:Hex): 0.35. ^1^H NMR (400 MHz, CDCl_3_) δ 7.94–7.88 (m, 2H), 7.82–7.76 (m, 2H), 7.44 (d, *J* = 3.5 Hz, 1H), 7.36 (dd, *J* = 5.1, 1.2 Hz, 1H), 7.01 (dd, *J* = 5.1, 3.6 Hz, 1H), 6.55 (s, 1H). ^13^C­{H} NMR (101 MHz, CDCl_3_) δ: 165.3, 134.9, 133.3, 131.2, 129.5, 128.1, 127.2, 124.2, 114.3, 38.2. IR ν_max_ (cm^–1^): 2911, 1776, 1716, 1466, 1377, 1334, 1105, 933, 879, 713. HRMS (ESI) *m*/*z*: [M–CN]^+^ Calcd for C_13_H_8_NO_2_S 242.0270; Found 242.0274

#### 2-Cyclohexyl-2-(1,3-dioxoisoindolin-2-yl)­acetonitrile (**4k**)

To a 22 mL screw top vial were added cyclohexyl­(1,3-dioxoisoindolin-2-yl)­methyl acetate (23 mg, 0.076 mmol), Ca­(NTf_2_)_2_ (2.3 mg, 0.004 mmol), *n*Bu_4_NPF_6_ (1.5 mg, 0.004 mmol), and TMSCN (19 μL, 0.153 mmol) in 1,2-DCE (0.38 mL), reacting overnight and then being isolated by FCC (1:6 EtOAc:Hex) as a white solid (18 mg, 88%).

RF (1:4 EtOAc:Hex): 0.43. ^1^H NMR (400 MHz, CDCl_3_) δ 7.95–7.88 (m, 2H), 7.84–7.76 (m, 2H), 4.86–4.77 (m, 1H), 2.34 (qt, *J* = 11.3, 3.5 Hz, 1H), 2.24–2.14 (m, 1H), 1.90–1.80 (m, 1H), 1.76–1.64 (m, 2H), 1.62–1.52 (m, 1H), 1.41–1.28 (m, 1H), 1.24–1.11 (m, 3H), 1.02–0.89 (m, 1H). ^13^C­{H} NMR (101 MHz, CDCl_3_) δ: 166.3, 134.8, 131.2, 124.0, 115.5, 45.4, 38.6, 30.1, 29.0, 25.7, 25.1, 25.0. IR ν_max_ (cm^–1^): 2927, 2849, 1772, 1718, 1466, 1453, 1377, 1080, 916, 862, 793, 713. HRMS (ESI) *m*/*z*: [M + NH_4_]^+^ Calcd for C_16_H_20_N_3_O_2_ 286.1550; Found 286.1554

#### 2-Cyclopropyl-2-(1,3-dioxoisoindolin-2-yl)­acetonitrile (**4l**)

To a 22 mL screw top vial were added cyclopropyl­(1,3-dioxoisoindolin-2-yl)­methyl acetate (75 mg, 0.289 mmol), Ca­(NTf_2_)_2_ (8.7 mg, 0.015 mmol), *n*Bu_4_NPF_6_ (5.6 mg, 0.015 mmol), and TMSCN (72 μL, 0.579 mmol) in 1,2-DCE (1.45 mL), reacting overnight and then being isolated by FCC (1:6 EtOAc:Hex) as a white solid (56 mg, 86%). RF (1:4 EtOAc:Hex): 0.38. ^1^H NMR (400 MHz, CDCl_3_) δ 7.95–7.90 (m, 2H), 7.84–7.80 (m, 2H), 4.45 (d, *J* = 9.6 Hz, 1H), 1.91–1.82 (m, 1H), 0.94–0.84 (m, 1H), 0.77–0.61 (m, 2H), 0.55–0.44 (m, 1H). ^13^C­{H} NMR (101 MHz, CDCl_3_) δ: 166.1, 134.8, 131.3, 124.0, 115.3, 44.3, 13.4, 4.8, 4.7. IR ν_max_ (cm^–1^): 3006, 2927, 1770, 1712, 1466, 1381, 1330, 1187, 1095, 1030, 903, 838, 797, 711. HRMS (ESI) *m*/*z*: [M + H]^+^ Calcd for C_13_H_11_N_2_O_2_ 227.0815; Found 227.0814.

#### *tert*-Butyl (1-phenylbut-3-en-1-yl)­carbamate (**5a**)

To a 22 mL screw top vial were added (1,3-dioxoisoindolin-2-yl)­(phenyl)­methyl acetate (100 mg, 0.339 mmol), Ca­(NTf_2_)_2_ (10 mg, 0.017 mmol), *n*Bu_4_NPF_6_ (7 mg, 0.017 mmol), and allyl-TMS (64 μL, 0.406 mmol) in 1,2-DCE (1.7 mL), reacting for 1 h, and then MeNH_2_ (33 wt % in EtOH) (206 μL, 1.695 mmol) in EtOH (3.4 mL) overnight, followed by Boc_2_O (156 μL, 0.678 mmol), Et_3_N (47 μL, 0.339 mmol), and DMAP (cat.) in dry THF (1.7 mL). Following completion of the Boc protection (1 h), purification by FCC (1:12 EtOAc:Hex) afforded the pure compound as a white solid (56 mg, 67% over 3 steps). RF (1:12 EtOAc:Hex):0.50. ^1^H NMR (400 MHz, CDCl_3_) δ 7.37–7.20 (m, 5H), 5.75–5.61 (m, 1H), 5.17–5.03 (m, 2H), 4.89 (s, br, 1H), 4.74 (s, br, 1H), 2.51 (s, br, 2H), 1.41 (s, 9H). ^13^C­{H} NMR (101 MHz, CDCl_3_) δ: 155.2, 142.4, 134.0, 128.5, 127.1, 126.2, 118.2, 79.5, 54.0, 41.3, 28.4. Data in accordance with the literature.^26^

#### *tert*-Butyl (1-(4-methoxyphenyl)­but-3-en-1-yl)­carbamate (**5b**)

To a 22 mL screw top vial were added (1,3-dioxoisoindolin-2-yl)­(4-methoxyphenyl)­methyl acetate (75 mg, 0.231 mmol), Ca­(NTf_2_)_2_ (7 mg, 0.012 mmol), *n*Bu_4_NPF_6_ (4.5 mg, 0.012 mmol), and allyl-TMS (44 μL, 0.277 mmol) in 1,2-DCE (1.2 mL), reacting for 1 h, and then MeNH_2_ (33 wt % in EtOH) (134 μL, 1.014 mmol) in EtOH (2.3 mL) overnight, followed by Boc_2_O (105 μL, 0.461 mmol), Et_3_N (32 μL, 0.231 mmol), and DMAP (cat.) in dry THF (1.2 mL). Following completion of the Boc protection (1 h), purification by FCC (1:12 EtOAc:Hex) afforded the pure compound as a white solid (49 mg, 77% over 3 steps). RF (1:12 EtOAc:Hex):0.17. ^1^H NMR (400 MHz, CDCl_3_) δ 7.18 (d, *J* = 8.5 Hz, 2H), 6.86 (d, *J* = 8.6 Hz, 2H), 5.74–5.61 (m, 1H), 5.14–5.02 (m, 2H), 4.81 (s, br, 1H), 4.67 (s, br, 1H), 3.79 (s, 3H), 2.55–2.45 (m, br, 2H), 1.41 (s, 9H). ^13^C­{H} NMR (101 MHz, CDCl_3_) δ: 158.7, 155.2, 134.6, 134.2, 127.4, 118.0, 113.9, 79.4, 55.3, 53.6, 41.2, 28.4. Data in accordance with the literature.^26^

#### *tert*-Butyl (1-(p-tolyl)­but-3-en-1-yl)­carbamate (**5c**)

To a 22 mL screw top vial were added (1,3-dioxoisoindolin-2-yl)­(p-tolyl)­methyl acetate (75 mg, 0.242 mmol), Ca­(NTf_2_)_2_ (7 mg, 0.012 mmol), *n*Bu_4_NPF_6_ (4.7 mg, 0.012 mmol), and allyl-TMS (46 μL, 0.290 mmol) in 1,2-DCE (1.2 mL), reacting for 1 h, and then MeNH_2_ (33 wt % in EtOH) (140 μL, 1.21 mmol) in EtOH (2.3 mL) overnight, followed by Boc_2_O (111 μL, 0.484 mmol), Et_3_N (34 μL, 0.242 mmol), and DMAP (cat.) in dry THF (1.2 mL). Following completion of the Boc protection (1 h), purification by FCC (1:19 EtOAc:Hex) afforded the pure compound as a white solid (35 mg, 55% over 3 steps). RF (1:12 EtOAc:Hex):0.29. ^1^H NMR (400 MHz, CDCl_3_) δ 7.20–7.07 (m, 4H), 5.74–5.61 (m, 1H), 5.15–5.02 (m, 2H), 4.84 (s, br, 1H), 4.70 (s, br, 1H), 2.56–2.44 (m, br, 2H), 2.32 (s, 3H), 1.41 (s, 9H). ^13^C­{H} NMR (101 MHz, CDCl_3_) δ: 155.2, 139.4, 136.7, 134.1, 129.2, 126.2, 118.0, 79.4, 53.8, 41.2, 28.4, 21.1. Data in accordance with the literature.^26^

#### *tert*-Butyl (1-(2-methoxyphenyl)­but-3-en-1-yl)­carbamate (**5d**)

To a 22 mL screw top vial were added (1,3-dioxoisoindolin-2-yl)­(2-methoxyphenyl)­methyl acetate (40 mg, 0.123 mmol), Ca­(NTf_2_)_2_ (3.7 mg, 0.006 mmol), *n*Bu_4_NPF_6_ (2.4 mg, 0.006 mmol), and allyl-TMS (23 μL, 0.148 mmol) in 1,2-DCE (0.62 mL), reacting for 1 h, and then MeNH_2_ (33 wt % in EtOH) (71 μL, 0.615 mmol) in EtOH (1.23 mL) overnight, followed by Boc_2_O (57 μL, 0.246 mmol), Et_3_N (18 μL, 0.123 mmol), and DMAP (cat.) in dry THF (0.62 mL). Following completion of the Boc protection (1 h), purification by FCC (1:15 EtOAc:Hex) afforded the pure compound as a white solid (24 mg, 70% over 3 steps). RF (1:12 EtOAc:Hex):0.37. ^1^H NMR (400 MHz, CDCl_3_) δ 7.25–7.19 (m, 1H), 7.17–7.13 (m, 1H), 6.94–6.84 (m, 2H), 5.75–5.61 (m, 1H), 5.34 (d, *J* = 6.6 Hz, 1H), 5.11–4.97 (m, 2H), 4.97–4.85 (m, 1H), 3.85 (s, 3H), 2.58–2.43 (m, 2H), 1.42 (s, 9H). ^13^C­{H} NMR (101 MHz, CDCl_3_) δ: 156.9, 155.2, 135.0, 130.0, 128.2, 128.1, 120.5, 117.2, 110.8, 79.1, 55.3, 51.9, 39.9, 28.4. Data in accordance with the literature.^26^

#### *tert*-Butyl (1-(4-fluorophenyl)­but-3-en-1-yl)­carbamate (**5e**)

To a 22 mL screw top vial were added (1,3-dioxoisoindolin-2-yl)­(4-fluorophenyl)­methyl acetate (75 mg, 0.229 mmol), Ca­(NTf_2_)_2_ (7 mg, 0.012 mmol), *n*Bu_4_NPF_6_ (4.6 mg, 0.012 mmol), and allyl-TMS (46 μL, 0.290 mmol) in 1,2-DCE (1.2 mL), reacting for 2 h, and then MeNH_2_ (33 wt % in EtOH) (134 μL, 1.21 mmol) in EtOH (2.3 mL) overnight, followed by Boc_2_O (110 μL, 0.484 mmol), Et_3_N (33 μL, 0.242 mmol), and DMAP (cat.) in dry THF (1.2 mL). Following completion of the Boc protection (1 h), purification by FCC (1:19 EtOAc:Hex) afforded the pure compound as a white solid (18 mg, 26% over 3 steps). RF (1:12 EtOAc:Hex):0.28. ^1^H NMR (400 MHz, CDCl_3_) δ 7.23 (dd, *J* = 8.2, 5.5 Hz, 2H), 7.01 (t, *J* = 8.6 Hz, 2H), 5.73–5.59 (m, 1H), 5.16–5.05 (m, 2H), 4.84 (s, br, 1H), 4.70 (s, br, 1H), 2.48 (m, br, 2H), 1.35 (s, 9H). ^13^C­{H} NMR (101 MHz, CDCl_3_) δ 161.9 (d, *J* = 244.9 Hz), 155.1 (s), 138.2 (s), 133.7 (s), 127.8 (d, *J* = 8.0 Hz), 118.5 (s), 115.3 (d, *J* = 21.4 Hz), 79.7 (s), 53.43 (s), 41.2 (s), 28.3 (s). ^19^F NMR (376 MHz, CDCl_3_) δ −115.84. Data in accordance with the literature.^26^

#### *tert*-Butyl (1-(3-chlorophenyl)­but-3-en-1-yl)­carbamate (**5f**)

To a 22 mL screw top vial were added (3-chlorophenyl)­(1,3-dioxoisoindolin-2-yl)­methyl acetate (75 mg, 0.227 mmol), Ca­(NTf_2_)_2_ (6.6 mg, 0.011 mmol), *n*Bu_4_NPF_6_ (4.3 mg, 0.011 mmol), and allyl-TMS (43 μL, 0.272 mmol) in 1,2-DCE (1.1 mL), reacting for 5 h, and then MeNH_2_ (33 wt % in EtOH) (132 μL, 1.14 mmol) in EtOH (2.3 mL) overnight, followed by Boc_2_O (104 μL, 0.454 mmol), Et_3_N (32 μL, 0.227 mmol), and DMAP (cat.) in dry THF (1.1 mL). Following completion of the Boc protection (1 h), purification by FCC (1:19 EtOAc:Hex) afforded the pure compound as a white solid (21 mg, 30% over 3 steps). RF (1:12 EtOAc:Hex):0.31. ^1^H NMR (400 MHz, CDCl_3_) δ 7.29–7.19 (m, 3H), 7.17–7.12 (m, 1H), 5.73–5.58 (m, 1H), 5.20–5.06 (m, 2H), 4.87 (s, br, 1H), 4.71 (s, br, 1H), 2.56–2.40 (m, br, 2H), 1.41 (s, 9H). ^13^C­{H} NMR (101 MHz, CDCl_3_) δ: 155.1, 144.7, 134.4, 133.4, 129.8, 127.3, 126.4, 124.5, 118.7, 79.8, 53.6, 41.1, 28.3. Data in accordance with the literature.^27^

#### *tert*-Butyl (1-(2-bromophenyl)­but-3-en-1-yl)­carbamate (**5g**)

To a 22 mL screw top vial were added (2-bromophenyl)­(1,3-dioxoisoindolin-2-yl)­methyl acetate (75 mg, 0.200 mmol), Ca­(NTf_2_)_2_ (6.0 mg, 0.010 mmol), *n*Bu_4_NPF_6_ (3.9 mg, 0.010 mmol), and allyl-TMS (38 μL, 0.240 mmol) in 1,2-DCE (1.0 mL), reacting for 5 h, and then MeNH_2_ (33 wt % in EtOH) (116 μL, 1.00 mmol) in EtOH (2.0 mL) overnight, followed by Boc_2_O (92 μL, 0.400 mmol), Et_3_N (29 μL, 0.200 mmol), and DMAP (cat.) in dry THF (1.0 mL). Following completion of the Boc protection (1 h), purification by FCC (1:19 EtOAc:Hex) afforded the pure compound as a white solid (22 mg, 31% over 3 steps). RF (1:12 EtOAc:Hex):0.28. ^1^H NMR (400 MHz, CDCl_3_) δ 7.53 (d, *J* = 7.9 Hz, 1H), 7.36–7.21 (m, 2H), 7.16–7.04 (m, 1H), 5.79–5.62 (m, 1H), 5.22–4.79 (m, 4H), 2.56 (s, 1H), 2.50–2.27 (m, 1H), 1.41 (s, 9H). ^13^C­{H} NMR (101 MHz, CDCl_3_) δ: 155.0, 141.5, 133.6, 133.2, 128.5, 127.5, 127.2, 122.7, 118.6, 79.7, 53.5, 39.5, 28.3. Data in accordance with the literature.^28^

#### *tert*-Butyl ((5-bromo-1-methyl-1*H*-indol-3-yl)­(phenyl)­methyl)­carbamate (**5h**)

To a 22 mL screw top vial were added (1,3-dioxoisoindolin-2-yl)­(phenyl)­methyl acetate (75 mg, 0.254 mmol), Ca­(NTf_2_)_2_ (7.6 mg, 0.013 mmol), *n*Bu_4_NPF_6_ (4.9 mg, 0.013 mmol), and 5-bromo-*N*-methylindole (64 mg, 0.305 mmol) in 1,2-DCE (1.27 mL), reacting for 15 min, and then MeNH_2_ (33 wt % in EtOH) (148 μL, 1.27 mmol) in EtOH (2.54 mL) overnight, followed by Boc_2_O (117 μL, 0.508 mmol), Et_3_N (35 μL, 0.254 mmol), and DMAP (cat.) in dry THF (1.27 mL). Following completion of the Boc protection (1 h), purification by FCC (1:5 EtOAc:Hex) afforded the pure compound as a white solid (41 mg, 39% over 3 steps). RF (1:4 EtOAc:Hex):0.33. ^1^H NMR (400 MHz, CDCl_3_) δ 7.73–7.60 (m, 1H), 7.39–7.32 (m, 4H), 7.31–7.26 (m, 2H), 7.16–7.10 (m, 1H), 6.57 (s, 1H), 6.14 (s, 1H), 5.15 (s, 1H), 3.64 (s, 3H), 1.46 (s, 9H). ^13^C­{H} NMR (101 MHz, CDCl_3_) δ: 155.2, 141.8, 136.2, 129.0, 128.5, 128.1, 127.3, 126.8, 124.9, 122.2, 116.3, 112.9, 110.9, 79.8, 51.5, 32.9, 28.4. IR ν_max_ (cm^–1^): 3408, 2976, 2931, 1710, 1495, 1474, 1366, 1237, 1157, 1043, 1012, 881, 799, 754, 705. HRMS (ESI) *m*/*z*: [M–BocNH]^+^ Calcd for C_16_H_13_N^81^Br 300.0205, Found 300.0211

#### *tert*-Butyl ((5-bromo-1-methyl-1*H*-indol-3-yl)­(4-methoxyphenyl)­methyl)­carbamate (**5i**)

To a 22 mL screw top vial were added (1,3-dioxoisoindolin-2-yl)­(4-methoxyphenyl)­methyl acetate (75 mg, 0.231 mmol), Ca­(NTf_2_)_2_ (6.9 mg, 0.012 mmol), *n*Bu_4_NPF_6_ (4.5 mg, 0.012 mmol), and 5-bromo-*N*-methylindole (58 mg, 0.277 mmol) in 1,2-DCE (1.20 mL), reacting for 15 min, and then MeNH_2_ (33 wt % in EtOH) (135 μL, 1.16 mmol) in EtOH (2.31 mL) overnight, followed by Boc_2_O (106 μL, 0.461 mmol), Et_3_N (32 μL, 0.231 mmol), and DMAP (cat.) in dry THF (1.20 mL). Following completion of the Boc protection (1 h), purification by FCC (1:8 to 1:6 EtOAc:Hex) afforded the pure compound as an off-white solid (22 mg, 21% over 3 steps). RF (1:4 EtOAc:Hex):0.28. ^1^H NMR (400 MHz, CDCl_3_) δ 7.62 (s, 1H), 7.33–7.24 (m, 3H), 7.14 (d, *J* = 8.7 Hz, 1H), 6.91–6.85 (m, 2H), 6.61 (s, 1H), 6.08 (s, 1H), 5.11 (s, 1H), 3.81 (s, 3H), 3.67 (s, 3H), 1.46 (s, 9H). ^13^C­{H} NMR (101 MHz, CDCl_3_) δ: 158.8, 155.1, 136.2, 128.8, 128.0, 128.0, 124.9, 122.2, 116.5, 113.9, 112.8, 110.9, 79.7, 55.3, 51.0, 32.9, 28.4. IR ν_max_ (cm^–1^): 3330, 2978, 2953, 2924, 1675, 1608, 1511, 1474, 1366, 1159, 1023, 790. HRMS (ESI) *m*/*z*: [M + Na]+ Calcd for C_22_H_25_N_2_O_3_Na^81^Br 469.0920; Found 469.0927

#### (9*H*-Fluoren-9-yl)­methyl (1-phenylbut-3-en-1-yl)­carbamate (**6a**)

To a 22 mL screw top vial were added (1,3-dioxoisoindolin-2-yl)­(phenyl)­methyl acetate (75 mg, 0.254 mmol), Ca­(NTf_2_)_2_ (7.6 mg, 0.013 mmol), *n*Bu_4_NPF_6_ (4.9 mg, 0.013 mmol), and allyl-TMS (48 μL, 0.305 mmol) in 1,2-DCE (1.27 mL), reacting for 1 h, and then MeNH_2_ (33 wt % in EtOH) (148 μL, 1.27 mmol) in EtOH (2.54 mL) overnight, followed by FmocCl (79 mg, 0.305 mmol) and Et_3_N (35 μL, 0.254 mmol) in dry DCM (1.27 mL). Following completion of the Fmoc protection (1 h), purification by FCC (1:19 to 1:6 EtOAc:Hex) afforded the pure compound as a white solid (59 mg, 63% over 3 steps). RF (1:4 EtOAc:Hex):0.83. ^1^H NMR (400 MHz, CDCl_3_) δ 7.78–7.68 (m, 2H), 7.63–7.10 (m, 11H), 5.76–5.55 (m, 1H), 5.21–5.00 (m, 3H), 4.87–4.58 (m, 1H), 4.49–4.30 (m, 2H), 4.26–4.05 (m, 1H), 2.65–2.34 (m, 2H). ^13^C­{H} NMR (101 MHz, CDCl_3_) δ: 155.7, 144.0, 141.9, 141.3, 133.9, 128.7, 127.7, 127.4, 127.1, 127.1, 126.3, 125.1, 120.0, 118.4, 66.6, 54.5, 47.3, 41.0. Data in accordance with the literature.^29^

#### *N*-(1-Phenylbut-3-en-1-yl)­benzamide (**6b**)

To a 22 mL screw top vial were added (1,3-dioxoisoindolin-2-yl)­(phenyl)­methyl acetate (75 mg, 0.254 mmol), Ca­(NTf_2_)_2_ (7.6 mg, 0.013 mmol), *n*Bu_4_NPF_6_ (4.9 mg, 0.013 mmol), and allyl-TMS (48 μL, 0.305 mmol) in 1,2-DCE (1.27 mL), reacting for 1 h, and then MeNH_2_ (33 wt % in EtOH) (148 μL, 1.27 mmol) in EtOH (2.54 mL) overnight, followed by BzCl (35 μL, 0.305 mmol) and Et_3_N (35 μL, 0.254 mmol) in dry DCM (1.27 mL). Following completion of the Bz protection (1 h), purification by FCC (1:6 EtOAc:Hex) afforded the pure compound as a white solid (31 mg, 49% over 3 steps). RF (1:4 EtOAc:Hex):0.31. ^1^H NMR (400 MHz, CDCl_3_) δ 7.81–7.72 (m, 2H), 7.52–7.45 (m, 1H), 7.45–7.38 (m, 2H), 7.34 (d, *J* = 4.4 Hz, 4H), 7.29–7.23 (m, 1H), 6.52 (d, *J* = 7.2 Hz, 1H), 5.76 (ddt, *J* = 17.1, 10.1, 7.0 Hz, 1H), 5.29 (dd, *J* = 14.4, 6.9 Hz, 1H), 5.22–5.07 (m, 2H), 2.69 (t, *J* = 6.8 Hz, 2H). ^13^C­{H} NMR (101 MHz, CDCl_3_) δ: 166.8, 141.6, 134.6, 134.1, 131.5, 128.7, 128.6, 127.4, 127.0, 126.5, 118.5, 52.8, 40.6. Data in accordance with the literature.^30^

#### Allyl (1-Phenylbut-3-en-1-yl)­carbamate (**6c**)

To a 22 mL screw top vial were added (1,3-dioxoisoindolin-2-yl)­(phenyl)­methyl acetate (75 mg, 0.254 mmol), Ca­(NTf_2_)_2_ (7.6 mg, 0.013 mmol), *n*Bu_4_NPF_6_ (4.9 mg, 0.013 mmol), and allyl-TMS (48 μL, 0.305 mmol) in 1,2-DCE (1.27 mL), reacting for 1 h, and then MeNH_2_ (33 wt % in EtOH) (148 μL, 1.27 mmol) in EtOH (2.54 mL) overnight, followed by AllocCl (31 μL, 0.305 mmol) and Et_3_N (35 μL, 0.254 mmol) in dry DCM (1.27 mL). Following completion of the Alloc protection (1 h), purification by FCC (1:9 EtOAc:Hex) afforded the pure compound as a viscous colorless oil. (34 mg, 58% over 3 steps). RF (1:8 EtOAc:Hex):0.22. ^1^H NMR (400 MHz, CDCl_3_) δ 7.39–7.22 (m, 5H), 5.99–5.80 (m, 1H), 5.68 (ddt, *J* = 17.2, 10.1, 7.0 Hz, 1H), 5.37–5.01 (m, 5H), 4.89–4.72 (m, 1H), 4.64–4.45 (m, 2H), 2.55 (t, *J* = 6.1 Hz, 2H). ^13^C­{H} NMR (101 MHz, CDCl_3_) δ: 155.5, 142.0, 133.8, 132.8, 128.6, 127.3, 126.2, 118.4, 117.8, 65.6, 54.4, 41.1. Data in accordance with the literature.^31^

#### *N*-(1-(Thiophen-2-yl)­but-3-en-1-yl)­acetamide (**6d**)

To a 22 mL screw top vial were added (1,3-dioxoisoindolin-2-yl)­(thiophen-2-yl)­methyl acetate (75 mg, 0.249 mmol), Ca­(NTf_2_)_2_ (7.4 mg, 0.012 mmol), *n*Bu_4_NPF_6_ (4.8 mg, 0.012 mmol), and allyl-TMS (47 μL, 0.299 mmol) in 1,2-DCE (1.25 mL), reacting for 1 h, and then MeNH_2_ (33 wt % in EtOH) (145 μL, 1.27 mmol) in EtOH (2.49 mL) overnight, followed by AcCl (21 μL, 0.299 mmol) and Et_3_N (35 μL, 0.249 mmol) in dry DCM (1.25 mL). Following completion of the Ac protection (1 h), purification by FCC (1:4 to 1:2 EtOAc:Hex) afforded the pure compound as a white solid (43 mg, 88% over 3 steps). RF (1:3 EtOAc:Hex):0.19. ^1^H NMR (400 MHz, CDCl_3_) δ 7.19 (dd, *J* = 4.2, 2.1 Hz, 1H), 6.98–6.90 (m, 2H), 6.10 (d, *J* = 7.4 Hz, 1H), 5.75 (ddt, *J* = 17.1, 10.2, 7.0 Hz, 1H), 5.37 (dd, *J* = 15.3, 6.8 Hz, 1H), 5.19–5.06 (m, 2H), 2.69–2.57 (m, 2H), 1.97 (s, 3H). ^13^C­{H} NMR (101 MHz, CDCl_3_) δ: 169.3, 145.5, 133.6, 126.8, 124.4, 124.2, 118.5, 48.3, 40.7, 23.24. IR ν_max_ (cm^–1^): 3240, 3065, 2953, 2849, 1634, 1554, 1433, 1366, 1291, 1077, 1034, 926, 834, 708. HRMS (ESI) *m*/*z*: [M + H]^+^ Calcd for C_10_H_13_NOS 196.0791; Found 196.0784

#### Benzyl (1-(2-Methoxyphenyl)­but-3-en-1-yl)­carbamate (**6e**)

To a 22 mL screw top vial were added (1,3-dioxoisoindolin-2-yl)­(2-methoxyphenyl)­methyl acetate (75 mg, 0.231 mmol), Ca­(NTf_2_)_2_ (7.2 mg, 0.012 mmol), *n*Bu_4_NPF_6_ (4.6 mg, 0.012 mmol), and allyl-TMS (44 μL, 0.277 mmol) in 1,2-DCE (1.15 mL), reacting for 1 h, and then MeNH_2_ (33 wt % in EtOH) (135 μL, 1.16 mmol) in EtOH (2.31 mL) overnight, followed by CbzCl (40 μL, 0.277 mmol) and Et_3_N (34 μL, 0.231 mmol) in dry DCM (1.15 mL). Following completion of the Cbz protection (1 h), purification by FCC (1:12 EtOAc:Hex) afforded the pure compound as a white solid (31 mg, 43% over 3 steps). RF (1:4 EtOAc:Hex):0.56. ^1^H NMR (400 MHz, CDCl_3_) δ 7.45–7.11 (m, 7H), 6.98–6.82 (m, 2H), 5.79–5.54 (m, 2H), 5.19–4.90 (m, 5H), 3.84 (s, 3H), 2.64–2.47 (m, 2H). ^13^C­{H} NMR (101 MHz, CDCl_3_) δ: 156.9, 155.7, 136.7, 134.8, 129.4, 128.5, 128.5, 128.4, 128.2, 128.1, 120.6, 117.5, 110.9, 66.7, 55.3, 52.9, 39.8. Data in accordance with the literature.^32^

#### Benzyl ((5-Bromo-1-methyl-1H-indol-3-yl)­(phenyl)­methyl)­carbamate (**6f**)

To a 22 mL screw top vial were added (1,3-dioxoisoindolin-2-yl)­(phenyl)­methyl acetate (75 mg, 0.254 mmol), Ca­(NTf_2_)_2_ (7.6 mg, 0.013 mmol), *n*Bu_4_NPF_6_ (4.9 mg, 0.013 mmol), and 5-bromo-*N*-methylindole (64 mg, 0.305 mmol) in 1,2-DCE (1.27 mL), reacting for 1 h, and then MeNH_2_ (33 wt % in EtOH) (148 μL, 1.27 mmol) in EtOH (2.54 mL) overnight, followed by CbzCl (79 mg, 0.305 mmol) and Et_3_N (35 μL, 0.254 mmol) in dry DCM (1.27 mL). Following completion of the Cbz protection (1 h), purification by FCC (1:12 to 1:4 EtOAc:Hex) afforded the pure compound as a viscous pale-yellow oil (36 mg, 32% over 3 steps). RF (1:4 EtOAc:Hex):0.27. ^1^H NMR (400 MHz, CDCl_3_) δ 7.59 (s, 1H), 7.42–7.27 (m, 11H), 7.17–7.10 (m, 1H), 6.66–6.55 (m, 1H), 6.19 (d, *J* = 7.3 Hz, 1H), 5.38 (t, *J* = 15.8 Hz, 1H), 5.13 (q, *J* = 12.3 Hz, 2H), 3.68–3.61 (m, 3H). ^13^C­{H} NMR (101 MHz, CDCl_3_) δ: 155.6, 141.3, 136.5, 136.2, 129.1, 128.6, 128.6, 128.1, 127.9, 127.5, 126.9, 126.4, 125.0, 122.1, 115.6, 113.0, 111.0, 66.9, 52.1, 33.0. IR ν_max_ (cm^–1^): 3403, 3311,3028, 2920, 1686, 1492, 1474, 1213, 1025, 695. HRMS (ESI) *m*/*z*: [M + K]^+^ Calcd for C_24_H_21_N_2_O_2_BrK 487.0418; Found 487.0411

#### *N*-((5-Bromo-1-methyl-1H-indol-3-yl)­(phenyl)­methyl)­acetamide (**6g**)

To a 22 mL screw top vial were added (1,3-dioxoisoindolin-2-yl)­(phenyl)­methyl acetate (75 mg, 0.254 mmol), Ca­(NTf_2_)_2_ (7.6 mg, 0.013 mmol), *n*Bu_4_NPF_6_ (4.9 mg, 0.013 mmol), and 5-bromo-*N*-methylindole (64 mg, 0.305 mmol) in 1,2-DCE (1.27 mL), reacting for 1 h, and then MeNH_2_ (33 wt % in EtOH) (148 μL, 1.27 mmol) in EtOH (2.54 mL) overnight, followed by AcCl (22 μL, 0.305 mmol) and Et_3_N (35 μL, 0.254 mmol) in dry DCM (1.27 mL). Following completion of the Ac protection (1 h), purification by FCC (1:2 to 1:1 EtOAc:Hex) afforded the pure compound as a white solid (37 mg, 41% over 3 steps). RF (1:1 EtOAc:Hex):0.26. ^1^H NMR (400 MHz, DMSO) δ 8.71 (d, *J* = 8.7 Hz, 1H), 7.50 (d, *J* = 1.8 Hz, 1H), 7.43–7.32 (m, 5H), 7.30–7.24 (m, 2H), 6.99 (s, 1H), 6.31 (d, *J* = 8.6 Hz, 1H), 3.72 (s, 3H), 1.91 (s, 3H). ^13^C­{H} NMR (101 MHz, DMSO) δ: 168.8, 142.8, 136.1, 129.9, 128.7, 128.2, 127.47, 127.32, 124.27, 121.8, 116.1, 112.5, 112.0, 49.1, 33.0, 23.0. Data in accordance with the literature.^33^

#### *N*-((5-Bromo-1-methyl-1H-indol-3-yl)­(phenyl)­methyl)­benzamide (**6h**)

To a 22 mL screw top vial were added (1,3-dioxoisoindolin-2-yl)­(phenyl)­methyl acetate (75 mg, 0.254 mmol), Ca­(NTf_2_)_2_ (7.6 mg, 0.013 mmol), *n*Bu_4_NPF_6_ (4.9 mg, 0.013 mmol), and 5-bromo-*N*-methylindole (64 mg, 0.305 mmol) in 1,2-DCE (1.27 mL), reacting for 1 h, and then MeNH_2_ (33 wt % in EtOH) (148 μL, 1.27 mmol) in EtOH (2.54 mL) overnight, followed by BzCl (35 μL, 0.305 mmol) and Et_3_N (35 μL, 0.254 mmol) in dry DCM (1.27 mL). Following completion of the Bz protection (1 h), purification by FCC (1:4 to 1:2 EtOAc:Hex) afforded the pure compound as a white solid (73 mg, 69% over 3 steps). RF (1:4 EtOAc:Hex):0.15. ^1^H NMR (400 MHz, DMSO) δ 9.24 (d, *J* = 8.6 Hz, 1H), 7.98–7.88 (m, 2H), 7.59–7.54 (m, 1H), 7.54–7.34 (m, 8H), 7.33–7.24 (m, 2H), 6.99 (s, 1H), 6.62 (d, *J* = 8.6 Hz, 1H), 3.73 (s, 3H). ^13^C­{H} NMR (101 MHz, DMSO) δ: 166.2, 142.6, 136.1, 134.9, 131.7, 130.3, 128.8, 128.7, 128.5, 128.0, 127.8, 127.5, 124.3, 121.7, 115.8, 112.5, 112.1, 49.8, 33.0. Data in accordance with the literature.^33^

#### Allyl ((5-Bromo-1-methyl-1H-indol-3-yl)­(phenyl)­methyl)­carbamate (**6i**)

To a 22 mL screw top vial were added (1,3-dioxoisoindolin-2-yl)­(phenyl)­methyl acetate (75 mg, 0.254 mmol), Ca­(NTf_2_)_2_ (7.6 mg, 0.013 mmol), *n*Bu_4_NPF_6_ (4.9 mg, 0.013 mmol), and 5-bromo-*N*-methylindole (64 mg, 0.305 mmol) in 1,2-DCE (1.27 mL), reacting for 1 h, and then MeNH_2_ (33 wt % in EtOH) (148 μL, 1.27 mmol) in EtOH (2.54 mL) overnight, followed by AllocCl (35 μL, 0.305 mmol) and Et_3_N (35 μL, 0.254 mmol) in dry DCM (1.27 mL). Following completion of the Alloc protection (1 h), purification by FCC (1:8 to 1:4 EtOAc:Hex) afforded the pure compound as a viscous pale-yellow oil (36 mg, 35% over 3 steps). RF (1:4 EtOAc:Hex):0.35. ^1^H NMR (400 MHz, CDCl_3_) δ 7.64–7.57 (m, 1H), 7.39–7.28 (m, 6H), 7.16–7.12 (m, 1H), 6.66–6.57 (m, 1H), 6.17 (d, *J* = 7.7 Hz, 1H), 5.98–5.85 (m, 1H), 5.43–5.08 (m, 3H), 4.67–4.53 (m, 2H), 3.66 (s, 3H). ^13^C­{H} NMR (101 MHz, CDCl_3_) δ: 155.5, 141.4, 136.2, 132.8, 129.1, 128.6, 127.9, 127.5, 126.8, 125.0, 122.1, 117.8, 115.6, 113.0, 111.0, 65.8, 52.1, 32.9. IR ν_max_ (cm^–1^): 3291, 3060, 2924, 1679, 1533, 1474, 1246, 1034, 767, 701. HRMS (ESI) *m*/*z*: [M–AllocNH]^+^ Calcd for C_16_H_13_N^81^Br 300.0205; Found 300.0214

## Supplementary Material



## Data Availability

The data underlying this study are available in the published article and its Supporting Information

## Notes

General information, experimental details, and NMR spectra and data (PDF)

## References

[1] Trowbridge A., Walton S. M., Gaunt M. J. (2020). New Strategies
for the Transition-Metal
Catalyzed Synthesis of Aliphatic Amines. Chem.
Rev..

[2] Afanasyev O. I., Kuchuk E. A., Muratov K. M., Denisov G. L., Chusov D. (2021). Symmetrical
Tertiary Amines: Applications and Synthetic Approaches. Eur. J. Org. Chem..

[3] Mitchell E. A., Peschiulli A., Lefevre N., Meerpoel L., Maes B. U. W. (2012). Direct
α-Functionalization of Saturated Cyclic Amines. Chem. - Eur. J..

[4] Vasu D., Fuentes de Arriba A. L., Leitch J. A., de Gombert A., Dixon D. J. (2019). Primary α-tertiary amine synthesis via α-C–H
functionalization. Chem. Sci..

[5] Deng T., Han X.-L., Yu Y., Cheng C., Liu X., Gao Y., Wu K., Li Z., Luo J., Deng L. (2024). Organocatalytic
asymmetric α-C–H functionalization of alkyl amines. Nat. Catal..

[6] Jain P., Verma P., Xia G., Yu J.-Q. (2017). Enantioselective
amine α-functionalization via palladium-catalysed C–H
arylation of thioamides. Nat. Chem..

[7] Leng L., Fu Y., Liu P., Ready J. M. (2020). Regioselective, Photocatalytic α-Functionalization
of Amines. J. Am. Chem. Soc..

[8] Ye J., Kalvet I., Schoenebeck F., Rovis T. (2018). Direct α-alkylation
of primary aliphatic amines enabled by CO2 and electrostatics. Nat. Chem..

[9] Ryder A. S. H., Cunningham W. B., Ballantyne G., Mules T., Kinsella A. G., Turner-Dore J., Turner-Dore J., Alder C. M., Edwards L. J., McKay B. S. J., Grayson M. N. (2020). Photocatalytic α-Tertiary Amine Synthesis via
C–H Alkylation of Unmasked Primary Amines. Angew. Chem., Int. Ed..

[10] Fu Z., Fu Y., Yin J., Hao G., Yi X., Zhong T., Guo S., Cai H. (2021). Electrochemical
strategies for N-cyanation of secondary
amines and α C-cyanation of tertiary amines under transition
metal-free conditions. Green. Chem..

[11] Liu Y., Li X., Li J. (2024). Photocatalytic
and electrocatalytic α-C–H
functionalization of tertiary amines. Tetrahedron.

[12] Brown D. G., Boström J. (2016). Analysis of
Past and Present Synthetic Methodologies
on Medicinal Chemistry: Where Have All the New Reactions Gone?. J. Med. Chem..

[13] Basson A. J., Cameron M. P., McLaughlin M. G. (2025). Calcium
catalysed Strecker-type reactions
towards α-aminonitriles. Chem. Commun..

[14] Basson A.
J., Halcovitch N. R., McLaughlin M. G. (2022). Unified Approach to Diverse Fused
Fragments via Catalytic Dehydrative Cyclization. Chem. - Eur. J..

[15] Basson A. J., McLaughlin M. G. (2023). Access to functionalized Mannich scaffolds via a calcium-catalyzed
dehydrative aza-Friedel-Crafts reaction. Cell
Rep. Phys. Sci..

[16] Abdallahi S. M., Ewies E. F., El-Shazly M., Ould Elemine B., Hadou A., Moncol J., Lawson A. M., Daich A., Othman M. (2021). Autotandem Catalysis: Inexpensive
and Green Access
to Functionalized Ketones by Intermolecular Iron-Catalyzed Amidoalkynylation/Hydration
Cascade Reaction via N-Acyliminium Ion Chemistry. Chem. - Eur. J..

[17] Vieira A. S., Ferreira F. P., Fiorante P. F., Guadagnin R. C., Stefani H. A. (2008). Nucleophilic addition of potassium organotrifluoroborates
to chiral cyclic N-acyliminium ions: stereoselective synthesis of
functionalized N-heterocycles. Tetrahedron.

[18] Jury J. C., Swamy N. K., Yazici A., Willis A. C., Pyne S. G. (2009). Metal-Catalyzed
Cycloisomerization Reactions of cis-4-Hydroxy-5-alkynylpyrrolidinones
and cis-5-Hydroxy-6-alkynylpiperidinones: Synthesis of Furo­[3,2-b]­pyrroles
and Furo­[3,2-b]­pyridines. J. Org. Chem..

[19] Felletti S., Spedicato M., Bozza D., De Luca C., Presini F., Giovannini P. P., Carraro M., Macis M., Cavazzini A., Catani M. (2023). Dimethyl carbonate as a green alternative to acetonitrile
in reversed-phase liquid chromatography. Part I: Separation of small
molecules. J. Chromatogr. A.

[20] Lebœuf D., Marin L., Michelet B., Perez-Luna A., Guillot R., Schulz E., Gandon V. (2016). Harnessing
the Lewis
Acidity of HFIP through its Cooperation with a Calcium­(II) Salt: Application
to the Aza-Piancatelli Reaction. Chem. - Eur.
J..

[21] Huang W., He D., Jiang H., Wu W. (2025). Recent advances in the transformation
of nitriles into diverse N-heterocycles. Chem.
Soc. Rev..

[22] Chen Z., Cai Q., Boni Y. T., Liu W., Fu J., Davies H. M. L. (2023). N-Phthalimide
as a Site-Protecting and Stereodirecting Group in Rhodium-Catalyzed
C–H Functionalization with Donor/Acceptor Carbenes. Org. Lett..

[23] Han S., Jones R. A., Quiclet-Sire B., Zard S. Z. (2014). A convergent route
to functional protected amines, diamines, and β-amino acids. Tetrahedron.

[24] Haubenreisser S., Niggemann M. (2011). Calcium-Catalyzed
Direct Amination of π-Activated
Alcohols. Adv. Synth. Catal..

[25] Indukuri K., Unnava R., Deka M. J., Saikia A. K. (2013). Stereoselective
Synthesis of Amido and Phenyl Azabicyclic Derivatives via a Tandem
Aza Prins-Ritter/Friedel–Crafts Type Reaction of Endocyclic
N-Acyliminium Ions. J. Org. Chem..

[26] Ghosh D., Saravanan S., Gupta N., Abdi S. H. R., Khan N.-u. H., Kureshy R. I., Bajaj H. C. (2014). Phosphotungstic Acid as an Efficient
Catalyst for Allylation of Isatins and N-tert-Butyloxycarbonylamido
Sulfones Under Solvent-Free Conditions. Asian.
J. Org. Chem..

[27] Vilaivan T., Winotapan C., Banphavichit V., Shinada T., Ohfune Y. (2005). Indium-Mediated
Asymmetric Barbier-Type Allylation of Aldimines in Alcoholic Solvents:
Synthesis of Optically Active Homoallylic Amines. J. Org. Chem..

[28] Delaye P.-O., Ahari Mh., Vasse J.-L., Szymoniak J. (2010). A straightforward
access to pyrrolidine-based ligands for asymmetric synthesis. Tetrahedron: Asymmetry.

[29] Peng B., Ma J., Guo J., Gong Y., Wang R., Zhang Y., Zeng J., Chen W.-W., Ding K., Zhao B. (2022). A Powerful
Chiral Super Brønsted C–H Acid for Asymmetric Catalysis. J. Am. Chem. Soc..

[30] Peng X., Wang K.-H., Huang D., Wang J., Wang Y., Su Y., Hu Y., Fu Y. (2017). Tin powder-promoted diastereoselective
allylation of chiral acylhydrazones. Appl. Organomet.
Chem..

[31] Ollevier T., Li Z. (2009). Bismuth Triflate-Catalyzed
Addition of Allylsilanes to N-Alkoxycarbonylamino
Sulfones: Convenient Access to 3-Cbz-Protected Cyclohexenylamines. Adv. Synth. Catal..

[32] Song Q.-Y., Yang B.-L., Tian S.-K. (2007). FeSO4·7H2O-Catalyzed
Four-Component
Synthesis of Protected Homoallylic Amines. J.
Org. Chem..

[33] Yi Z.-J., Sun J.-T., Yang T.-Y., Yu X.-Y., Han X.-L., Wei B.-G. (2022). Cu­(OTf)­2-catalyzed C3 aza-Friedel–Crafts alkylation
of indoles with N,O-acetals. Org. Biomol. Chem..

