# Supplementary material for: Access to α‑Functionalized Amines via Calcium-Catalyzed Deacetylations
Source: J Org Chem. 2026 Mar 23;91(13):4698–709. doi: 10.1021/acs.joc.5c03185 (PMC13054857; doi:10.1021/acs.joc.5c03185)

Supporting Information
Access to α-functionalized amines via calcium catalyzed deacetylations.

Michael P. Cameron and Mark G. McLaughlin*.

School of Chemistry and Chemical Engineering, Queen's University Belfast, Belfast, United Kingdom, BT9 5AG.

*Corresponding Author: mark.mclaughlin@qub.ac.uk

Table of Contents

General Information and Procedures	S2 – S4
Synthesis of Starting Materials (**1a – 1p**)	S5 – S14
Synthesis of Homoallyl Phth-*N* amines (**2a – 2o**)	S15 – S23
Synthesis of Other α-Functionalised Phth-N amines (**3a – 3o**)	S23 – S31
Synthesis of Phth protected α-aminonitriles (**4a – 4l**)	S32 – S38
Synthesis of *N*-Boc α-functionalised amines (**5a – 5i**)	S38 – S44
Synthesis of *N*-Protected α-functionalised amines (**6a – 6i**)	S44 – S50
References	S50 – S51
^{1}H and ^{13}C NMR Spectra	S52 – S128

Experimental

General Information

Solvents & reagents were purchased in the highest purity available from Acros Organics, Alfa Aesar, Fluorochem, TCI, Fisher Scientific or Merck. All solvents were purchased from commercial sources and used without purification (reagent grade). Metal salts and ligands were stored in a desiccator when not in use. Anhydrous solvent was prepared by storing solvent over activated 4Å MS for 72 hours. Standard vacuum line techniques were used, and glassware was oven dried prior to use. Organic solvents were dried during workup using anhydrous $MgSO_4$. All reactions were performed using DrySyn heating mantles and pressure regulated vials or round bottom flasks.

Thin Layer Chromatography (TLC) was carried out using aluminium plates coated with 60 F_{254} silica gel. Plates were visualised using UV light (254 or 365 nm) and developed with iodine, basic permanganate solution or ninhydrin. Flash column chromatography (FCC) was performed on Fluorochem Silica gel 60, 40–63 microns RE as the stationary phase and the solvents employed were of reagent grade.

^{1}H NMR spectroscopic data were obtained at 400 MHz (Bruker Ultrashield 400 Plus) or 600 MHz (Bruker Ultrashield 600 Plus) and ^{13}C NMR data were obtained at 100 MHz (Bruker Ultrashield 400 Plus) or 151 MHz (Bruker Ultrashield 600 Plus) at 298 K. Infrared spectra were recorded on an Agilent Technologies Cary 630 FTIR spectrometer. High resolution mass spectrometry data was recorded using electron spray ionization (ESI) on an Agilent 6560 Ion Mobility LC/Q-TOF mass spectrometer.

General Procedures

General Procedure A (adapted from literature procedure)[1]

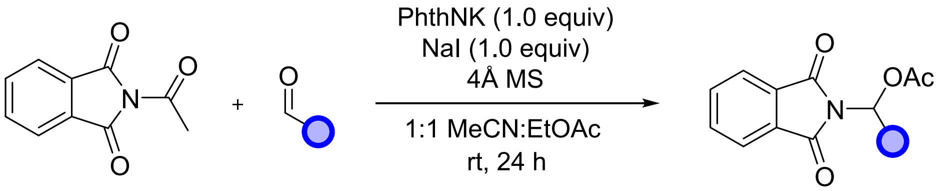

In a screw top vial, aldehyde (2.0 equiv) was added to a mixture of *N*-acyl phthalimide (1.0 equiv), PhthNK (1.0 equiv), NaI (1.0 equiv) and 4 Å MS (equal weight of PhthNK) in 1:1 mixture of dry MeCN and dry EtOAc (0.5 M). The mixture was stirred at room temperature for 24 hours, after which it is diluted in Et_2O, washed with sat. NH_4Cl soln, 1M NaOH (x3) and brine. The organic layer was dried over $MgSO_4$, filtered, concentrated and purified by FCC (EtOAc/Hex).

General procedure B (literature procedure)[2]

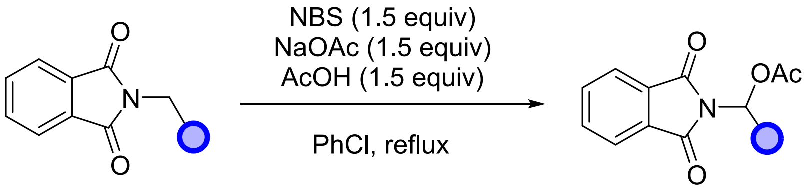

In a round bottom flask, Phth – protected amine (1.0 equiv) was dissolved in PhCl (0.25 M) and NBS (1.5 equiv), NaOAc (1.5 equiv) and AcOH (1.5 equiv) were added to reaction solution. With a condenser fitted, the reaction was refluxed overnight with constant stirring. After cooling to room temperature, the reaction solvent was removed *in vacuo* and the residue is redissolved in EtOAc and washed with sat $NaHCO_3$. The organic layer was then washed with brine and dried over Na_2SO_4, filtered, concentrated and purified by FCC (EtOAc/Hex).

General procedure C*

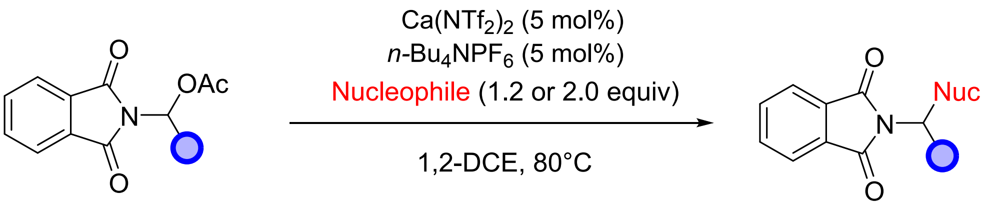

In a screw top vial, α-acetate phthalimide (1.0 equiv), $Ca(NTf_2)_2$ (5 mol%), and *n*Bu_4NPF_6 (5 mol%) were dissolved in 1,2-DCE (0.2 M). Nucleophile (1.2 or 2.0 equiv) was added and the reaction was heated at 80°C until complete by TLC. The reaction mixture was then concentrated *in vacuo* and purified by FCC (EtOAc/Hex).

*When using TMSCN as a nucleophile, upon completion, the reaction was quenched with sat. $NaHCO_3$ and extracted with DCM x3. The combined organic fractions were dried over $MgSO_4$, filtered, concentrated and purified by FCC (EtOAc/Hex).

General procedure D

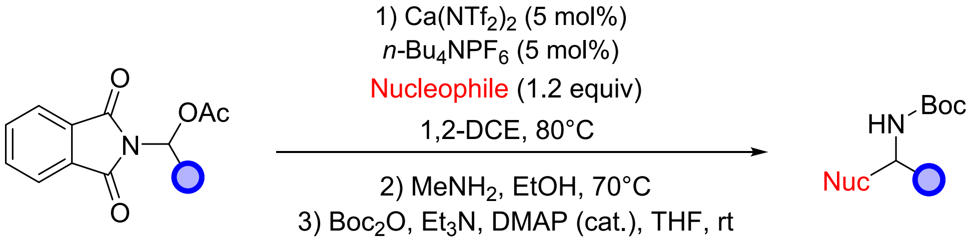

In a screw top vial, α-acetate phthalimide (1.0 equiv), $Ca(NTf_2)_2$ (5 mol%), and *n*Bu_4NPF_6 (5 mol%) were dissolved in 1,2-DCE (0.2M). Nucleophile (1.2 equiv) was added and the reaction was heated at 80 °C until complete by TLC.

The reaction mixture was then concentrated *in vacuo* and resuspended in EtOH (0.1M), then $MeNH_2$ (33 wt% in EtOH) (5 equiv) was added and the reaction was heated at 70 °C until the α-acetate phthalimide was full consumed (TLC).

The reaction mixture was again concentrated *in vacuo* and resuspended in Et_2O and filtered through cotton wool into another screw top vial, and the Et_2O was gently evaporated. The residue was then redissolved in dry THF (0.2M), and Boc_2O (2.0 equiv), Et_3N (1.0 equiv) and DMAP (cat.) were added and the reaction was stirred at room temperature. After completion, the reaction mixture was concentrated and purified by FCC (EtOAc/Hex).

General procedure E

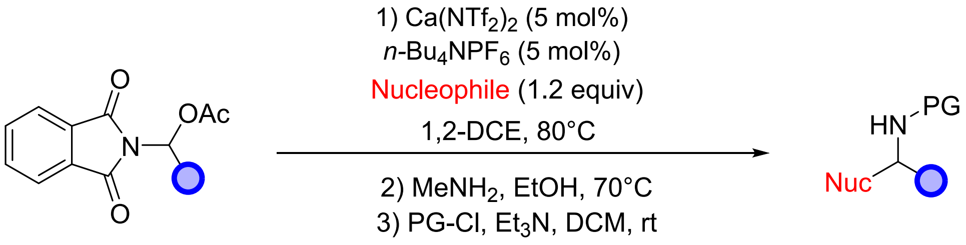

In a screw top vial, α-acetate phthalimide (1.0 equiv), $Ca(NTf_2)_2$ (5 mol%), and *n*Bu_4NPF_6 (5 mol%) were dissolved in 1,2-DCE (0.2M). Nucleophile (1.2 equiv) was added and the reaction was heated at 80 °C until complete by TLC.

The reaction mixture was then concentrated *in vacuo* and resuspended in EtOH (0.1M), then $MeNH_2$ (33 wt% in EtOH) (5 equiv) was added and the reaction was heated at 70 °C until the α-acetate phthalimide was full consumed (TLC).

The reaction mixture was again concentrated *in vacuo* and resuspended in Et_2O and filtered through cotton wool into another screw top vial, and the Et_2O was gently evaporated. The residue was then redissolved in dry DCM (0.2M), and PG-Cl (1.2 equiv) and Et_3N (1.0 equiv) were added and the reaction was stirred at room temperature. After completion, the reaction mixture was concentrated and purified by FCC (EtOAc/Hex).

Synthesis of Starting Materials

(1,3-dioxoisoindolin-2-yl)(phenyl)methyl acetate (1a)

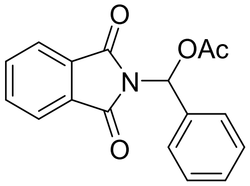

The title compound was prepared according to general procedure **A** from *N*-acyl phthalimide (500 mg, 2.64 mmol), PhthNK (500 mg, 2.64 mmol), NaI (400 mg, 2.64 mmol), 4 Å MS (500 mg) and benzaldehyde (0.54 mL, 5.28 mmol) in 1:1 MeCN:EtOAc (4.3 mL). Following completion of the reaction (24 h) and work-up, purification by FCC (1:4 EtOAc:Hex) afforded the pure compound as a white solid (577 mg, 74 %).

RF (1:3 EtOAc:Hex): 0.39

^{1}H NMR (400 MHz, $CDCl_3$) δ 7.89 – 7.83 (m, 2H), 7.77 – 7.71 (m, 2H), 7.68 (s, 1H), 7.56 (d, *J* = 7.4 Hz, 2H), 7.43 – 7.31 (m, 3H), 2.22 (s, 3H).

^{13}C{H}NMR (101 MHz, $CDCl_3$) δ 169.3, 166.3, 135.0, 134.5, 131.6, 129.0, 128.5, 126.4, 123.8, 74.2, 20.8.

*Data in accordance with literature.[1]

(1,3-dioxoisoindolin-2-yl)(4-methoxyphenyl)methyl acetate (1b)

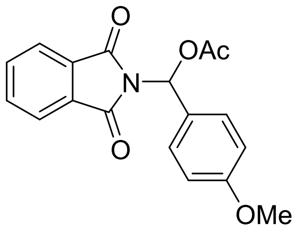

The title compound was prepared according to general procedure **A** from *N*-acyl phthalimide (500 mg, 2.64 mmol), PhthNK (500 mg, 2.64 mmol), NaI (400 mg, 2.64 mmol), 4 Å MS (500 mg) and 4-methoxybenzaldehyde (0.64 mL, 5.28 mmol) in 1:1 MeCN:EtOAc (4.3 mL). Following completion of the reaction (24 h) and work-up, purification by FCC (1:6 to 1:4 EtOAc:Hex) afforded the pure compound as a white solid (375 mg, 44 %).

RF (1:4 EtOAc:Hex): 0.24

^{1}H NMR (400 MHz, $CDCl_3$) δ 7.88 – 7.83 (m, 2H), 7.76 – 7.70 (m, 2H), 7.62 (s, 1H), 7.55 – 7.50 (m, 2H), 6.93 – 6.87 (m, 2H), 3.80 (s, 3H), 2.19 (s, 3H).

$^{13}C\{H\}$ NMR (101 MHz, $CDCl_3$) δ 169.3, 166.4, 160.0, 134.4, 131.6, 128.0, 127.2, 123.7, 113.81, 74.3, 55.3, 20.8.

*Data in accordance with literature.[1]

(1,3-dioxoisoindolin-2-yl)(p-tolyl)methyl acetate (1c)

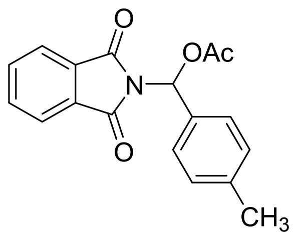

The title compound was prepared according to general procedure **A** from *N*-acyl phthalimide (500 mg, 2.64 mmol), PhthNK (500 mg, 2.64 mmol), NaI (400 mg, 2.64 mmol), 4 Å MS (500 mg) and *p*-tolualdehyde (0.62 mL, 5.28 mmol) in 1:1 MeCN:EtOAc (4.3 mL). Following completion of the reaction (24 h) and work-up, purification by FCC (1:6 to 1:4 EtOAc:Hex) afforded the pure compound as a white solid (495 mg, 61%).

RF (1:4 EtOAc:Hex): 0.31

^{1}H NMR (400 MHz, $CDCl_3$) δ 7.88 – 7.82 (m, 2H), 7.76 – 7.70 (m, 2H), 7.65 (s, 1H), 7.45 (d, *J* = 8.1 Hz, 2H), 7.19 (d, *J* = 8.0 Hz, 2H), 2.34 (s, 3H), 2.20 (s, 3H).

$^{13}C\{H\}$ NMR (101 MHz, $CDCl_3$) δ 169.3, 166.4, 138.9, 134.4, 132.1, 131.6, 129.2, 126.3, 123.8, 74.3, 21.2, 20.8.

*Data in accordance with literature.[1]

(1,3-dioxoisoindolin-2-yl)(4-fluorophenyl)methyl acetate (1d)

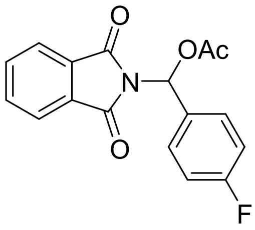

The title compound was prepared according to general procedure **A** from *N*-acyl phthalimide (500 mg, 2.64 mmol), PhthNK (500 mg, 2.64 mmol), NaI (400 mg, 2.64 mmol), 4 Å MS (500 mg) and 4-fluorobenzaldehyde (0.57 mL, 5.28 mmol) in 1:1 MeCN:EtOAc (4.3 mL). Following completion of the reaction (24 h) and work-up, purification by FCC (1:4 EtOAc:Hex) afforded the pure compound as a white solid (583 mg, 70%).

RF (1:4 EtOAc:Hex): 0.29

^{1}H NMR (400 MHz, $CDCl_3$) δ 7.90 – 7.84 (m, 2H), 7.78 – 7.72 (m, 2H), 7.64 (s, 1H), 7.60 – 7.54 (m, 2H), 7.11 – 7.04 (m, 2H), 2.21 (s, 3H).

$^{13}C\{H\}$ NMR (101 MHz, $CDCl_3$) δ 169.1 (s), 166.3 (d, J = 6.6 Hz), 162.9 (d, J = 248.1 Hz), 134.5 (s), 131.5 (s), 131.0 (d, J = 3.3 Hz), 128.5 (d, J = 8.4 Hz), 123.8 (s), 115.5 (d, J = 21.9 Hz), 73.8 (s), 20.8 (s).

^{19}F NMR (376 MHz, $CDCl_3$) δ -112.47.

IR v_{max} (cm^{-1}): 3062, 2965, 1753, 1716, 1509, 1354, 1220, 1026, 833, 719

HRMS (ESI) m/z: [M – OAc]+ Calcd for $C_{15}H_9FNO_2$ 254.0612; Found 254.0623

(1,3-dioxoisoindolin-2-yl)(4-(trifluoromethyl)phenyl)methyl acetate (1e)

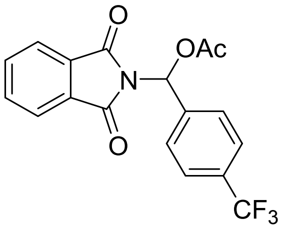

The title compound was prepared according to general procedure **B** from *N*-Phth-(4-trifluoromethyl)benzyl amine (1.00 g, 3.28 mmol), NBS (667 mg, 4.91 mmol), NaOAc (403 mg, 4.91 mmol) and AcOH (0.28 mL, 4.91 mmol) in chlorobenzene (14 mL). Following completion of the reaction (18 h) and work-up, purification by FCC (1:7 EtOAc:Hex) afforded the pure compound as a white solid (140 mg, 12 %).

RF (1:3 EtOAc:Hex): 0.39

^{1}H NMR (400 MHz, $CDCl_3$) δ 7.91 – 7.85 (m, 2H), 7.79 – 7.74 (m, 2H), 7.71 (s, 1H), 7.67 (q, J = 8.8 Hz, 4H), 2.23 (s, 3H).

$^{13}C\{H\}$ NMR (101 MHz, $CDCl_3$) δ 169.1 (s), 166.2 (s), 138.9 (s), 134.7 (s), 134.3 (s), 131.4(s), 131.1 (q, J = 32.7 Hz), 126.9 (s), 125.5 (q, J = 3.8 Hz), 124.0 (s), 73.3 (s), 20.7 (s).

^{19}F NMR (376 MHz, $CDCl_3$) δ -62.76.

IR v_{max} (cm^{-1}): 2957, 1766, 1720, 1362, 1323, 1209, 1166, 1120, 1068, 1029, 997, 718

HRMS (ESI) m/z: [M - OAc]+ Calcd for $C_{16}H_9F_3NO_2$ 304.0580; Found 304.0581

(1,3-dioxoisoindolin-2-yl)(2-methoxyphenyl)methyl acetate (1f)

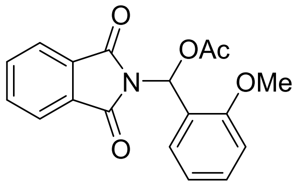

The title compound was prepared according to general procedure **A** from *N*-acyl phthalimide (500 mg, 2.64 mmol), PhthNK (500 mg, 2.64 mmol), NaI (400 mg, 2.64 mmol), 4 Å MS (500 mg) and 2-methoxybenzaldehyde (0.64 mL, 5.28 mmol) in 1:1 MeCN:EtOAc (4.3 mL). Following completion of the reaction (24 h) and work-up, purification by FCC (1:6 to 1:4 EtOAc:Hex) afforded the pure compound as a white solid (58 mg, 7%).

RF (1:3 EtOAc:Hex): 0.26

^{1}H NMR (400 MHz, $CDCl_3$) δ 7.88 (s, 1H), 7.86 – 7.81 (m, 2H), 7.79 (dd, *J* = 7.7, 0.9 Hz, 1H), 7.74 – 7.69 (m, 2H), 7.33 (td, *J* = 8.0, 1.5 Hz, 1H), 7.04 (t, *J* = 7.6 Hz, 1H), 6.84 (d, *J* = 8.1 Hz, 1H), 3.75 (s, 3H), 2.20 (s, 3H).

^{13}C{H} NMR (101 MHz, $CDCl_3$) δ 168.9, 166.2, 156.3, 134.2, 131.7, 130.1, 128.2, 123.6, 122.7, 120.0, 110.4, 70.3, 55.5, 20.8.

IR v_{max} (cm^{-1}): 2982, 2942, 1750, 1716, 1464, 1349, 1217, 1021, 749, 712

HRMS (ESI) m/z: [M + Na]+ Calcd for $C_{18}H_{15}NO_5Na$ 348.0848; Found 348.0854

(2-bromophenyl)(1,3-dioxoisoindolin-2-yl)methyl acetate (1g)

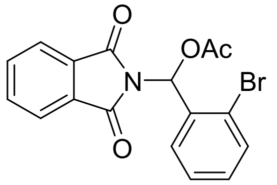

The title compound was prepared according to general procedure **A** from *N*-acyl phthalimide (500 mg, 2.64 mmol), PhthNK (500 mg, 2.64 mmol), NaI (400 mg, 2.64 mmol), 4 Å MS (500 mg) and 2-bromobenzaldehyde (0.62 mL, 5.28 mmol) in 1:1 MeCN:EtOAc (4.3 mL). Following completion of the reaction (24 h) and work-up, purification by FCC (1:4 EtOAc:Hex then 100% EtOAc) afforded the pure compound as a white solid (703 mg, 71%).

RF (1:4 EtOAc:Hex): 0.14

^{1}H NMR (400 MHz, $CDCl_3$) δ 7.89 (dd, *J* = 8.0, 1.3 Hz, 1H), 7.87 – 7.83 (m, 2H), 7.78 (s, 1H), 7.77 – 7.72 (m, 2H), 7.54 (dd, *J* = 8.0, 1.0 Hz, 1H), 7.42 (td, *J* = 7.8, 0.9 Hz, 1H), 7.24 (td, *J* = 7.8, 1.7 Hz, 1H), 2.21 (s, 3H).

$^{13}C\{H\}$ NMR (101 MHz, $CDCl_3$) δ 168.6, 166.1, 134.5, 133.6, 133.0, 131.4, 130.5, 129.6, 126.9, 123.8, 121.6, 73.7, 20.8.

IR v_{max} (cm^{-1}): 1776, 1748, 1722, 1470, 1381, 1347, 1222, 1015, 894, 713

HRMS (ESI) m/z: [M + Na]+ Calcd for $C_{17}H_{12}BrNO_4Na$ 395.9847; Found 395.9843

(3-chlorophenyl)(1,3-dioxoisoindolin-2-yl)methyl acetate (1h)

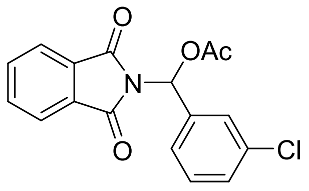

The title compound was prepared according to general procedure **A** from *N*-acyl phthalimide (500 mg, 2.64 mmol), PhthNK (500 mg, 2.64 mmol), NaI (400 mg, 2.64 mmol), 4 Å MS (500 mg) and 3-chlorobenzaldehyde (0.57 mL, 5.28 mmol) in 1:1 MeCN:EtOAc (4.3 mL). Following completion of the reaction (24 h) and work-up, purification by FCC (1:4 EtOAc:Hex) afforded the pure compound as a white solid (646 mg, 74%).

RF (1:4 EtOAc:Hex): 0.14

^1H NMR (400 MHz, $CDCl_3$) δ 7.90 – 7.84 (m, 2H), 7.79 – 7.73 (m, 2H), 7.63 (s, 1H), 7.57 (s, 1H), 7.45 – 7.39 (m, 1H), 7.35 – 7.30 (m, 2H), 2.22 (s, 3H).

$^{13}C\{H\}$ NMR (101 MHz, $CDCl_3$) δ 169.1, 166.2, 137.0, 134.6, 134.5, 131.5, 129.8, 129.2, 126.7, 124.6, 123.9, 73.3, 20.8.

IR v_{max} (cm^{-1}): 3080, 2969, 1750, 1716, 1356, 1334, 1230, 1116, 1032, 734, 682

HRMS (ESI) m/z: : [M – OAc]+ Calcd for $C_{15}H_9ClNO_2$ 270.0316; Found 270.0319

(3-bromo-2-fluorophenyl)(1,3-dioxoisoindolin-2-yl)methyl acetate (1i)

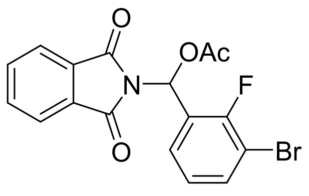

The title compound was prepared according to general procedure **A** from *N*-acyl phthalimide (500 mg, 2.64 mmol), PhthNK (500 mg, 2.64 mmol), NaI (400 mg, 2.64 mmol), 4 Å MS (500 mg) and 3-bromo-2-fluorobenzaldehyde (1.072 g, 5.28 mmol) in 1:1 MeCN:EtOAc (4.3 mL). Following completion of the reaction (24 h) and work-up, purification by FCC (1:4 EtOAc:Hex) afforded the pure compound as a white solid (531 mg, 51%).

RF (1:4 EtOAc:Hex): 0.22

^{1}H NMR (400 MHz, $CDCl_3$) δ 7.90 – 7.84 (m, 3H), 7.80 – 7.73 (m, 3H), 7.59 – 7.51 (m, 1H), 7.12 (t, *J* = 8.0 Hz, 1H), 2.21 (s, 3H).

^{13}C{H} NMR (101 MHz, $CDCl_3$) δ 168.6 (s), 165.9 (s), 155.9 (d, *J* = 249.6 Hz), 134.6 (s), 134.2 (s), 131.4 (s), 127.9 (d, *J* = 2.2 Hz), 124.8 (d, *J* = 4.5 Hz), 124.0 (s), 123.9 (s), 109.3 (d, *J* = 20.4 Hz), 68.9 (d, *J* = 3.4 Hz), 20.7 (s).

^{19}F NMR (376 MHz, $CDCl_3$) δ -110.42.

IR v_{max} (cm^{-1}): 3088, 1753, 1723, 1455, 1356, 1220, 1032, 721

HRMS (ESI) m/z: [M + Na]+ Calcd for $C_{17}H_{11}BrFNO_4Na$ 413.9753; Found 413.9744

(3,5-bis(trifluoromethyl)phenyl)(1,3-dioxoisoindolin-2-yl)methyl acetate (1j)

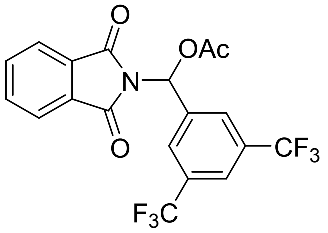

The title compound was prepared according to general procedure **A** from *N*-acyl phthalimide (500 mg, 2.64 mmol), PhthNK (500 mg, 2.64 mmol), NaI (400 mg, 2.64 mmol), 4 Å MS (500 mg) and 3,5-bis(trifluoromethyl)benzaldehyde (0.87 mL, 5.28 mmol) in 1:1 MeCN:EtOAc (4.3 mL). Following completion of the reaction (24 h) and work-up, purification by FCC (1:6 to 1:4 EtOAc:Hex) afforded the pure compound as a white solid (935 mg, 82%).

RF (1:4 EtOAc:Hex): 0.41

^{1}H NMR (400 MHz, $CDCl_3$) δ 8.04 (s, 2H), 7.93 – 7.86 (m, 3H), 7.81 – 7.76 (m, 2H), 7.72 (s, 1H), 2.25 (s, 3H).

^{13}C{H} NMR (101 MHz, $CDCl_3$) δ 168.8 (s), 166.0 (s), 137.7 (s), 134.9 (s), 132.0 (q, *J* = 33.7 Hz), 131.3 (s), 127.1 (d, *J* = 3.2 Hz), 124.1 (s), 123.2 – 122.9 (m), 121.7 (s), 74.1 – 71.0 (m), 22.9 – 18.7 (m).

^{19}F NMR (376 MHz, $CDCl_3$) δ -62.82.

IR v_{max} (cm^{-1}): 2989, 1763, 1720, 1274, 1172, 1122, 1029, 718

HRMS (ESI) m/z: [M – OAc]+ Calcd for $C_{17}H_8F_6NO_2$ 372.0454; Found 372.0450

(1,3-dioxoisoindolin-2-yl)(4-nitrophenyl)methyl acetate (1k)

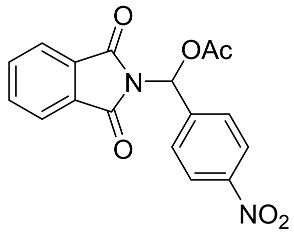

The title compound was prepared according to general procedure **A** from *N*-acyl phthalimide (500 mg, 2.64 mmol), PhthNK (500 mg, 2.64 mmol), NaI (400 mg, 2.64 mmol), 4 Å MS (500 mg) and 4-nitrobenzaldehyde (798 mg, 5.28 mmol) in 1:1 MeCN:EtOAc (4.3 mL). Following completion of the reaction (24 h) and work-up, purification by FCC (1:6 to 1:4 EtOAc:Hex) afforded the pure compound as a white solid (331 mg, 37%).

RF (1:4 EtOAc:Hex): 0.17

^1H NMR (400 MHz, $CDCl_3$) δ 8.25 (d, *J* = 8.9 Hz, 2H), 7.91 – 7.86 (m, 2H), 7.81 – 7.77 (m, 2H), 7.74 (d, *J* = 8.5 Hz, 2H), 7.73 (s, 1H), 2.25 (s, 3H).

$^{13}C\{H\}$ NMR (101 MHz, $CDCl_3$) δ 168.9, 166.1, 148.1, 141.9, 134.8, 131.4, 127.5, 124.0, 123.8, 72.9, 20.7.

*Data in accordance with literature.[1]

(1,3-dioxoisoindolin-2-yl)(naphthalen-2-yl)methyl acetate (1l)

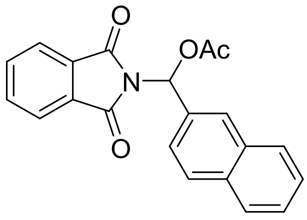

The title compound was prepared according to general procedure **A** from *N*-acyl phthalimide (500 mg, 2.64 mmol), PhthNK (500 mg, 2.64 mmol), NaI (400 mg, 2.64 mmol), 4 Å MS (500

mg) and 2-naphthaldehyde (825 mg, 5.28 mmol) in 1:1 MeCN:EtOAc (4.3 mL). Following completion of the reaction (24 h) and work-up, purification by FCC (1:5 EtOAc:Hex) afforded the pure compound as a white solid (702 mg, 77%).

RF (1:4 EtOAc:Hex): 0.34

^{1}H NMR (400 MHz, $CDCl_3$) δ 8.04 (s, 1H), 7.89 – 7.82 (m, 6H), 7.73 (dd, *J* = 5.5, 3.1 Hz, 2H), 7.66 (dd, *J* = 8.6, 1.8 Hz, 1H), 7.52 – 7.46 (m, 2H), 2.26 (s, 3H).

^{13}C{H} NMR (101 MHz, $CDCl_3$) δ 169.3, 166.4, 134.5, 133.4, 132.9, 132.4, 131.6, 128.4, 128.3, 127.7, 126.7, 126.5, 125.9, 123.8, 123.7, 74.4, 20.9.

IR v_{max} (cm^{-1}): 3053, 1746, 1716, 1347, 1211, 1116, 1019, 714

HRMS (ESI) m/z: [M + Na]+ Calcd for $C_{21}H_{15}NO_4Na$ 368.0899; Found 368.0891

(1,3-dioxoisoindolin-2-yl)(furan-2-yl)methyl acetate (1m)

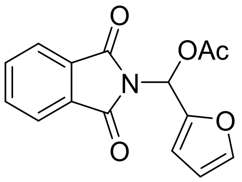

The title compound was prepared according to general procedure **A** from *N*-acyl phthalimide (500 mg, 2.64 mmol), PhthNK (500 mg, 2.64 mmol), NaI (400 mg, 2.64 mmol), 4 Å MS (500 mg) and 2-furfural (0.44 mL, 5.28 mmol) in 1:1 MeCN:EtOAc (4.3 mL). Following completion of the reaction (24 h) and work-up, purification by FCC (6:1 to 1:1 EtOAc:Hex) afforded the pure compound as a white solid (527 mg, 70%).

RF (1:4 EtOAc:Hex): 0.23

^{1}H NMR (400 MHz, $CDCl_3$) δ 7.94 – 7.87 (m, 2H), 7.81 – 7.74 (m, 2H), 7.70 (s, 1H), 7.40 (d, *J* = 0.7 Hz, 1H), 6.58 (d, *J* = 3.3 Hz, 1H), 6.40 (dd, *J* = 3.3, 1.8 Hz, 1H), 2.18 (s, 3H).

^{13}C{H} NMR (101 MHz, $CDCl_3$) δ 169.0, 166.0, 147.3, 143.0, 134.6, 131.6, 123.9, 110.7, 109.4, 68.8, 20.7.

*Data in accordance with literature.[1]

(1,3-dioxoisoindolin-2-yl)(thiophen-2-yl)methyl acetate (1n)

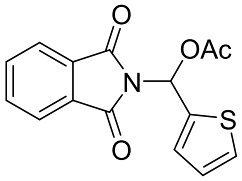

The title compound was prepared according to general procedure **A** from *N*-acyl phthalimide (500 mg, 2.64 mmol), PhthNK (500 mg, 2.64 mmol), NaI (400 mg, 2.64 mmol), 4 Å MS (500 mg) and 2-thiophenecarboxaldehyde (0.49 mL, 5.28 mmol) in 1:1 MeCN:EtOAc (4.3 mL). Following completion of the reaction (24 h) and work-up, purification by FCC (1:4 EtOAc:Hex) afforded the pure compound as a yellow solid (563 mg, 71%).

RF (1:4 EtOAc:Hex): 0.29

^{1}H NMR (400 MHz, $CDCl_3$) δ 7.92 – 7.85 (m, 3H), 7.80 – 7.72 (m, 2H), 7.35 (dd, *J* = 5.1, 1.2 Hz, 1H), 7.24 (d, *J* = 3.5 Hz, 1H), 6.99 (dd, *J* = 5.1, 3.6 Hz, 1H), 2.17 (s, 3H).

^{13}C{H} NMR (101 MHz, $CDCl_3$) δ 169.0, 165.9, 137.4, 134.6, 131.5, 127.4, 127.0, 126.6, 123.9, 71.3, 20.7.

IR v_{max} (cm^{-1}): 2953, 1757, 1718, 1351, 1209, 1114, 1019, 706

HRMS (ESI) m/z: [M - OAc]+ Calcd for $C_{13}H_8NO_2S$ 242.0270; Found 242.0290

cyclohexyl(1,3-dioxoisoindolin-2-yl)methyl acetate (1o)

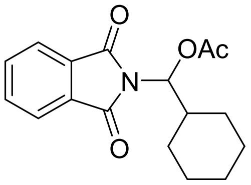

The title compound was prepared according to general procedure **A** from *N*-acyl phthalimide (500 mg, 2.64 mmol), PhthNK (500 mg, 2.64 mmol), NaI (400 mg, 2.64 mmol), 4 Å MS (500 mg) and cyclohexanecarboxaldehyde (0.64 mL, 5.28 mmol) in 1:1 MeCN:EtOAc (4.3 mL). Following completion of the reaction (24 h) and work-up, purification by FCC (4:1 EtOAc:Hex) afforded the pure compound as a white solid (591 mg, 74%).

RF (1:4 EtOAc:Hex): 0.39

^{1}H NMR (400 MHz, $CDCl_3$) δ 7.91 – 7.85 (m, 2H), 7.79 – 7.71 (m, 2H), 6.31 (d, *J* = 10.4 Hz, 1H), 2.58 (qt, *J* = 11.3, 3.6 Hz, 1H), 2.09 (s, 3H), 2.00 – 1.90 (m, 1H), 1.85 – 1.74 (m, 1H), 1.73 – 1.60 (m, 2H), 1.46 (d, *J* = 12.7 Hz, 1H), 1.36 – 0.95 (m, 5H).

^{13}C{H} NMR (101 MHz, $CDCl_3$) δ 169.8, 166.9, 134.4, 131.5, 123.7, 78.1, 38.0, 29.2, 27.9, 25.93, 25.20, 25.08, 20.7.

*Data in accordance with literature.[1]

cyclopropyl(1,3-dioxoisoindolin-2-yl)methyl acetate (1p)

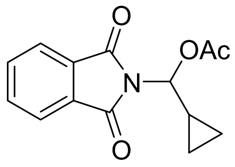

The title compound was prepared according to general procedure **A** from *N*-acyl phthalimide (500 mg, 2.64 mmol), PhthNK (500 mg, 2.64 mmol), NaI (400 mg, 2.64 mmol), 4 Å MS (500 mg) and cyclopropanecarboxaldehyde (0.39 mL, 5.28 mmol) in 1:1 MeCN:EtOAc (4.3 mL). Following completion of the reaction (24 h) and work-up, purification by FCC (4:1 EtOAc:Hex) afforded the pure compound as a white solid (478 mg, 70%).

RF (1:4 EtOAc:Hex): 0.32

^{1}H NMR (400 MHz, $CDCl_3$) δ 7.92 – 7.86 (m, 2H), 7.81 – 7.72 (m, 2H), 5.85 (d, *J* = 9.7 Hz, 1H), 2.20 – 2.11 (m, 1H), 2.10 (s, 3H), 0.80 – 0.63 (m, 2H), 0.63 – 0.38 (m, 2H).

^{13}C{H} NMR (101 MHz, $CDCl_3$) δ 169.4, 166.7, 134.4, 131.6, 123.7, 78.5, 20.8, 13.0, 4.7, 2.4.

*Data in accordance with literature.[1]

Homoallyl Phth-N amines

2-(1-phenylbut-3-en-1-yl)isoindoline-1,3-dione (2a)

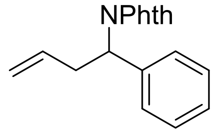

The title compound was prepared according to general procedure **C** from (1,3-dioxoisoindolin-2-yl)(phenyl)methyl acetate (100 mg, 0.339 mmol), $Ca(NTf_2)_2$ (10 mg, 0.017 mmol), *n*Bu_4NPF_6 (6.5 mg, 0.017 mmol) and allyl TMS (46 µL, 0.406 mmol) in 1,2- DCE (1.69 mL), reacting for 1 hr then being isolated by FCC (1:12 EtOAc:Hex) as a colourless viscous oil (79 mg, 84%).

RF (1:3 EtOAc:Hex): 0.74

^{1}H NMR (400 MHz, $CDCl_3$) δ 7.81 – 7.75 (m, 2H), 7.68 – 7.63 (m, 2H), 7.57 – 7.52 (m, 2H), 7.37 – 7.30 (m, 2H), 7.29 – 7.23 (m, 1H), 5.77 (dddd, *J* = 17.1, 10.2, 8.3, 5.6 Hz, 1H), 5.45 (dd, *J* = 10.6, 5.9 Hz, 1H), 5.14 (ddd, *J* = 17.1, 2.9, 1.7 Hz, 1H), 5.01 (d, *J* = 10.1 Hz, 1H), 3.48 – 3.36 (m, 1H), 3.02 – 2.91 (m, 1H).

^{13}C{H} NMR (101 MHz, $CDCl_3$) δ 168.3, 139.2, 134.4, 133.9, 131.8, 128.6, 128.1, 127.9, 123.2, 118.3, 54.4, 35.3.

*Data in accordance with literature.[3]

2-(1-(4-methoxyphenyl)but-3-en-1-yl)isoindoline-1,3-dione (2b)

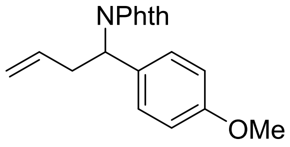

The title compound was prepared according to general procedure **C** from (1,3-dioxoisoindolin-2-yl)(4-methoxyphenyl)methyl acetate (75 mg, 0.231 mmol), $Ca(NTf_2)_2$ (6.9 mg, 0.012 mmol), *n*Bu_4NPF_6 (4.5 mg, 0.012 mmol) and allyl TMS (44 µL, 0.277 mmol) in 1,2- DCE (1.2 mL), reacting for 1 hr then being isolated by FCC (1:9 EtOAc:Hex) as a colourless viscous oil (60 mg, 85%).

RF (1:4 EtOAc:Hex): 0.56

^{1}H NMR (400 MHz, $CDCl_3$) δ 7.82 – 7.74 (m, 2H), 7.71 – 7.62 (m, 2H), 7.55 – 7.45 (m, 2H), 6.92 – 6.82 (m, 2H), 5.76 (dddd, *J* = 17.0, 10.2, 8.2, 5.7 Hz, 1H), 5.39 (dt, *J* = 17.1, 8.5 Hz, 1H), 5.13 (dd, *J* = 17.1, 1.2 Hz, 1H), 5.00 (d, *J* = 10.1 Hz, 1H), 3.77 (s, 3H), 3.44 – 3.31 (m, 1H), 3.01 – 2.89 (m, 1H).

^{13}C{H} NMR (101 MHz, $CDCl_3$) δ 168.3, 159.2, 134.5, 133.9, 131.9, 131.4, 129.4, 123.2, 118.1, 113.8, 55.2, 53.9, 35.5.

*Data in accordance with literature.[3]

2-(1-(p-tolyl)but-3-en-1-yl)isoindoline-1,3-dione (2c)

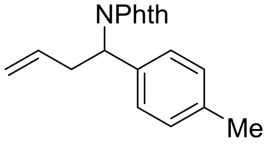

The title compound was prepared according to general procedure **C** from (1,3-dioxoisoindolin-2-yl)(p-tolyl)methyl acetate (75 mg, 0.242 mmol), $Ca(NTf_2)_2$ (7.3 mg, 0.012 mmol), $n$$Bu_4NPF_6$ (4.7 mg, 0.012 mmol) and allyl TMS (46 μL, 0.290 mmol) in 1,2- DCE (1.2 mL), reacting for 1 hr then being isolated by FCC (1:12 EtOAc:Hex) as a colourless viscous oil (55 mg, 78%).

RF (1:4 EtOAc:Hex): 0.68

^{1}H NMR (400 MHz, $CDCl_3$) δ 7.82 – 7.75 (m, 2H), 7.69 – 7.63 (m, 2H), 7.43 (d, J = 8.1 Hz, 2H), 7.13 (d, J = 8.0 Hz, 2H), 5.77 (dddd, J = 17.0, 10.2, 8.3, 5.7 Hz, 1H), 5.41 (dd, J = 10.5, 5.9 Hz, 1H), 5.13 (dd, J = 17.1, 1.2 Hz, 1H), 5.00 (d, J = 10.1 Hz, 1H), 3.45 – 3.33 (m, 1H), 3.01 – 2.91 (m, 1H), 2.30 (s, 3H).

^{13}C{H} NMR (101 MHz, $CDCl_3$) δ 168.3, 137.6, 136.2, 134.5, 133.9, 131.9, 129.2, 128.0, 123.2, 118.1, 54.1, 35.4, 21.1.

IR v_{max} (cm^{-1}): 2961, 2918, 1766, 1705, 1382, 1351, 1326, 1071, 713

HRMS (ESI) m/z: [M + H]+ Calcd for $C_{19}H_{18}NO_2$ 292.1338; Found 292.1333

2-(1-(4-fluorophenyl)but-3-en-1-yl)isoindoline-1,3-dione (2d)

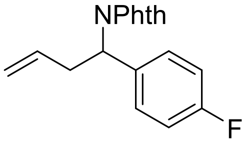

The title compound was prepared according to general procedure **C** from (1,3-dioxoisoindolin-2-yl)(4-fluorophenyl)methyl acetate (75 mg, 0.239 mmol), $Ca(NTf_2)_2$ (7.2 mg, 0.012 mmol), $n$$Bu_4NPF_6$ (4.6 mg, 0.012 mmol) and allyl TMS (46 μL, 0.287 mmol) in 1,2- DCE (1.2 mL), reacting for 1 hr then being isolated by FCC (1:12 EtOAc:Hex) as a colourless viscous oil (54 mg, 77%).

RF (1:4 EtOAc:Hex): 0.67

^{1}H NMR (400 MHz, $CDCl_3$) δ 7.82 – 7.77 (m, 2H), 7.71 – 7.65 (m, 2H), 7.56 – 7.49 (m, 2H), 7.06 – 6.96 (m, 2H), 5.75 (dddd, J = 17.1, 10.2, 8.2, 5.7 Hz, 1H), 5.41 (dd, J = 10.4, 6.1 Hz, 1H), 5.13 (ddd, J = 17.1, 2.9, 1.6 Hz, 1H), 5.01 (d, J = 10.0 Hz, 1H), 3.46 – 3.30 (m, 1H), 3.02 – 2.89 (m, 1H).

$^{13}C\{H\}$ NMR (101 MHz, $CDCl_3$) δ 168.6 (s), 162.6 (d, *J* = 246.6 Hz), 135.3 (d, *J* = 3.3 Hz), 134.4 (s), 134.3 (s), 132.1 (s), 130.2 (d, *J* = 8.1 Hz), 123.6 (s), 118.7 (s), 115.7 (d, *J* = 21.4 Hz), 54.0 (s), 35.8 (s).

^{19}F NMR (376 MHz, $CDCl_3$) δ -114.22.

IR v_{max} (cm^{-1}): 3075, 2922, 1768, 1705, 1507, 1384, 1351, 1328, 1223, 1071, 836, 713

HRMS (ESI) m/z: [M + H]+ Calcd for $C_{18}H_{15}FNO_2$ 296.1087; Found 296.1078

2-(1-(4-(trifluoromethyl)phenyl)but-3-en-1-yl)isoindoline-1,3-dione (2e)

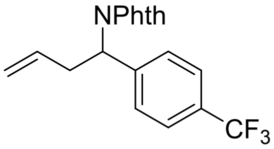

The title compound was prepared according to general procedure **C** from (1,3-dioxoisoindolin-2-yl)(4-(trifluoromethyl)phenyl)methyl acetate (49 mg, 0.135 mmol), $Ca(NTf_2)_2$ (4.1 mg, 0.007 mmol), *n*Bu_4NPF_6 (2.6 mg, 0.007 mmol) and allyl TMS (26 µL, 0.162 mmol) in 1,2- DCE (0.68 mL), reacting overnight then being isolated by FCC (1:12 EtOAc:Hex) as a colourless viscous oil (26 mg, 56%).

RF (1:4 EtOAc:Hex): 0.68

^{1}H NMR (400 MHz, $CDCl_3$) δ 7.85 – 7.78 (m, 2H), 7.74 – 7.69 (m, 2H), 7.66 (d, *J* = 8.4 Hz, 2H), 7.59 (d, *J* = 8.3 Hz, 2H), 5.76 (dddd, *J* = 17.0, 10.1, 8.2, 5.7 Hz, 1H), 5.49 (dd, *J* = 10.5, 6.0 Hz, 1H), 5.15 (dd, *J* = 17.1, 1.2 Hz, 1H), 5.04 (d, *J* = 10.1 Hz, 1H), 3.49 – 3.32 (m, 1H), 3.06 – 2.92 (m, 1H).

$^{13}C\{H\}$ NMR (101 MHz, $CDCl_3$) δ 168.2 (s), 143.0 (d, *J* = 1.2 Hz), 134.1 (s), 133.7 (s), 131.6 (s), 130.1 (q, *J* = 32.4 Hz), 128.5 (s), 125.6 (q, *J* = 3.8 Hz), 124.0 (q, *J* = 272.2 Hz), 123.4 (s), 118.8 (s), 53.8 (s), 35.1 (s).

^{19}F NMR (376 MHz, $CDCl_3$) δ -62.64.

IR v_{max} (cm^{-1}): 3047, 1769, 1705, 1383, 1323, 1167, 1120, 1067, 922, 716

HRMS (ESI) m/z: [M + H]+ Calcd for $C_{19}H_{15}F_3NO_2$ 346.1055; Found 346.1046

2-(1-(2-methoxyphenyl)but-3-en-1-yl)isoindoline-1,3-dione (2f)

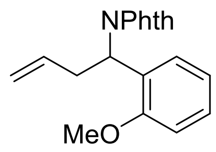

The title compound was prepared according to general procedure **C** from (1,3-dioxoisoindolin-2-yl)(2-methoxyphenyl)methyl acetate (78 mg, 0.240 mmol), $Ca(NTf_2)_2$ (7.2 mg, 0.012 mmol), *n*Bu_4NPF_6 (4.6 mg, 0.012 mmol) and allyl TMS (46 µL, 0.277 mmol) in 1,2- DCE (1.15 mL), reacting for 1 hr then being isolated by FCC (1:12 EtOAc:Hex) as a colourless viscous oil (72 mg, 98%).

RF (1:4 EtOAc:Hex): 0.54

^{1}H NMR (400 MHz, $CDCl_3$) δ 7.83 – 7.74 (m, 2H), 7.71 – 7.62 (m, 3H), 7.33 – 7.20 (m, 1H), 6.97 (td, *J* = 7.6, 0.8 Hz, 1H), 6.84 (d, *J* = 8.2 Hz, 1H), 5.11 (dd, *J* = 17.1, 1.1 Hz, 1H), 4.99 (d, *J* = 10.2 Hz, 1H), 3.78 (s, 3H), 3.39 – 3.25 (m, 1H), 2.93 – 2.81 (m, 1H).

^{13}C{H} NMR (101 MHz, $CDCl_3$) δ 168.3, 156.9, 134.7, 133.7, 131.9, 128.9, 128.9, 126.9, 123.1, 120.2, 118.0, 110.4, 55.5, 48.0, 35.2.

IR v_{max} (cm^{-1}): 2978, 2946, 1763, 1699, 1494, 1466, 1436, 1390, 1358, 1254, 1120, 911, 721

HRMS (ESI) m/z: [M + H]+ Calcd for $C_{19}H_{18}NO_3$ 308.1287; Found 308.1281

2-(1-(2-bromophenyl)but-3-en-1-yl)isoindoline-1,3-dione (2g)

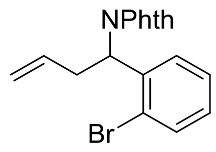

The title compound was prepared according to general procedure **C** from (2-bromophenyl)(1,3-dioxoisoindolin-2-yl)methyl acetate (75 mg, 0.200 mmol), $Ca(NTf_2)_2$ (6.0 mg, 0.010 mmol), *n*Bu_4NPF_6 (3.9 mg, 0.010 mmol) and allyl TMS (38 µL, 0.24 mmol) in 1,2-DCE (1.00 mL), reacting for 5 hr then being isolated by FCC (1:12 EtOAc:Hex) as a colourless viscous oil (69 mg, 97%).

RF (1:4 EtOAc:Hex): 0.56

^{1}H NMR (400 MHz, $CDCl_3$) δ 7.86 – 7.77 (m, 3H), 7.72 – 7.66 (m, 2H), 7.55 (dd, *J* = 8.0, 1.3 Hz, 1H), 7.33 (td, *J* = 7.7, 1.2 Hz, 1H), 7.14 (td, *J* = 7.7, 1.6 Hz, 1H), 5.91 – 5.74 (m, 2H), 5.13 (ddd, *J* = 17.1, 2.7, 1.5 Hz, 1H), 5.02 (d, *J* = 10.1 Hz, 1H), 3.39 – 3.23 (m, 1H), 2.97 – 2.84 (m, 1H).

^{13}C{H} NMR (101 MHz, $CDCl_3$) δ 168.2, 137.9, 134.0, 133.1, 131.7, 130.2, 129.4, 127.4, 124.0, 123.3, 118.6, 53.8, 35.9.

IR v_{max} (cm^{-1}): 2916, 1770, 1705, 1381, 1347, 1079, 1008, 915, 880, 714

HRMS (ESI) m/z: [M + H]+ Calcd for $C_{18}H_{15}BrNO_4$ 356.0281; Found 356.0275

2-(1-(3-chlorophenyl)but-3-en-1-yl)isoindoline-1,3-dione (2h)

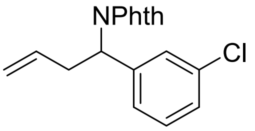

The title compound was prepared according to general procedure **C** from (3-chlorophenyl)(1,3-dioxoisoindolin-2-yl)methyl acetate (75 mg, 0.227 mmol), $Ca(NTf_2)_2$ (6.6 mg, 0.011 mmol), *n*Bu_4NPF_6 (4.3 mg, 0.011 mmol) and allyl TMS (43 µL, 0.272 mmol) in 1,2- DCE (1.10 mL), reacting for 5 hr then being isolated by FCC (1:12 EtOAc:Hex) as a colourless viscous oil (61 mg, 86%).

RF (1:4 EtOAc:Hex): 0.56

^{1}H NMR (400 MHz, $CDCl_3$) δ 7.84 – 7.77 (m, 2H), 7.73 – 7.66 (m, 2H), 7.55 – 7.51 (m, 1H), 7.46 – 7.40 (m, 1H), 7.30 – 7.22 (m, 2H), 5.75 (dddd, *J* = 17.1, 10.2, 8.2, 5.7 Hz, 1H), 5.40 (dd, *J* = 10.6, 5.9 Hz, 1H), 5.14 (ddd, *J* = 17.1, 2.8, 1.6 Hz, 1H), 5.02 (d, *J* = 9.8 Hz, 1H), 3.44 – 3.31 (m, 1H), 3.00 – 2.89 (m, 1H).

^{13}C{H} NMR (101 MHz, $CDCl_3$) δ 168.1, 141.1, 134.4, 134.1, 133.9, 131.7, 129.8, 128.3, 128.1, 126.2, 123.3, 118.6, 53.8, 35.2.

IR v_{max} (cm^{-1}): 3065, 2920, 1770, 1705, 1381, 1349, 1071, 915, 714

HRMS (ESI) m/z: [M + H]+ Calcd for $C_{18}H_{14}BrFNO_2$ 312.0786; Found 312.0782

2-(1-(3-bromo-2-fluorophenyl)but-3-en-1-yl)isoindoline-1,3-dione (2i)

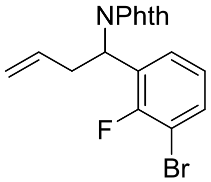

The title compound was prepared according to general procedure **C** from (3-bromo-2-fluorophenyl)(1,3-dioxoisoindolin-2-yl)methyl acetate (75 mg, 0.191 mmol), $Ca(NTf_2)_2$ (5.7 mg, 0.010 mmol), *n*Bu_4NPF_6 (3.7 mg, 0.010 mmol) and allyl TMS (36 µL, 0.23 mmol) in 1,2- DCE (0.96 mL), reacting overnight then being isolated by FCC (1:9 EtOAc:Hex) as a white solid (51 mg, 71%).

RF (1:4 EtOAc:Hex): 0.61

^{1}H NMR (400 MHz, $CDCl_3$) δ 7.84 – 7.78 (m, 2H), 7.74 – 7.65 (m, 3H), 7.52 – 7.44 (m, 1H), 7.05 (t, *J* = 7.9 Hz, 1H), 5.84 – 5.71 (m, 2H), 5.14 (dd, *J* = 17.1, 1.0 Hz, 1H), 5.04 (d, *J* = 10.1 Hz, 1H), 3.39 – 3.24 (m, 1H), 2.97 – 2.85 (m, 1H).

$^{13}C\{H\}$ NMR (101 MHz, $CDCl_3$) δ 167.9 (s), 156.8 (d, *J* = 248.7 Hz), 134.1 (s), 133.5 (s), 133.1 (s), 131.6 (s), 128.8 (d, *J* = 2.9 Hz), 127.5 (d, *J* = 14.7 Hz), 124.9 (d, *J* = 4.5 Hz), 123.4 (s), 118.8 (d, *J* = 21.0 Hz), 109.3 (d, *J* = 21.7 Hz), 47.2 (d, *J* = 2.6 Hz), 35.0 (s).

^{19}F NMR (376 MHz, $CDCl_3$) δ -109.65.

IR v_{max} (cm^{-1}): 3082, 2924, 1770, 1709, 1450, 1385, 1347, 1064, 928, 714

HRMS (ESI) m/z: [M + H]+ Calcd for $C_{18}H_{14}BrFNO_2$ 374.0186; Found 374.0180

2-(1-(3,5-bis(trifluoromethyl)phenyl)but-3-en-1-yl)isoindoline-1,3-dione (2j)

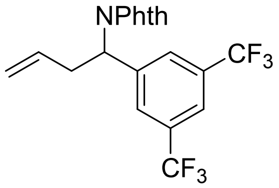

The title compound was prepared according to general procedure **C** from (3,5-bis(trifluoromethyl)phenyl)(1,3-dioxoisoindolin-2-yl)methyl acetate (100 mg, 0.232 mmol), $Ca(NTf_2)_2$ (7.0 mg, 0.012 mmol), *n*Bu_4NPF_6 (4.5 mg, 0.012 mmol) and allyl TMS (44 µL, 0.278 mmol) in 1,2- DCE (1.16 mL), reacting overnight then being isolated by FCC (1:12 EtOAc:Hex) as a colourless viscous oil (22 mg, 23%).

RF (1:4 EtOAc:Hex): 0.61

^1H NMR (400 MHz, $CDCl_3$) δ 8.01 (s, 2H), 7.87 – 7.79 (m, 3H), 7.77 – 7.70 (m, 2H), 5.81 – 5.68 (m, 1H), 5.53 (dd, *J* = 10.4, 6.0 Hz, 1H), 5.17 (d, *J* = 17.0 Hz, 1H), 5.06 (d, *J* = 10.2 Hz, 1H), 3.47 – 3.36 (m, 1H), 3.05 – 2.94 (m, 1H).

$^{13}C\{H\}$ NMR (101 MHz, $CDCl_3$) δ 168.0 (s), 141.5 (s), 134.3 (s), 133.1 (s), 131.9 (q, *J* = 33.4 Hz), 131.5 (s), 128.6 (d, *J* = 2.6 Hz), 123.6 (s), 127.7 – 118.6 (m), 122.2 – 121.9 (m), 119.3 (s), 53.3 (s), 35.2 (s).

^{19}F NMR (376 MHz, $CDCl_3$) δ -62.80.

IR v_{max} (cm^{-1}): 3080, 1765, 1703, 1381, 1351, 1274, 1128, 924, 893, 721, 699

HRMS (ESI) m/z: [M + H]+ Calcd for $C_{20}H_{14}F_6NO_2$ 414.0915; Found 414.0923

2-(1-(4-nitrophenyl)but-3-en-1-yl)isoindoline-1,3-dione (2k)

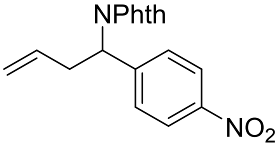

The title compound was prepared according to general procedure **C** from (1,3-dioxoisoindolin-2-yl)(4-nitrophenyl)methyl acetate (75 mg, 0.22 mmol), $Ca(NTf_2)_2$ (6.6 mg, 0.011 mmol), *n*Bu_4NPF_6 (4.3 mg, 0.011 mmol) and allyl TMS (30 µL, 0.264 mmol) in 1,2- DCE (1.10 mL),

reacting overnight then being isolated by FCC (1:6 EtOAc:Hex) as a colourless viscous oil (31 mg, 44%).

RF (1:4 EtOAc:Hex): 0.56

^{1}H NMR (400 MHz, $CDCl_3$) δ 8.19 (d, *J* = 8.8 Hz, 2H), 7.88 – 7.77 (m, 2H), 7.78 – 7.66 (m, 4H), 5.83 – 5.69 (m, 1H), 5.52 (dd, *J* = 10.4, 6.1 Hz, 1H), 5.16 (dd, *J* = 17.1, 0.9 Hz, 1H), 5.05 (d, *J* = 10.1 Hz, 1H), 3.45 – 3.32 (m, 1H), 3.07 – 2.95 (m, 1H).

^{13}C{H} NMR (101 MHz, $CDCl_3$) δ 168.1, 147.5, 146.1, 134.3, 133.3, 131.5, 129.1, 123.8, 123.5, 119.1, 53.5, 35.1.

IR v_{max} (cm^{-1}): 3460, 3080, 1770, 1705, 1599, 1509, 1382, 1345, 1077, 993, 851, 721

HRMS (ESI) m/z: [M + H]+ Calcd for $C_{18}H_{15}N_2O_4$ 323.1026; Found 323.1022

2-(1-(naphthalen-2-yl)but-3-en-1-yl)isoindoline-1,3-dione (2l)

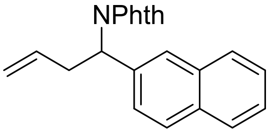

The title compound was prepared according to general procedure **C** from (1,3-dioxoisoindolin-2-yl)(naphthalen-2-yl)methyl acetate (100 mg, 0.290 mmol), $Ca(NTf_2)_2$ (9.0 mg, 0.015 mmol), *n*Bu_4NPF_6 (5.8 mg, 0.015 mmol) and allyl TMS (55 µL, 0.35 mmol) in 1,2- DCE (1.50 mL), reacting for 1 hr then being isolated by FCC (1:12 EtOAc:Hex) as a colourless viscous oil (66 mg, 70%).

RF (1:4 EtOAc:Hex): 0.60

^{1}H NMR (400 MHz, $CDCl_3$) δ 8.01 (s, 1H), 7.88 – 7.77 (m, 5H), 7.73 – 7.62 (m, 3H), 7.50 – 7.42 (m, 2H), 5.84 (dddd, *J* = 17.1, 10.2, 8.2, 5.7 Hz, 1H), 5.63 (dd, *J* = 10.5, 6.0 Hz, 1H), 5.20 (dd, *J* = 17.1, 1.2 Hz, 1H), 5.05 (d, *J* = 10.1 Hz, 1H), 3.60 – 3.46 (m, 1H), 3.19 – 3.05 (m, 1H).

^{13}C{H} NMR (101 MHz, $CDCl_3$) δ 168.6, 136.8, 134.6, 134.1, 133.4, 133.1, 132.0, 128.6, 128.4, 127.8, 127.3, 126.4, 126.4, 126.2, 123.4, 118.5, 54.8, 35.6.

IR v_{max} (cm^{-1}): 3047, 2955, 2909, 1768, 1701, 1382, 1351, 1328, 1077, 923, 713

HRMS (ESI) m/z: [M + H]+ Calcd for $C_{22}H_{18}NO_2$ 328.1338; Found 328.1328

2-(1-(furan-2-yl)but-3-en-1-yl)isoindoline-1,3-dione (2m)

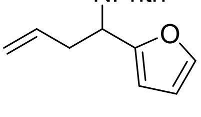

The title compound was prepared according to general procedure **C** from (1,3-dioxoisoindolin-2-yl)(furan-2-yl)methyl acetate (75 mg, 0.263 mmol), $Ca(NTf_2)_2$ (7.8 mg, 0.013 mmol), *n*Bu_4NPF_6 (5.0 mg, 0.013 mmol) and allyl TMS (50 µL, 0.32 mmol) in 1,2- DCE (1.30 mL),

reacting for 1 hr then being isolated by FCC (1:9 EtOAc:Hex) as a colourless viscous oil (34 mg, 48%).

RF (1:4 EtOAc:Hex): 0.60

^{1}H NMR (400 MHz, $CDCl_3$) δ 7.85 – 7.79 (m, 2H), 7.73 – 7.67 (m, 2H), 7.32 (d, *J* = 1.1 Hz, 1H), 6.40 (d, *J* = 3.3 Hz, 1H), 6.33 (dd, *J* = 3.3, 1.8 Hz, 1H), 5.77 (dddd, *J* = 17.0, 10.2, 8.3, 5.7 Hz, 1H), 5.50 (dd, *J* = 10.3, 5.8 Hz, 1H), 5.14 (ddd, *J* = 17.1, 2.8, 1.6 Hz, 1H), 5.03 (d, *J* = 10.0 Hz, 1H), 3.28 – 3.17 (m, 1H), 3.03 – 2.93 (m, 1H).

^{13}C{H} NMR (101 MHz, $CDCl_3$) δ 167.8, 151.9, 141.9, 134.0, 133.5, 131.8, 123.3, 118.7, 110.4, 107.7, 47.7, 34.4.

IR v_{max} (cm^{-1}): 3118, 2924, 1772, 1707, 1377, 1355, 1135, 1084, 1008, 934, 876, 713

HRMS (ESI) m/z: [M + H]+ Calcd for $C_{16}H_{14}NO_3$ 268.0968; Found 268.0962

2-(1-(thiophen-2-yl)but-3-en-1-yl)isoindoline-1,3-dione (2n)

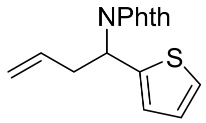

The title compound was prepared according to general procedure **C** from (1,3-dioxoisoindolin-2-yl)(thiophen-2-yl)methyl acetate (75 mg, 0.249 mmol), $Ca(NTf_2)_2$ (7.5 mg, 0.013 mmol), *n*Bu_4NPF_6 (4.8 mg, 0.013 mmol) and allyl TMS (47 µL, 0.30 mmol) in 1,2- DCE (1.2 mL), reacting for 1 hr then being isolated by FCC (1:9 EtOAc:Hex) as a colourless viscous oil (65 mg, 92%).

RF (1:4 EtOAc:Hex): 0.59

^{1}H NMR (400 MHz, $CDCl_3$) δ 7.86 – 7.77 (m, 2H), 7.73 – 7.65 (m, 2H), 7.22 (dd, *J* = 5.1, 1.0 Hz, 1H), 7.16 (d, *J* = 3.5 Hz, 1H), 6.94 (dd, *J* = 5.1, 3.6 Hz, 1H), 5.81 – 5.71 (m, 1H), 5.68 (dd, *J* = 10.4, 6.1 Hz, 1H), 5.14 (dd, *J* = 17.1, 1.2 Hz, 1H), 5.02 (d, *J* = 10.1 Hz, 1H), 3.46 – 3.31 (m, 1H), 3.09 – 2.93 (m, 1H).

^{13}C{H} NMR (101 MHz, $CDCl_3$) δ 167.8, 142.0, 134.0, 133.8, 131.7, 126.6, 126.3, 125.3, 123.3, 118.6, 49.6, 37.1.

IR v_{max} (cm^{-1}): 3076, 1768, 1701, 1466, 1371, 1347, 1325, 1232, 1067, 916, 708

HRMS (ESI) m/z: [M + H]+ Calcd for $C_{16}H_{14}NO_2S$ 284.0740; Found 284.0735

2-(1-cyclohexylbut-3-en-1-yl)isoindoline-1,3-dione (2o)

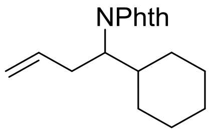

The title compound was prepared according to general procedure **C** from cyclohexyl(1,3-dioxoisoindolin-2-yl)methyl acetate (75 mg, 0.250 mmol), $Ca(NTf_2)_2$ (7.5 mg, 0.013 mmol), *n*Bu_4NPF_6 (4.8 mg, 0.013 mmol) and allyl TMS (47 µL, 0.30 mmol) in 1,2- DCE (1.2 mL), reacting overnight then being isolated by FCC (1:15 EtOAc:Hex) as a colourless viscous oil (68 mg, 96%).

RF (1:4 EtOAc:Hex): 0.72

^{1}H NMR (400 MHz, $CDCl_3$) δ 7.85 – 7.77 (m, 2H), 7.74 – 7.66 (m, 2H), 5.65 (dtd, *J* = 16.9, 9.6, 5.2 Hz, 1H), 4.98 (d, *J* = 17.0 Hz, 1H), 4.88 (d, *J* = 10.1 Hz, 1H), 4.07 – 3.94 (m, 1H), 2.90 – 2.74 (m, 1H), 2.66 – 2.52 (m, 1H), 2.16 – 2.03 (m, 1H), 1.99 – 1.89 (m, 1H), 1.82 – 1.72 (m, 1H), 1.70 – 1.60 (m, 2H), 1.59 – 1.51 (m, 1H), 1.36 – 0.86 (m, 5H).

^{13}C{H} NMR (101 MHz, $CDCl_3$) δ 168.8, 135.1, 133.8, 131.7, 123.1, 117.6, 56.9, 39.2, 33.8, 30.7, 30.3, 26.2, 25.8, 25.7.

*Data in accordance with literature.[3]

Other α-Functionalised Phth-N amines

2-(phenyl(phenylthio)methyl)isoindoline-1,3-dione (3a)

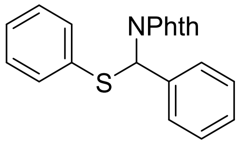

The title compound was prepared according to general procedure **C** from (1,3-dioxoisoindolin-2-yl)(phenyl)methyl acetate (50 mg, 0.169 mmol), $Ca(NTf_2)_2$ (5.1 mg, 0.009 mmol), *n*Bu_4NPF_6 (3.3 mg, 0.009 mmol) and thiophenol (21 µL, 0.203 mmol) in 1,2- DCE (0.85 mL), reacting for 15 mins then being isolated by FCC (1:6 EtOAc:Hex) as a colourless viscous oil (58 mg, 99%).

RF (1:4 EtOAc:Hex): 0.46

^{1}H NMR (400 MHz, $CDCl_3$) δ 7.80 – 7.74 (m, 2H), 7.73 – 7.64 (m, 4H), 7.50 – 7.43 (m, 2H), 7.39 – 7.28 (m, 3H), 7.24 – 7.19 (m, 3H), 6.74 (s, 1H).

^{13}C{H} NMR (101 MHz, $CDCl_3$) δ 166.8, 136.8, 134.2, 133.5, 133.1, 131.5, 129.2, 128.6, 128.3, 128.1, 123.5, 61.0.

IR v_{max} (cm^{-1}): 3058, 1764, 1712, 1375, 1343, 1310, 1068, 889, 695

HRMS (ESI) m/z: [M-PhS]+ Calcd for $C_{15}H_{10}N_2O_4$ 236.0706; Found 236.0711

2-((3-bromo-2-fluorophenyl)(phenylthio)methyl)isoindoline-1,3-dione (3b)

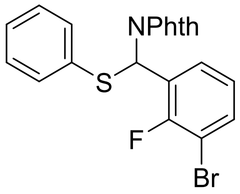

The title compound was prepared according to general procedure **C** from (3-bromo-2-fluorophenyl)(1,3-dioxoisoindolin-2-yl)methyl acetate (75 mg, 0.191 mmol), $Ca(NTf_2)_2$ (5.7 mg, 0.010 mmol), $n$$Bu_4NPF_6$ (3.7 mg, 0.010 mmol) and thiophenol (23 µL, 0.229 mmol) in 1,2-DCE (0.96 mL), reacting for 15 mins then being isolated by FCC (1:6 EtOAc:Hex) as a colourless viscous oil (79 mg, 94%).

RF (1:4 EtOAc:Hex): 0.63

^{1}H NMR (400 MHz, $CDCl_3$) δ 8.17 – 8.11 (m, 1H), 7.81 – 7.75 (m, 2H), 7.72 – 7.66 (m, 2H), 7.56 – 7.46 (m, 3H), 7.27 – 7.22 (m, 3H), 7.11 (td, J = 8.0, 1.0 Hz, 1H), 6.96 (s, 1H).

^{13}C{H} NMR (101 MHz, $CDCl_3$) δ 166.3 (s), 155.9 (d, J = 249.3 Hz), 134.3 (s), 134.0 (s), 133.7 (s), 132.3 (s), 131.4 (s), 130.4 (d, J = 1.8 Hz), 129.3 (s), 128.8 (s), 125.5 (d, J = 14.1 Hz), 125.0 (d, J = 4.6 Hz), 123.6 (s), 109.1 (d, J = 21.4 Hz), 54.2 (d, J = 3.2 Hz).

^{19}F NMR (376 MHz, $CDCl_3$) δ -108.95 (t, J = 6.7 Hz).

IR v_{max} (cm^{-1}): 3058, 1763, 1716, 1449, 1373, 1321, 1224, 1067, 889, 713

HRMS (ESI) m/z: [M-SPh]+ Calcd for $C_{15}H_8NO_2FBr$ 331.9717; 331.9714

2-((4-nitrophenyl)(phenylthio)methyl)isoindoline-1,3-dione (3c)

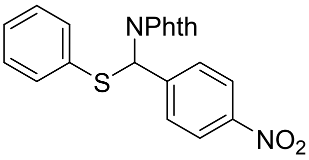

The title compound was prepared according to general procedure **C** from (1,3-dioxoisoindolin-2-yl)(4-nitrophenyl)methyl acetate (75 mg, 0.220 mmol), $Ca(NTf_2)_2$ (6.6 mg, 0.011 mmol), $n$$Bu_4NPF_6$ (4.3 mg, 0.011 mmol) and thiophenol (27 µL, 0.264 mmol) in 1,2- DCE (1.10 mL), reacting for 15 mins then being isolated by FCC (1:4 EtOAc:Hex) as an off-white solid obtained (79 mg, 92%).

RF (1:3 EtOAc:Hex): 0.23

^{1}H NMR (400 MHz, $CDCl_3$) δ 8.25 – 8.18 (m, 2H), 7.90 – 7.84 (m, 2H), 7.85 – 7.79 (m, 2H), 7.78 – 7.70 (m, 2H), 7.51 – 7.42 (m, 2H), 7.31 – 7.20 (m, 3H), 6.75 (s, 1H).

^{13}C{H} NMR (101 MHz, $CDCl_3$) δ 166.6, 147.8, 143.8, 134.6, 133.5, 132.5, 131.3, 129.5, 129.2, 128.9, 123.8, 123.8, 60.3.

IR v_{max} (cm^{-1}): 3106, 3080, 1772, 1714, 1597, 1515, 1343, 1319, 1079, 892, 713

HRMS (ESI) m/z: [M - H]- Calcd for $C_{21}H_{13}N_2O_4S$ 389.0602; Found 389.0592

2-(((2-fluorophenyl)thio)(thiophen-2-yl)methyl)isoindoline-1,3-dione (3d)

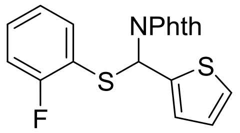

The title compound was prepared according to general procedure **C** from (1,3-dioxoisoindolin-2-yl)(thiophen-2-yl)methyl acetate (75 mg, 0.249 mmol), $Ca(NTf_2)_2$ (7.5 mg, 0.012 mmol), *n*Bu_4NPF_6 (4.8 mg, 0.012 mmol) and 2-fluorothiophenol (32 μL, 0.299 mmol) in 1,2- DCE (1.25 mL), reacting for 15 mins then being isolated by FCC (1:6 EtOAc:Hex) as a white solid obtained (59 mg, 64%).

RF (1:4 EtOAc:Hex): 0.43

^{1}H NMR (400 MHz, $CDCl_3$) δ 7.84 – 7.78 (m, 2H), 7.73 – 7.68 (m, 2H), 7.46 (td, *J* = 7.5, 1.7 Hz, 1H), 7.32 – 7.26 (m, 3H), 7.08 – 7.01 (m, 1H), 7.00 – 6.94 (m, 2H), 6.90 (d, *J* = 0.6 Hz, 1H).

^{13}C{H} NMR (101 MHz, $CDCl_3$) δ 166.3 (s), 162.8 (d, *J* = 248.0 Hz), 139.1 (s), 136.5 (s), 134.3 (s), 131.6 (d, *J* = 8.1 Hz), 131.5 (s), 127.7 (s), 126.8 (s), 126.63 (s), 124.7 (d, *J* = 3.9 Hz), 123.6 (s), 119.8 (d, *J* = 18.4 Hz), 116.1 (d, *J* = 23.0 Hz), 55.4 (d, *J* = 2.3 Hz).

^{19}F NMR (376 MHz, $CDCl_3$) δ -106.62 (ddd, *J* = 9.1, 7.3, 5.1 Hz).

IR v_{max} (cm^{-1}): 3067, 2924, 1764, 1714, 1466, 1353, 1325, 1218, 1081, 885, 762, 702

HRMS (ESI) m/z: [M-S(C_6H_5F)]+ Calcd for $C_{13}H_8NO_2S$ 242.0270; Found 242.0270

2-(cyclopropyl((4-methoxyphenyl)thio)methyl)isoindoline-1,3-dione (3e)

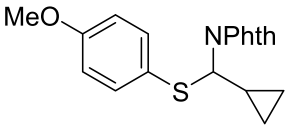

The title compound was prepared according to general procedure **C** from cyclopropyl(1,3-dioxoisoindolin-2-yl)methyl acetate (75 mg, 0.289 mmol), $Ca(NTf_2)_2$ (8.7 mg, 0.015 mmol), *n*Bu_4NPF_6 (5.6 mg, 0.015 mmol) and 4-methoxythiophenol (43 μL, 0.347 mmol) in 1,2- DCE (1.45 mL), reacting for 15 mins then being isolated by FCC (1:8 EtOAc:Hex) as a pale yellow solid obtained (85 mg, 87%).

RF (1:4 EtOAc:Hex): 0.47

^{1}H NMR (400 MHz, $CDCl_3$) δ 7.86 – 7.74 (m, 2H), 7.73 – 7.66 (m, 2H), 7.36 – 7.31 (m, 2H), 6.75 – 6.65 (m, 2H), 4.61 (d, *J* = 10.5 Hz, 1H), 3.71 (s, 3H), 2.11 – 1.99 (m, 1H), 0.90 – 0.78 (m, 1H), 0.64 – 0.52 (m, 2H), 0.39 – 0.30 (m, 1H).

$^{13}C\{H\}$ NMR (101 MHz, $CDCl_3$) δ 167.0, 160.1, 136.6, 134.0, 131.6, 131.6, 123.3, 114.5, 64.5, 55.2, 14.2, 6.6, 5.7.

IR v_{max} (cm^{-1}): 2996, 2927, 2834, 1764, 1712, 1589, 1494, 1371, 1246, 1079, 1030, 837, 710

HRMS (ESI) m/z: [M-S($C_6H_5OCH_3$)]+ Calcd for $C_{12}H_{10}NO_2$ 200.0706; Found 200.0706

2-((ethylthio)(4-methoxyphenyl)methyl)isoindoline-1,3-dione (3f)

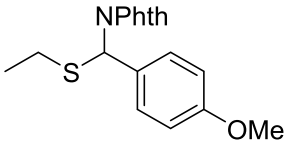

The title compound was prepared according to general procedure **C** from (1,3-dioxoisoindolin-2-yl)(4-methoxyphenyl)methyl acetate (50 mg, 0.154 mmol), $Ca(NTf_2)_2$ (4.6 mg, 0.008 mmol), *n*Bu_4NPF_6 (3.0 mg, 0.008 mmol) and ethanethiol (13 µL, 0.185 mmol) in 1,2- DCE (0.77 mL), reacting for 15 mins then being isolated by FCC (1:6 EtOAc:Hex) as a colourless oil obtained (41 mg, 81%).

RF (1:4 EtOAc:Hex): 0.45

^{1}H NMR (400 MHz, $CDCl_3$) δ 7.86 – 7.81 (m, 2H), 7.74 – 7.68 (m, 2H), 7.61 – 7.55 (m, 2H), 6.89 – 6.83 (m, 2H), 6.46 (s, 1H), 3.78 (s, 3H), 2.73 – 2.54 (m, 2H), 1.30 (t, *J* = 7.4 Hz, 3H).

$^{13}C\{H\}$ NMR (101 MHz, $CDCl_3$) δ 167.1, 159.5, 134.2, 131.7, 129.4, 129.3, 123.5, 113.7, 56.6, 55.3, 26.6, 14.5.

IR v_{max} (cm^{-1}): 2929, 2840, 1754, 1709, 1608, 1511, 1373, 1312, 1250, 1189, 1098, 1021, 891, 829, 710

HRMS (ESI) m/z: [M-SEt]+ Calcd for $C_{16}H_{12}NO_3$ 266.0812; Found 266.0806

2-((5-bromo-1-methyl-1H-indol-3-yl)(phenyl)methyl)isoindoline-1,3-dione (3g)

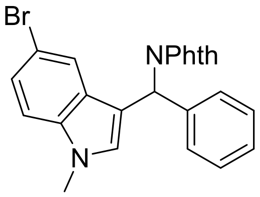

The title compound was prepared according to general procedure **C** from (1,3-dioxoisoindolin-2-yl)(phenyl)methyl acetate (50 mg, 0.169 mmol), $Ca(NTf_2)_2$ (5.1 mg, 0.008 mmol), *n*Bu_4NPF_6 (3.3 mg, 0.008 mmol) and 5-bromo-*N*-methylindole (43 mg, 0.203 mmol) in 1,2- DCE (0.85 mL), reacting for 15 mins then being isolated by FCC (1:8 to 1:6 EtOAc:Hex) as an off white solid obtained (71 mg, 94%).

RF (1:4 EtOAc:Hex): 0.31

^{1}H NMR (400 MHz, $CDCl_3$) δ 7.83 – 7.78 (m, 2H), 7.71 – 7.66 (m, 2H), 7.64 (d, *J* = 1.7 Hz, 1H), 7.50 – 7.45 (m, 2H), 7.36 – 7.25 (m, 4H), 7.17 – 7.10 (m, 2H), 6.95 (s, 1H), 3.72 (s, 3H).

$^{13}C\{H\}$ NMR (101 MHz, $CDCl_3$) δ 166.9, 137.6, 134.3, 133.0, 130.8, 130.2, 127.8, 127.4, 126.8, 126.6, 123.7, 122.4, 120.3, 112.0, 110.5, 109.9, 48.9, 32.1.

IR v_{max} (cm^{-1}): 3058, 3030, 2920, 165, 1701, 1474, 1382, 1353, 1321, 1070, 894, 792, 714

HRMS (ESI) m/z: [M-C_9H_7NBr]+ Calcd for $C_{15}H_{10}NO_2$ 236.0706 Found 236.0703

2-((5-bromo-1-methyl-1H-indol-3-yl)(2-methoxyphenyl)methyl)isoindoline-1,3-dione (3h)

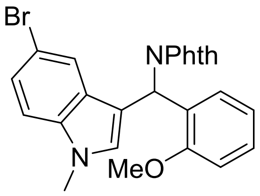

The title compound was prepared according to general procedure **C** from (1,3-dioxoisoindolin-2-yl)(2-methoxyphenyl)methyl acetate (75 mg, 0.231 mmol), $Ca(NTf_2)_2$ (6.9 mg, 0.012 mmol), *n*Bu_4NPF_6 (4.5 mg, 0.012 mmol) and 5-bromo-*N*-methylindole (58 mg, 0.277 mmol) in 1,2-DCE (1.16 mL), reacting for 15 mins then being isolated by FCC (1:4 EtOAc:Hex) as an off white solid obtained (90 mg, 82%).

RF (1:4 EtOAc:Hex): 0.24

^{1}H NMR (400 MHz, $CDCl_3$) δ 7.83 – 7.76 (m, 2H), 7.70 – 7.63 (m, 2H), 7.58 (d, *J* = 1.7 Hz, 1H), 7.47 (dd, *J* = 7.6, 1.4 Hz, 1H), 7.30 – 7.24 (m, 2H), 7.21 (s, 1H), 7.13 (d, *J* = 8.7 Hz, 1H), 7.09 (s, 1H), 6.88 (td, *J* = 8.3, 2.0 Hz, 2H), 3.76 (s, 3H), 3.70 (s, 3H).

$^{13}C\{H\}$ NMR (101 MHz, $CDCl_3$) δ 168.1, 156.9, 135.6, 133.9, 132.0, 130.4, 130.3, 129.2, 128.6, 126.4, 124.7, 123.2, 121.6, 120.2, 112.9, 111.6, 110.9, 110.4, 55.7, 45.1, 33.0.

IR v_{max} (cm^{-1}): 3052, 2961, 2920, 1772, 1705, 1476, 1354, 1239, 1107, 869, 874, 756, 728, 712

HRMS (ESI) m/z: [M-C_9H_7NBr]+ Calcd for $C_{16}H_{12}NO_3$ 266.0812; Found 266.0811

2-((5-bromo-1-methyl-1H-indol-3-yl)(furan-2-yl)methyl)isoindoline-1,3-dione (3i)

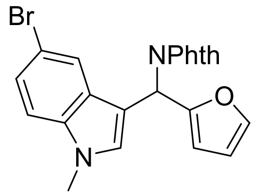

The title compound was prepared according to general procedure **C** from (1,3-dioxoisoindolin-2-yl)(furan-2-yl)methyl acetate (75 mg, 0.263 mmol), $Ca(NTf_2)_2$ (7.9 mg, 0.013 mmol), $n$$Bu_4NPF_6$ (5.1 mg, 0.013 mmol) and 5-bromo-*N*-methylindole (66 mg, 0.316 mmol) in 1,2-DCE (1.32 mL), reacting for 15 mins then being isolated by FCC (1:6 to 1:4 EtOAc:Hex) as a brown solid. (82 mg, 72%).

RF (1:4 EtOAc:Hex): 0.29

^{1}H NMR (400 MHz, $CDCl_3$) δ 7.83 (t, *J* = 3.5 Hz, 1H), 7.78 (td, *J* = 5.3, 2.1 Hz, 2H), 7.69 – 7.62 (m, 2H), 7.43 (s, 1H), 7.37 (t, *J* = 1.1 Hz, 1H), 7.27 (dd, *J* = 8.7, 1.8 Hz, 1H), 7.14 (d, *J* = 8.7 Hz, 1H), 6.92 (s, 1H), 6.35 (t, *J* = 2.3 Hz, 2H), 3.74 (s, 3H).

^{13}C{H} NMR (101 MHz, $CDCl_3$) δ 167.5, 151.2, 142.2, 135.2, 134.0, 131.9, 131.4, 128.4, 124.9, 123.4, 121.5, 113.3, 111.0, 110.6, 109.4, 108.9, 43.7, 33.2.

IR v_{max} (cm^{-1}): 3119, 2920, 1763, 1705, 1476, 1343, 1317, 1142, 1107, 1008, 790, 728

HRMS (ESI) m/z: [M-C_9H_7NBr]+ Calcd for $C_{13}H_8NO_3$ 226.0499; Found 226.0495

2-((3,5-bis(trifluoromethyl)phenyl)(5-bromo-1-methyl-1H-indol-3-yl)methyl) isoindoline-1,3-dione (3j)

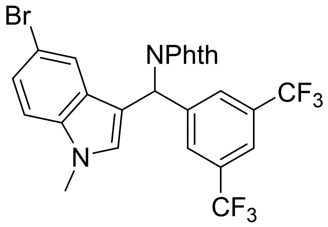

The title compound was prepared according to general procedure **C** from (3,5-bis(trifluoromethyl)phenyl)(1,3-dioxoisoindolin-2-yl)methyl acetate (75 mg, 0.174 mmol), $Ca(NTf_2)_2$ (5.2 mg, 0.009 mmol), $n$$Bu_4NPF_6$ (3.4 mg, 0.009 mmol) and 5-bromo-*N*-methylindole (44 mg, 0.209 mmol) in 1,2- DCE (0.87 mL), reacting for 15 mins then being isolated by FCC (1:6 EtOAc:Hex) as a white solid. (94 mg, 93%).

RF (1:4 EtOAc:Hex): 0.45

^{1}H NMR (400 MHz, $CDCl_3$) δ 7.93 (s, 2H), 7.88 – 7.81 (m, 3H), 7.76 – 7.70 (m, 2H), 7.67 (d, *J* = 1.6 Hz, 1H), 7.32 (dd, *J* = 8.7, 1.8 Hz, 1H), 7.19 (d, *J* = 8.7 Hz, 1H), 7.15 (s, 1H), 7.04 (s, 1H), 3.77 (s, 3H).

$^{13}C\{H\}$ NMR (101 MHz, $CDCl_3$) δ 167.7 (s), 141.5 (s), 135.4 (s), 134.5 (s), 132.4 – 131.3 (m), 131.6 (s), 131.1 (s), 128.4 (s), 128.1 (d, *J* = 2.7 Hz), 125.3 (s), 123.7 (s), 123.2 (q, *J* = 272.8 Hz), 121.9 – 121.7 (m), 121.1 (s), 113.5 (s), 111.2 (s), 110.0 (s), 49.1 (s), 33.3 (s).

^{19}F NMR (376 MHz, $CDCl_3$) δ -62.67.

IR v_{max} (cm^{-1}): 3062, 2922, 1774, 1705, 1477, 1354, 1280, 1164, 1108, 921, 794, 712

HRMS (ESI) m/z: [M + H]+ Calcd for $C_{26}H_{16}F_6N_2O_2$ 581.0294; Found 581.0283

2-((5-bromo-1-methyl-1H-indol-3-yl)(cyclohexyl)methyl)isoindoline-1,3-dione (3k)

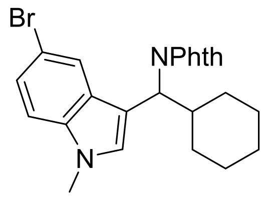

The title compound was prepared according to general procedure **C** from cyclohexyl(1,3-dioxoisoindolin-2-yl)methyl acetate (75 mg, 0.242 mmol), $Ca(NTf_2)_2$ (7.2 mg, 0.012 mmol), *n*Bu_4NPF_6 (4.6 mg, 0.012 mmol) and 5-bromo-*N*-methylindole (61 mg, 0.290 mmol) in 1,2-DCE (1.21 mL), reacting for 15 mins then being isolated by FCC (1:6 EtOAc:Hex) as a white solid. (97 mg, 89%).

RF (1:4 EtOAc:Hex): 0.47

^{1}H NMR (400 MHz, $CDCl_3$) δ 7.93 (d, *J* = 1.7 Hz, 1H), 7.78 – 7.72 (m, 2H), 7.65 – 7.59 (m, 2H), 7.40 (s, 1H), 7.25 (dd, *J* = 8.6, 1.8 Hz, 1H), 7.10 (d, *J* = 8.6 Hz, 1H), 5.26 (d, *J* = 11.4 Hz, 1H), 3.74 (s, 3H), 2.76 (qt, *J* = 11.2, 3.2 Hz, 1H), 1.77 – 1.61 (m, 5H), 1.36 – 1.14 (m, 3H), 1.13 – 1.00 (m, 1H), 0.98 – 0.85 (m, 1H).

$^{13}C\{H\}$ NMR (101 MHz, $CDCl_3$) δ 168.4, 134.9, 133.8, 131.9, 130.2, 129.6, 124.6, 123.1, 121.6, 113.0, 111.8, 110.7, 51.7, 38.5, 33.1, 31.3, 30.5, 26.3, 25.76, 25.67.

IR v_{max} (cm^{-1}): 2926, 2845, 1761, 1697, 1474, 1377, 1326, 1071, 793, 731

HRMS (ESI) m/z: [M + H]+ Calcd for $C_{24}H_{24}N_2O_2Br$ 451.1016; Found 451.1007

N-((1,3-dioxoisoindolin-2-yl)(phenyl)methyl)benzamide (3l)

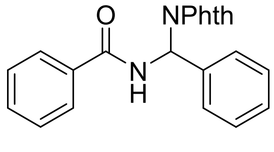

The title compound was prepared according to general procedure **C** from (1,3-dioxoisoindolin-2-yl)(phenyl)methyl acetate (75 mg, 0.254 mmol), $Ca(NTf_2)_2$ (7.6 mg, 0.013 mmol), *n*Bu_4NPF_6 (4.9 mg, 0.013 mmol) and benzamide (37 mg, 0.305 mmol) in 1,2- DCE (1.27 mL), reacting for 1 hr then being isolated by FCC (1:3 EtOAc:Hex) as a white solid. (52 mg, 57%).

RF (1:1 EtOAc:Hex): 0.71

^{1}H NMR (400 MHz, $CDCl_3$) δ 7.93 (d, *J* = 9.6 Hz, 1H), 7.89 – 7.83 (m, 4H), 7.76 – 7.70 (m, 2H), 7.62 (d, *J* = 9.6 Hz, 1H), 7.55 – 7.47 (m, 3H), 7.47 – 7.42 (m, 2H), 7.41 – 7.30 (m, 3H).

^{13}C{H} NMR (101 MHz, $CDCl_3$) δ 167.7, 166.3, 137.1, 134.4, 133.3, 132.2, 131.7, 129.0, 128.7, 127.3, 126.1, 123.7, 58.8.

IR v_{max} (cm^{-1}): 3369, 3050, 1776, 1710, 1664, 1511, 1312, 1258, 1123, 878, 718

HRMS (ESI) m/z: : [M + H]+ Calcd for $C_{22}H_{17}N_2O_3$ 357.1234; Found 357.1236

N-((3-chlorophenyl)(1,3-dioxoisoindolin-2-yl)methyl)-4-methoxybenzamide (3m)

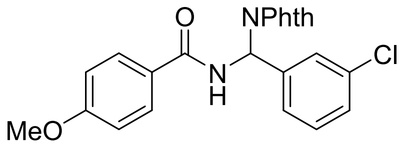

The title compound was prepared according to general procedure **C** from (3-chlorophenyl)(1,3-dioxoisoindolin-2-yl)methyl acetate (75 mg, 0.227 mmol), $Ca(NTf_2)_2$ (6.8 mg, 0.011 mmol), *n*Bu_4NPF_6 (4.4 mg, 0.011 mmol) and 4-methoxybenzamide (41 mg, 0.272 mmol) in 1,2- DCE (1.14 mL), reacting for 1 hr then being isolated by FCC (1:3 EtOAc:Hex) as a white solid. (34 mg, 36%).

RF (1:3 EtOAc:Hex): 0.20

^{1}H NMR (400 MHz, $CDCl_3$) δ 7.90 – 7.86 (m, 2H), 7.86 – 7.80 (m, 3H), 7.78 – 7.72 (m, 2H), 7.59 (d, *J* = 9.7 Hz, 1H), 7.49 – 7.46 (m, 1H), 7.38 – 7.33 (m, 1H), 7.33 – 7.29 (m, 2H), 6.97 – 6.91 (m, 2H), 3.85 (s, 3H).

^{13}C{H} NMR (101 MHz, $CDCl_3$) δ 167.6, 165.8, 162.9, 139.4, 135.0, 134.5, 131.6, 130.2, 129.3, 128.9, 126.4, 125.3, 124.4, 123.9, 114.0, 58.1, 55.5.

IR v_{max} (cm^{-1}): 3367, 2922, 1774, 1707, 1604, 1489, 1313, 1246, 1172, 1026, 842, 721

HRMS (ESI) m/z: [M + H]+ Calcd for $C_{23}H_{18}N_2O_4Cl$ 421.0950; Found 421.0952

N-((1,3-dioxoisoindolin-2-yl)(naphthalen-2-yl)methyl)propionamide (3n)

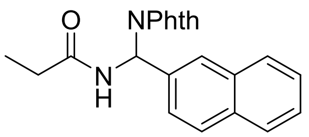

The title compound was prepared according to general procedure **C** from (1,3-dioxoisoindolin-2-yl)(naphthalen-2-yl)methyl acetate (75 mg, 0.217 mmol), $Ca(NTf_2)_2$ (6.5 mg, 0.011 mmol), *n*Bu_4NPF_6 (4.2 mg, 0.011 mmol) and propanamide (19 mg, 0.260 mmol) in 1,2- DCE (1.09 mL), reacting for 1 hr then being isolated by FCC (1:3 to 1:2 EtOAc:Hex) as a white solid. (31 mg, 40%).

RF (1:3 EtOAc:Hex): 0.17

^{1}H NMR (400 MHz, $CDCl_3$) δ 7.89 – 7.77 (m, 6H), 7.76 – 7.70 (m, 2H), 7.57 (d, *J* = 9.7 Hz, 1H), 7.53 (dd, *J* = 8.6, 1.9 Hz, 1H), 7.50 – 7.44 (m, 2H), 7.31 (d, *J* = 9.7 Hz, 1H), 2.44 – 2.28 (m, 2H), 1.20 (t, *J* = 7.6 Hz, 3H).

^{13}C{H} NMR (101 MHz, $CDCl_3$) δ 173.0, 167.6, 134.5, 134.4, 133.2, 133.0, 131.7, 129.0, 128.2, 127.6, 126.6, 126.6, 125.2, 123.7, 58.4, 29.5, 9.4.

IR v_{max} (cm^{-1}): 3371, 2924, 1774, 1707, 1500, 1351, 1209, 1097, 715

HRMS (ESI) m/z: [M + H]+ Calcd for $C_{22}H_{19}N_2O_3$ 359.1390; Found 359.1393

benzyl ((1,3-dioxoisoindolin-2-yl)(phenyl)methyl)carbamate (3o)

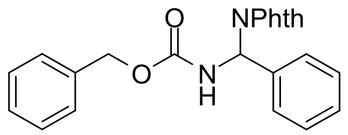

The title compound was prepared according to general procedure **C** from (1,3-dioxoisoindolin-2-yl)(phenyl)methyl acetate (75 mg, 0.254 mmol), $Ca(NTf_2)_2$ (7.6 mg, 0.013 mmol), *n*Bu_4NPF_6 (4.9 mg, 0.013 mmol) and benzyl carbamate (46 mg, 0.304 mmol) in 1,2- DCE (1.27 mL), reacting for 1 hr then being isolated by FCC (1:4 EtOAc:Hex) as a colourless oil. (98 mg, 99%).

RF (1:3 EtOAc:Hex): 0.34

^{1}H NMR (400 MHz, $CDCl_3$) δ 7.86 – 7.79 (m, 2H), 7.74 – 7.67 (m, 2H), 7.46 – 7.39 (m, 2H), 7.36 – 7.27 (m, 7H), 7.22 – 7.12 (m, 1H), 6.61 (d, *J* = 7.5 Hz, 1H), 5.14 (dd, *J* = 39.6, 12.2 Hz, 2H).

^{13}C{H} NMR (101 MHz, $CDCl_3$) δ 167.4, 155.3, 136.9, 135.9, 134.4, 131.8, 128.9, 128.6, 128.6, 128.3, 126.0, 123.7, 67.5, 60.5.

IR v_{max} (cm^{-1}): 3350, 3062, 3032, 2952, 1774, 1703, 1494, 1381, 1351, 1215, 1116, 1038, 883, 717, 693

HRMS (ESI) m/z: [M + Na]+ Calcd for $C_{23}H_{18}N_2O_4Na$ 409.1159; Found 409.1181

Phth protected α-aminonitriles

2-(1,3-dioxoisoindolin-2-yl)-2-phenylacetonitrile (4a)

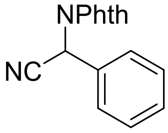

The title compound was prepared according to general procedure **C*** from (1,3-dioxoisoindolin-2-yl)(phenyl)methyl acetate (295 mg, 1.00 mmol), $Ca(NTf_2)_2$ (30 mg, 0.05 mmol), $n$$Bu_4NPF_6$ (19 mg, 0.05 mmol) and TMSCN (250 µL, 2.00 mmol) in 1,2- DCE (5.0 mL), reacting overnight then being isolated by FCC (1:6 EtOAc:Hex) as a white solid (202 mg, 77%).

RF (1:4 EtOAc:Hex): 0.42

^{1}H NMR (400 MHz, $CDCl_3$) δ 7.93 – 7.86 (m, 2H), 7.81 – 7.74 (m, 2H), 7.66 – 7.60 (m, 2H), 7.45 – 7.38 (m, 3H), 6.39 (s, 1H).

^{13}C{H} NMR (101 MHz, $CDCl_3$) δ 165.7, 134.8, 131.7, 131.3, 129.7, 129.2, 127.8, 124.1, 114.7, 43.0.

IR v_{max} (cm^{-1}): 2911, 1712, 1377, 1341, 1099, 885, 712

HRMS (ESI) m/z: [M + K]+ Calcd for $C_{16}H_{10}N_2O_2K$ 301.0374; Found 301.0380

2-(1,3-dioxoisoindolin-2-yl)-2-(4-fluorophenyl)acetonitrile (4b)

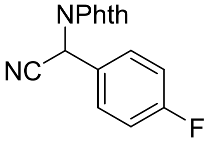

The title compound was prepared according to general procedure **C*** from (1,3-dioxoisoindolin-2-yl)(4-fluorophenyl)methyl acetate (75 mg, 0.239 mmol), $Ca(NTf_2)_2$ (7.2 mg, 0.012 mmol), $n$$Bu_4NPF_6$ (4.6 mg, 0.012 mmol) and TMSCN (60 µL, 0.478 mmol) in 1,2- DCE (1.20 mL), reacting overnight then being isolated by FCC (1:6 EtOAc:Hex) as a white solid (56 mg, 83%).

RF (1:4 EtOAc:Hex): 0.44

^{1}H NMR (400 MHz, $CDCl_3$) δ 7.92 – 7.86 (m, 2H), 7.82 – 7.76 (m, 2H), 7.67 – 7.60 (m, 2H), 7.14 – 7.06 (m, 2H), 6.36 (s, 1H).

^{13}C{H} NMR (101 MHz, $CDCl_3$) δ 165.6 (s), 163.3 (d, J = 250.1 Hz), 134.9 (s), 131.2 (s), 130.1 (d, J = 8.7 Hz), 127.8 (d, J = 3.4 Hz), 124.1 (s), 116.3 (d, J = 22.1 Hz), 114.6 (s), 42.4 (s).

^{19}F NMR (376 MHz, $CDCl_3$) δ -110.73.

IR v_{max} (cm^{-1}): 2912, 1716, 1604, 1507, 1377, 1330, 1220, 1095, 840, 713

HRMS (ESI) m/z: [M-CN]+ Calcd for $C_{15}H_9NO_2F$ 254.0612; Found 254.0615

2-(3-chlorophenyl)-2-(1,3-dioxoisoindolin-2-yl)acetonitrile (4c)

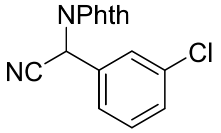

The title compound was prepared according to general procedure **C*** from (3-chlorophenyl)(1,3-dioxoisoindolin-2-yl)methyl acetate (50 mg, 0.152 mmol), $Ca(NTf_2)_2$ (4.6 mg, 0.0076 mmol), *n*Bu_4NPF_6 (2.9 mg, 0.0076 mmol) and TMSCN (38 μL, 0.303 mmol) in 1,2- DCE (0.76 mL), reacting overnight then being isolated by FCC (1:6 EtOAc:Hex) as a white solid (38 mg, 84%).

RF (1:4 EtOAc:Hex): 0.32

^{1}H NMR (400 MHz, $CDCl_3$) δ 7.94 – 7.87 (m, 2H), 7.83 – 7.77 (m, 2H), 7.61 – 7.57 (m, 1H), 7.56 – 7.51 (m, 1H), 7.40 – 7.35 (m, 2H), 6.36 (s, 1H).

$^{13}C\{H\}$ NMR (101 MHz, $CDCl_3$) δ 165.5, 135.2, 135.0, 133.5, 131.2, 130.5, 130.1, 127.9, 126.0, 124.2, 114.2, 42.4.

IR v_{max} (cm^{-1}): 2933, 1776, 1722, 1470, 1433, 1381, 1325, 1079, 788, 721, 684

HRMS (ESI) m/z: [M-H]- Calcd for $C_{16}H_8N_2O_2Cl$ 295.0280; Found 295.0280

2-(2-bromophenyl)-2-(1,3-dioxoisoindolin-2-yl)acetonitrile (4d)

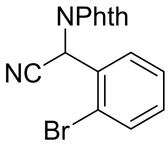

The title compound was prepared according to general procedure **C*** from (2-bromophenyl)(1,3-dioxoisoindolin-2-yl)methyl acetate (75 mg, 0.200 mmol), $Ca(NTf_2)_2$ (6.0 mg, 0.010 mmol), *n*Bu_4NPF_6 (3.9 mg, 0.010 mmol) and TMSCN (50 μL, 0.400 mmol) in 1,2-DCE (1.00 mL), reacting overnight then being isolated by FCC (1:6 EtOAc:Hex) as a white solid (50 mg, 73%).

RF (1:4 EtOAc:Hex): 0.47

^{1}H NMR (400 MHz, $CDCl_3$) δ 8.15 (dd, *J* = 7.9, 1.6 Hz, 1H), 7.92 – 7.85 (m, 2H), 7.83 – 7.74 (m, 2H), 7.58 (dd, *J* = 8.0, 1.2 Hz, 1H), 7.47 (td, *J* = 7.7, 1.2 Hz, 1H), 7.30 (td, *J* = 7.7, 1.6 Hz, 1H), 6.66 (s, 1H).

$^{13}C\{H\}$ NMR (101 MHz, $CDCl_3$) δ 165.5, 134.9, 133.5, 132.2, 131.5, 131.2, 130.0, 127.8, 124.1, 122.7, 114.8, 43.8.

IR v_{max} (cm^{-1}): 2916, 1772, 1718, 1466, 1373, 1326, 1082, 1026, 890, 717

HRMS (ESI) m/z: [M+Na]+ Calcd for $C_{16}H_9N_2O_2BrNa$ 362.9740; Found 362.9735

2-(3-bromo-2-fluorophenyl)-2-(1,3-dioxoisoindolin-2-yl)acetonitrile (4e)

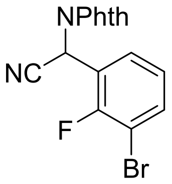

The title compound was prepared according to general procedure **C*** from (3-bromo-2-fluorophenyl)(1,3-dioxoisoindolin-2-yl)methyl acetate (75 mg, 0.191 mmol), $Ca(NTf_2)_2$ (5.7 mg, 0.010 mmol), *n*Bu_4NPF_6 (3.7 mg, 0.010 mmol) and TMSCN (48 µL, 0.382 mmol) in 1,2- DCE (0.96 mL), reacting overnight then being isolated by FCC (1:8 EtOAc:Hex) as a white solid (19 mg, 28%).

RF (1:4 EtOAc:Hex): 0.43

^{1}H NMR (400 MHz, $CDCl_3$) δ 8.02 – 7.97 (m, 1H), 7.93 – 7.87 (m, 2H), 7.82 – 7.76 (m, 2H), 7.63 (ddd, *J* = 8.1, 6.6, 1.5 Hz, 1H), 7.18 (td, *J* = 8.0, 1.0 Hz, 1H), 6.67 (s, 1H).

$^{13}C\{H\}$ NMR (101 MHz, $CDCl_3$) δ 165.2 (s), 156.2 (d, *J* = 251.4 Hz), 135.5 (s), 134.9 (s), 131.2 (s), 130.1 (d, *J* = 1.5 Hz), 125.5 (d, *J* = 4.7 Hz), 124.2 (s), 120.1 (d, *J* = 14.3 Hz), 113.8 (s), 109.7 (d, *J* = 20.4 Hz), 37.8 (d, *J* = 4.5 Hz).

^{19}F NMR (376 MHz, $CDCl_3$) δ -109.38.

IR v_{max} (cm^{-1}): 2920, 1772, 1729, 1466, 1377, 1328, 1080, 894, 792, 743, 719

HRMS (ESI) m/z: [M-H]- Calcd for $C_{16}H_7N_2O_2BrF$ 356.9680; Found 356.9682

2-(1,3-dioxoisoindolin-2-yl)-2-(p-tolyl)acetonitrile (4f)

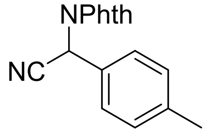

The title compound was prepared according to general procedure **C*** from (1,3-dioxoisoindolin-2-yl)(p-tolyl)methyl acetate (50 mg, 0.162 mmol), $Ca(NTf_2)_2$ (4.9 mg, 0.008 mmol), *n*Bu_4NPF_6 (3.1 mg, 0.008 mmol) and TMSCN (41 µL, 0.324 mmol) in 1,2- DCE (0.81 mL), reacting overnight then being isolated by FCC (1:6 EtOAc:Hex) as a white solid (39 mg, 87%).

RF (1:4 EtOAc:Hex): 0.36

^{1}H NMR (400 MHz, $CDCl_3$) δ 7.94 – 7.83 (m, 2H), 7.80 – 7.73 (m, 2H), 7.51 (d, *J* = 8.1 Hz, 2H), 7.20 (d, *J* = 8.0 Hz, 2H), 6.35 (s, 1H), 2.34 (s, 3H).

^{13}C{H} NMR (101 MHz, $CDCl_3$) δ 165.7, 139.9, 134.8, 131.3, 129.9, 128.8, 127.8, 124.0, 114.9, 42.8, 21.2.

IR v_{max} (cm^{-1}): 3069, 2909, 1787, 1716, 1513, 1466, 1369, 1332, 1097, 1082, 939, 885, 820, 713

HRMS (ESI) m/z: [M+H]+ Calcd for $C_{17}H_{13}N_2O_2$ 277.0972; Found 277.0974

2-(1,3-dioxoisoindolin-2-yl)-2-(2-methoxyphenyl)acetonitrile (4g)

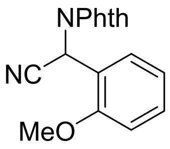

The title compound was prepared according to general procedure **C*** from (1,3-dioxoisoindolin-2-yl)(2-methoxyphenyl)methyl acetate (50 mg, 0.154 mmol), $Ca(NTf_2)_2$ (4.6 mg, 0.008 mmol), *n*Bu_4NPF_6 (3.0 mg, 0.008 mmol) and TMSCN (39 µL, 0.308 mmol) in 1,2-DCE (0.77 mL), reacting overnight then being isolated by FCC (1:6 EtOAc:Hex) as a white solid (35 mg, 78%).

RF (1:4 EtOAc:Hex): 0.30

^{1}H NMR (400 MHz, $CDCl_3$) δ 7.97 – 7.90 (m, 1H), 7.91 – 7.83 (m, 2H), 7.79 – 7.72 (m, 2H), 7.38 (td, *J* = 8.1, 1.6 Hz, 1H), 7.05 (td, *J* = 7.6, 1.0 Hz, 1H), 6.91 – 6.84 (m, 1H), 6.71 (s, 1H), 3.80 (s, 3H).

^{13}C{H} NMR (101 MHz, $CDCl_3$) δ 165.6, 156.3, 134.6, 131.4, 131.3, 130.5, 123.9, 120.7, 119.1, 115.3, 110.7, 55.7, 38.7.

IR v_{max} (cm^{-1}): 2914, 2845, 1774, 1720, 1492, 1466, 1375, 1332, 1250, 1108, 1025, 754, 715

HRMS (ESI) m/z: [M + H]+ Calcd for $C_{17}H_{13}N_2O_3$ 293.0921; Found 293.0924

2-(1,3-dioxoisoindolin-2-yl)-2-(naphthalen-2-yl)acetonitrile (4h)

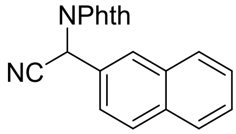

The title compound was prepared according to general procedure **C*** (1,3-dioxoisoindolin-2-yl)(naphthalen-2-yl)methyl acetate (50 mg, 0.145 mmol), $Ca(NTf_2)_2$ (4.3 mg, 0.007 mmol), nBu_4NPF_6 (2.8 mg, 0.007 mmol) and TMSCN (36 µL, 0.290 mmol) in 1,2- DCE (0.73 mL), reacting overnight then being isolated by FCC (1:6 EtOAc:Hex) as a white solid (41 mg, 91%).

RF (1:4 EtOAc:Hex): 0.23

^{1}H NMR (400 MHz, $CDCl_3$) δ 8.17 (d, *J* = 0.9 Hz, 1H), 7.91 – 7.78 (m, 5H), 7.77 – 7.71 (m, 2H), 7.60 (dd, *J* = 8.6, 1.9 Hz, 1H), 7.55 – 7.48 (m, 2H), 6.55 (s, 1H).

^{13}C{H} NMR (101 MHz, $CDCl_3$) δ 165.7, 134.8, 133.5, 132.9, 131.3, 129.4, 128.9, 128.4, 127.8, 127.7, 127.3, 127.0, 124.4, 124.1, 114.8, 43.2.

IR v_{max} (cm^{-1}): 2901, 1787, 1716, 1602, 1466, 1369, 1328, 1097, 1084, 911, 833, 758, 711

HRMS (ESI) m/z: [M+NH_4]+ Calcd for $C_{20}H_{16}N_3O_2$ 330.1237; Found 330.1243

2-(1,3-dioxoisoindolin-2-yl)-2-(furan-2-yl)acetonitrile (4i)

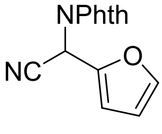

The title compound was prepared according to general procedure **C*** (1,3-dioxoisoindolin-2-yl)(furan-2-yl)methyl acetate (50 mg, 0.175 mmol), $Ca(NTf_2)_2$ (5.3 mg, 0.009 mmol), nBu_4NPF_6 (3.4 mg, 0.009 mmol) and TMSCN (44 µL, 0.350 mmol) in 1,2- DCE (0.88 mL), reacting overnight then being isolated by FCC (1:6 EtOAc:Hex) as a white solid (13 mg, 29%).

RF (1:4 EtOAc:Hex): 0.28

^{1}H NMR (400 MHz, $CDCl_3$) δ 7.95 – 7.89 (m, 2H), 7.82 – 7.77 (m, 2H), 7.39 (dd, *J* = 1.8, 0.7 Hz, 1H), 6.78 (dt, *J* = 3.4, 0.8 Hz, 1H), 6.45 – 6.40 (m, 2H).

$^{13}C\{H\}$ NMR (101 MHz, $CDCl_3$) δ 165.3, 144.0, 143.6, 134.9, 131.2, 124.2, 113.2, 111.4, 111.2, 36.9.

IR v_{max} (cm^{-1}): 2918, 2849, 1771, 1720, 1371, 1321, 1108, 1012, 922, 751, 713

HRMS (ESI) m/z: [M-CN]+ Calcd for $C_{13}H_8NO_3$ 226.0499; Found 226.0501

2-(1,3-dioxoisoindolin-2-yl)-2-(thiophen-2-yl)acetonitrile (4j)

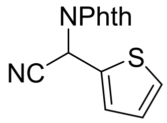

The title compound was prepared according to general procedure **C*** from (1,3-dioxoisoindolin-2-yl)(thiophen-2-yl)methyl acetate (75 mg, 0.249 mmol), $Ca(NTf_2)_2$ (7.5 mg, 0.012 mmol), *n*Bu_4NPF_6 (4.8 mg, 0.012 mmol) and TMSCN (63 µL, 0.500 mmol) in 1,2- DCE (1.25 mL), reacting overnight then being isolated by FCC (1:6 EtOAc:Hex) as a white solid (53 mg, 79%).

RF (1:4 EtOAc:Hex): 0.35

^1H NMR (400 MHz, $CDCl_3$) δ 7.94 – 7.88 (m, 2H), 7.82 – 7.76 (m, 2H), 7.44 (d, *J* = 3.5 Hz, 1H), 7.36 (dd, *J* = 5.1, 1.2 Hz, 1H), 7.01 (dd, *J* = 5.1, 3.6 Hz, 1H), 6.55 (s, 1H).

$^{13}C\{H\}$ NMR (101 MHz, $CDCl_3$) δ 165.3, 134.9, 133.3, 131.2, 129.5, 128.1, 127.2, 124.2, 114.3, 38.2.

IR v_{max} (cm^{-1}): 2911, 1776, 1716, 1466, 1377, 1334, 1105, 933, 879, 713

HRMS (ESI) m/z: [M-CN]+ Calcd for $C_{13}H_8NO_2S$ 242.0270; Found 242.0274

2-cyclohexyl-2-(1,3-dioxoisoindolin-2-yl)acetonitrile (4k)

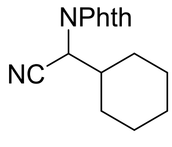

The title compound was prepared according to general procedure **C*** from cyclohexyl(1,3-dioxoisoindolin-2-yl)methyl acetate (23 mg, 0.076 mmol), $Ca(NTf_2)_2$ (2.3 mg, 0.004 mmol), *n*Bu_4NPF_6 (1.5 mg, 0.004 mmol) and TMSCN (19 µL, 0.153 mmol) in 1,2- DCE (0.38 mL), reacting overnight then being isolated by FCC (1:6 EtOAc:Hex) as a white solid (18 mg, 88%).

RF (1:4 EtOAc:Hex): 0.43

^{1}H NMR (400 MHz, $CDCl_3$) δ 7.95 – 7.88 (m, 2H), 7.84 – 7.76 (m, 2H), 4.86 – 4.77 (m, 1H), 2.34 (qt, *J* = 11.3, 3.5 Hz, 1H), 2.24 – 2.14 (m, 1H), 1.90 – 1.80 (m, 1H), 1.76 – 1.64 (m, 2H), 1.62 – 1.52 (m, 1H), 1.41 – 1.28 (m, 1H), 1.24 – 1.11 (m, 3H), 1.02 – 0.89 (m, 1H).

$^{13}C\{H\}$ NMR (101 MHz, $CDCl_3$) δ 166.3, 134.8, 131.2, 124.0, 115.5, 45.4, 38.6, 30.1, 29.0, 25.7, 25.1, 25.0.

IR v_{max} (cm^{-1}): 2927, 2849, 1772, 1718, 1466, 1453, 1377, 1080, 916, 862, 793, 713

HRMS (ESI) m/z: [M + NH_4]+ Calcd for $C_{16}H_{20}N_3O_2$ 286.1550; Found 286.1554

2-cyclopropyl-2-(1,3-dioxoisoindolin-2-yl)acetonitrile (4l)

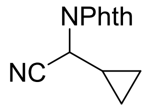

The title compound was prepared according to general procedure **C*** from cyclopropyl(1,3-dioxoisoindolin-2-yl)methyl acetate (75 mg, 0.289 mmol), $Ca(NTf_2)_2$ (8.7 mg, 0.015 mmol), *n*Bu_4NPF_6 (5.6 mg, 0.015 mmol) and TMSCN (72 µL, 0.579 mmol) in 1,2- DCE (1.45 mL), reacting overnight then being isolated by FCC (1:6 EtOAc:Hex) as a white solid (56 mg, 86%).

RF (1:4 EtOAc:Hex): 0.38

^{1}H NMR (400 MHz, $CDCl_3$) δ 7.95 – 7.90 (m, 2H), 7.84 – 7.80 (m, 2H), 4.45 (d, *J* = 9.6 Hz, 1H), 1.91 – 1.82 (m, 1H), 0.94 – 0.84 (m, 1H), 0.77 – 0.61 (m, 2H), 0.55 – 0.44 (m, 1H).

$^{13}C\{H\}$ NMR (101 MHz, $CDCl_3$) δ 166.1, 134.8, 131.3, 124.0, 115.3, 44.3, 13.4, 4.8, 4.7.

IR v_{max} (cm^{-1}): 3006, 2927, 1770, 1712, 1466, 1381, 1330, 1187, 1095, 1030, 903, 838, 797, 711

HRMS (ESI) m/z: [M+H]+ Calcd for $C_{13}H_{11}N_2O_2$ 227.0815; Found 227.0814

N-Boc α-functionalised amines

***tert*-butyl (1-phenylbut-3-en-1-yl)carbamate (5a)**

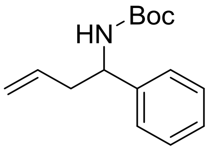

The title compound was prepared according to general procedure **D** from (1,3-dioxoisoindolin-2-yl)(phenyl)methyl acetate (100 mg, 0.339 mmol), $Ca(NTf_2)_2$ (10 mg, 0.017 mmol), *n*Bu_4NPF_6 (7 mg, 0.017 mmol) and allyl TMS (64 µL, 0.406 mmol) in 1,2- DCE (1.7 mL), reacting for 1

hr, then $MeNH_2$ (33wt% in EtOH) (206 µL, 1.695 mmol) in EtOH (3.4 mL) overnight, followed by Boc_2O (156 µL, 0.678 mmol), Et_3N (47 µL, 0.339 mmol) and DMAP (cat.) in dry THF (1.7 mL). Following completion of the Boc-protection (1 h), purification by FCC (1:12 EtOAc:Hex) afforded the pure compound as a white solid (56 mg, 67% over 3 steps).

RF (1:12 EtOAc:Hex):0.50

^{1}H NMR (400 MHz, $CDCl_3$) δ 7.37 – 7.20 (m, 5H), 5.75 – 5.61 (m, 1H), 5.17 – 5.03 (m, 2H), 4.89 (s, br, 1H), 4.74 (s, br, 1H), 2.51 (s, br, 2H), 1.41 (s, 9H).

^{13}C{H} NMR (101 MHz, $CDCl_3$) δ 155.2, 142.4, 134.0, 128.5, 127.1, 126.2, 118.2, 79.5, 54.0, 41.3, 28.4.

*Data in accordance with literature [4]

***tert*-butyl (1-(4-methoxyphenyl)but-3-en-1-yl)carbamate (5b)**

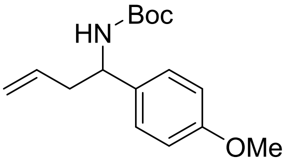

The title compound was prepared according to general procedure **D** from (1,3-dioxoisoindolin-2-yl)(4-methoxyphenyl)methyl acetate (75 mg, 0.231 mmol), $Ca(NTf_2)_2$ (7 mg, 0.012 mmol), nBu_4NPF_6 (4.5 mg, 0.012 mmol) and allyl TMS (44 µL, 0.277 mmol) in 1,2- DCE (1.2 mL), reacting for 1 hr, then $MeNH_2$ (33wt% in EtOH) (134 µL, 1.014 mmol) in EtOH (2.3 mL) overnight, followed by Boc_2O (105 µL, 0.461 mmol), Et_3N (32 µL, 0.231 mmol) and DMAP (cat.) in dry THF (1.2 mL). Following completion of the Boc-protection (1 h), purification by FCC (1:12 EtOAc:Hex) afforded the pure compound as a white solid (49 mg, 77% over 3 steps).

RF (1:12 EtOAc:Hex):0.17

^{1}H NMR (400 MHz, $CDCl_3$) δ 7.18 (d, J = 8.5 Hz, 2H), 6.86 (d, J = 8.6 Hz, 2H), 5.74 – 5.61 (m, 1H), 5.14 – 5.02 (m, 2H), 4.81 (s, br, 1H), 4.67 (s, br, 1H), 3.79 (s, 3H), 2.55 – 2.45 (m, br, 2H), 1.41 (s, 9H).

^{13}C{H} NMR (101 MHz, $CDCl_3$) δ 158.7, 155.2, 134.6, 134.2, 127.4, 118.0, 113.9, 79.4, 55.3, 53.6, 41.2, 28.4.

*Data in accordance with literature [4]

***tert*-butyl (1-(p-tolyl)but-3-en-1-yl)carbamate (5c)**

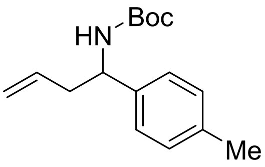

The title compound was prepared according to general procedure **D** from (1,3-dioxoisoindolin-2-yl)(p-tolyl)methyl acetate (75 mg, 0.242 mmol), $Ca(NTf_2)_2$ (7 mg, 0.012 mmol), *n*Bu_4NPF_6 (4.7 mg, 0.012 mmol) and allyl TMS (46 μL, 0.290 mmol) in 1,2- DCE (1.2 mL), reacting for 1 hr, then $MeNH_2$ (33wt% in EtOH) (140 μL, 1.21 mmol) in EtOH (2.3 mL) overnight, followed by Boc_2O (111 μL, 0.484 mmol), Et_3N (34 μL, 0.242 mmol) and DMAP (cat.) in dry THF (1.2 mL). Following completion of the Boc-protection (1 h), purification by FCC (1:19 EtOAc:Hex) afforded the pure compound as a white solid (35 mg, 55% over 3 steps).

RF (1:12 EtOAc:Hex):0.29

^{1}H NMR (400 MHz, $CDCl_3$) δ 7.20 – 7.07 (m, 4H), 5.74 – 5.61 (m, 1H), 5.15 – 5.02 (m, 2H), 4.84 (s, br, 1H), 4.70 (s, br, 1H), 2.56 – 2.44 (m, br, 2H), 2.32 (s, 3H), 1.41 (s, 9H).

^{13}C{H} NMR (101 MHz, $CDCl_3$) δ 155.2, 139.4, 136.7, 134.1, 129.2, 126.2, 118.0, 79.4, 53.8, 41.2, 28.4, 21.1.

*Data in accordance with literature [4]

***tert*-butyl (1-(2-methoxyphenyl)but-3-en-1-yl)carbamate (5d)**

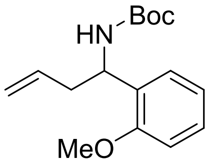

The title compound was prepared according to general procedure **D** from (1,3-dioxoisoindolin-2-yl)(2-methoxyphenyl)methyl acetate (40 mg, 0.123 mmol), $Ca(NTf_2)_2$ (3.7 mg, 0.006 mmol), *n*Bu_4NPF_6 (2.4 mg, 0.006 mmol) and allyl TMS (23 μL, 0.148 mmol) in 1,2- DCE (0.62 mL), reacting for 1 hr, then $MeNH_2$ (33wt% in EtOH) (71 μL, 0.615 mmol) in EtOH (1.23 mL) overnight, followed by Boc_2O (57 μL, 0.246 mmol), Et_3N (18 μL, 0.123 mmol) and DMAP (cat.) in dry THF (0.62 mL). Following completion of the Boc-protection (1 h), purification by FCC (1:15 EtOAc:Hex) afforded the pure compound as a white solid (24 mg, 70% over 3 steps).

RF (1:12 EtOAc:Hex):0.37

^{1}H NMR (400 MHz, $CDCl_3$) δ 7.25 – 7.19 (m, 1H), 7.17 – 7.13 (m, 1H), 6.94 – 6.84 (m, 2H), 5.75 – 5.61 (m, 1H), 5.34 (d, *J* = 6.6 Hz, 1H), 5.11 – 4.97 (m, 2H), 4.97 – 4.85 (m, 1H), 3.85 (s, 3H), 2.58 – 2.43 (m, 2H), 1.42 (s, 9H).

$^{13}C\{H\}$ NMR (101 MHz, $CDCl_3$) δ 156.9, 155.2, 135.0, 130.0, 128.2, 128.1, 120.5, 117.2, 110.8, 79.1, 55.3, 51.9, 39.9, 28.4.

*Data in accordance with literature [4]

***tert*-butyl (1-(4-fluorophenyl)but-3-en-1-yl)carbamate (5e)**

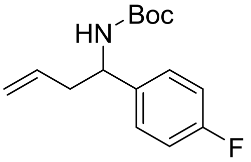

The title compound was prepared according to general procedure **D** from (1,3-dioxoisoindolin-2-yl)(4-fluorophenyl)methyl acetate (75 mg, 0.229 mmol), $Ca(NTf_2)_2$ (7 mg, 0.012 mmol), $n Bu_4NPF_6$ (4.6 mg, 0.012 mmol) and allyl TMS (46 µL, 0.290 mmol) in 1,2- DCE (1.2 mL), reacting for 2 hr, then $MeNH_2$ (33wt% in EtOH) (134 µL, 1.21 mmol) in EtOH (2.3 mL) overnight, followed by Boc_2O (110 µL, 0.484 mmol), Et_3N (33 µL, 0.242 mmol) and DMAP (cat.) in dry THF (1.2 mL). Following completion of the Boc-protection (1 h), purification by FCC (1:19 EtOAc:Hex) afforded the pure compound as a white solid (18 mg, 26% over 3 steps).

RF (1:12 EtOAc:Hex):0.28

^{1}H NMR (400 MHz, $CDCl_3$) δ 7.23 (dd, *J* = 8.2, 5.5 Hz, 2H), 7.01 (t, *J* = 8.6 Hz, 2H), 5.73 – 5.59 (m, 1H), 5.16 – 5.05 (m, 2H), 4.84 (s, br, 1H), 4.70 (s, br, 1H), 2.48 (m, br, 2H), 1.35 (s, 9H).

$^{13}C\{H\}$ NMR (101 MHz, $CDCl_3$) δ 161.9 (d, *J* = 244.9 Hz), 155.1 (s), 138.2 (s), 133.7 (s), 127.8 (d, *J* = 8.0 Hz), 118.5 (s), 115.3 (d, *J* = 21.4 Hz), 79.7 (s), 53.43 (s), 41.2 (s), 28.3 (s).

^{19}F NMR (376 MHz, $CDCl_3$) δ -115.84.

*Data in accordance with literature [4]

tert-butyl (1-(3-chlorophenyl)but-3-en-1-yl)carbamate (5f)

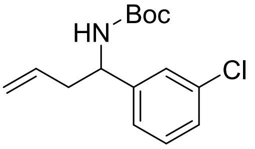

The title compound was prepared according to general procedure **D** from (3-chlorophenyl)(1,3-dioxoisoindolin-2-yl)methyl acetate (75 mg, 0.227 mmol), $Ca(NTf_2)_2$ (6.6 mg, 0.011 mmol), *n*Bu_4NPF_6 (4.3 mg, 0.011 mmol) and allyl TMS (43 µL, 0.272 mmol) in 1,2-DCE (1.1 mL reacting for 5 hr, then $MeNH_2$ (33wt% in EtOH) (132 µL, 1.14 mmol) in EtOH (2.3 mL) overnight, followed by Boc_2O (104 µL, 0.454 mmol), Et_3N (32 µL, 0.227 mmol) and DMAP (cat.) in dry THF (1.1 mL). Following completion of the Boc-protection (1 h), purification by FCC (1:19 EtOAc:Hex) afforded the pure compound as a white solid (21 mg, 30% over 3 steps).

RF (1:12 EtOAc:Hex):0.31

^{1}H NMR (400 MHz, $CDCl_3$) δ 7.29 – 7.19 (m, 3H), 7.17 – 7.12 (m, 1H), 5.73 – 5.58 (m, 1H), 5.20 – 5.06 (m, 2H), 4.87 (s, br, 1H), 4.71 (s, br, 1H), 2.56 – 2.40 (m, br, 2H), 1.41 (s, 9H).

^{13}C{H} NMR (101 MHz, $CDCl_3$) δ 155.1, 144.7, 134.4, 133.4, 129.8, 127.3, 126.4, 124.5, 118.7, 79.8, 53.6, 41.1, 28.3.

*Data in accordance with literature [5]

tert-butyl (1-(2-bromophenyl)but-3-en-1-yl)carbamate (5g)

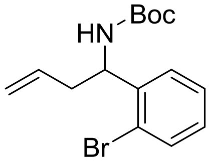

The title compound was prepared according to general procedure **D** from (2-bromophenyl)(1,3-dioxoisoindolin-2-yl)methyl acetate (75 mg, 0.200 mmol), $Ca(NTf_2)_2$ (6.0 mg, 0.010 mmol), *n*Bu_4NPF_6 (3.9 mg, 0.010 mmol) and allyl TMS (38 µL, 0.240 mmol) in 1,2-DCE (1.0 mL reacting for 5 hr, then $MeNH_2$ (33wt% in EtOH) (116 µL, 1.00 mmol) in EtOH (2.0 mL) overnight, followed by Boc_2O (92 µL, 0.400 mmol), Et_3N (29 µL, 0.200 mmol) and DMAP (cat.) in dry THF (1.0 mL). Following completion of the Boc-protection (1 h), purification by FCC (1:19 EtOAc:Hex) afforded the pure compound as a white solid (22 mg, 31% over 3 steps).

RF (1:12 EtOAc:Hex):0.28

^{1}H NMR (400 MHz, $CDCl_3$) δ 7.53 (d, *J* = 7.9 Hz, 1H), 7.36 – 7.21 (m, 2H), 7.16 – 7.04 (m, 1H), 5.79 – 5.62 (m, 1H), 5.22 – 4.79 (m, 4H), 2.56 (s, 1H), 2.50 – 2.27 (m, 1H), 1.41 (s, 9H).

$^{13}C\{H\}$ NMR (101 MHz, $CDCl_3$) δ 155.0, 141.5, 133.6, 133.2, 128.5, 127.5, 127.2, 122.7, 118.6, 79.7, 53.5, 39.5, 28.3.

*Data in accordance with literature [6]

tert-butyl ((5-bromo-1-methyl-1H-indol-3-yl)(phenyl)methyl)carbamate (5h)

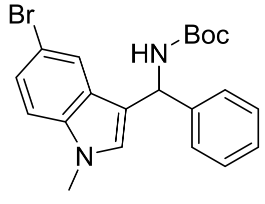

The title compound was prepared according to general procedure **D** from (1,3-dioxoisoindolin-2-yl)(phenyl)methyl acetate (75 mg, 0.254 mmol), $Ca(NTf_2)_2$ (7.6 mg, 0.013 mmol), *n*Bu_4NPF_6 (4.9 mg, 0.013 mmol) and 5-bromo-*N*-methylindole (64 mg, 0.305 mmol) in 1,2- DCE (1.27 mL), reacting for 15 min, then $MeNH_2$ (33wt% in EtOH) (148 μL, 1.27 mmol) in EtOH (2.54 mL) overnight, followed by Boc_2O (117 μL, 0.508 mmol), Et_3N (35 μL, 0.254 mmol) and DMAP (cat.) in dry THF (1.27 mL). Following completion of the Boc-protection (1 h), purification by FCC (1:5 EtOAc:Hex) afforded the pure compound as a white solid. (41 mg, 39% over 3 steps).

RF (1:4 EtOAc:Hex):0.33

^{1}H NMR (400 MHz, $CDCl_3$) δ 7.73 – 7.60 (m, 1H), 7.39 – 7.32 (m, 4H), 7.31 – 7.26 (m, 2H), 7.16 – 7.10 (m, 1H), 6.57 (s, 1H), 6.14 (s, 1H), 5.15 (s, 1H), 3.64 (s, 3H), 1.46 (s, 9H).

$^{13}C\{H\}$ NMR (101 MHz, $CDCl_3$) δ 155.2, 141.8, 136.2, 129.0, 128.5, 128.1, 127.3, 126.8, 124.9, 122.2, 116.3, 112.9, 110.9, 79.8, 51.5, 32.9, 28.4.

IR ν_{max} (cm^{-1}): 3408, 2976, 2931, 1710, 1495, 1474, 1366, 1237, 1157, 1043, 1012, 881, 799, 754, 705

HRMS (ESI) m/z: [M-BocNH]+ Calcd for $C_{16}H_{13}N^{81}Br$ 300.0205, Found 300.0211

tert-butyl ((5-bromo-1-methyl-1H-indol-3-yl)(4-methoxyphenyl)methyl)carbamate (5i)

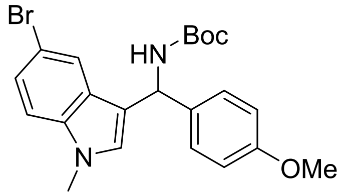

The title compound was prepared according to general procedure **D** from (1,3-dioxoisoindolin-2-yl)(4-methoxyphenyl)methyl acetate (75 mg, 0.231 mmol), $Ca(NTf_2)_2$ (6.9 mg, 0.012 mmol), *n*Bu_4NPF_6 (4.5 mg, 0.012 mmol) and 5-bromo-*N*-methylindole (58 mg, 0.277 mmol) in 1,2-DCE (1.20 mL), reacting for 15 min, then $MeNH_2$ (33wt% in EtOH) (135 μL, 1.16 mmol) in EtOH (2.31 mL) overnight, followed by Boc_2O (106 μL, 0.461 mmol), Et_3N (32 μL, 0.231 mmol) and DMAP (cat.) in dry THF (1.20 mL). Following completion of the Boc-protection (1 h), purification by FCC (1:8 to 1:6 EtOAc:Hex) afforded the pure compound as an off-white solid. (22 mg, 21% over 3 steps).

RF (1:4 EtOAc:Hex):0.28

^{1}H NMR (400 MHz, $CDCl_3$) δ 7.62 (s, 1H), 7.33 – 7.24 (m, 3H), 7.14 (d, *J* = 8.7 Hz, 1H), 6.91 – 6.85 (m, 2H), 6.61 (s, 1H), 6.08 (s, 1H), 5.11 (s, 1H), 3.81 (s, 3H), 3.67 (s, 3H), 1.46 (s, 9H).

^{13}C{H} NMR (101 MHz, $CDCl_3$) δ 158.8, 155.1, 136.2, 128.8, 128.0, 128.0, 124.9, 122.2, 116.5, 113.9, 112.8, 110.9, 79.7, 55.3, 51.0, 32.9, 28.4.

IR v_{max} (cm^{-1}): 3330, 2978, 2953, 2924, 1675, 1608, 1511, 1474, 1366, 1159, 1023, 790

HRMS (ESI) m/z: [M+Na]+ Calcd for $C_{22}H_{25}N_2O_3Na^{81}Br$ 469.0920; Found 469.0927

N-Protected α-functionalised amines

(9H-fluoren-9-yl)methyl (1-phenylbut-3-en-1-yl)carbamate (6a)

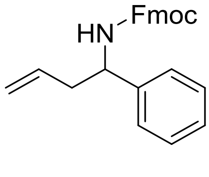

The title compound was prepared according to general procedure **E** from (1,3-dioxoisoindolin-2-yl)(phenyl)methyl acetate (75 mg, 0.254 mmol), $Ca(NTf_2)_2$ (7.6 mg, 0.013 mmol), *n*Bu_4NPF_6 (4.9 mg, 0.013 mmol) and allyl TMS (48 μL, 0.305 mmol) in 1,2- DCE (1.27 mL), reacting for 1 hr, then $MeNH_2$ (33wt% in EtOH) (148 μL, 1.27 mmol) in EtOH (2.54 mL) overnight, followed by FmocCl (79 mg, 0.305 mmol) and Et_3N (35 μL, 0.254 mmol) in dry DCM (1.27 mL). Following completion of the Fmoc-protection (1 h), purification by FCC (1:19 to 1:6 EtOAc:Hex) afforded the pure compound as a white solid (59 mg, 63% over 3 steps).

RF (1:4 EtOAc:Hex):0.83

^{1}H NMR (400 MHz, $CDCl_3$) δ 7.78 – 7.68 (m, 2H), 7.63 – 7.10 (m, 11H), 5.76 – 5.55 (m, 1H), 5.21 – 5.00 (m, 3H), 4.87 – 4.58 (m, 1H), 4.49 – 4.30 (m, 2H), 4.26 – 4.05 (m, 1H), 2.65 – 2.34 (m, 2H).

$^{13}C\{H\}$ NMR (101 MHz, $CDCl_3$) δ 155.7, 144.0, 141.9, 141.3, 133.9, 128.7, 127.7, 127.4, 127.1, 127.1, 126.3, 125.1, 120.0, 118.4, 66.6, 54.5, 47.3, 41.0.

*Data in accordance with literature.[7]

***N*-(1-phenylbut-3-en-1-yl)benzamide (6b)**

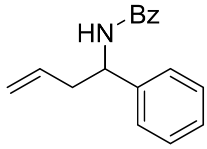

The title compound was prepared according to general procedure **E** from (1,3-dioxoisoindolin-2-yl)(phenyl)methyl acetate (75 mg, 0.254 mmol), $Ca(NTf_2)_2$ (7.6 mg, 0.013 mmol), *n*Bu_4NPF_6 (4.9 mg, 0.013 mmol) and allyl TMS (48 μL, 0.305 mmol) in 1,2- DCE (1.27 mL), reacting for 1 hr, then $MeNH_2$ (33wt% in EtOH) (148 μL, 1.27 mmol) in EtOH (2.54 mL) overnight, followed by BzCl (35 μL, 0.305 mmol) and Et_3N (35 μL, 0.254 mmol) in dry DCM (1.27 mL). Following completion of the Bz-protection (1 h), purification by FCC (1:6 EtOAc:Hex) afforded the pure compound as a white solid (31 mg, 49% over 3 steps).

RF (1:4 EtOAc:Hex):0.31

^{1}H NMR (400 MHz, $CDCl_3$) δ 7.81 – 7.72 (m, 2H), 7.52 – 7.45 (m, 1H), 7.45 – 7.38 (m, 2H), 7.34 (d, J = 4.4 Hz, 4H), 7.29 – 7.23 (m, 1H), 6.52 (d, J = 7.2 Hz, 1H), 5.76 (ddt, J = 17.1, 10.1, 7.0 Hz, 1H), 5.29 (dd, J = 14.4, 6.9 Hz, 1H), 5.22 – 5.07 (m, 2H), 2.69 (t, J = 6.8 Hz, 2H).

$^{13}C\{H\}$ NMR (101 MHz, $CDCl_3$) δ 166.8, 141.6, 134.6, 134.1, 131.5, 128.7, 128.6, 127.4, 127.0, 126.5, 118.5, 52.8, 40.6.

*Data in accordance with literature.[8]

allyl (1-phenylbut-3-en-1-yl)carbamate (6c)

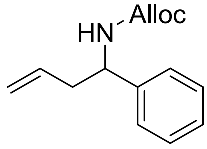

The title compound was prepared according to general procedure **E** from (1,3-dioxoisoindolin-2-yl)(phenyl)methyl acetate (75 mg, 0.254 mmol), $Ca(NTf_2)_2$ (7.6 mg, 0.013 mmol), *n*Bu_4NPF_6 (4.9 mg, 0.013 mmol) and allyl TMS (48 µL, 0.305 mmol) in 1,2- DCE (1.27 mL), reacting for 1 hr, then $MeNH_2$ (33wt% in EtOH) (148 µL, 1.27 mmol) in EtOH (2.54 mL) overnight, followed by AllocCl (31 µL, 0.305 mmol) and Et_3N (35 µL, 0.254 mmol) in dry DCM (1.27 mL). Following completion of the Alloc-protection (1 h), purification by FCC (1:9 EtOAc:Hex) afforded the pure compound as a viscous colourless oil. (34 mg, 58% over 3 steps).

RF (1:8 EtOAc:Hex):0.22

^{1}H NMR (400 MHz, $CDCl_3$) δ 7.39 – 7.22 (m, 5H), 5.99 – 5.80 (m, 1H), 5.68 (ddt, *J* = 17.2, 10.1, 7.0 Hz, 1H), 5.37 – 5.01 (m, 5H), 4.89 – 4.72 (m, 1H), 4.64 – 4.45 (m, 2H), 2.55 (t, *J* = 6.1 Hz, 2H).

^{13}C{H} NMR (101 MHz, $CDCl_3$) δ 155.5, 142.0, 133.8, 132.8, 128.6, 127.3, 126.2, 118.4, 117.8, 65.6, 54.4, 41.1.

*Data in accordance with literature.[9]

***N*-(1-(thiophen-2-yl)but-3-en-1-yl)acetamide (6d)**

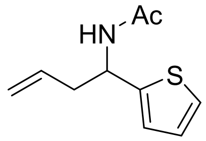

The title compound was prepared according to general procedure **E** from (1,3-dioxoisoindolin-2-yl)(thiophen-2-yl)methyl acetate (75 mg, 0.249 mmol), $Ca(NTf_2)_2$ (7.4 mg, 0.012 mmol), *n*Bu_4NPF_6 (4.8 mg, 0.012 mmol) and allyl TMS (47 µL, 0.299 mmol) in 1,2- DCE (1.25 mL), reacting for 1 hr, then $MeNH_2$ (33wt% in EtOH) (145 µL, 1.27 mmol) in EtOH (2.49 mL) overnight, followed by AcCl (21 µL, 0.299 mmol) and Et_3N (35 µL, 0.249 mmol) in dry DCM (1.25 mL). Following completion of the Ac-protection (1 h), purification by FCC (1:4 to 1:2 EtOAc:Hex) afforded the pure compound as a white solid. (43 mg, 88% over 3 steps).

RF (1:3 EtOAc:Hex):0.19

^{1}H NMR (400 MHz, $CDCl_3$) δ 7.19 (dd, *J* = 4.2, 2.1 Hz, 1H), 6.98 – 6.90 (m, 2H), 6.10 (d, *J* = 7.4 Hz, 1H), 5.75 (ddt, *J* = 17.1, 10.2, 7.0 Hz, 1H), 5.37 (dd, *J* = 15.3, 6.8 Hz, 1H), 5.19 – 5.06 (m, 2H), 2.69 – 2.57 (m, 2H), 1.97 (s, 3H).

$^{13}C\{H\}$ NMR (101 MHz, $CDCl_3$) δ 169.3, 145.5, 133.6, 126.8, 124.4, 124.2, 118.5, 48.3, 40.7, 23.24.

IR v_{max} (cm^{-1}): 3240, 3065, 2953, 2849, 1634, 1554, 1433, 1366, 1291, 1077, 1034, 926, 834, 708

HRMS (ESI) m/z: [M + H]+ Calcd for $C_{10}H_{13}NOS$ 196.0791; Found 196.0784

benzyl (1-(2-methoxyphenyl)but-3-en-1-yl)carbamate (6e)

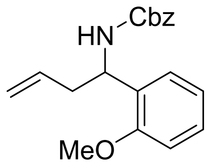

The title compound was prepared according to general procedure **E** from (1,3-dioxoisoindolin-2-yl)(2-methoxyphenyl)methyl acetate (75 mg, 0.231 mmol), $Ca(NTf_2)_2$ (7.2 mg, 0.012 mmol), *n*Bu_4NPF_6 (4.6 mg, 0.012 mmol) and allyl TMS (44 μL, 0.277 mmol) in 1,2- DCE (1.15 mL), reacting for 1 hr, then $MeNH_2$ (33wt% in EtOH) (135 μL, 1.16 mmol) in EtOH (2.31 mL) overnight, followed by CbzCl (40 μL, 0.277 mmol) and Et_3N (34 μL, 0.231 mmol) in dry DCM (1.15 mL). Following completion of the Cbz-protection (1 h), purification by FCC (1:12 EtOAc:Hex) afforded the pure compound as a white solid (31 mg, 43% over 3 steps).

RF (1:4 EtOAc:Hex):0.56

^1H NMR (400 MHz, $CDCl_3$) δ 7.45 – 7.11 (m, 7H), 6.98 – 6.82 (m, 2H), 5.79 – 5.54 (m, 2H), 5.19 – 4.90 (m, 5H), 3.84 (s, 3H), 2.64 – 2.47 (m, 2H).

$^{13}C\{H\}$ NMR (101 MHz, $CDCl_3$) δ 156.9, 155.7, 136.7, 134.8, 129.4, 128.5, 128.5, 128.4, 128.2, 128.1, 120.6, 117.5, 110.9, 66.7, 55.3, 52.9, 39.8.

Data in accordance with literature.[10]

benzyl ((5-bromo-1-methyl-1H-indol-3-yl)(phenyl)methyl)carbamate (6f)

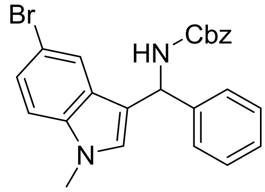

The title compound was prepared according to general procedure **E** from (1,3-dioxoisoindolin-2-yl)(phenyl)methyl acetate (75 mg, 0.254 mmol), $Ca(NTf_2)_2$ (7.6 mg, 0.013 mmol), *n*Bu_4NPF_6 (4.9 mg, 0.013 mmol) and 5-bromo-*N*-methylindole (64 mg, 0.305 mmol) in 1,2- DCE (1.27 mL), reacting for 1 hr, then $MeNH_2$ (33wt% in EtOH) (148 µL, 1.27 mmol) in EtOH (2.54 mL) overnight, followed by CbzCl (79 mg, 0.305 mmol) and Et_3N (35 µL, 0.254 mmol) in dry DCM (1.27 mL). Following completion of the Cbz-protection (1 h), purification by FCC (1:12 to 1:4 EtOAc:Hex) afforded the pure compound as a viscous pale-yellow oil. (36 mg, 32% over 3 steps).

RF (1:4 EtOAc:Hex):0.27

^1H NMR (400 MHz, $CDCl_3$) δ 7.59 (s, 1H), 7.42 – 7.27 (m, 11H), 7.17 – 7.10 (m, 1H), 6.66 – 6.55 (m, 1H), 6.19 (d, *J* = 7.3 Hz, 1H), 5.38 (t, *J* = 15.8 Hz, 1H), 5.13 (q, *J* = 12.3 Hz, 2H), 3.68 – 3.61 (m, 3H).

$^{13}C\{H\}$ NMR (101 MHz, $CDCl_3$) δ 155.6, 141.3, 136.5, 136.2, 129.1, 128.6, 128.6, 128.1, 127.9, 127.5, 126.9, 126.4, 125.0, 122.1, 115.6, 113.0, 111.0, 66.9, 52.1, 33.0.

IR v_{max} (cm^{-1}): 3403, 3311,3028, 2920, 1686, 1492, 1474, 1213, 1025, 695

HRMS (ESI) m/z: [M + K]+ Calcd for $C_{24}H_{21}N_2O_2BrK$ 487.0418; Found 487.0411

N-((5-bromo-1-methyl-1H-indol-3-yl)(phenyl)methyl)acetamide (6g)

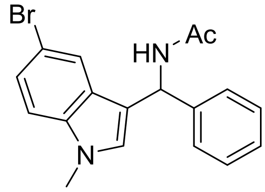

The title compound was prepared according to general procedure **E** from (1,3-dioxoisoindolin-2-yl)(phenyl)methyl acetate (75 mg, 0.254 mmol), $Ca(NTf_2)_2$ (7.6 mg, 0.013 mmol), *n*Bu_4NPF_6 (4.9 mg, 0.013 mmol) and 5-bromo-*N*-methylindole (64 mg, 0.305 mmol) in 1,2- DCE (1.27 mL), reacting for 1 hr, then $MeNH_2$ (33wt% in EtOH) (148 µL, 1.27 mmol) in EtOH (2.54 mL) overnight, followed by AcCl (22 µL, 0.305 mmol) and Et_3N (35 µL, 0.254 mmol) in dry DCM (1.27 mL). Following completion of the Ac-protection (1 h), purification by FCC (1:2 to 1:1 EtOAc:Hex) afforded the pure compound as a white solid. (37 mg, 41% over 3 steps).

RF (1:1 EtOAc:Hex):0.26

^{1}H NMR (400 MHz, DMSO) δ 8.71 (d, *J* = 8.7 Hz, 1H), 7.50 (d, *J* = 1.8 Hz, 1H), 7.43 – 7.32 (m, 5H), 7.30 – 7.24 (m, 2H), 6.99 (s, 1H), 6.31 (d, *J* = 8.6 Hz, 1H), 3.72 (s, 3H), 1.91 (s, 3H).

^{13}C{H} NMR (101 MHz, DMSO) δ 168.8, 142.8, 136.1, 129.9, 128.7, 128.2, 127.47, 127.32, 124.27, 121.8, 116.1, 112.5, 112.0, 49.1, 33.0, 23.0.

Data in accordance with literature.[11]

N-((5-bromo-1-methyl-1H-indol-3-yl)(phenyl)methyl)benzamide (6h)

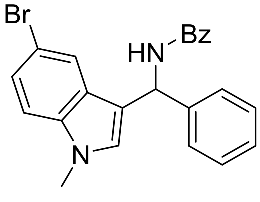

The title compound was prepared according to general procedure **E** from (1,3-dioxoisoindolin-2-yl)(phenyl)methyl acetate (75 mg, 0.254 mmol), $Ca(NTf_2)_2$ (7.6 mg, 0.013 mmol), *n*Bu_4NPF_6 (4.9 mg, 0.013 mmol) and 5-bromo-*N*-methylindole (64 mg, 0.305 mmol) in 1,2- DCE (1.27 mL), reacting for 1 hr, then $MeNH_2$ (33wt% in EtOH) (148 µL, 1.27 mmol) in EtOH (2.54 mL) overnight, followed by BzCl (35 µL, 0.305 mmol) and Et_3N (35 µL, 0.254 mmol) in dry DCM (1.27 mL). Following completion of the Bz-protection (1 h), purification by FCC (1:4 to 1:2 EtOAc:Hex) afforded the pure compound as a white solid. (73 mg, 69% over 3 steps).

RF (1:4 EtOAc:Hex):0.15

^{1}H NMR (400 MHz, DMSO) δ 9.24 (d, *J* = 8.6 Hz, 1H), 7.98 – 7.88 (m, 2H), 7.59 – 7.54 (m, 1H), 7.54 – 7.34 (m, 8H), 7.33 – 7.24 (m, 2H), 6.99 (s, 1H), 6.62 (d, *J* = 8.6 Hz, 1H), 3.73 (s, 3H).

^{13}C{H} NMR (101 MHz, DMSO) δ 166.2, 142.6, 136.1, 134.9, 131.7, 130.3, 128.8, 128.7, 128.5, 128.0, 127.8, 127.5, 124.3, 121.7, 115.8, 112.5, 112.1, 49.8, 33.0.

Data in accordance with literature.[11]

allyl ((5-bromo-1-methyl-1H-indol-3-yl)(phenyl)methyl)carbamate (6i)

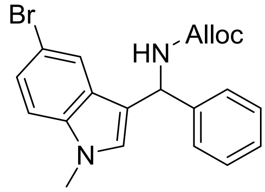

The title compound was prepared according to general procedure **E** from (1,3-dioxoisoindolin-2-yl)(phenyl)methyl acetate (75 mg, 0.254 mmol), $Ca(NTf_2)_2$ (7.6 mg, 0.013 mmol), *n*Bu_4NPF_6 (4.9 mg, 0.013 mmol) and 5-bromo-*N*-methylindole (64 mg, 0.305 mmol) in 1,2- DCE (1.27 mL), reacting for 1 hr, then $MeNH_2$ (33wt% in EtOH) (148 µL, 1.27 mmol) in EtOH (2.54 mL) overnight, followed by AllocCl (35 µL, 0.305 mmol) and Et_3N (35 µL, 0.254 mmol) in dry DCM (1.27 mL). Following completion of the Alloc-protection (1 h), purification by FCC (1:8 to 1:4 EtOAc:Hex) afforded the pure compound as a viscous pale-yellow oil. (36 mg, 35% over 3 steps).

RF (1:4 EtOAc:Hex):0.35

^{1}H NMR (400 MHz, $CDCl_3$) δ 7.64 – 7.57 (m, 1H), 7.39 – 7.28 (m, 6H), 7.16 – 7.12 (m, 1H), 6.66 – 6.57 (m, 1H), 6.17 (d, *J* = 7.7 Hz, 1H), 5.98 – 5.85 (m, 1H), 5.43 – 5.08 (m, 3H), 4.67 – 4.53 (m, 2H), 3.66 (s, 3H).

^{13}C{H} NMR (101 MHz, $CDCl_3$) δ 155.5, 141.4, 136.2, 132.8, 129.1, 128.6, 127.9, 127.5, 126.8, 125.0, 122.1, 117.8, 115.6, 113.0, 111.0, 65.8, 52.1, 32.9.

IR ν_{max} (cm^{-1}): 3291, 3060, 2924, 1679, 1533, 1474, 1246, 1034, 767, 701

HRMS (ESI) m/z: [M – AllocNH]+ Calcd for $C_{16}H_{13}N^{81}Br$ 300.0205; Found 300.0214

References

1. R. N. Enright, J. L. Grinde, L. I. Wurtz, M. S. Paeth, T. R. Wittman, E. R. Cliff, Y. T. Sankari, L. T. Henningsen, C. Tan, J. D. Scanlon and P. H. Willoughby, *Tetrahedron*, 2016, **72**, 6397-6408.
2. S.-D. Cho, H.-J. Kim, C. Ahn, J. R. Falck and D.-S. Shin, *Tetrahedron Lett.*, 1999, **40**, 8215-8217.
3. K. Indukuri, R. Unnava, M. J. Deka and A. K. Saikia, *J. Org. Chem.*, 2013, **78**, 10629-10641.
4. D. Ghosh , S. Saravanan, N. Gupta, S. H. R. Abdi, N.-u. H. Khan, R. I. Kureshy and H. C. Bajaj, *Asian. J. Org. Chem.*, 2014, **3**, 1173-1181.
5. T. Vilaivan, C. Winotapan, V. Banphavichit, T. Shinada and Y. Ohfune, *J. Org. Chem.*, 2005, **70**, 3464-3471.
6. P.-O. Delaye, M. h. Ahari, J.-L. Vasse and J. Szymoniak, *Tetrahedron: Asymmetry*, 2010, **21**, 2505-2511.
7. B. Peng, J. Ma, J. Guo, Y. Gong, R. Wang, Y. Zhang, J. Zeng, W.-W. Chen, K. Ding and B. Zhao, *JACS*, 2022, **144**, 2853-2860.

8. X. Peng, K.-H. Wang, D. Huang, J. Wang, Y. Wang, Y. Su, Y. Hu and Y. Fu, *Appl. Organomet. Chem.*, 2017, **31**, e3731.
9. T. Ollevier and Z. Li, *Adv. Synth. Catal.*, 2009, **351**, 3251-3259.
10. Q.-Y. Song, B.-L. Yang and S.-K. Tian, *J. Org. Chem.*, 2007, **72**, 5407-5410.
11. Z.-J. Yi, J.-T. Sun, T.-Y. Yang, X.-Y. Yu, X.-L. Han and B.-G. Wei, *Org. Biomol. Chem.*, 2022, **20**, 2261-2270.

Copies of ^{1}H and ^{13}C NMR Spectra

(1,3-dioxoisoindolin-2-yl)(phenyl)methyl acetate (1a)

^{1}H NMR (400 MHz, $CDCl_3$)

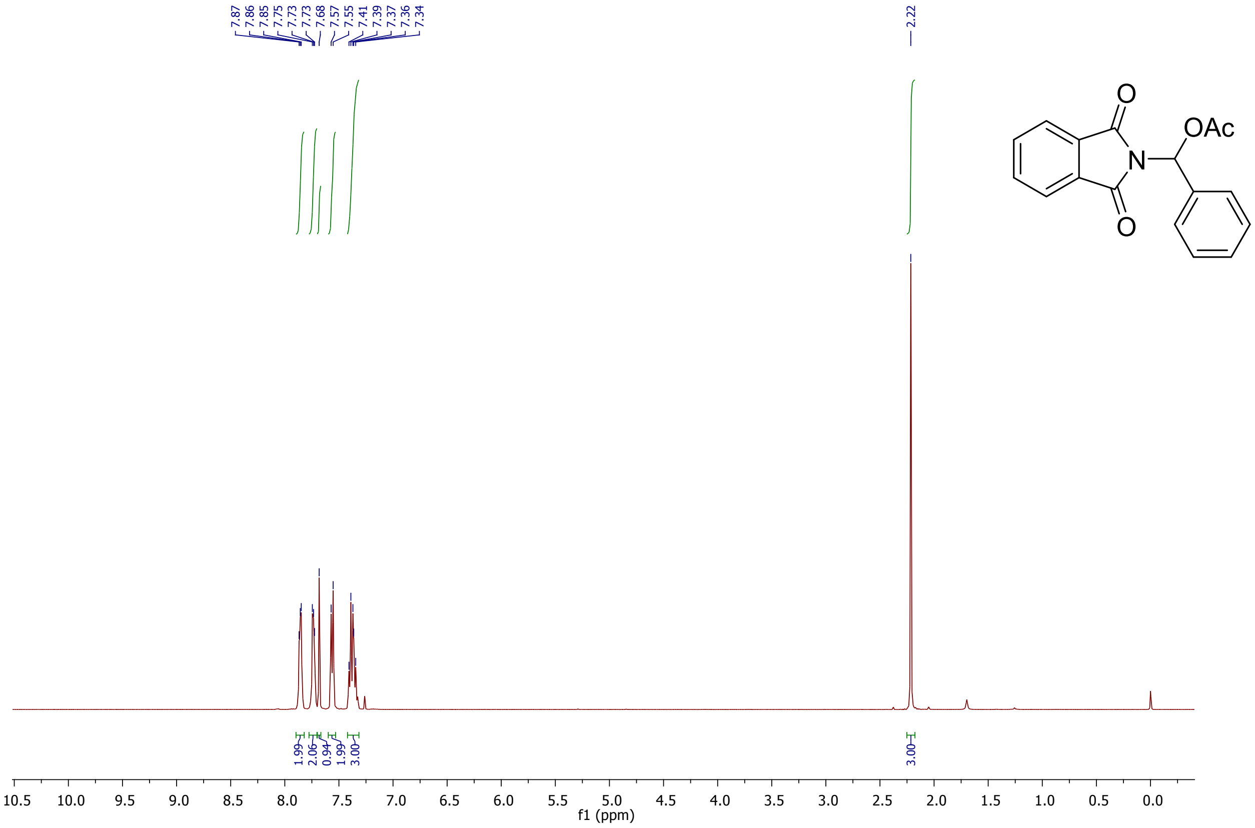

^{13}C{^{1}H} NMR (101 MHz, $CDCl_3$)

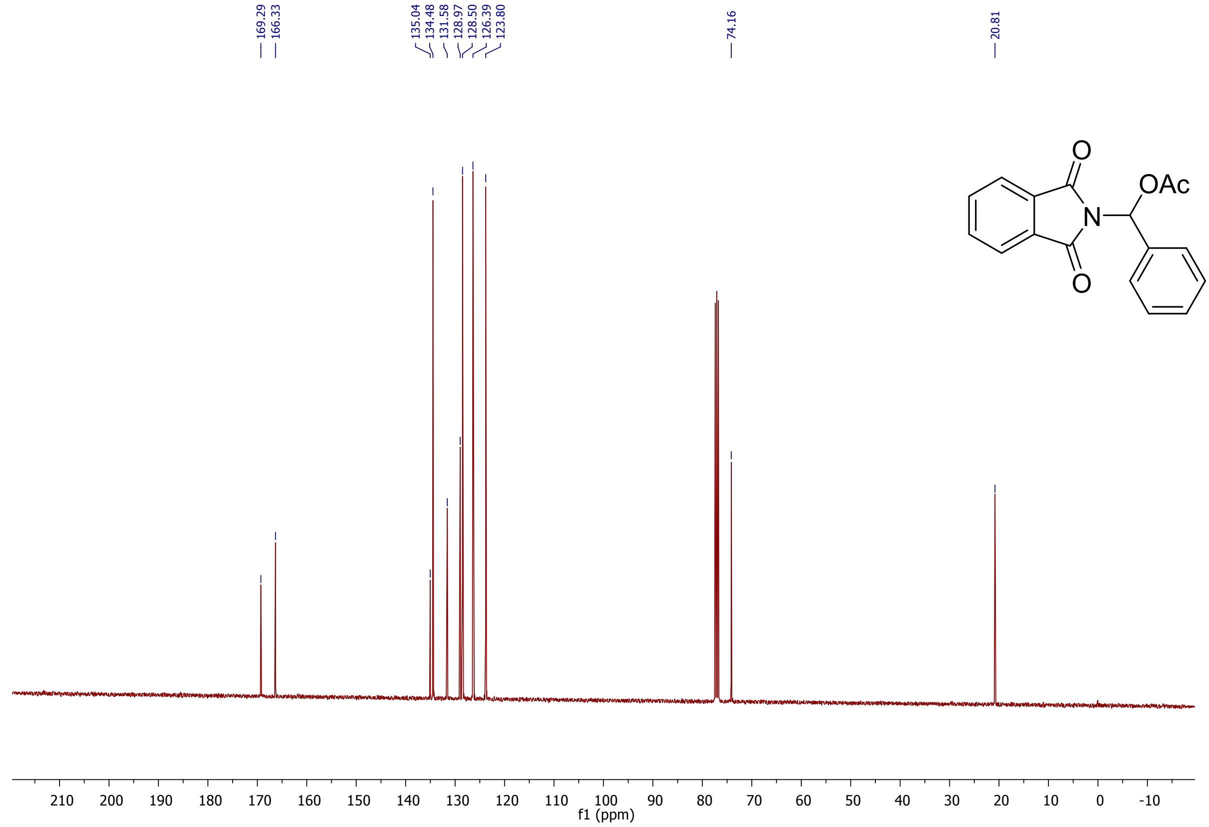

(1,3-dioxoisoindolin-2-yl)(4-methoxyphenyl)methyl acetate (1b)

^{1}H NMR (400 MHz, $CDCl_3$)

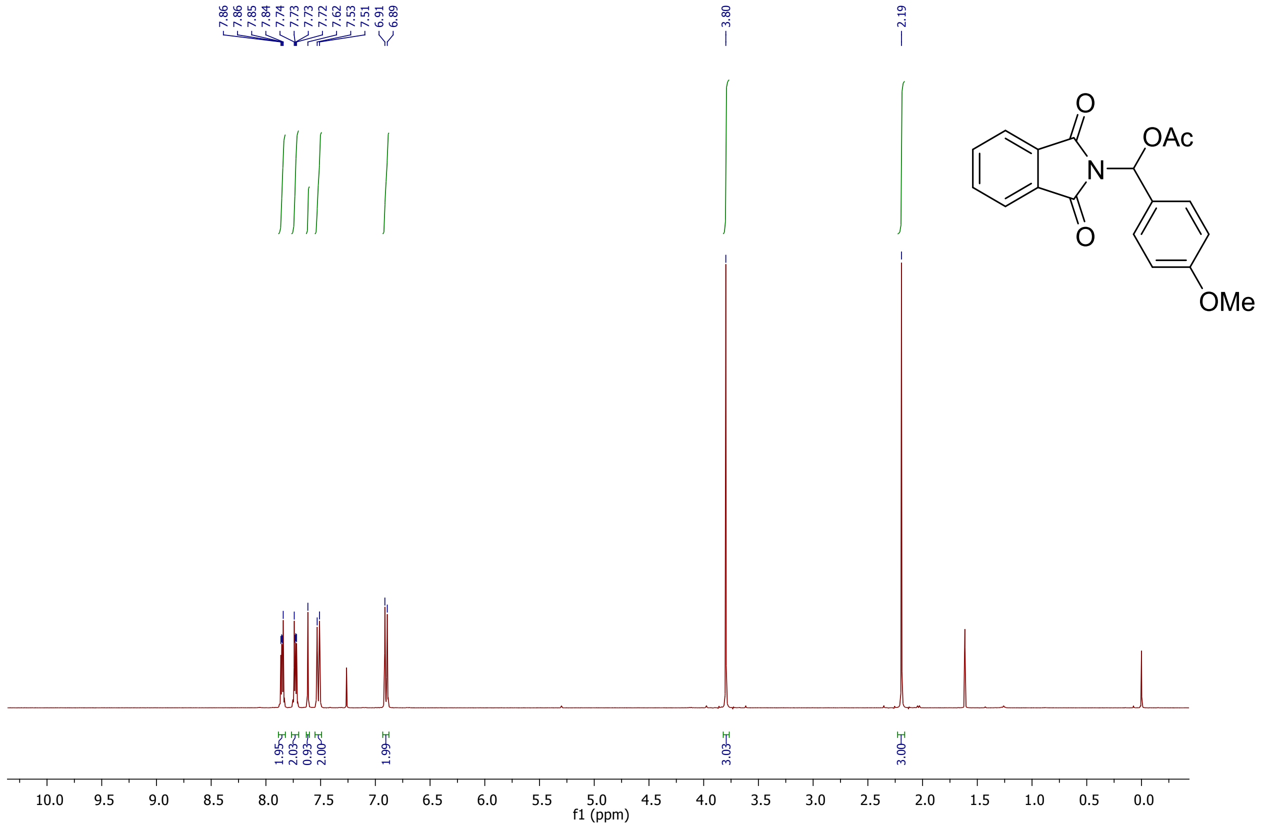

$^{13}C\{^{1}H\}$ NMR (101 MHz, $CDCl_3$)

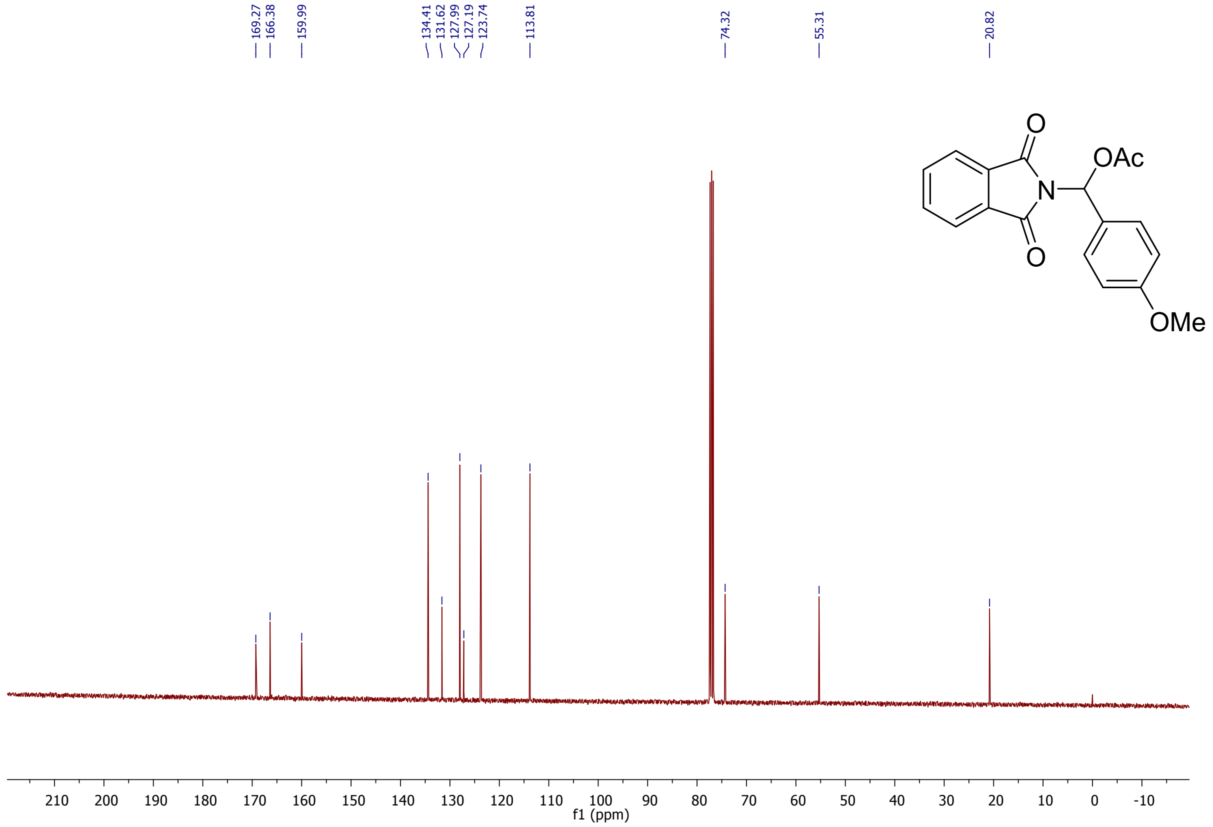

(1,3-dioxoisoindolin-2-yl)(p-tolyl)methyl acetate (1c)

^{1}H NMR (400 MHz, $CDCl_3$)

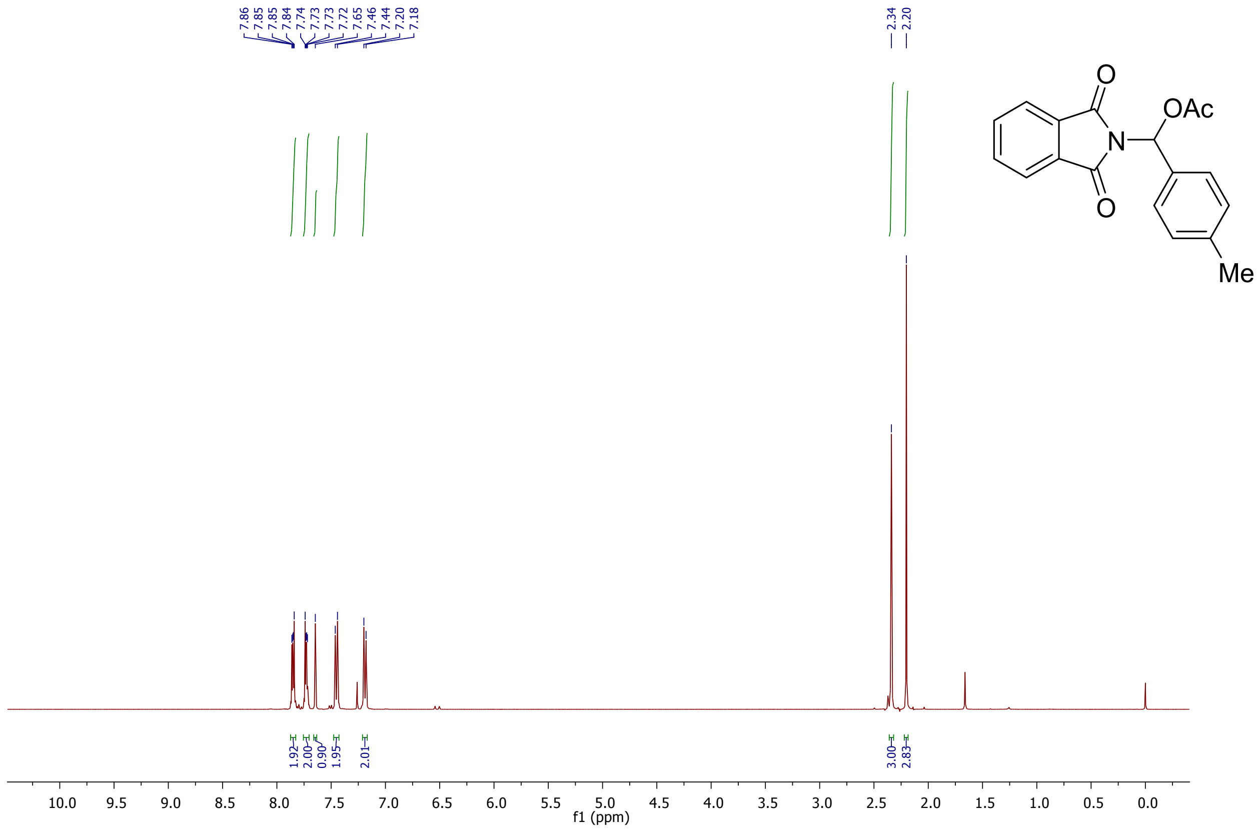

$^{13}C\{^{1}H\}$ NMR (101 MHz, $CDCl_3$)

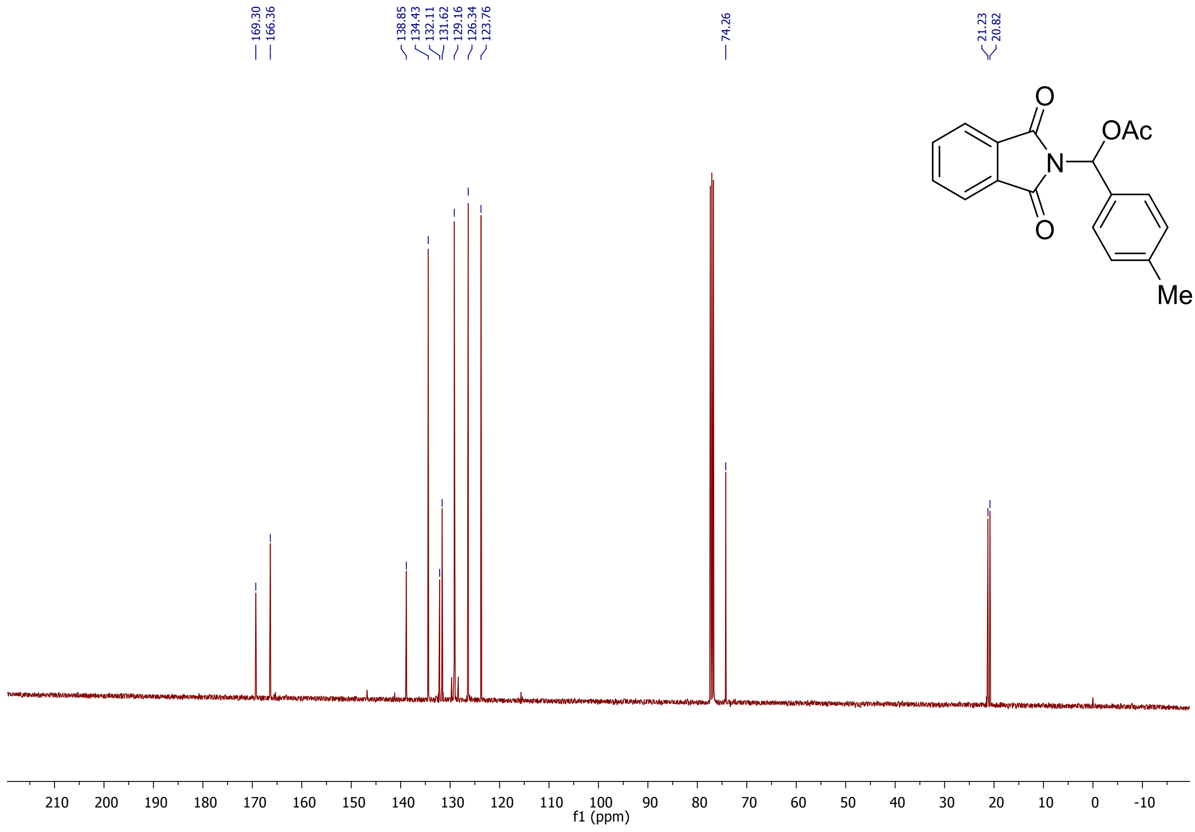

(1,3-dioxoisoindolin-2-yl)(4-fluorophenyl)methyl acetate (1d)

^{1}H NMR (400 MHz, $CDCl_3$)

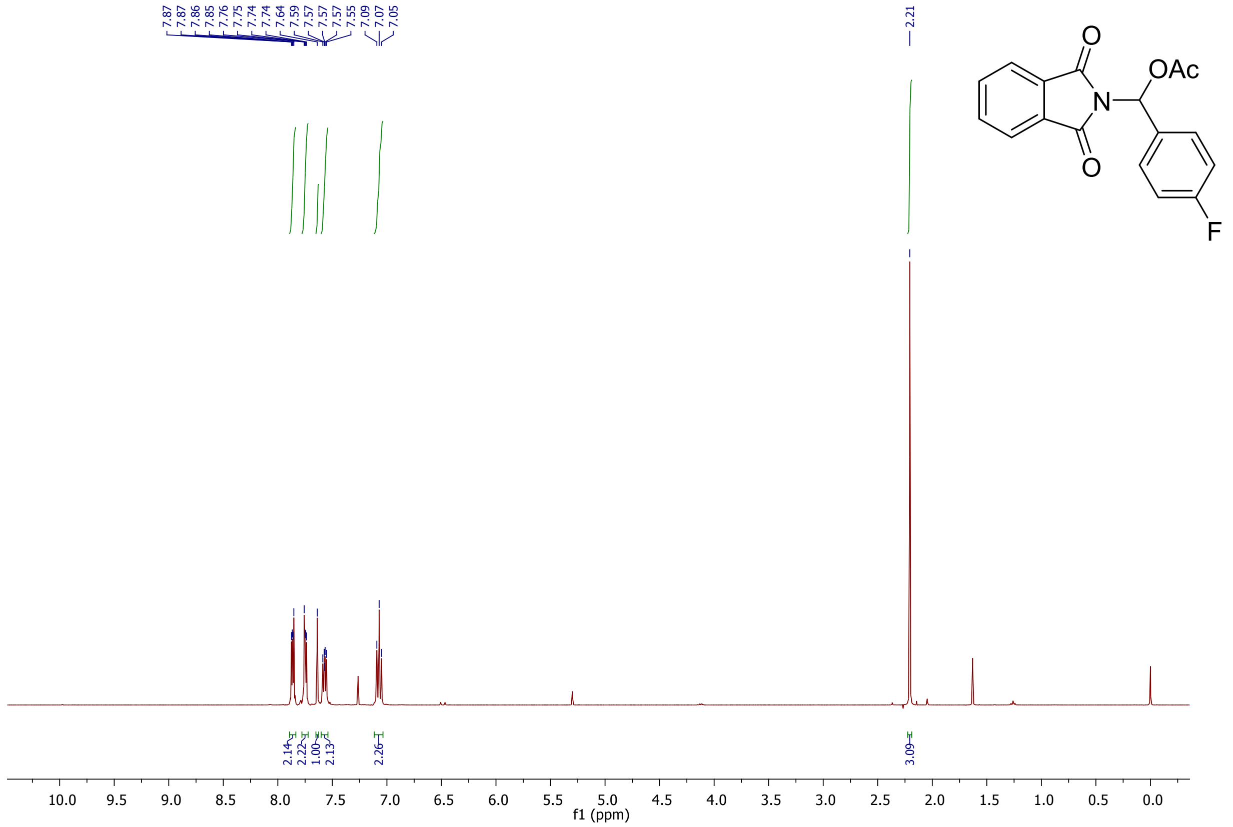

$^{13}C\{^{1}H\}$ NMR (101 MHz, $CDCl_3$)

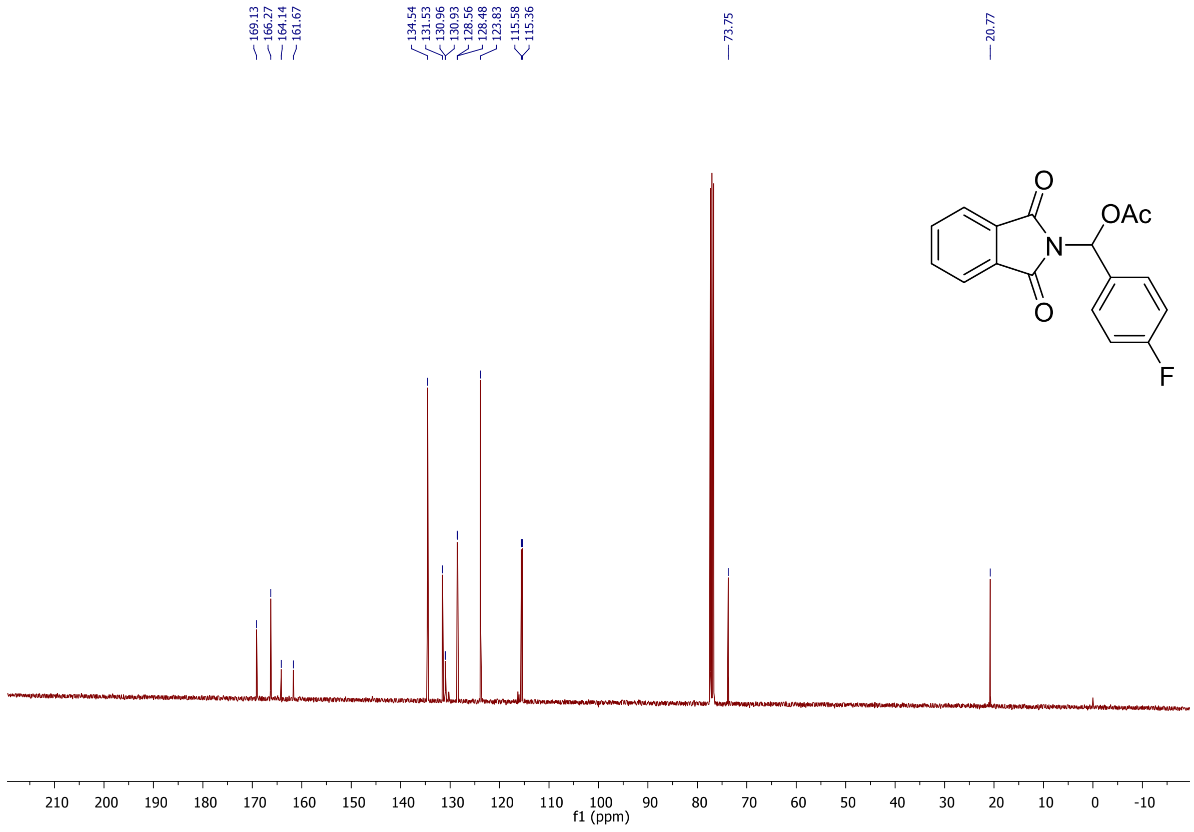

(1,3-dioxoisoindolin-2-yl)(4-(trifluoromethyl)phenyl)methyl acetate (1e)

^{1}H NMR (400 MHz, CDCl$_3$)

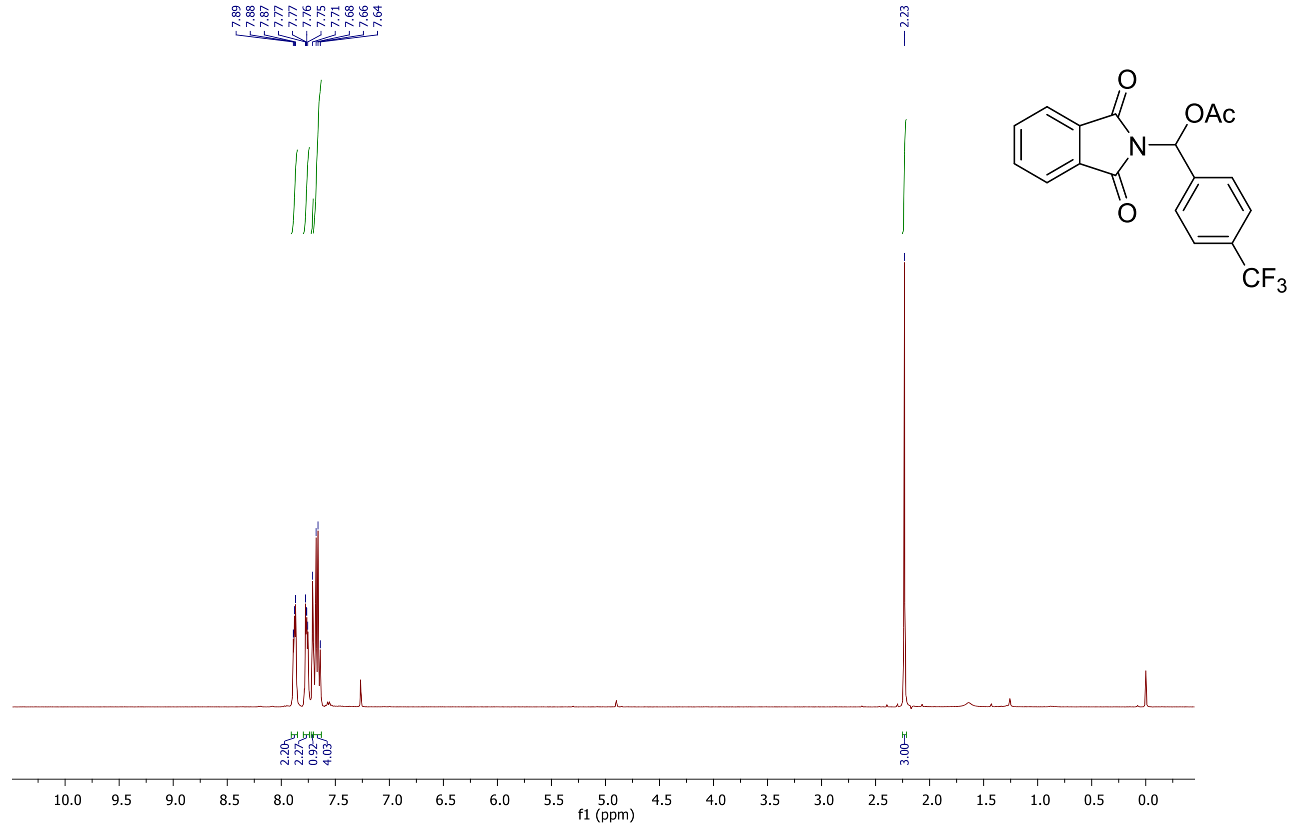

^{13}C{^{1}H} NMR (101 MHz, CDCl$_3$)

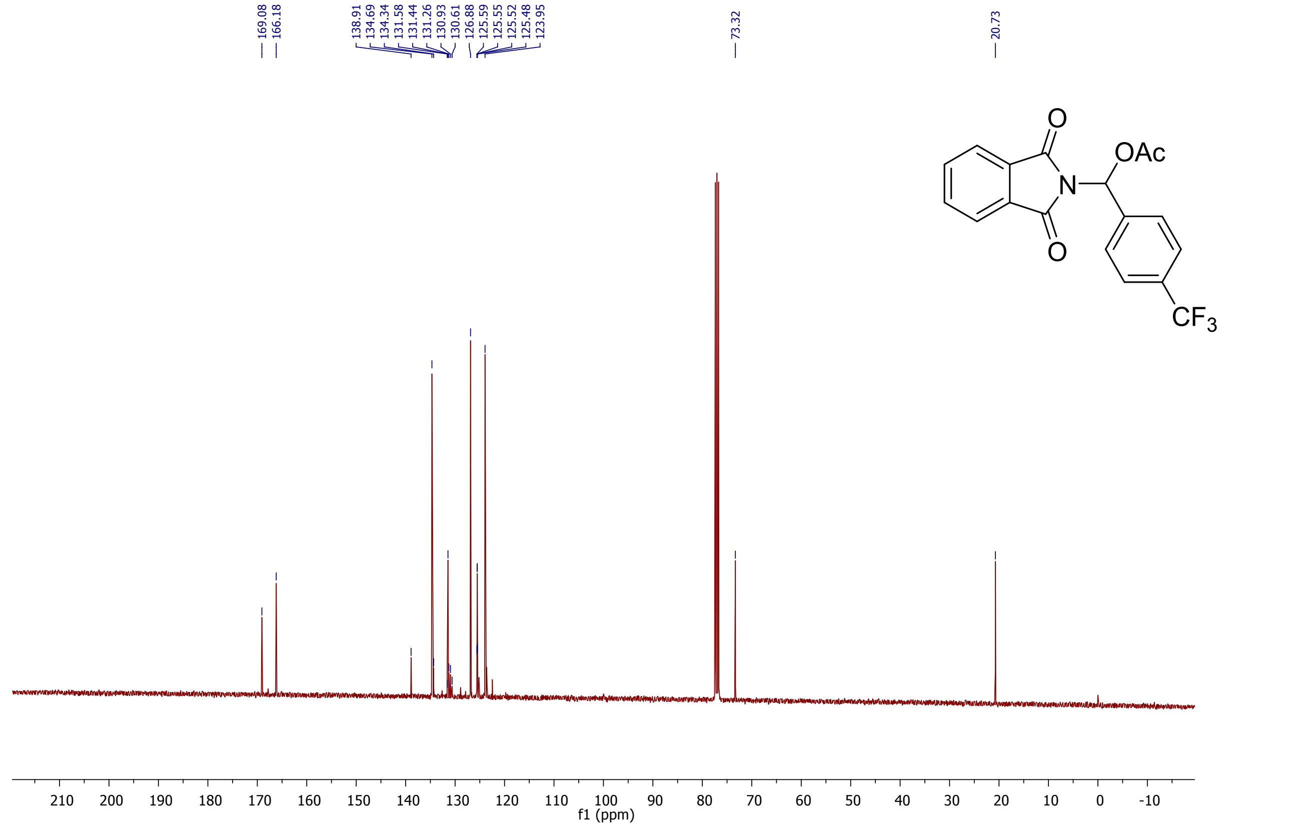

(1,3-dioxoisoindolin-2-yl)(2-methoxyphenyl)methyl acetate (1f)

^{1}H NMR (400 MHz, $CDCl_3$)

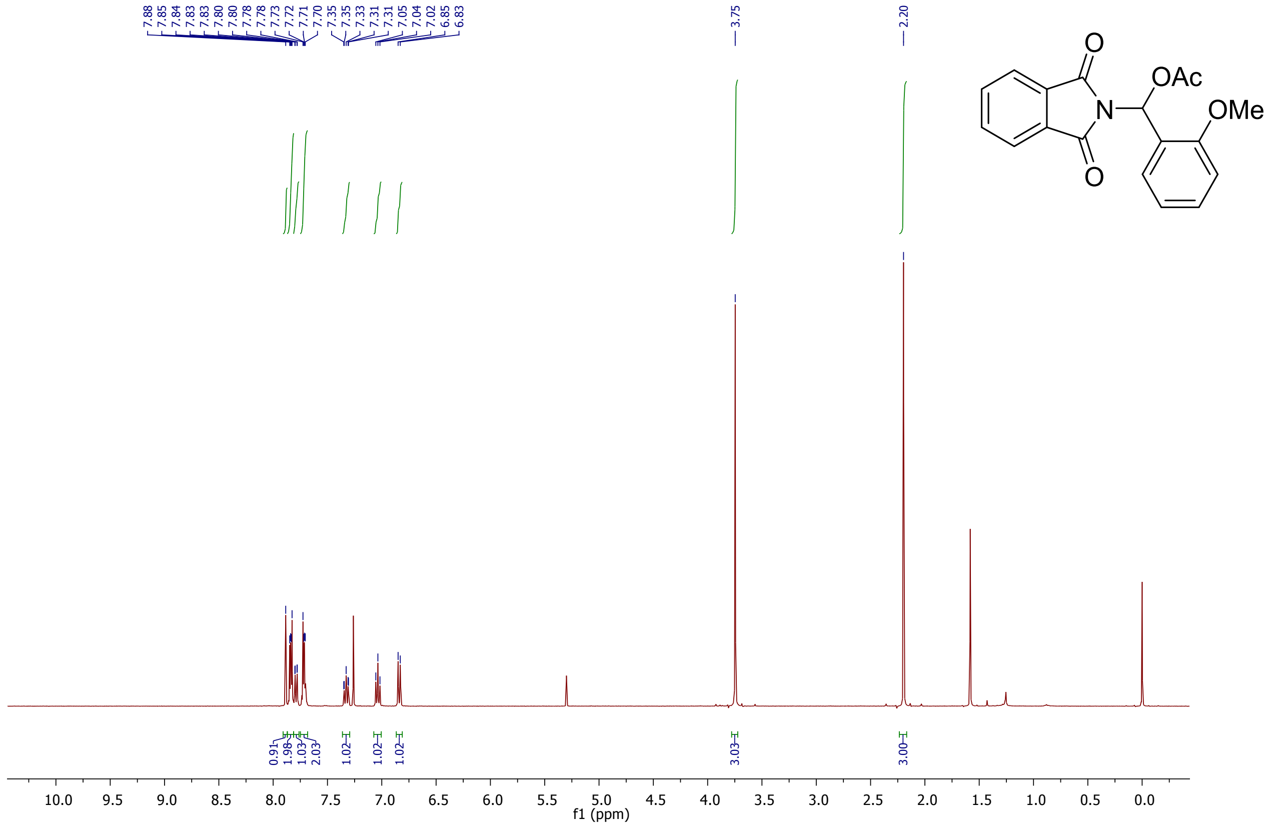

^{13}C{^{1}H} NMR (101 MHz, $CDCl_3$)

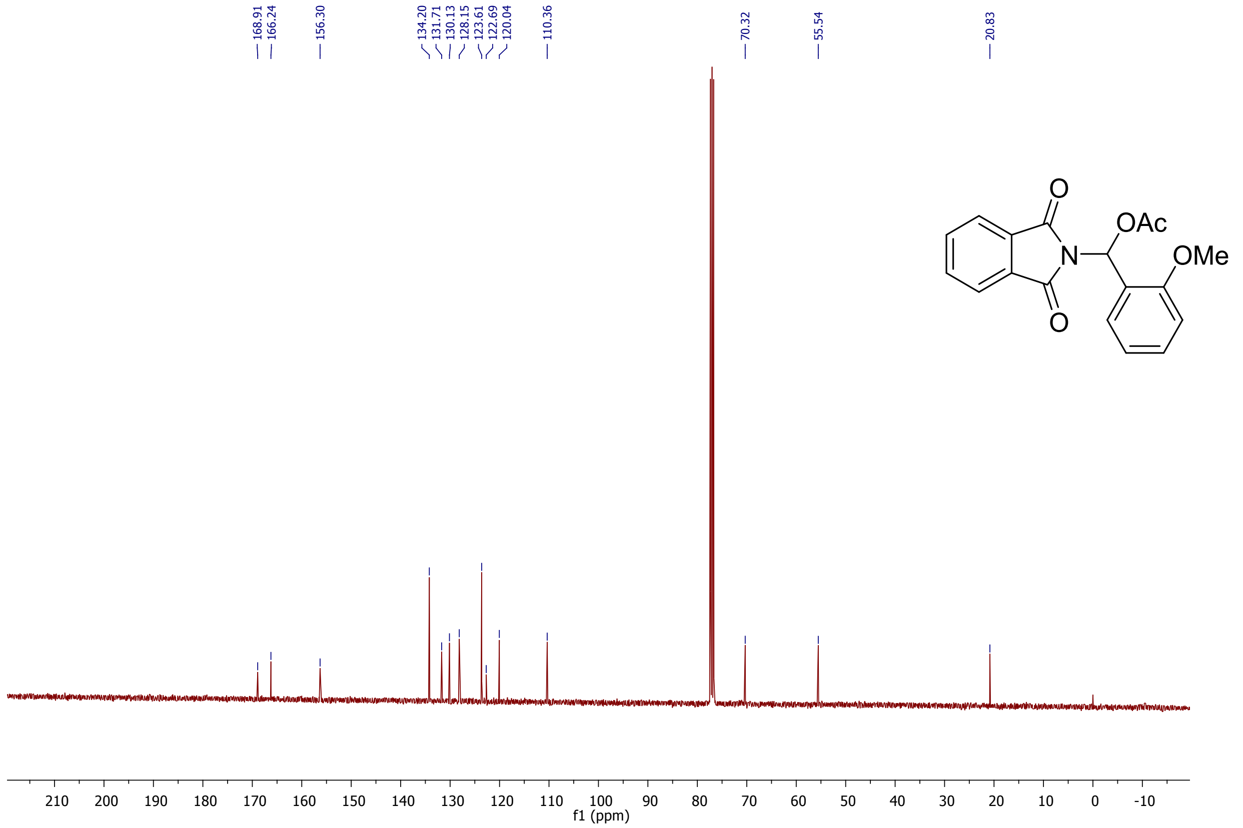

(2-bromophenyl)(1,3-dioxoisoindolin-2-yl)methyl acetate (1g)

^{1}H NMR (400 MHz, $CDCl_3$)

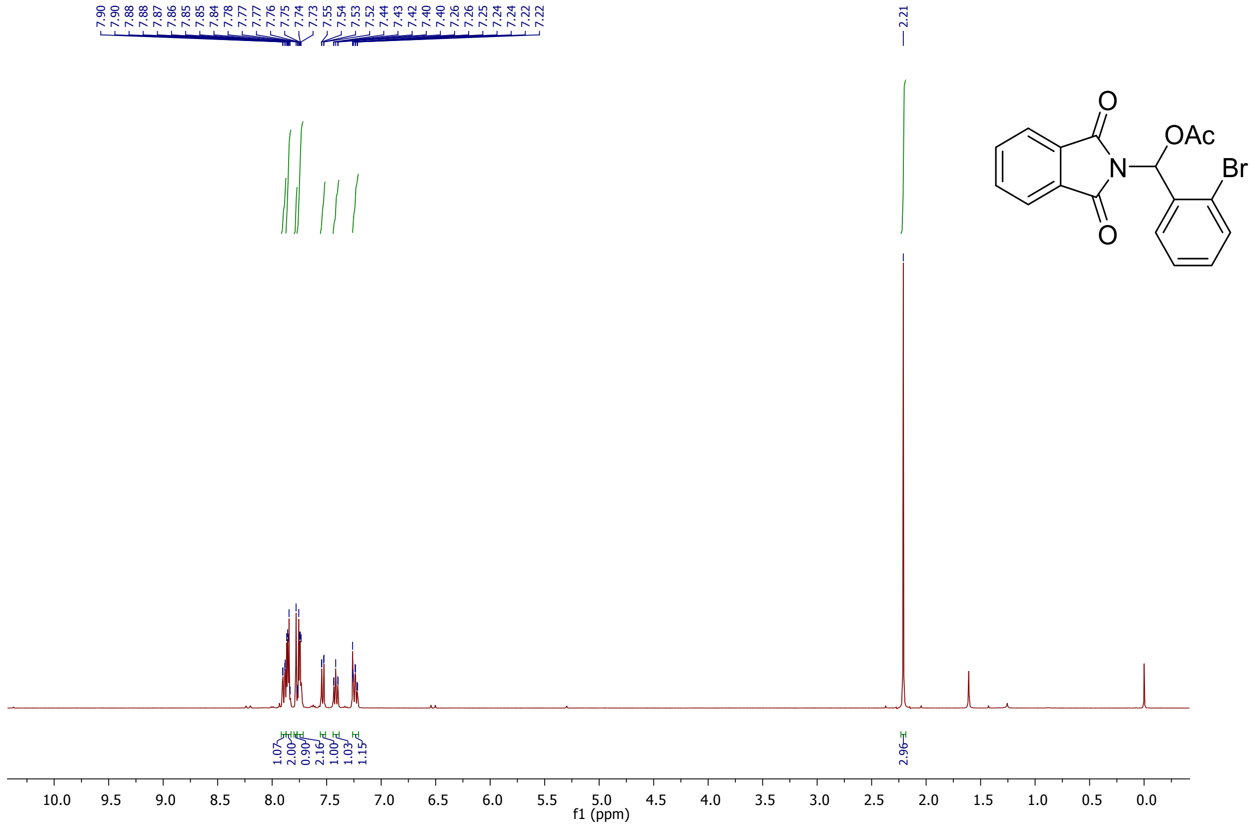

$^{13}C\{^{1}H\}$ NMR (101 MHz, $CDCl_3$)

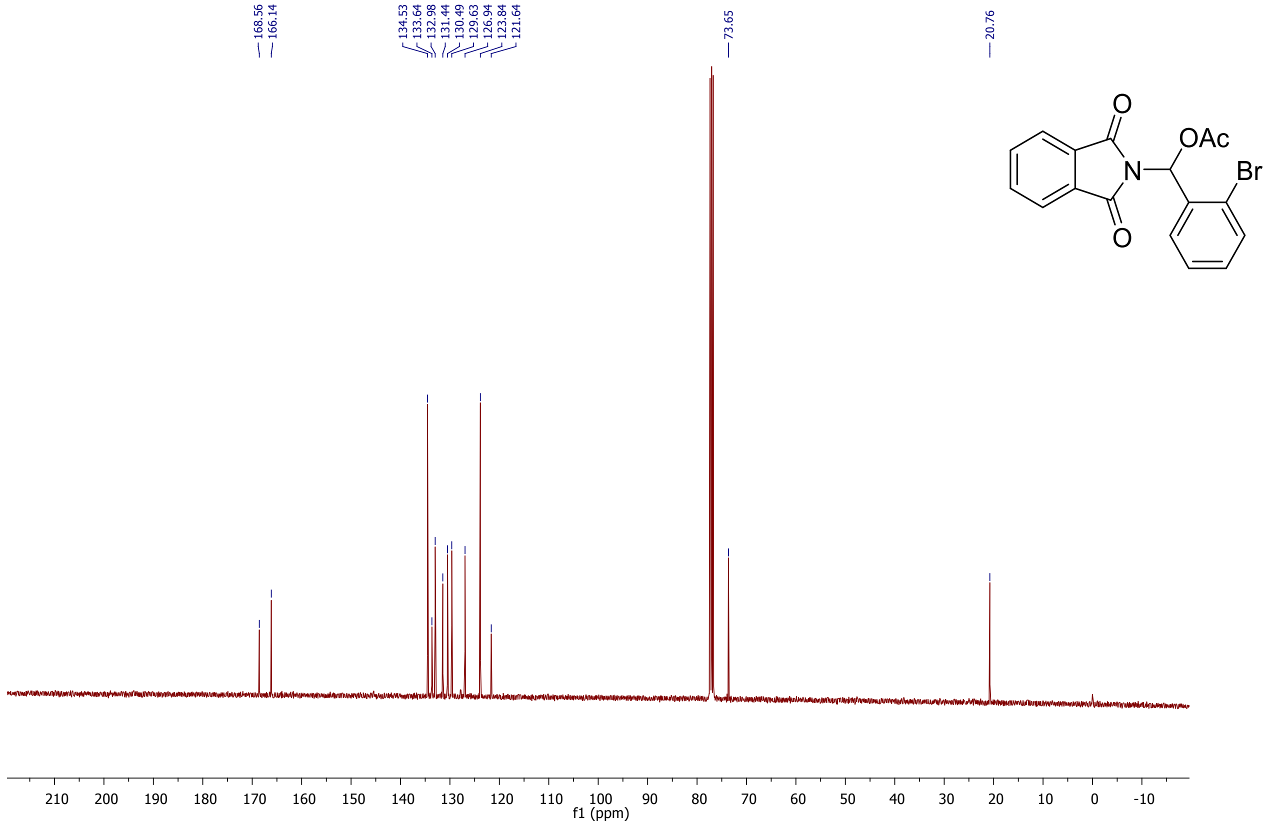

(3-chlorophenyl)(1,3-dioxoisoindolin-2-yl)methyl acetate (1h)

^{1}H NMR (400 MHz, $CDCl_3$)

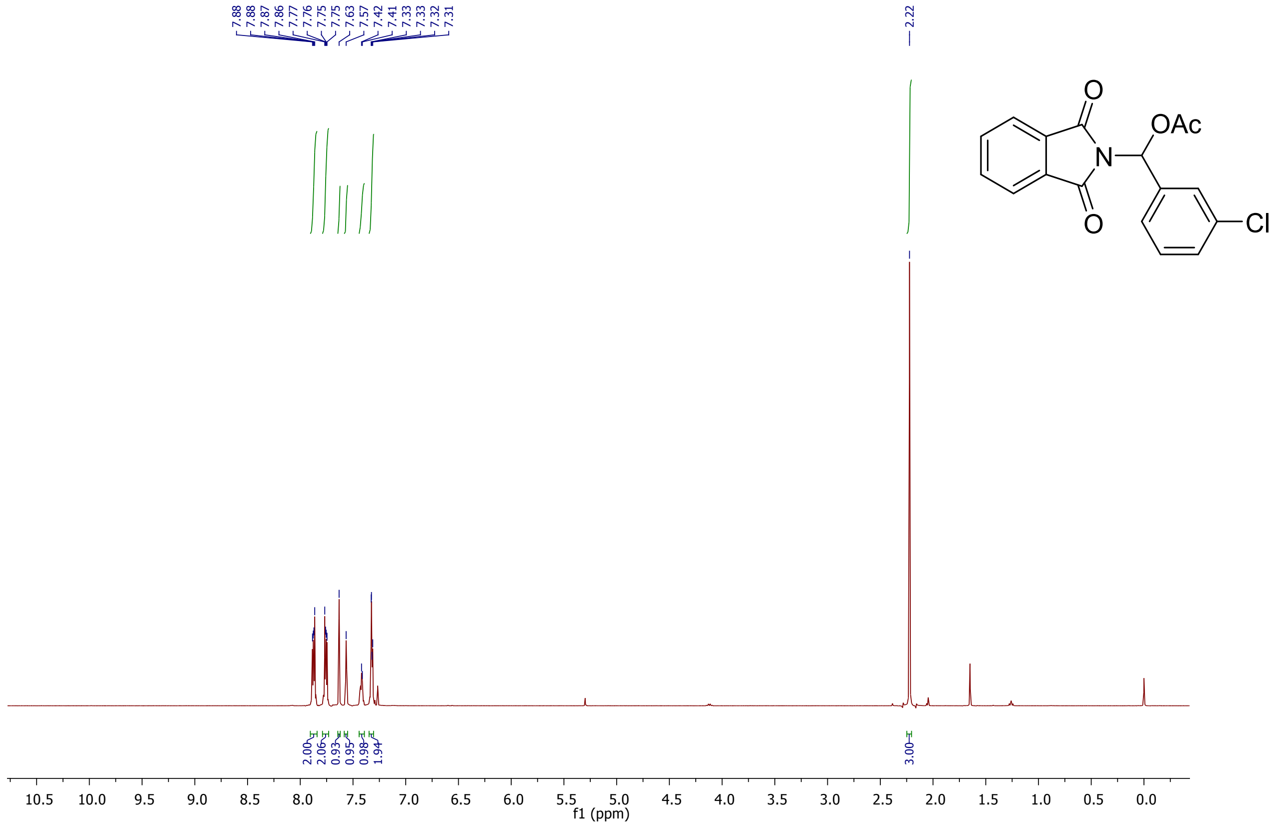

$^{13}C\{^{1}H\}$ NMR (101 MHz, $CDCl_3$)

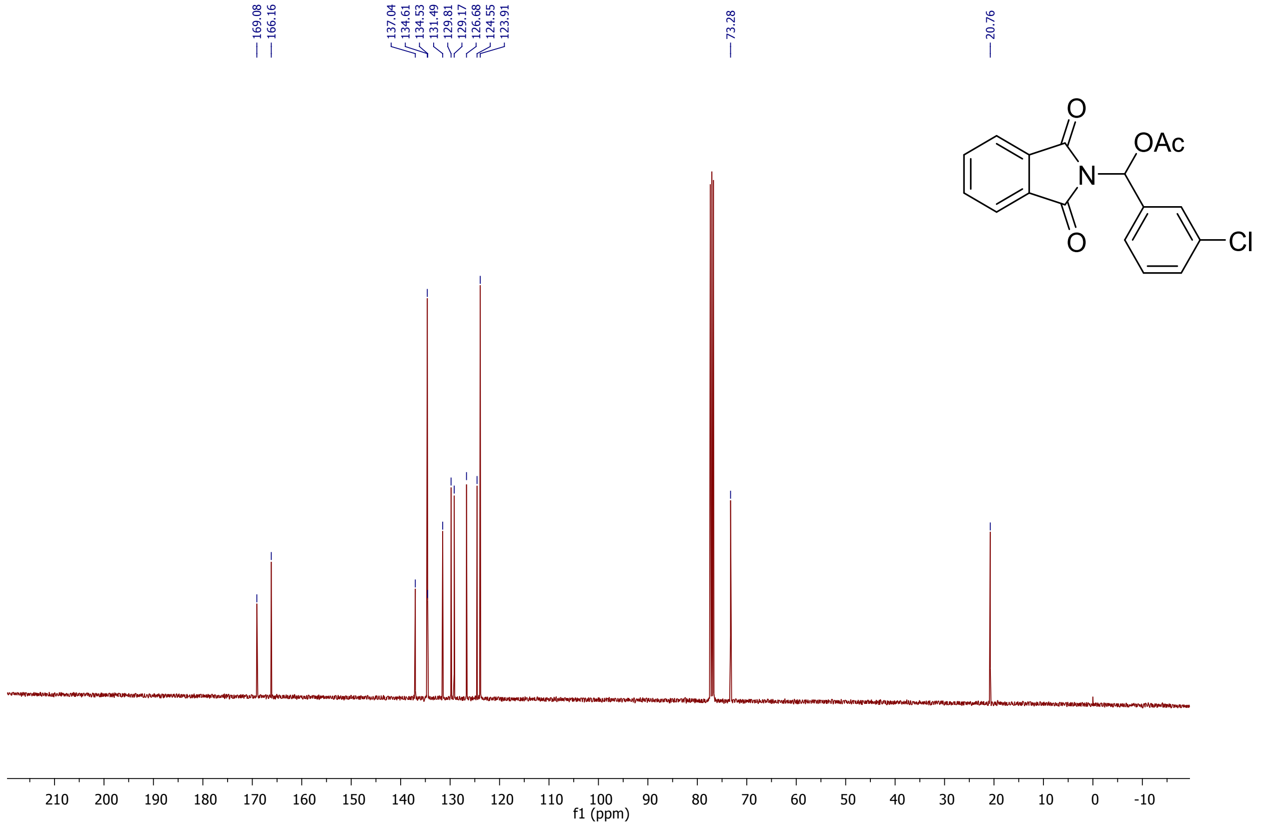

(3-bromo-2-fluorophenyl)(1,3-dioxoisoindolin-2-yl)methyl acetate (1i)

^{1}H NMR (400 MHz, $CDCl_3$)

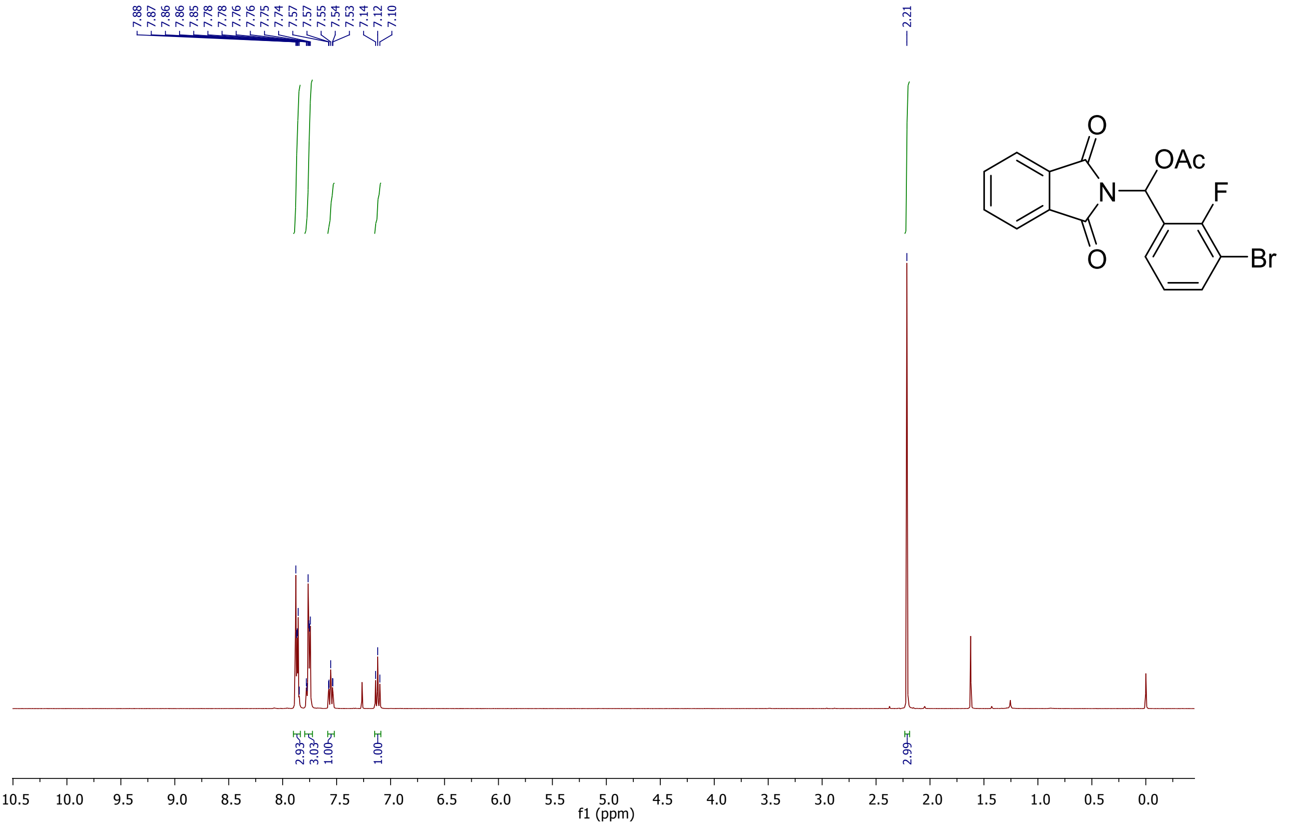

$^{13}C\{^{1}H\}$ NMR (101 MHz, $CDCl_3$)

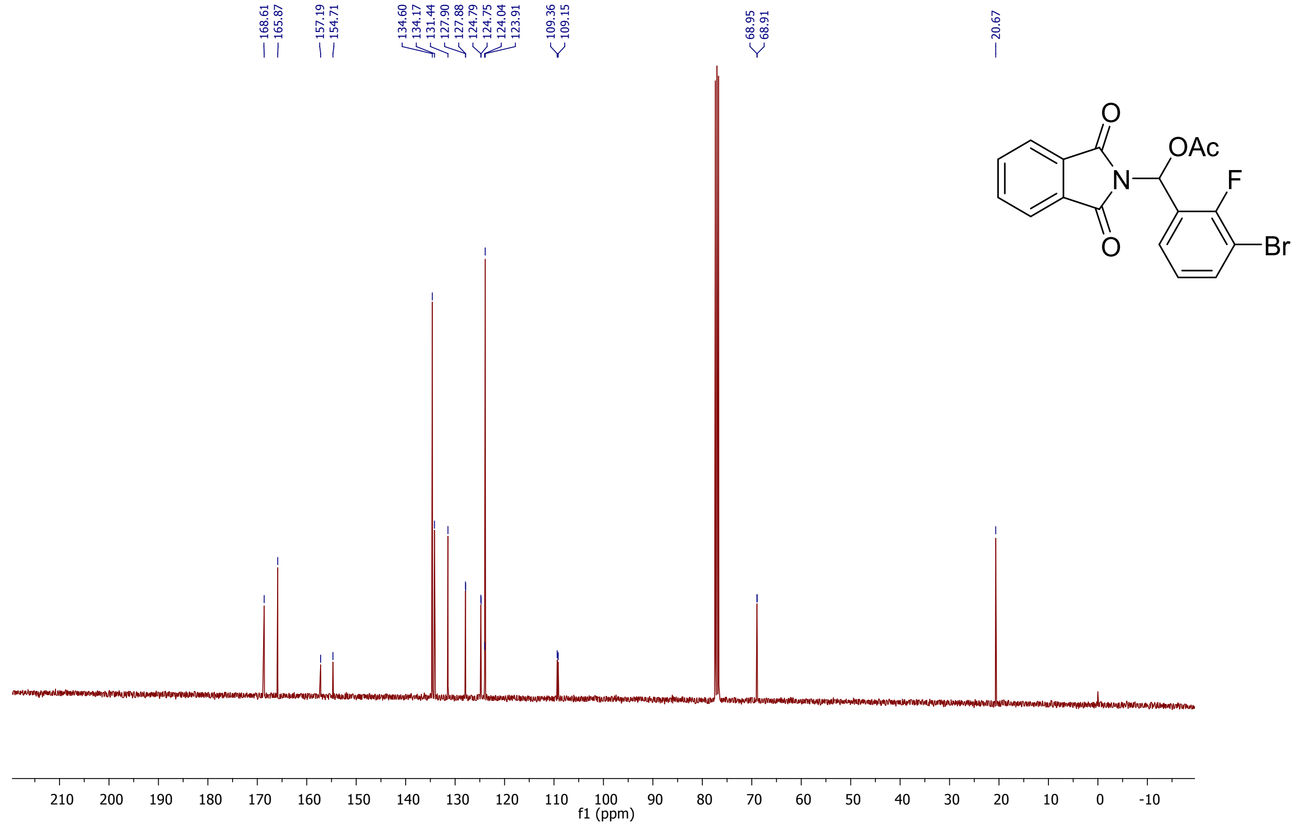

(3,5-bis(trifluoromethyl)phenyl)(1,3-dioxoisoindolin-2-yl)methyl acetate (1j)

^{1}H NMR (400 MHz, $CDCl_3$)

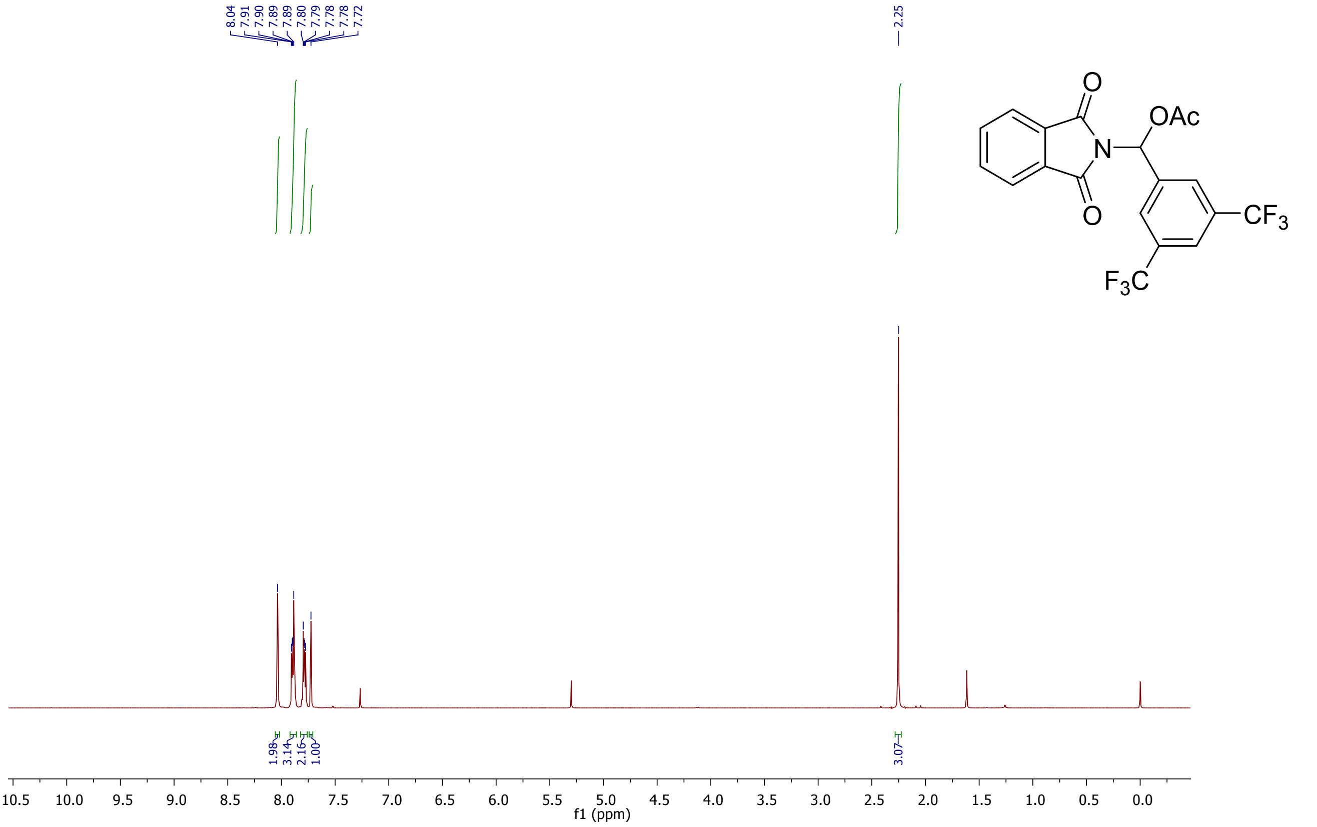

$^{13}C\{^{1}H\}$ NMR (101 MHz, $CDCl_3$)

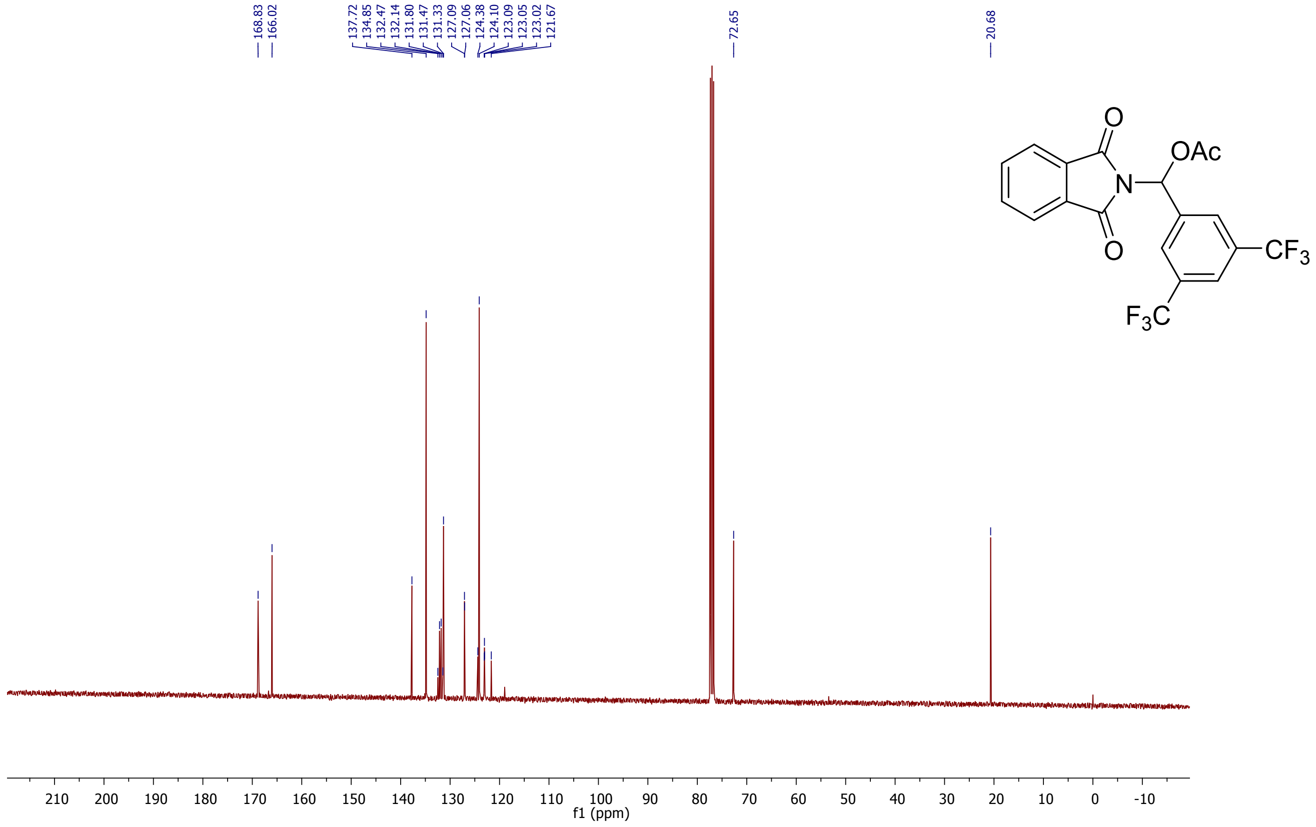

(1,3-dioxoisoindolin-2-yl)(4-nitrophenyl)methyl acetate (1k)

^{1}H NMR (400 MHz, $CDCl_3$)

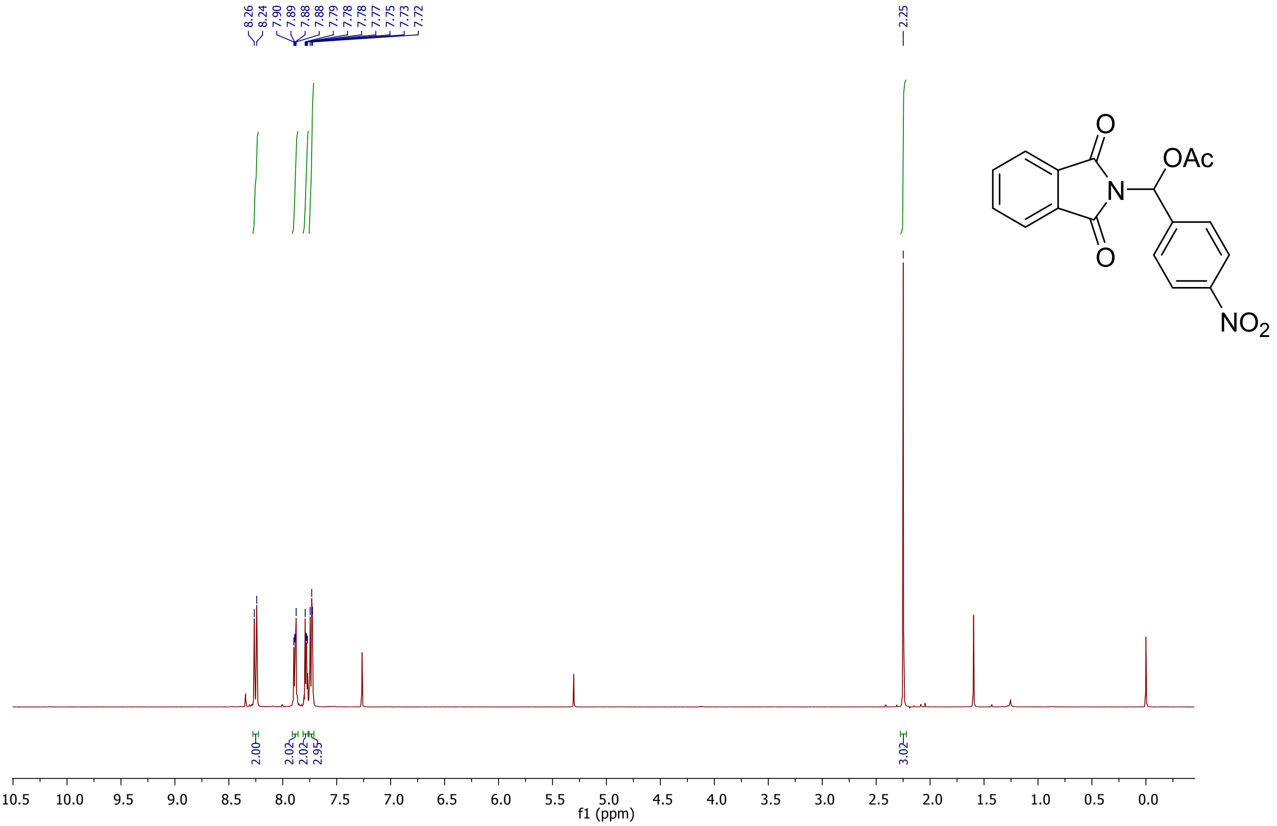

^{13}C{^{1}H} NMR (101 MHz, $CDCl_3$)

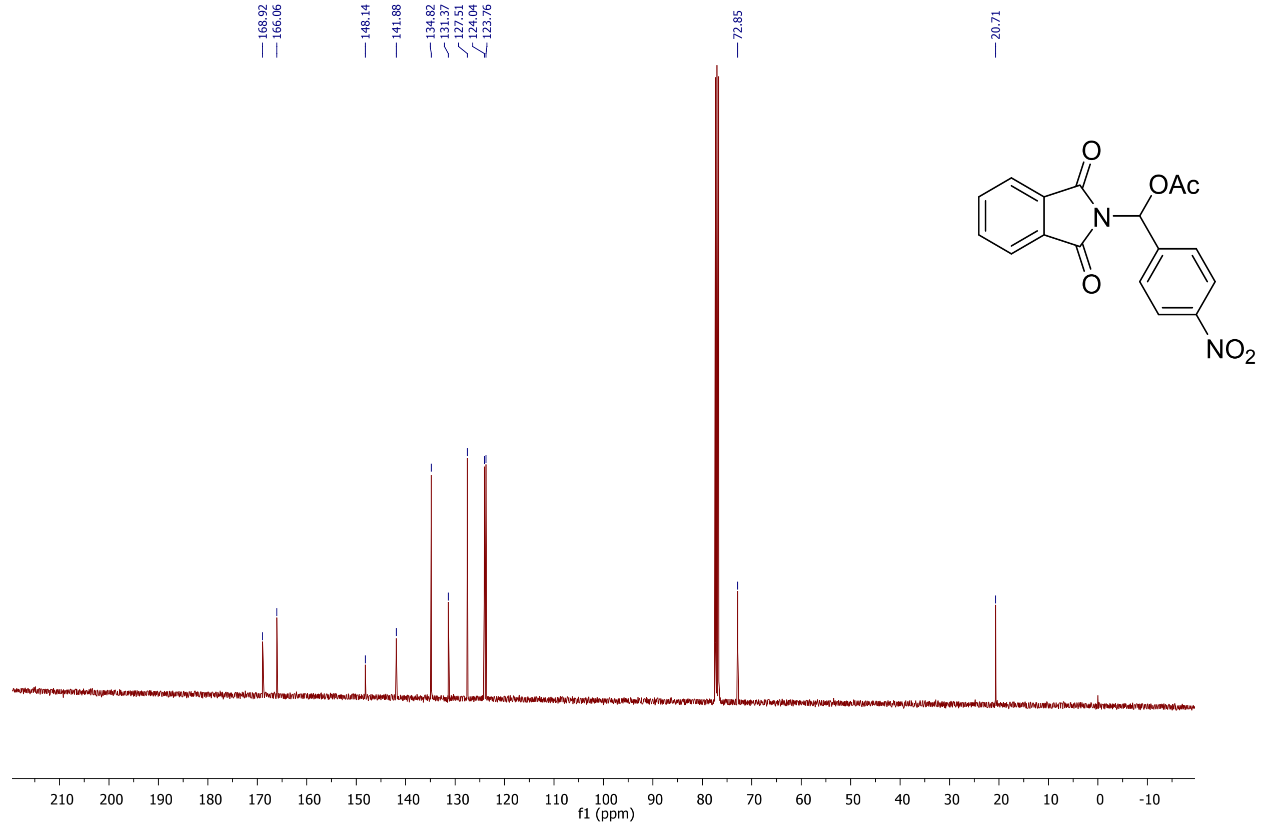

(1,3-dioxoisoindolin-2-yl)(naphthalen-2-yl)methyl acetate (1l)

^{1}H NMR (400 MHz, $CDCl_3$)

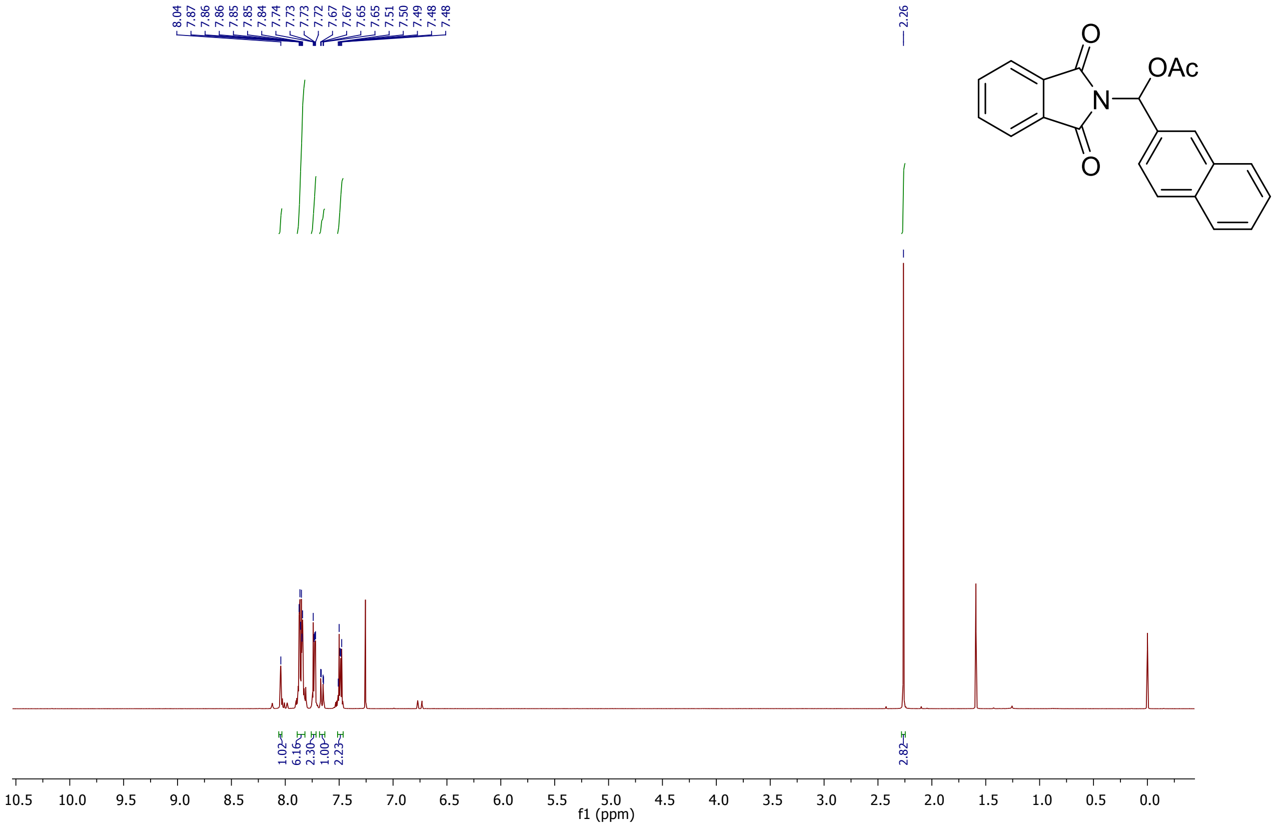

$^{13}C\{^{1}H\}$ NMR (101 MHz, $CDCl_3$)

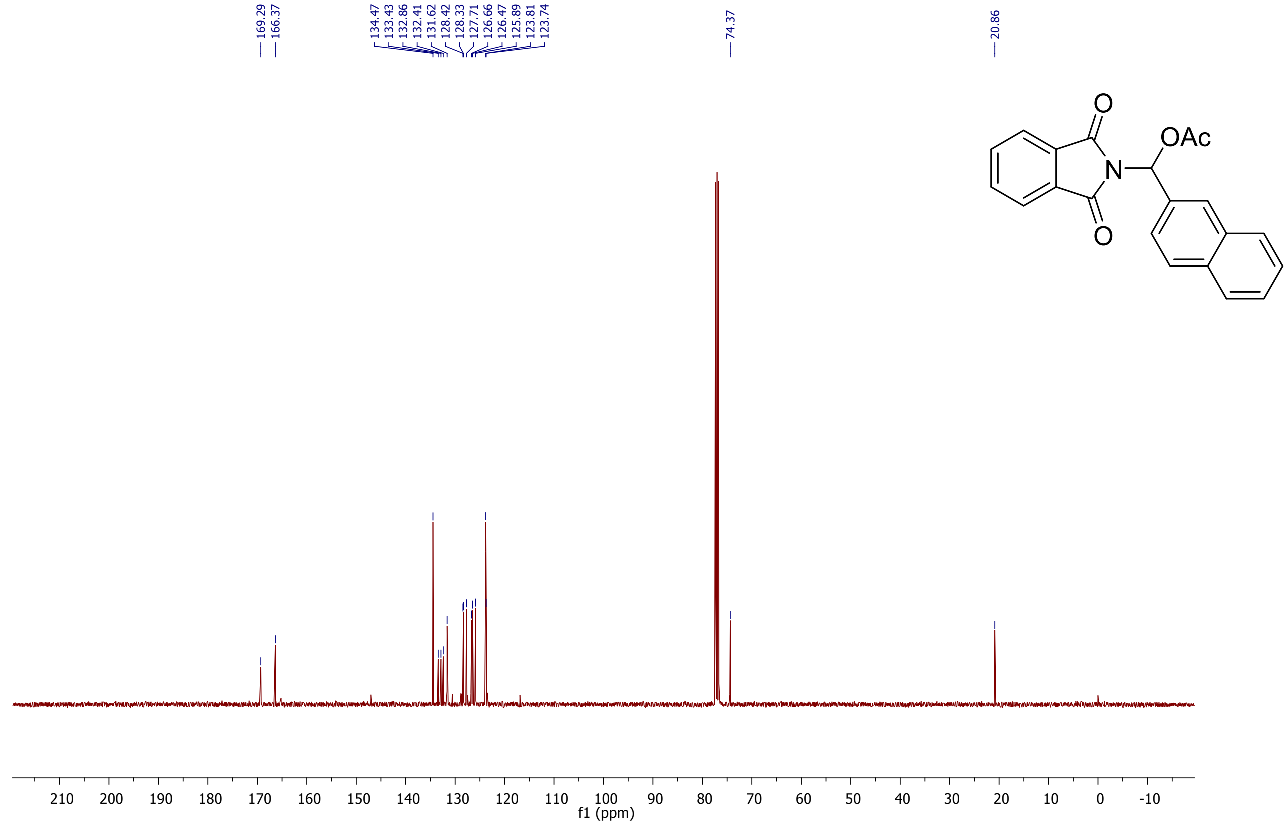

(1,3-dioxoisoindolin-2-yl)(furan-2-yl)methyl acetate (1m)

^{1}H NMR (400 MHz, $CDCl_3$)

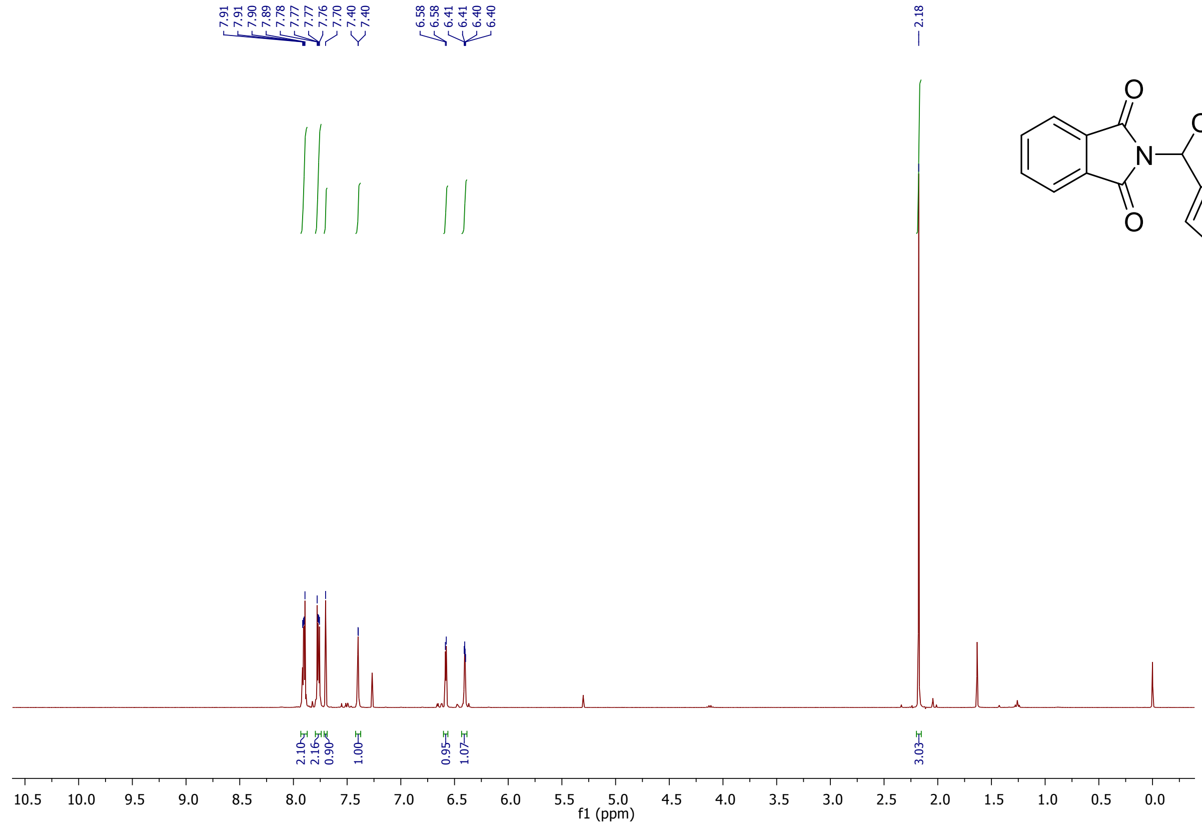

$^{13}C\{^{1}H\}$ NMR (101 MHz, $CDCl_3$)

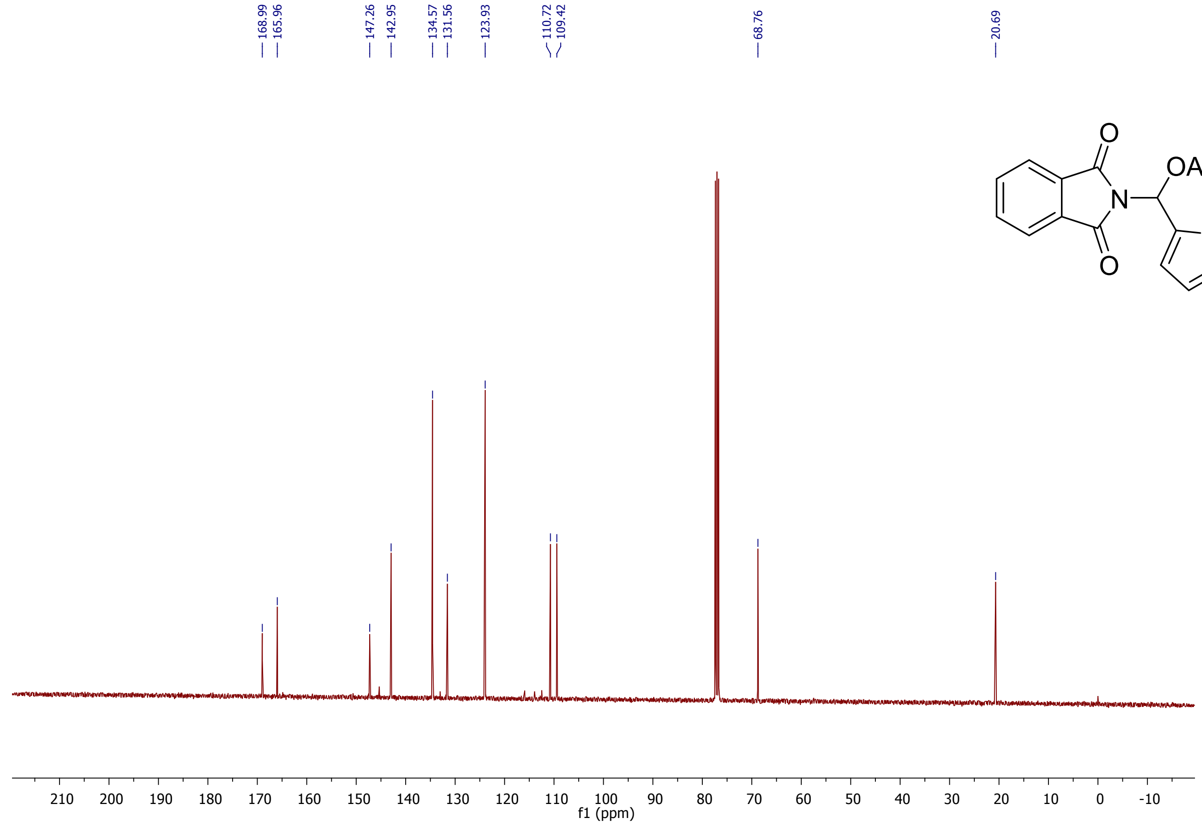

(1,3-dioxoisoindolin-2-yl)(thiophen-2-yl)methyl acetate (1n)

^{1}H NMR (400 MHz, $CDCl_3$)

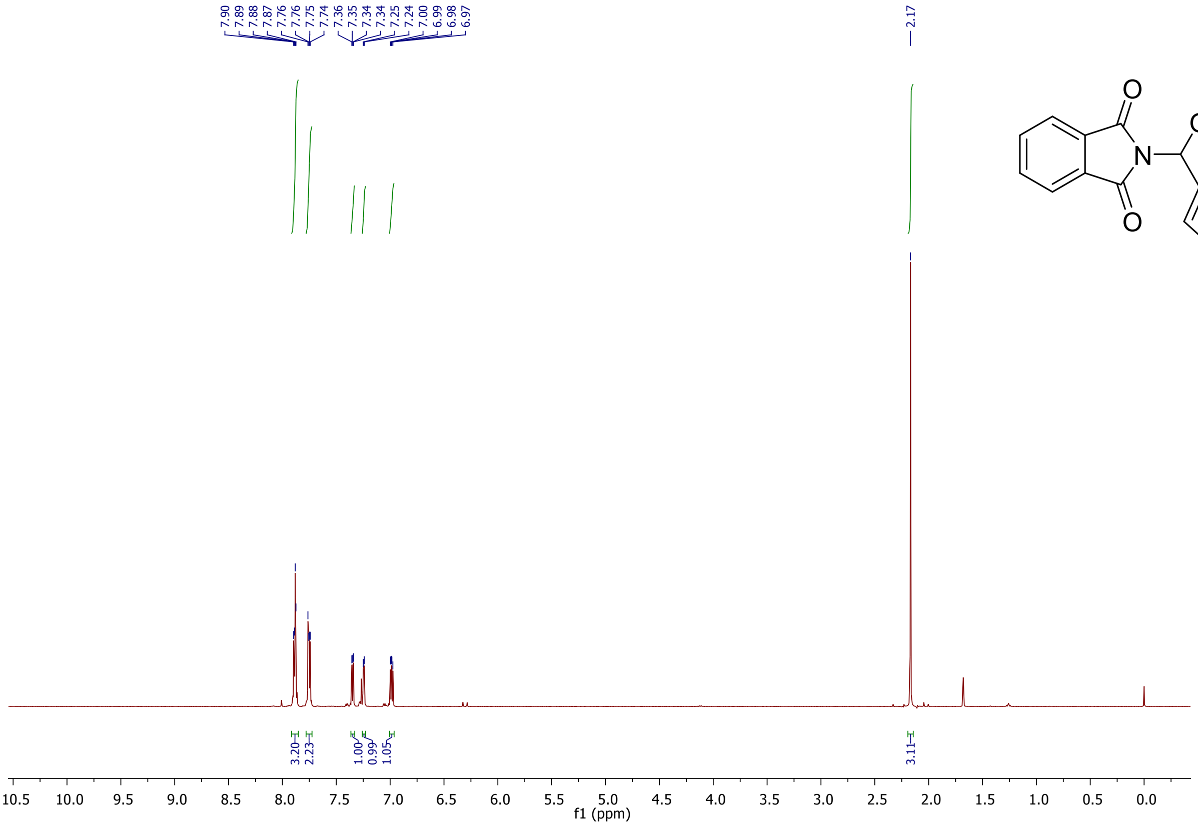

$^{13}C\{^1H\}$ NMR (101 MHz, $CDCl_3$)

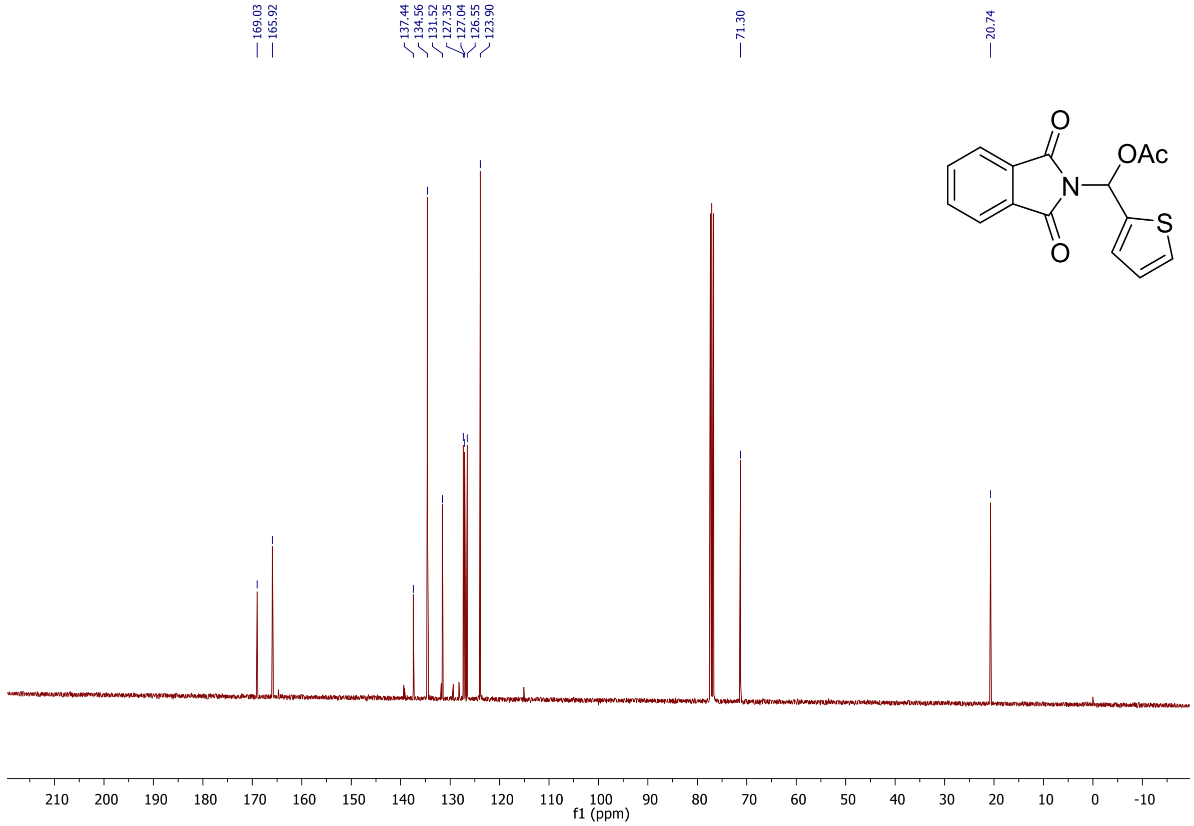

cyclohexyl(1,3-dioxoisoindolin-2-yl)methyl acetate (1o)

^{1}H NMR (400 MHz, $CDCl_3$)

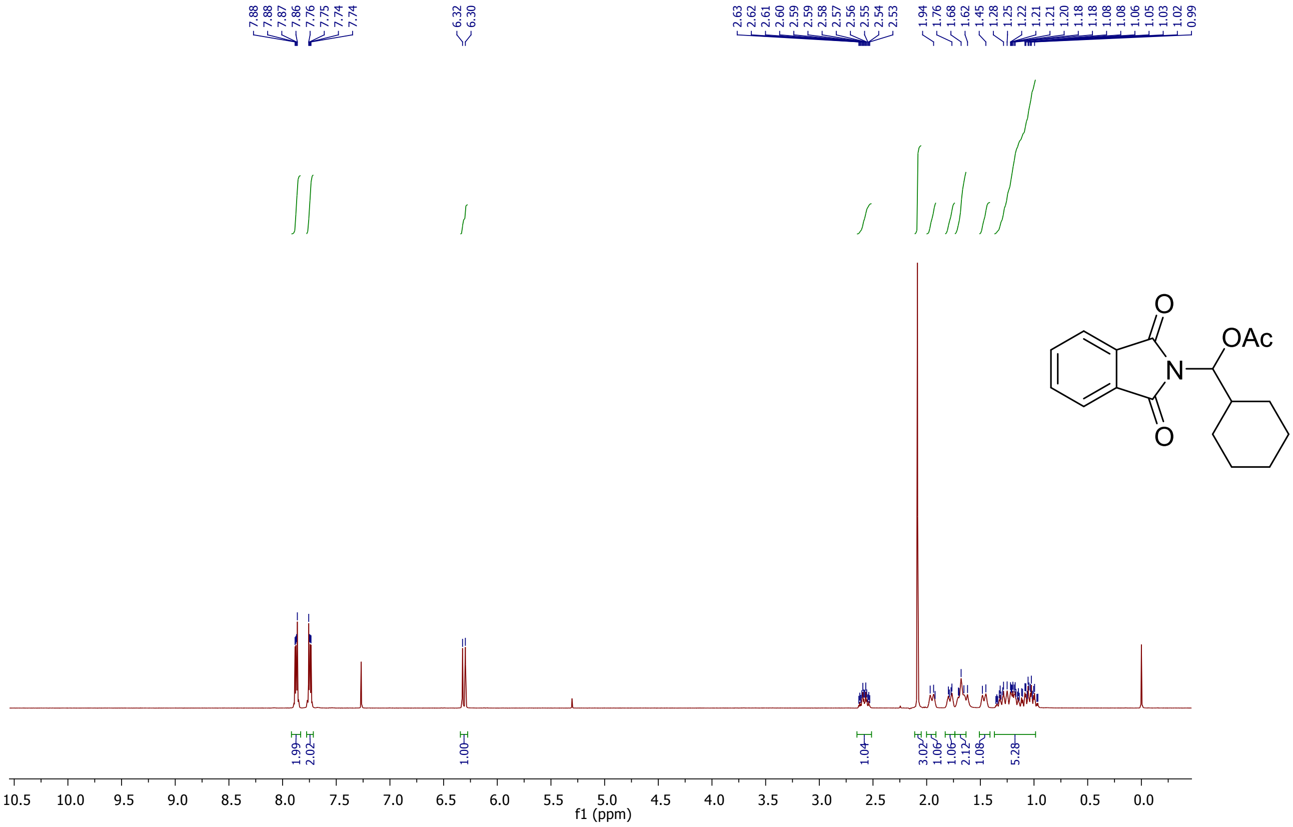

^{13}C{^{1}H} NMR (101 MHz, $CDCl_3$)

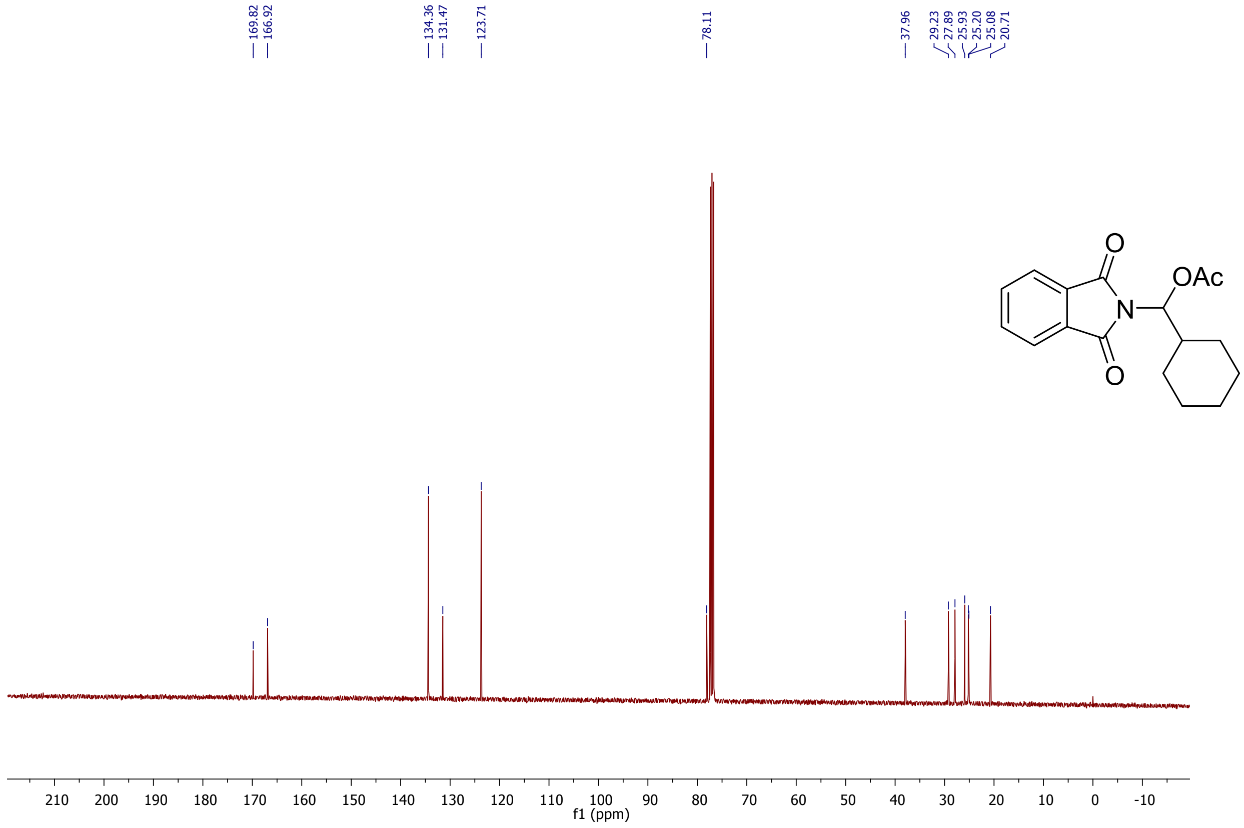

cyclopropyl(1,3-dioxoisoindolin-2-yl)methyl acetate (1p)

^{1}H NMR (400 MHz, $CDCl_3$)

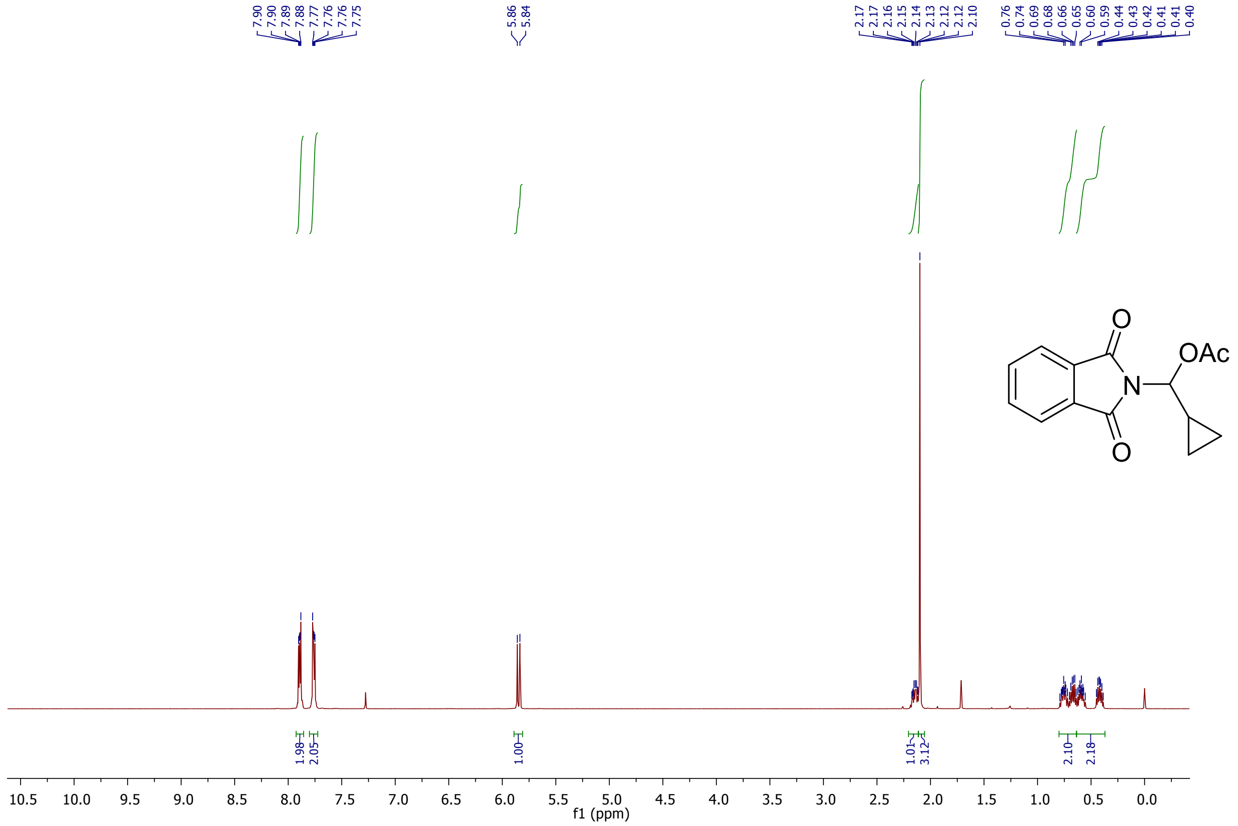

$^{13}C\{^{1}H\}$ NMR (101 MHz, $CDCl_3$)

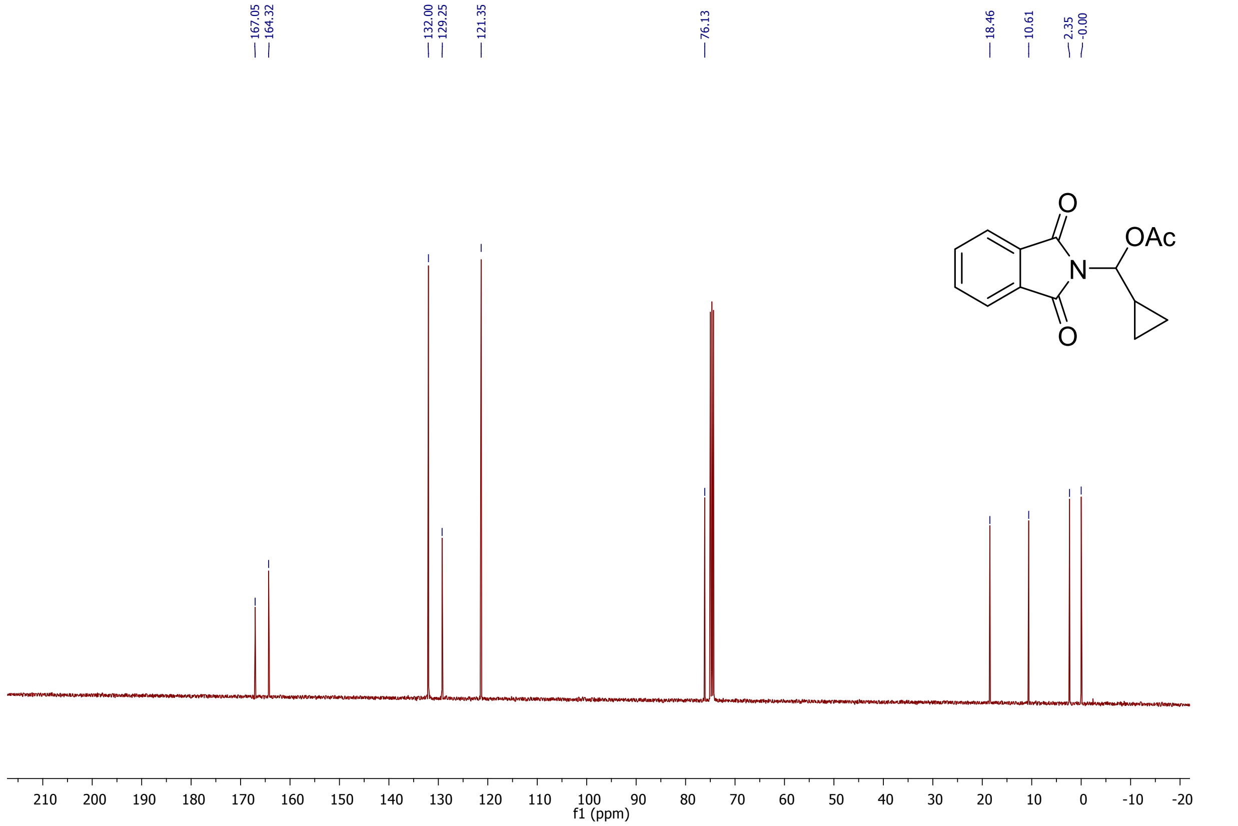

2-(1-phenylbut-3-en-1-yl)isoindoline-1,3-dione (2a)

^{1}H NMR (400 MHz, $CDCl_3$)

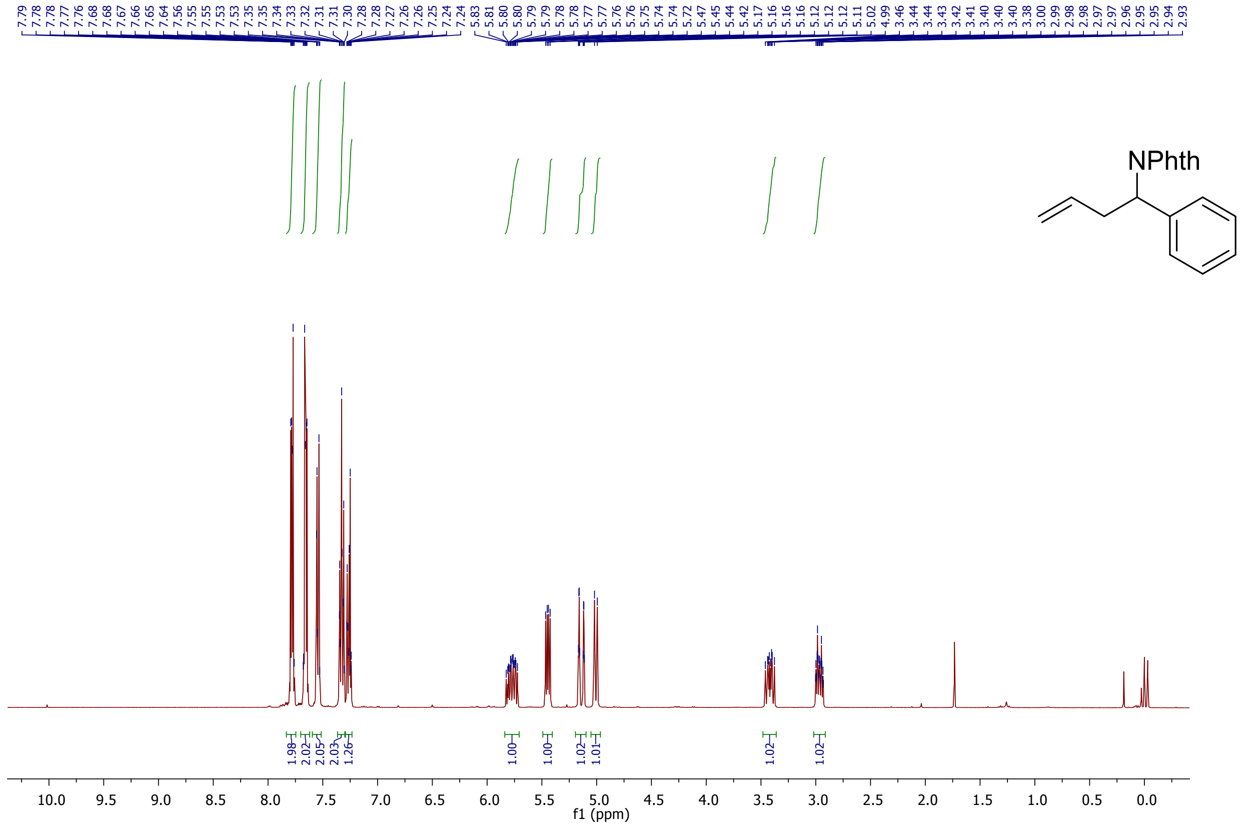

$^{13}C\{^{1}H\}$ NMR (101 MHz, $CDCl_3$)

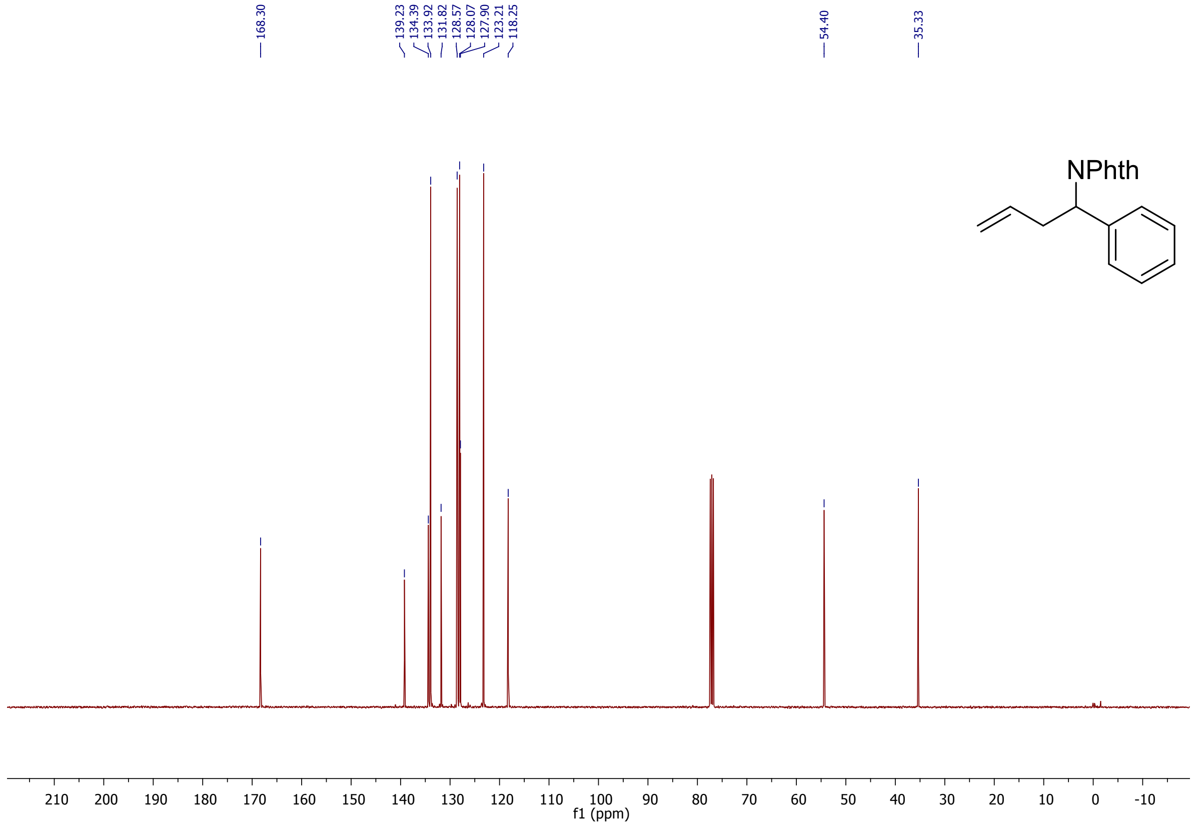

2-(1-(4-methoxyphenyl)but-3-en-1-yl)isoindoline-1,3-dione (2b)

^{1}H NMR (400 MHz, $CDCl_3$)

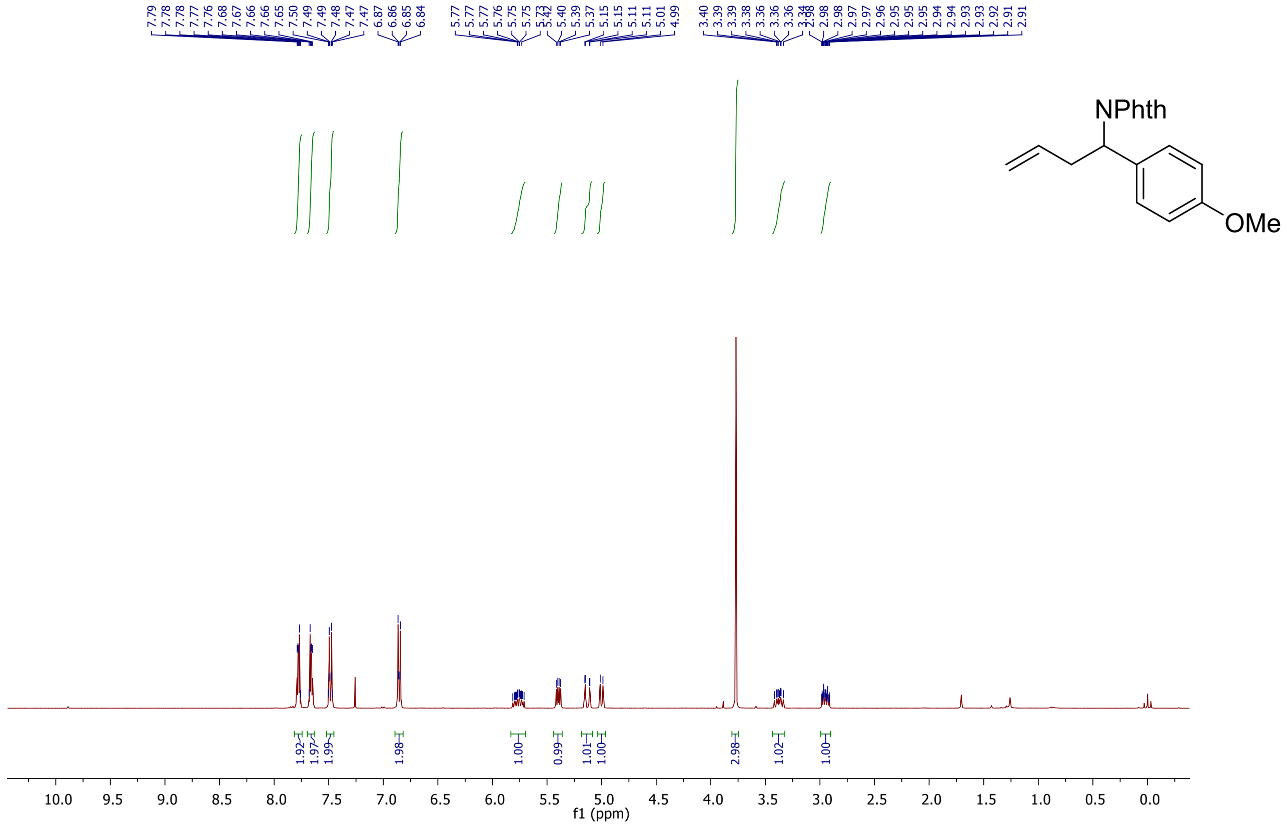

$^{13}C\{^1H\}$ NMR (101 MHz, $CDCl_3$)

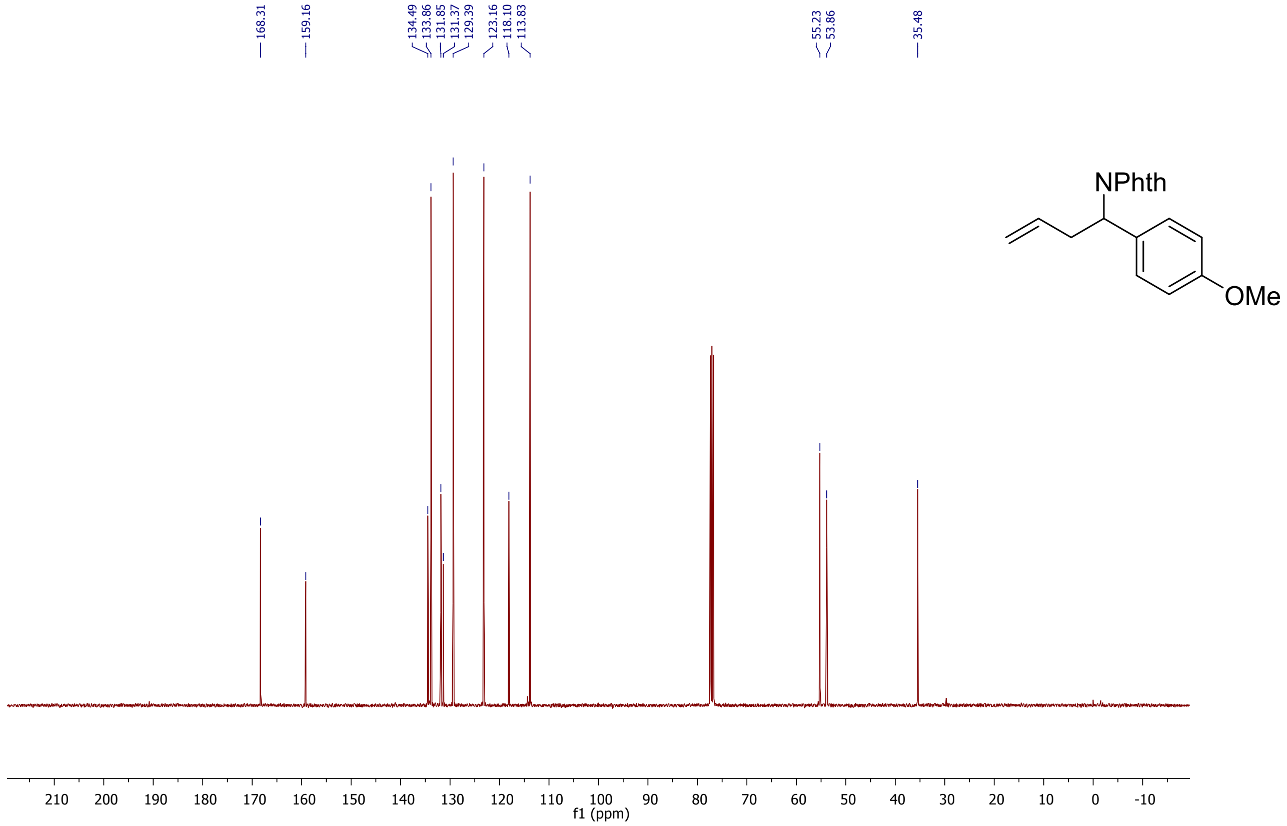

2-(1-(p-tolyl)but-3-en-1-yl)isoindoline-1,3-dione (2c)

^{1}H NMR (400 MHz, $CDCl_3$)

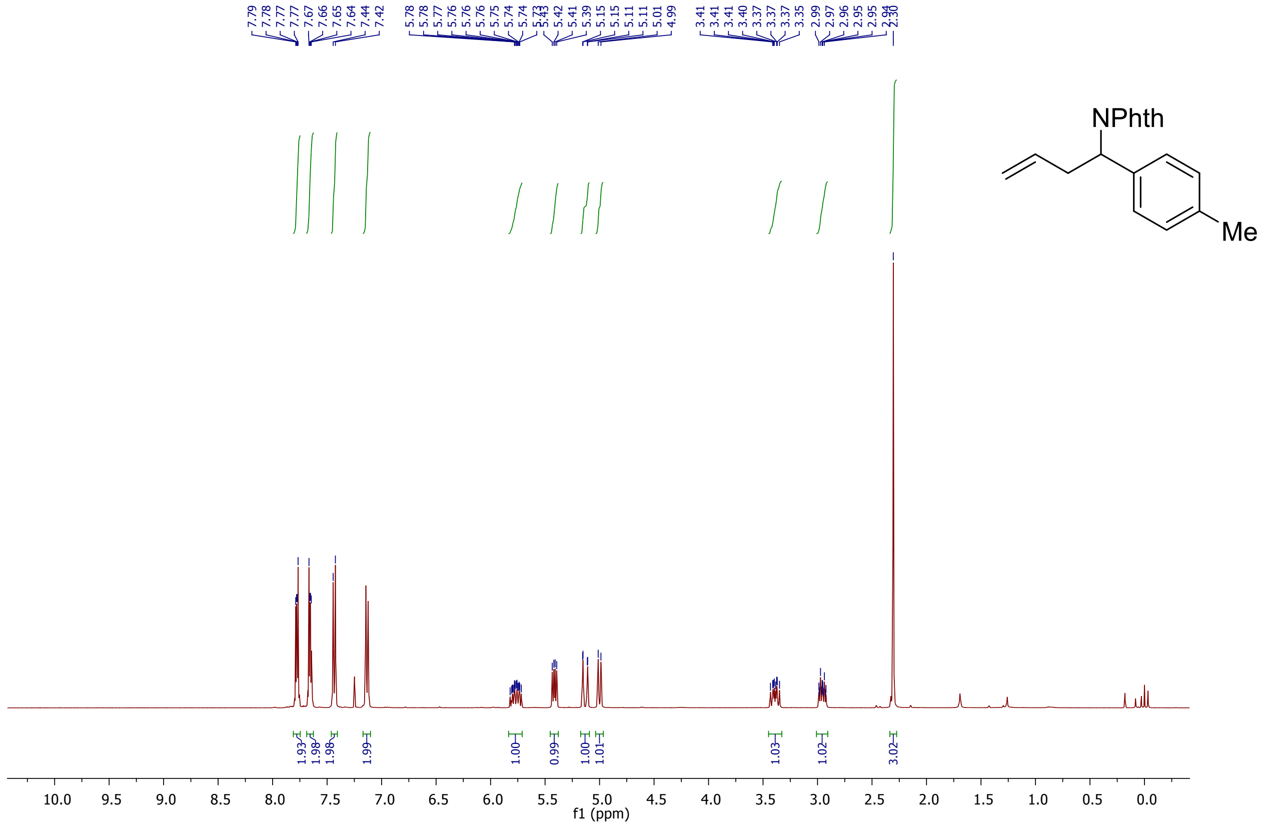

$^{13}C\{^{1}H\}$ NMR (101 MHz, $CDCl_3$)

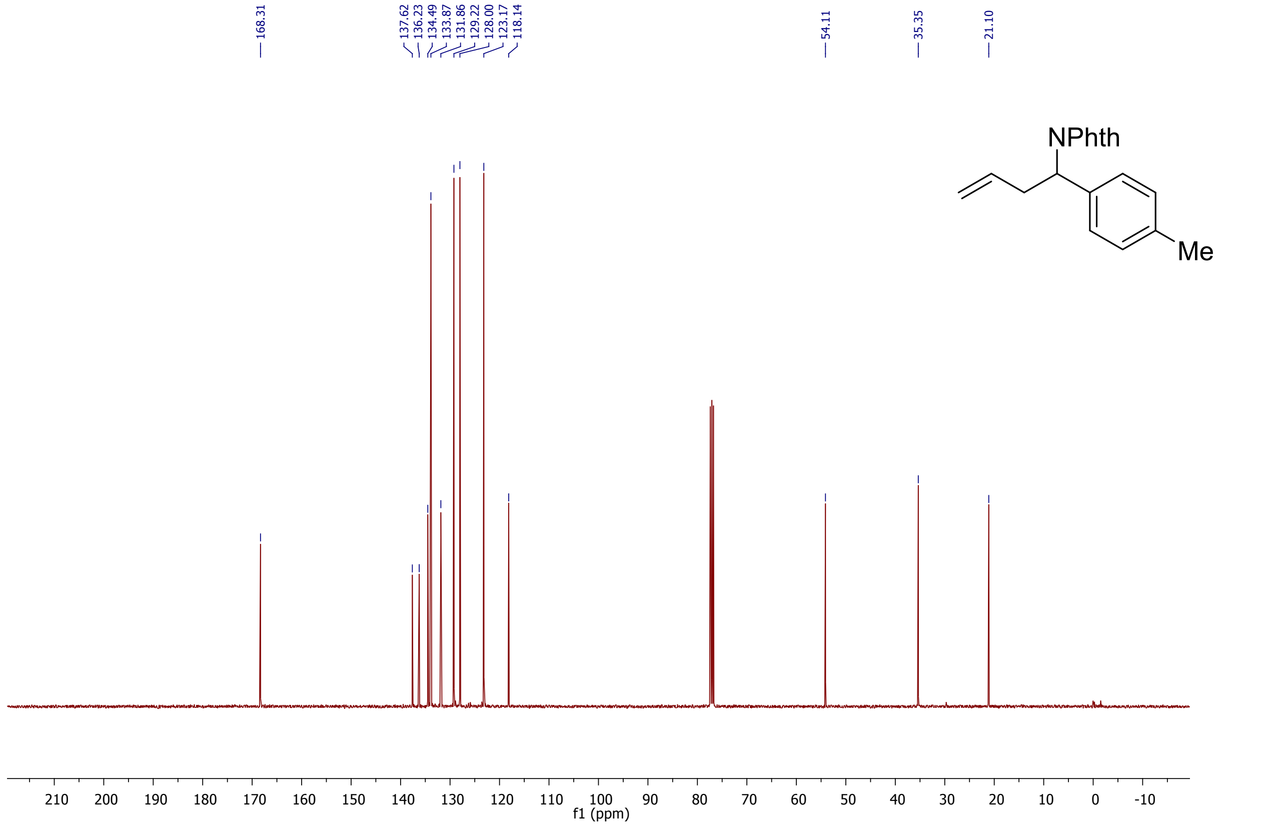

2-(1-(4-fluorophenyl)but-3-en-1-yl)isoindoline-1,3-dione (2d)

^{1}H NMR (400 MHz, $CDCl_3$)

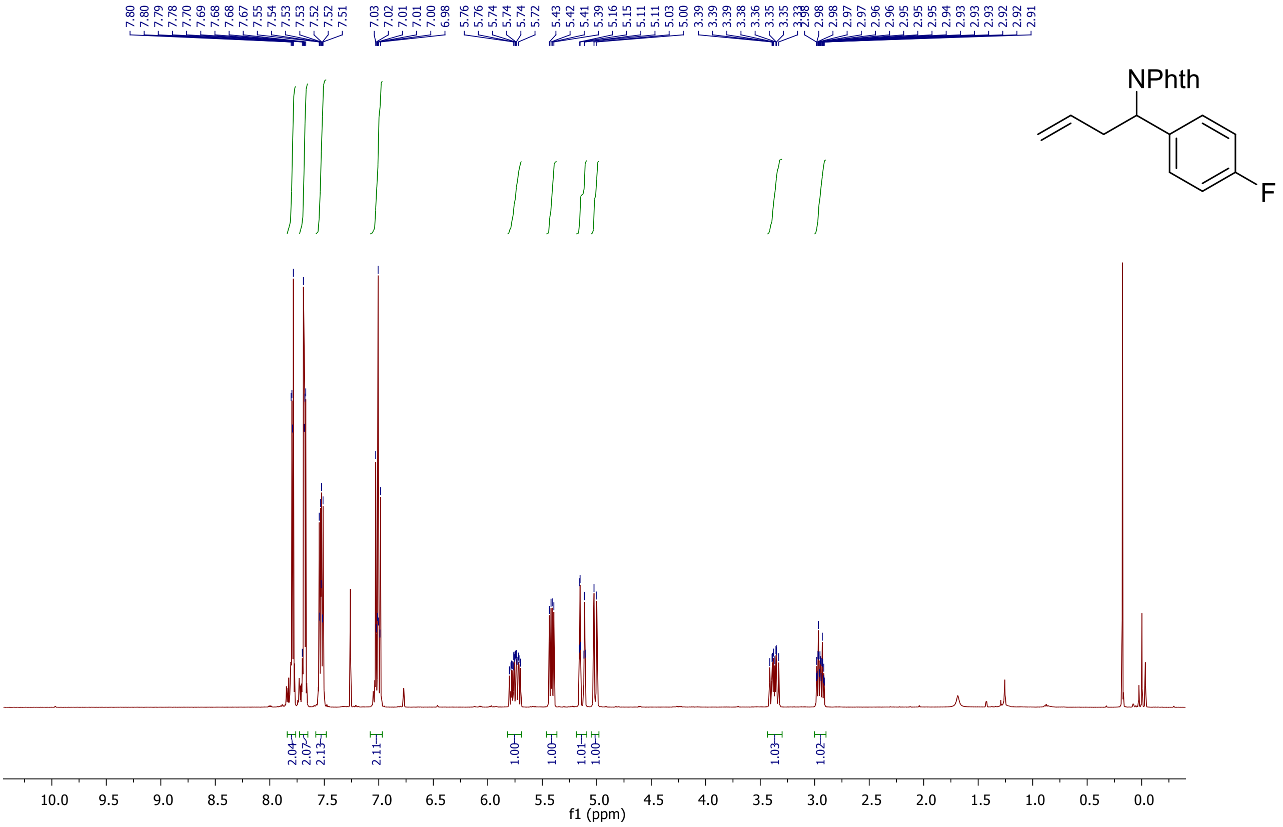

^{13}C{^{1}H} NMR (101 MHz, $CDCl_3$)

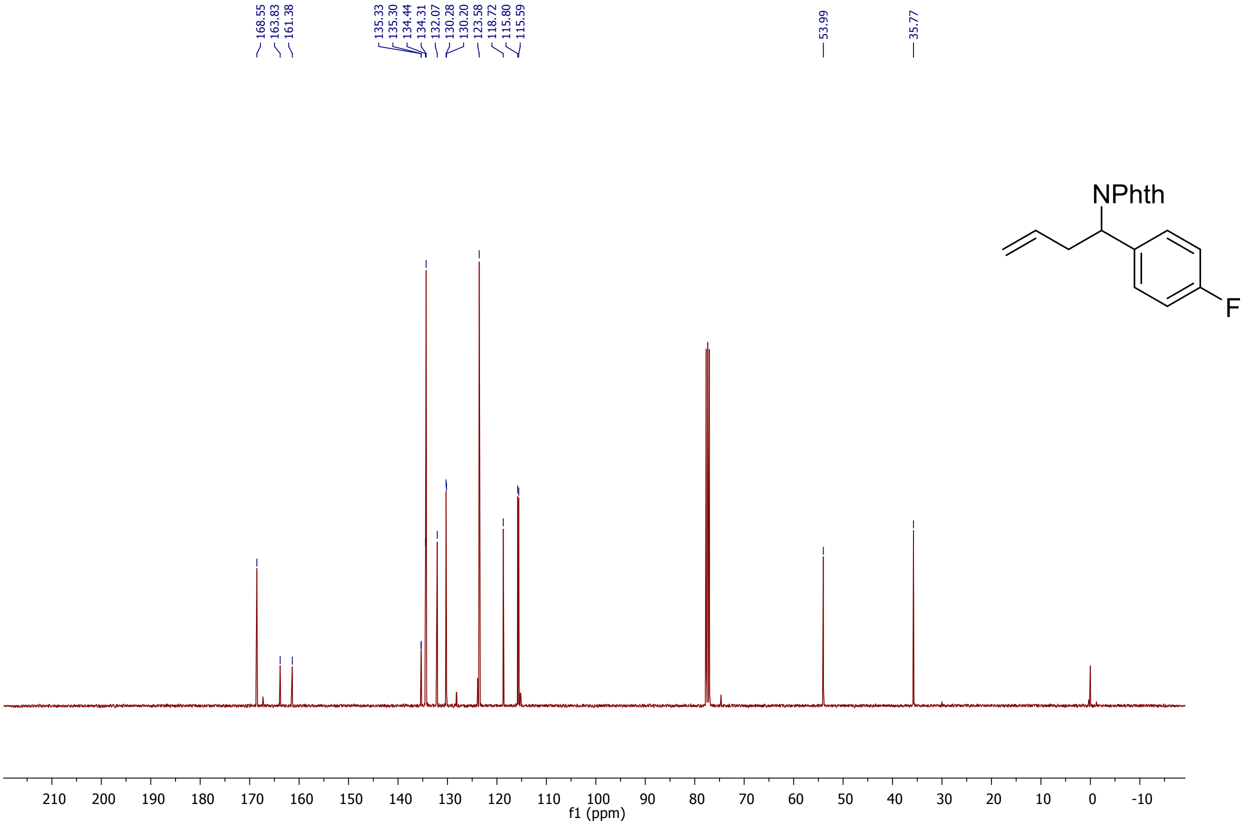

2-(1-(4-(trifluoromethyl)phenyl)but-3-en-1-yl)isoindoline-1,3-dione (2e)

^{1}H NMR (400 MHz, $CDCl_3$)

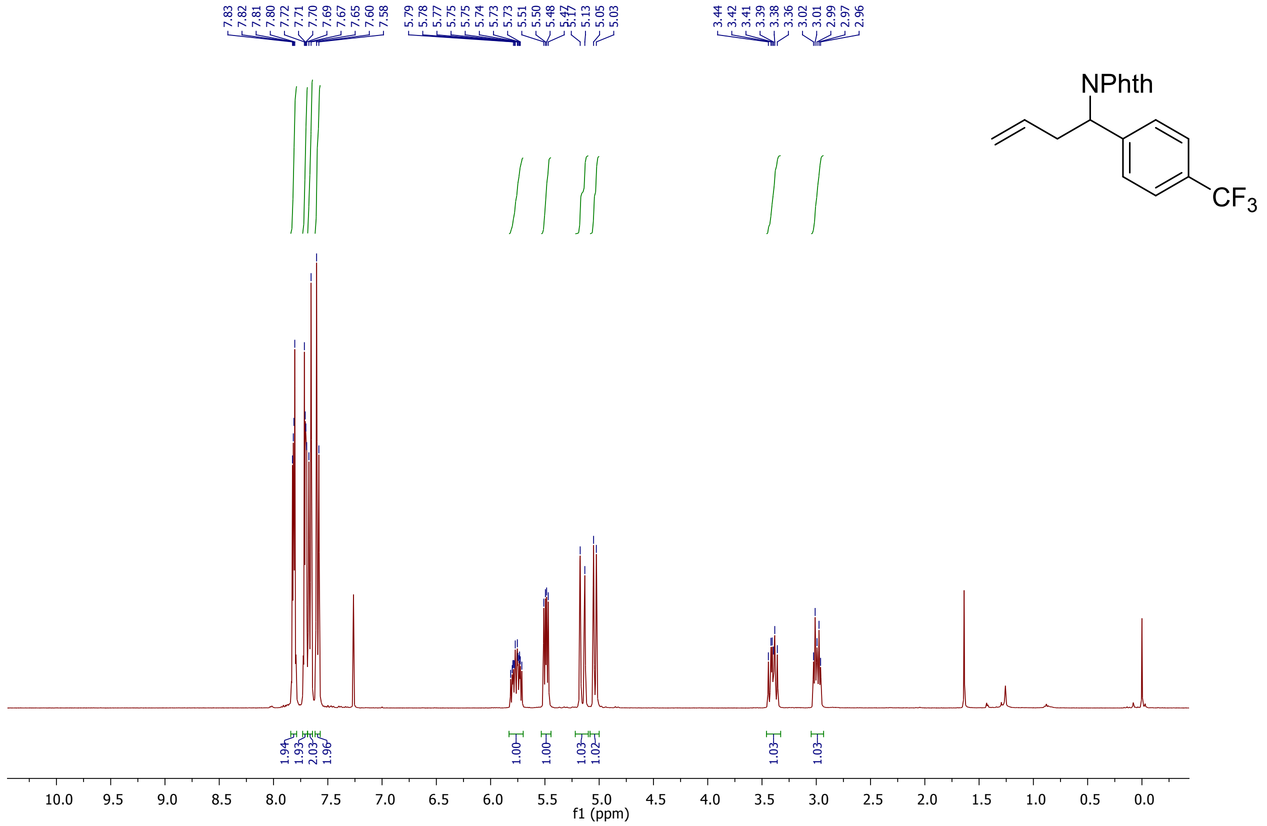

$^{13}C\{^1H\}$ NMR (101 MHz, $CDCl_3$)

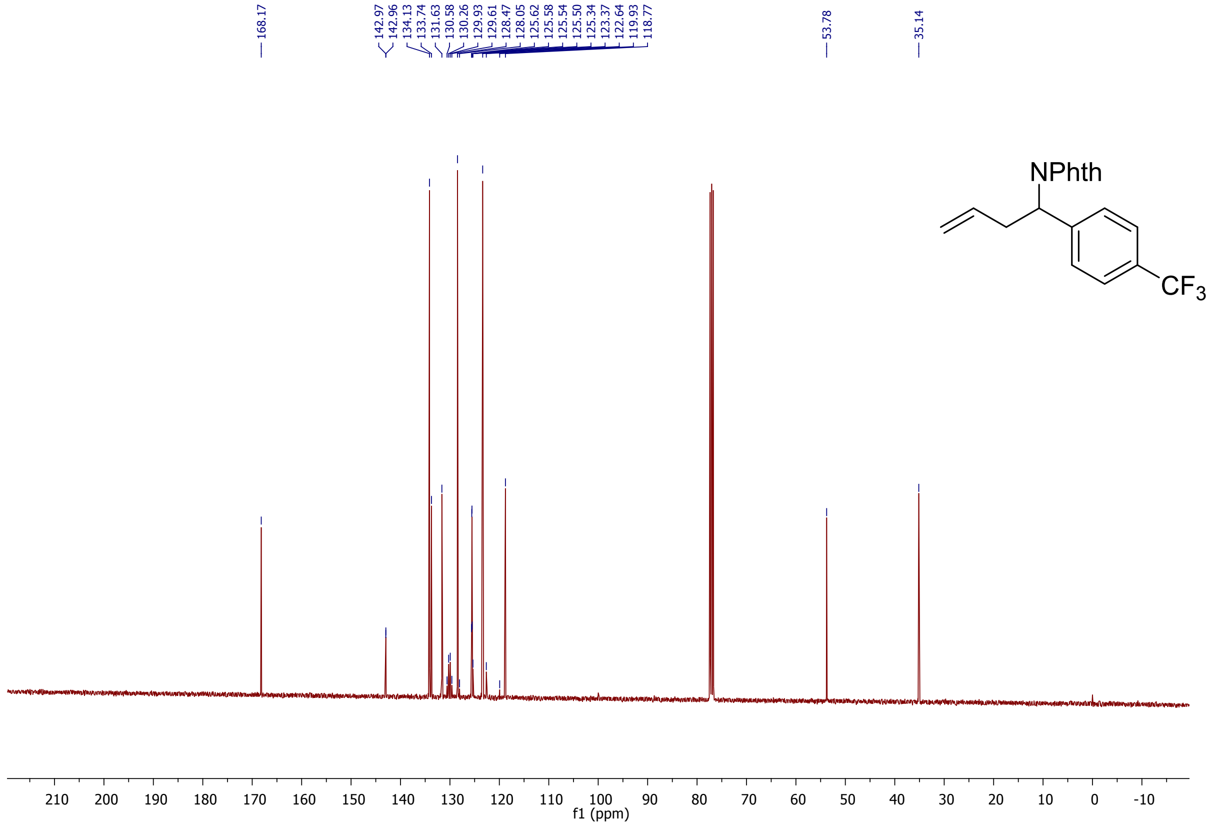

2-(1-(2-methoxyphenyl)but-3-en-1-yl)isoindoline-1,3-dione (2f)

^{1}H NMR (400 MHz, $CDCl_3$)

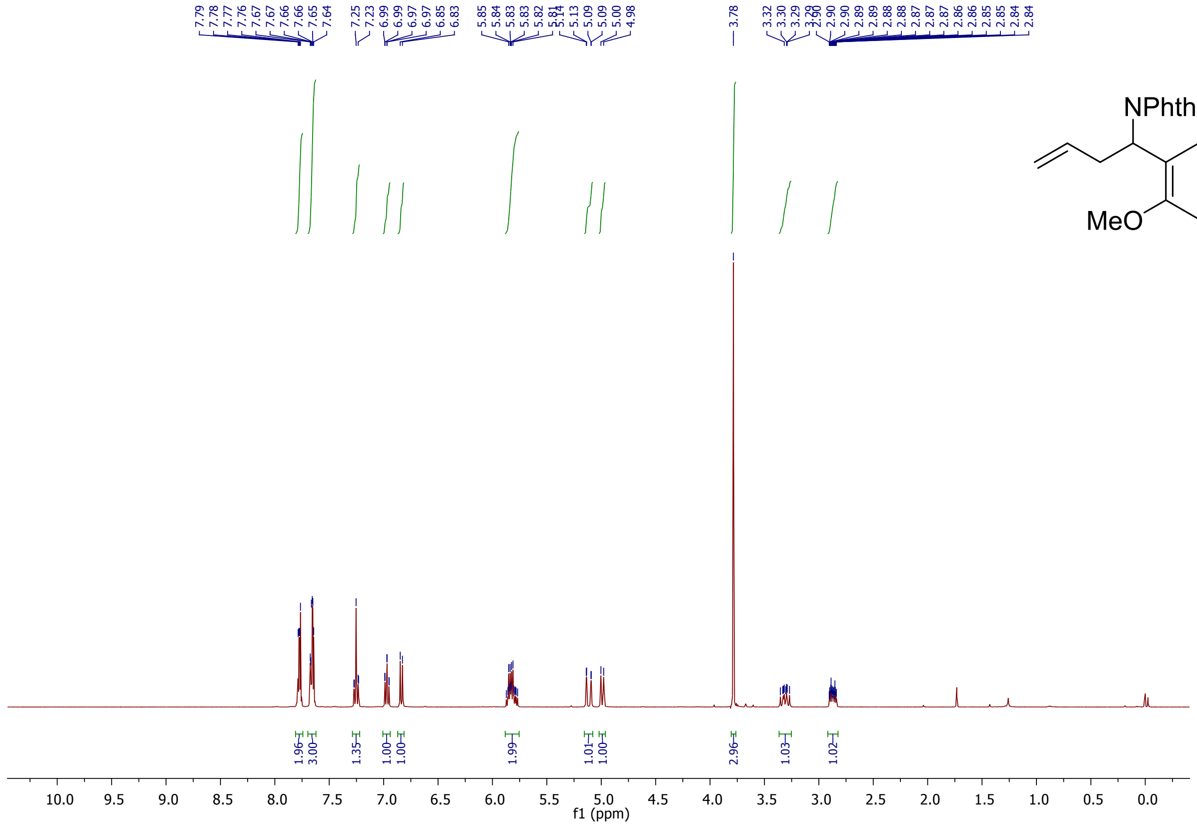

$^{13}C\{^{1}H\}$ NMR (101 MHz, $CDCl_3$)

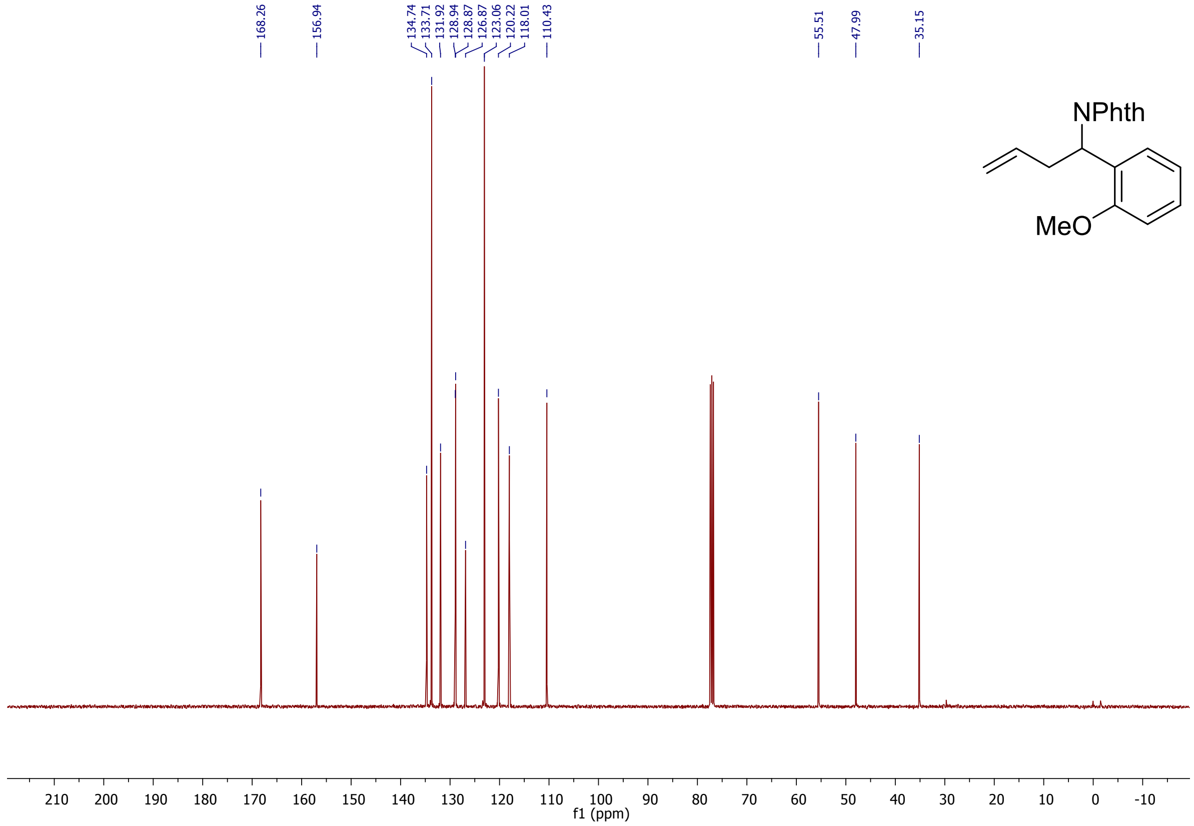

2-(1-(2-bromophenyl)but-3-en-1-yl)isoindoline-1,3-dione (2g)

^{1}H NMR (400 MHz, $CDCl_3$)

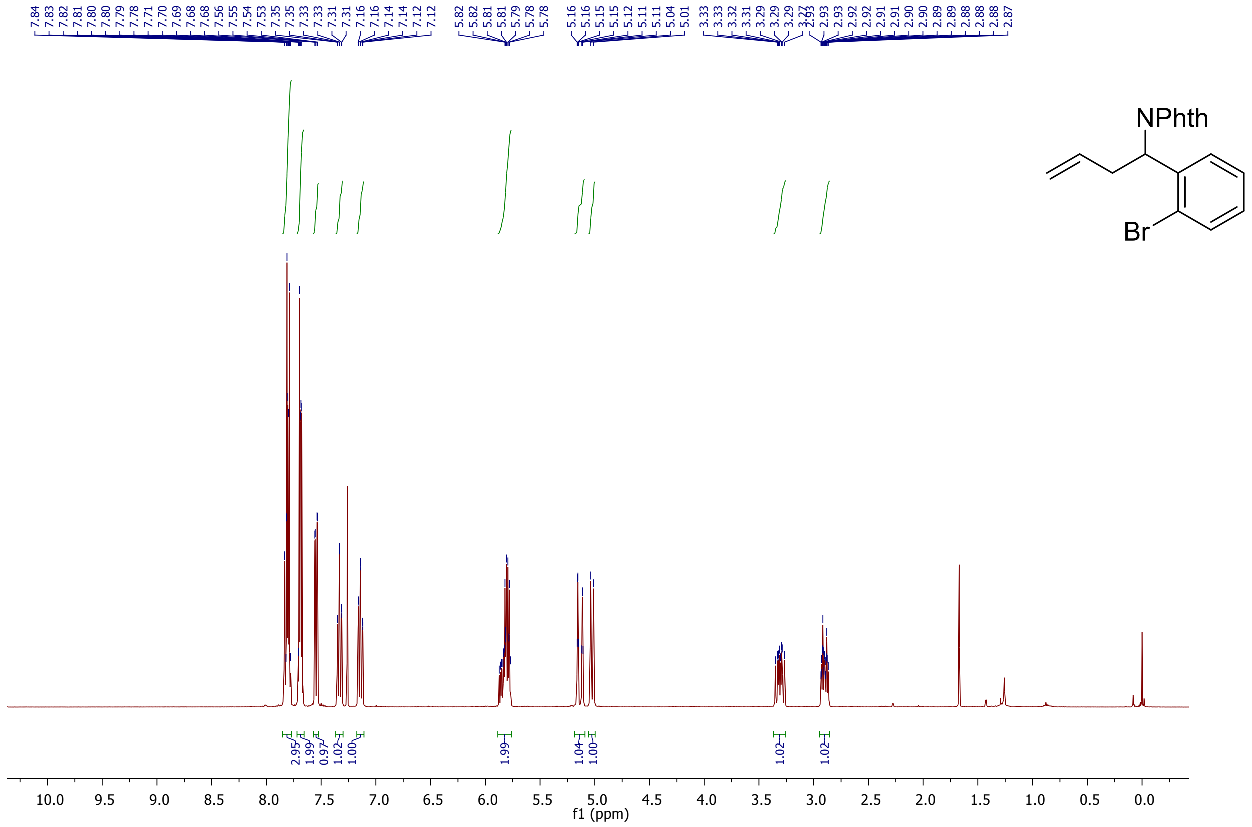

$^{13}C\{^{1}H\}$ NMR (101 MHz, $CDCl_3$)

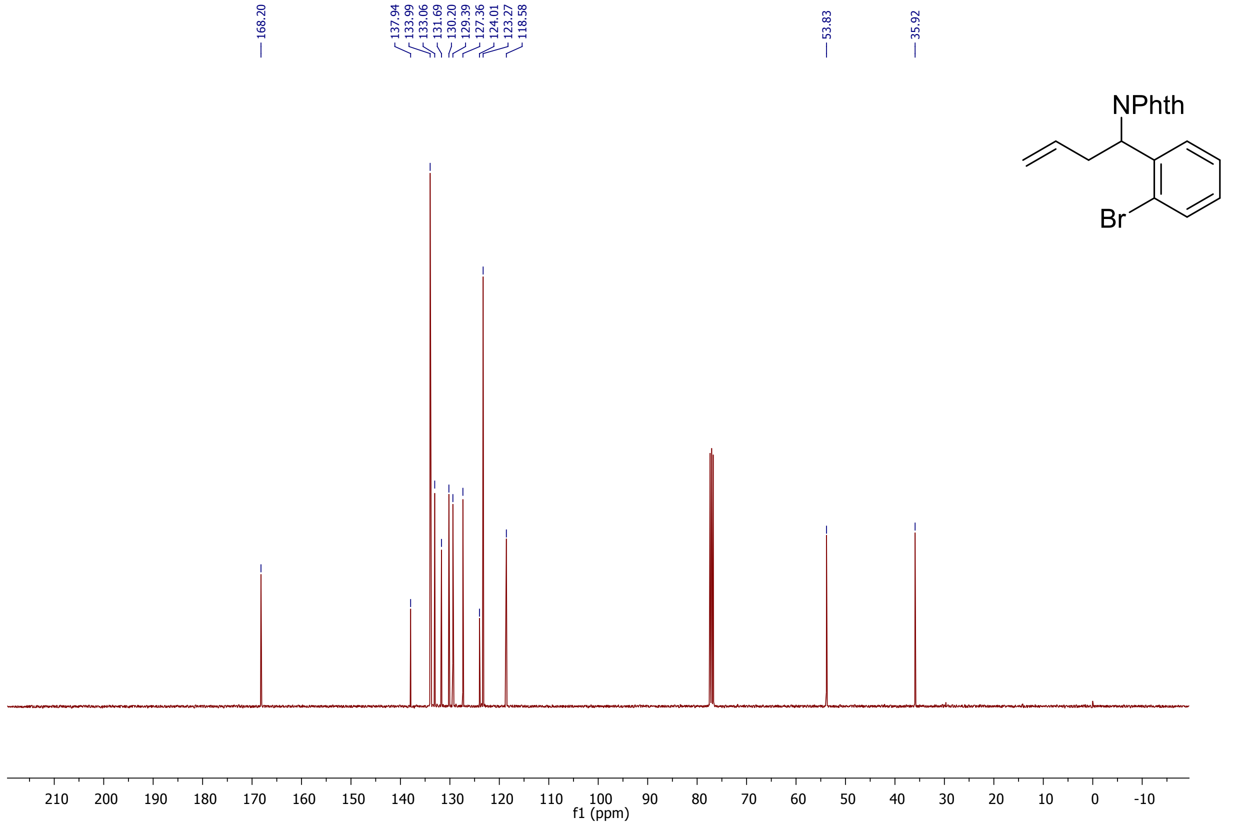

2-(1-(3-chlorophenyl)but-3-en-1-yl)isoindoline-1,3-dione (2h)

^{1}H NMR (400 MHz, $CDCl_3$)

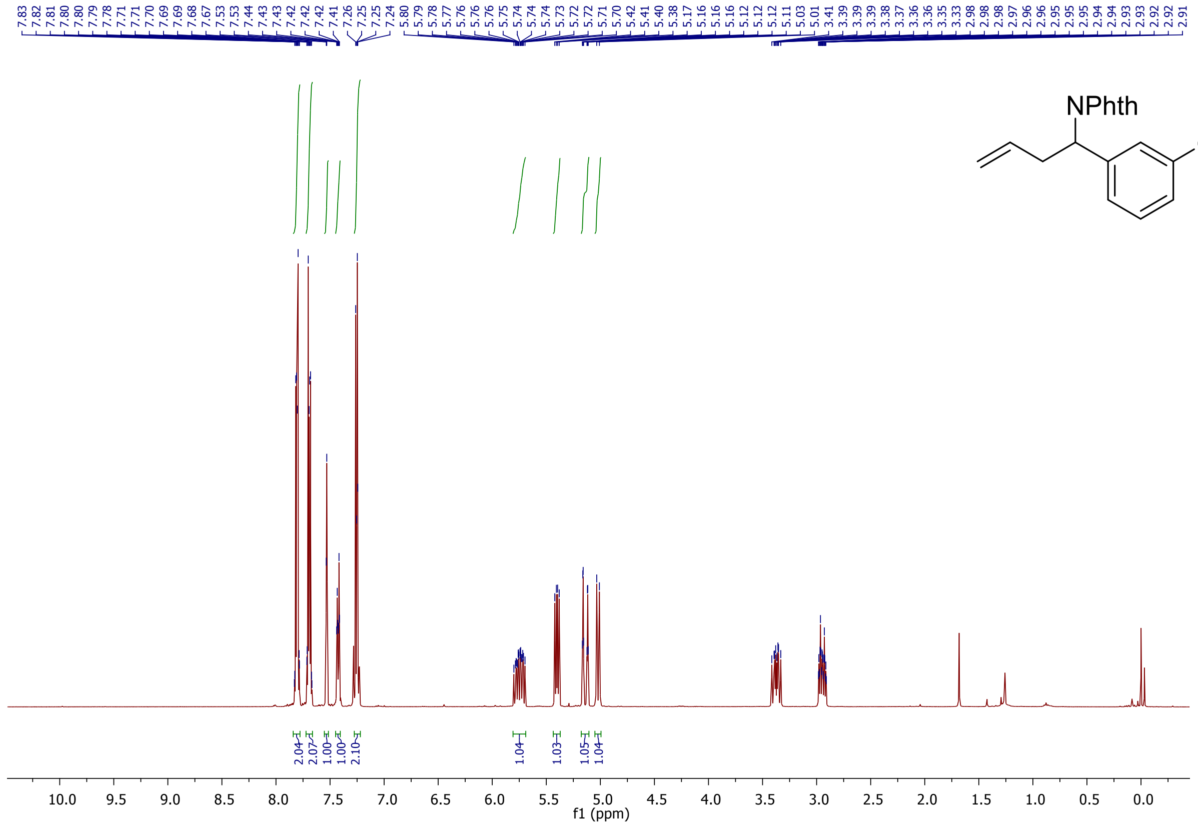

^{13}C{^{1}H} NMR (101 MHz, $CDCl_3$)

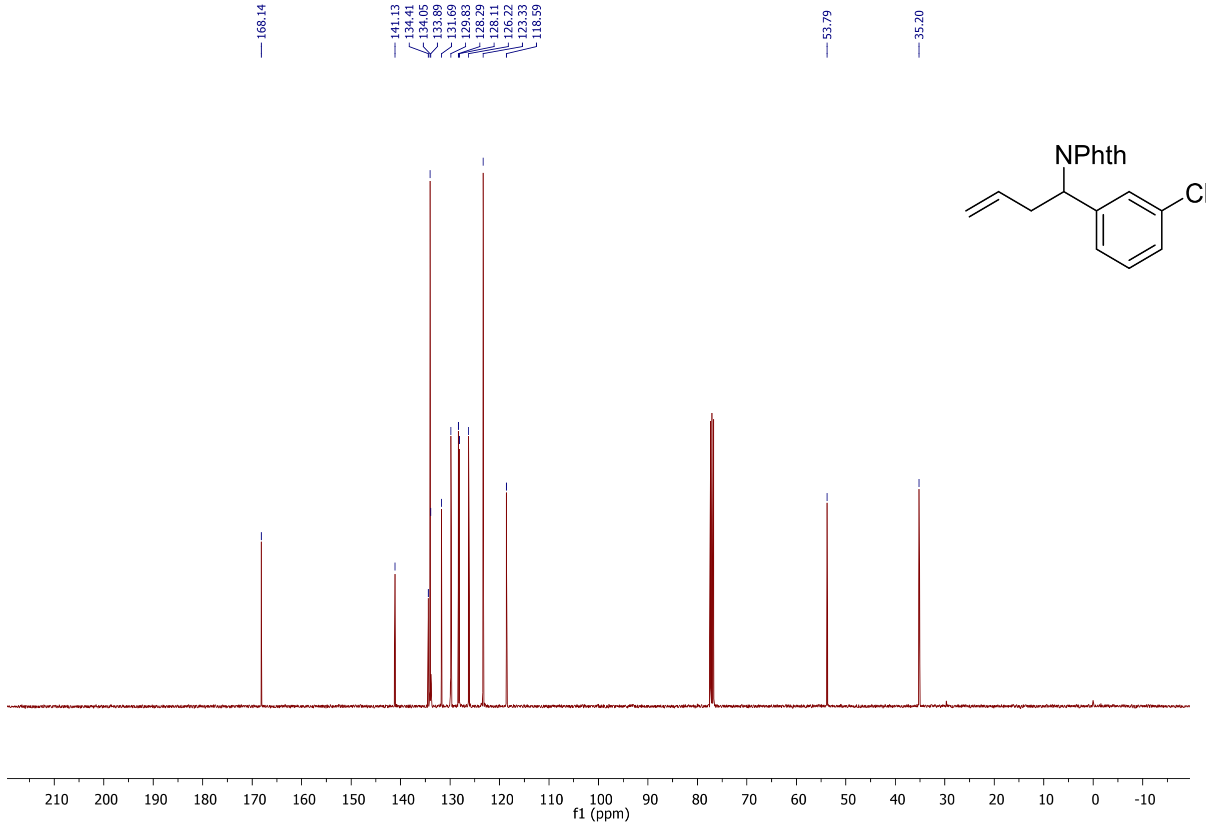

2-(1-(3-bromo-2-fluorophenyl)but-3-en-1-yl)isoindoline-1,3-dione (2i)

^{1}H NMR (400 MHz, $CDCl_3$)

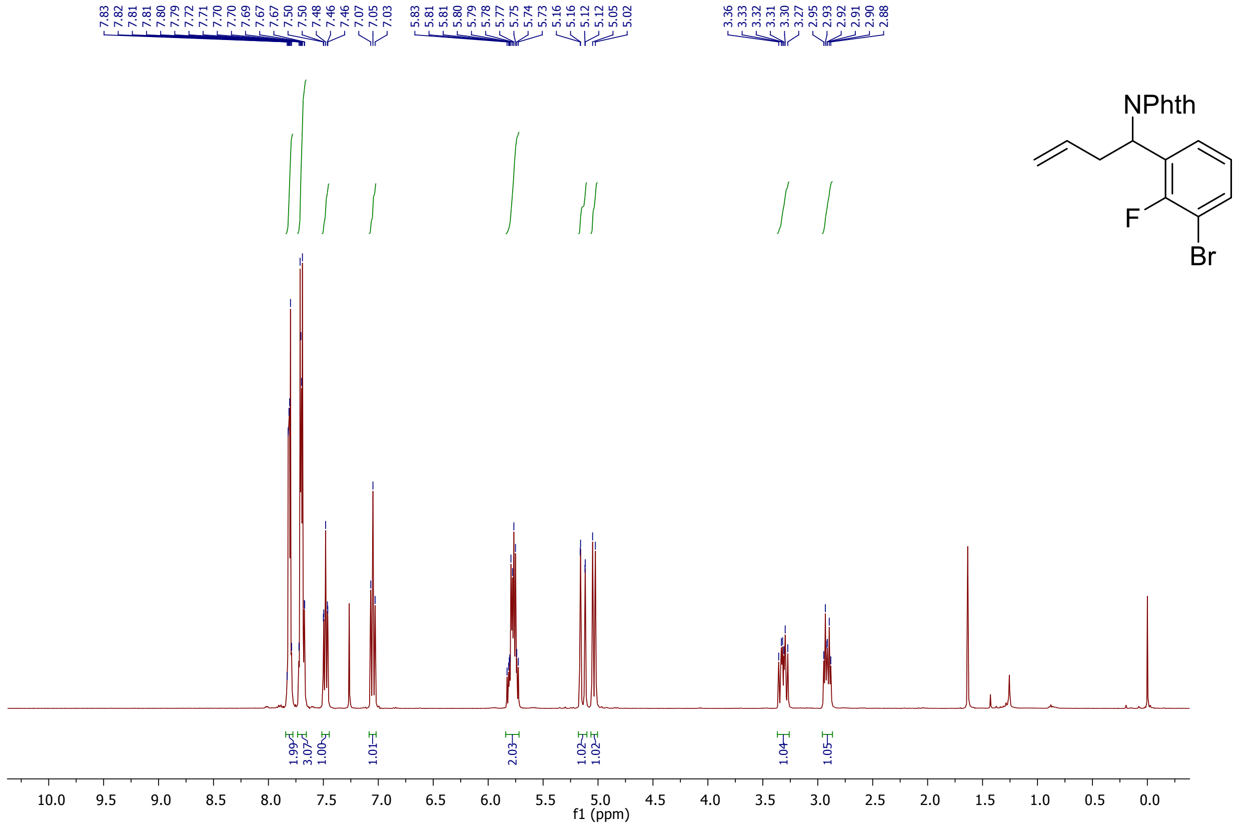

$^{13}C\{^{1}H\}$ NMR (101 MHz, $CDCl_3$)

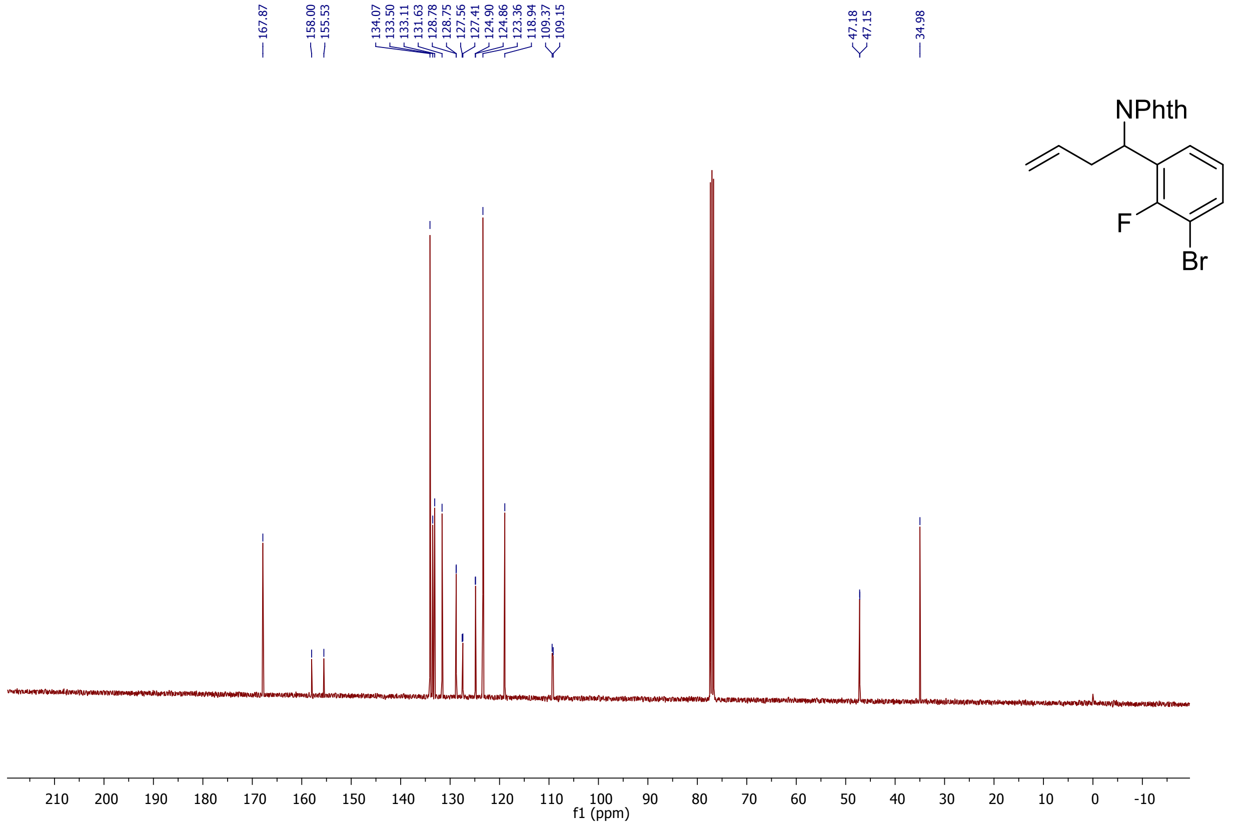

2-(1-(3,5-bis(trifluoromethyl)phenyl)but-3-en-1-yl)isoindoline-1,3-dione (2j)

^{1}H NMR (400 MHz, $CDCl_3$)

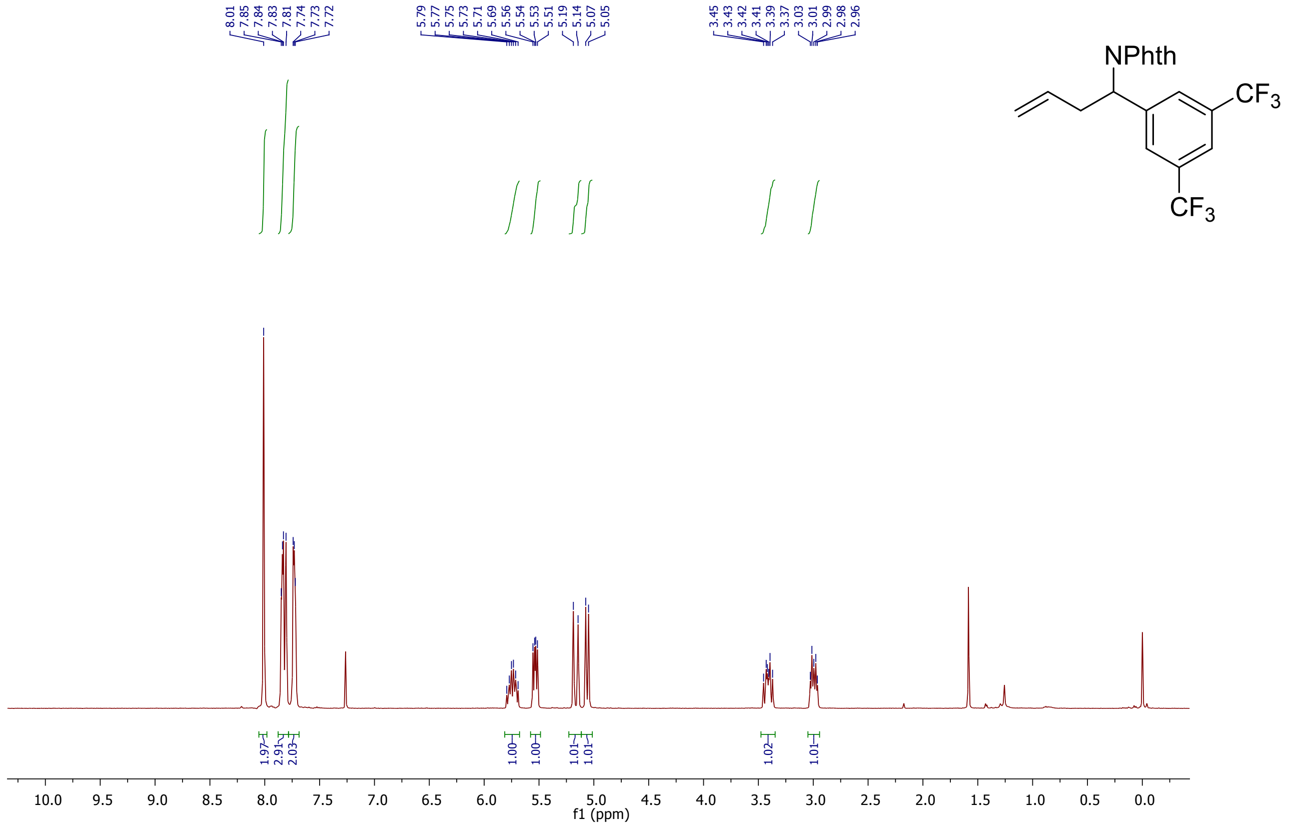

$^{13}C\{^{1}H\}$ NMR (101 MHz, $CDCl_3$)

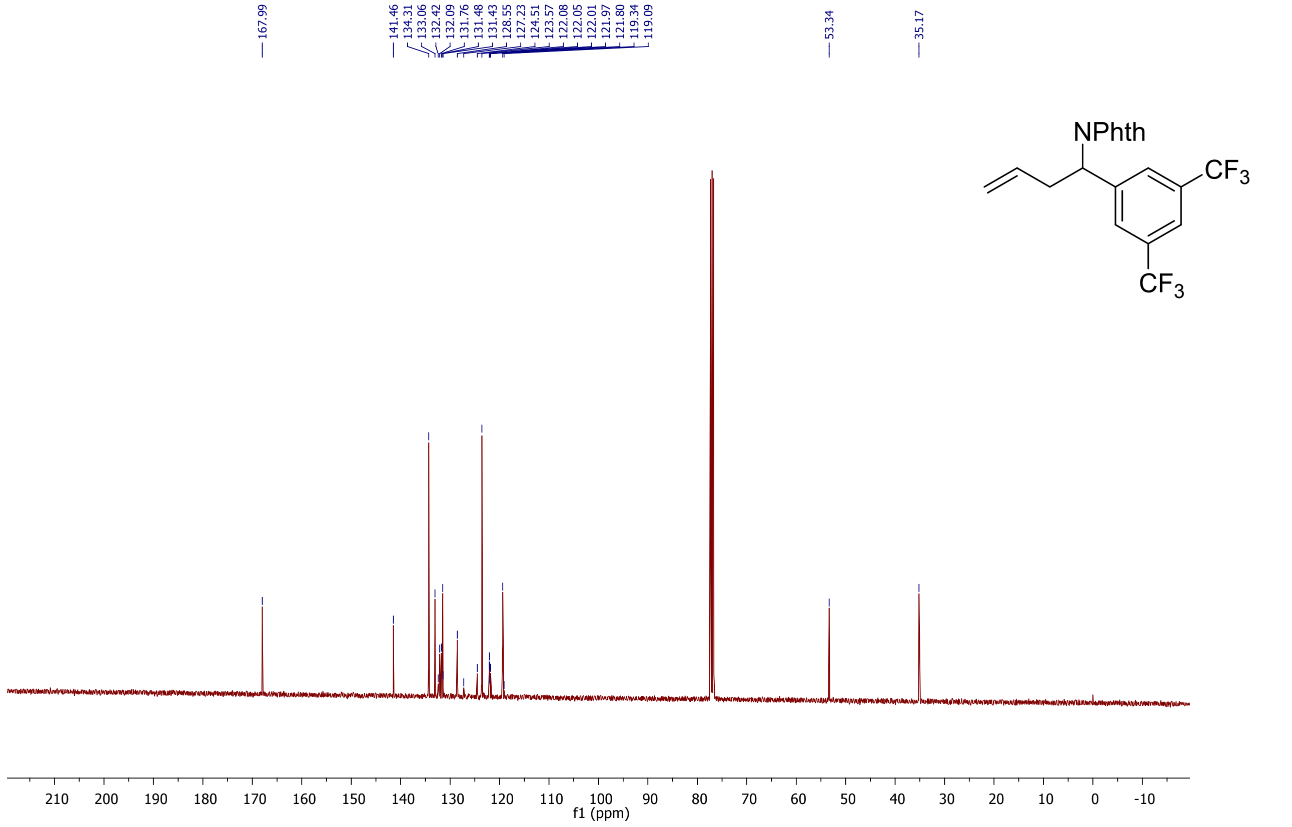

2-(1-(4-nitrophenyl)but-3-en-1-yl)isoindoline-1,3-dione (2k)

^{1}H NMR (400 MHz, $CDCl_3$)

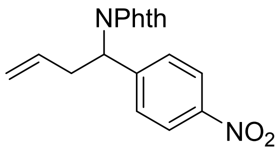

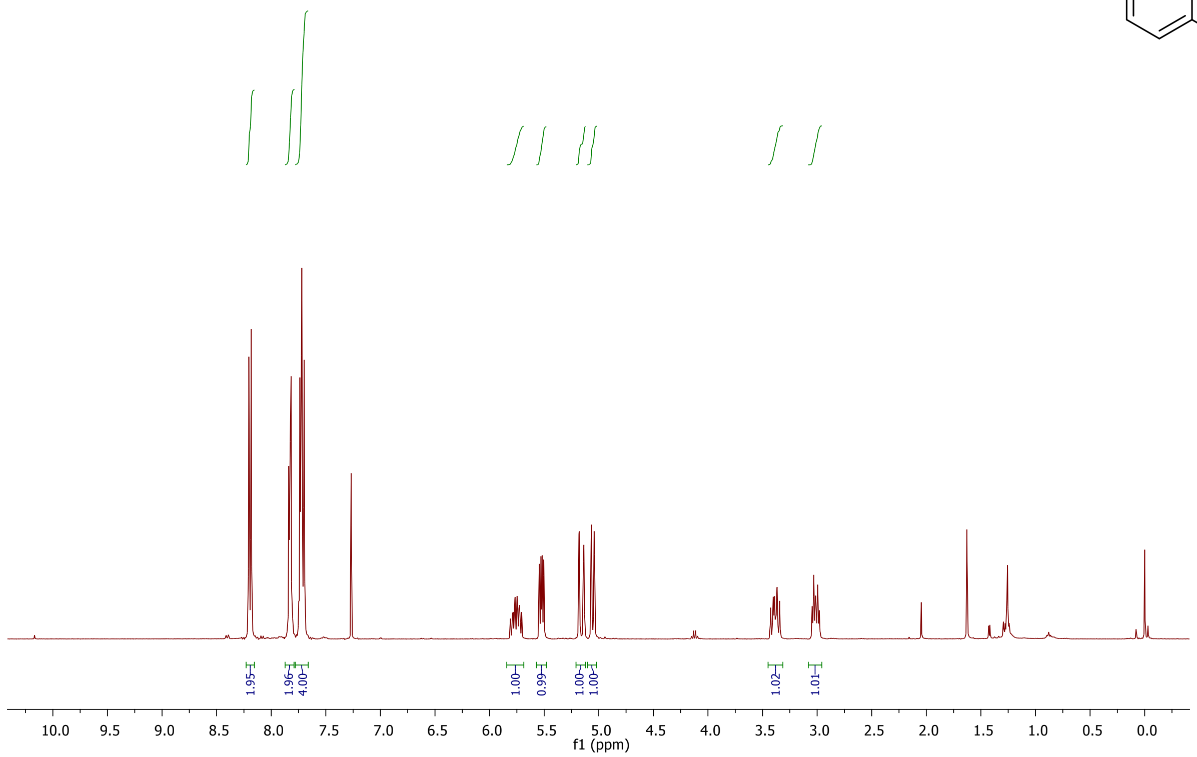

$^{13}C\{^{1}H\}$ NMR (101 MHz, $CDCl_3$)

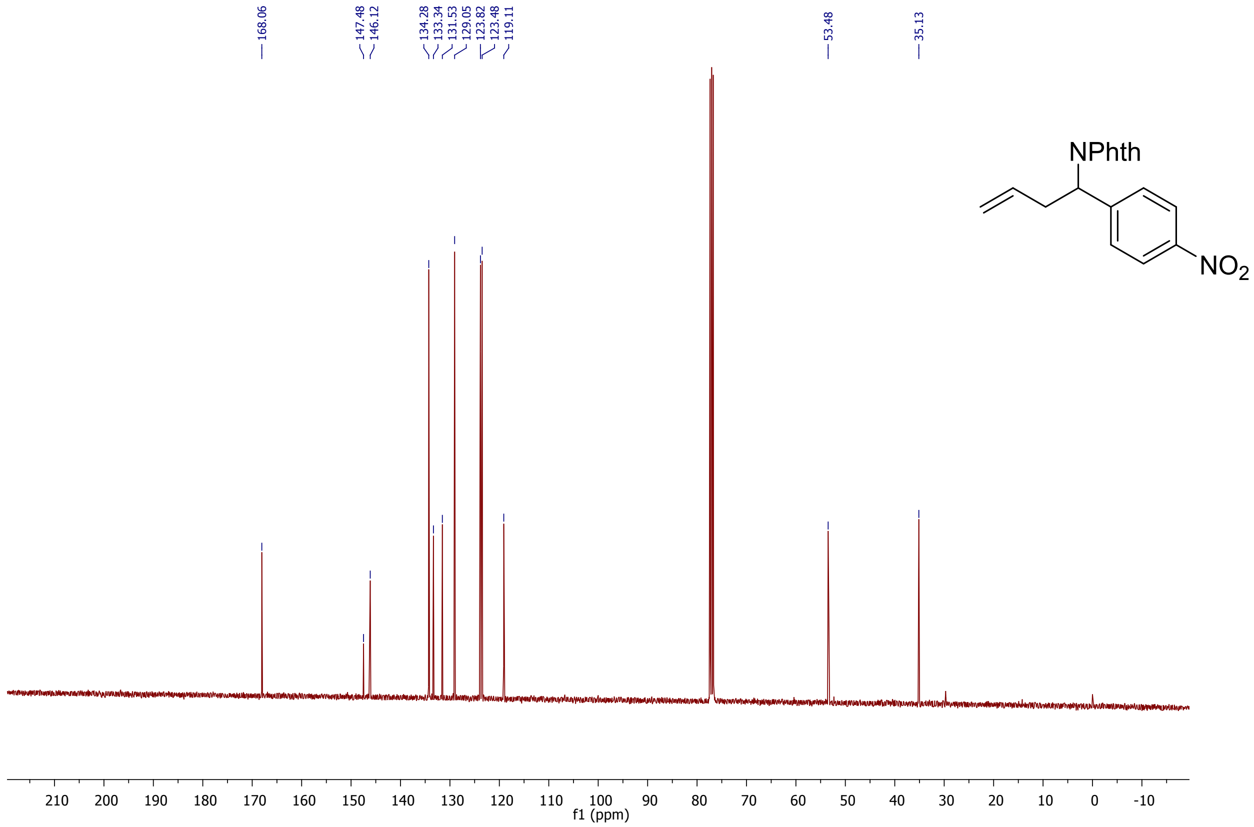

2-(1-(naphthalen-2-yl)but-3-en-1-yl)isoindoline-1,3-dione (2l)

^{1}H NMR (400 MHz, $CDCl_3$)

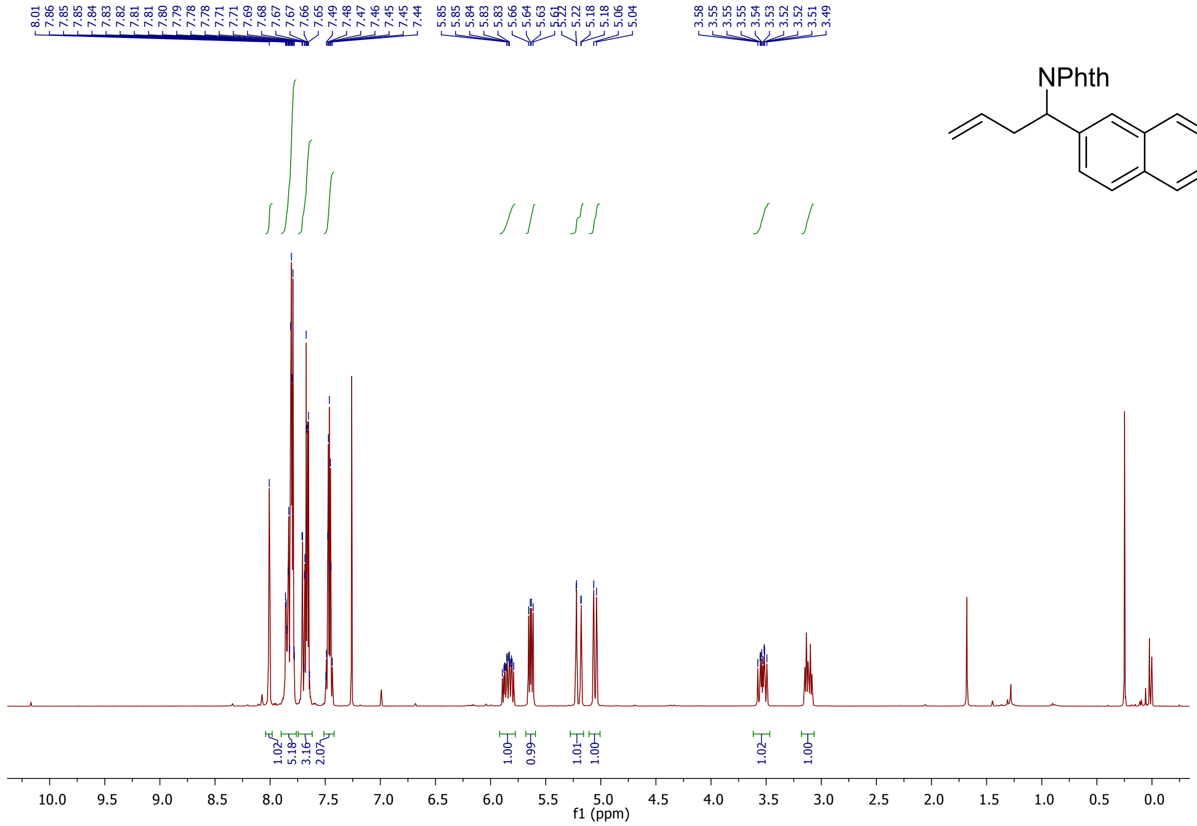

$^{13}C\{^{1}H\}$ NMR (101 MHz, $CDCl_3$)

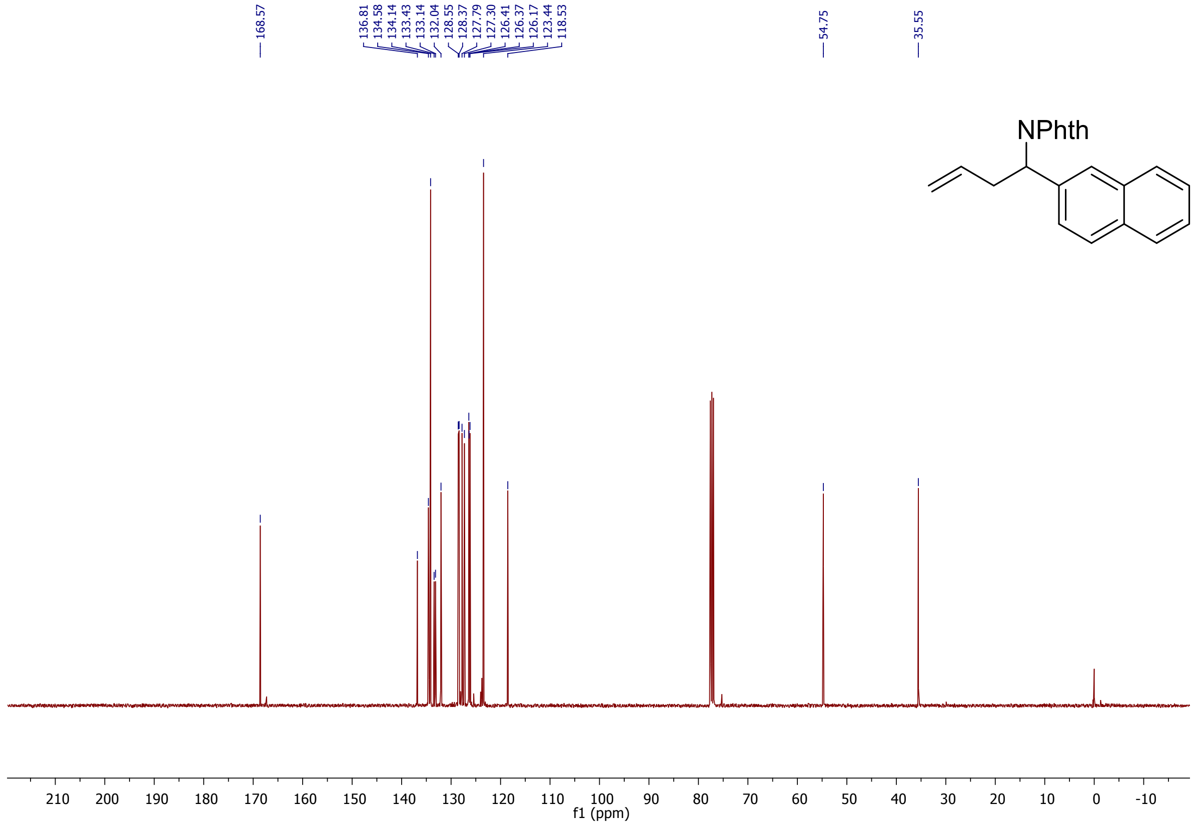

2-(1-(furan-2-yl)but-3-en-1-yl)isoindoline-1,3-dione (2m)

^{1}H NMR (400 MHz, $CDCl_3$)

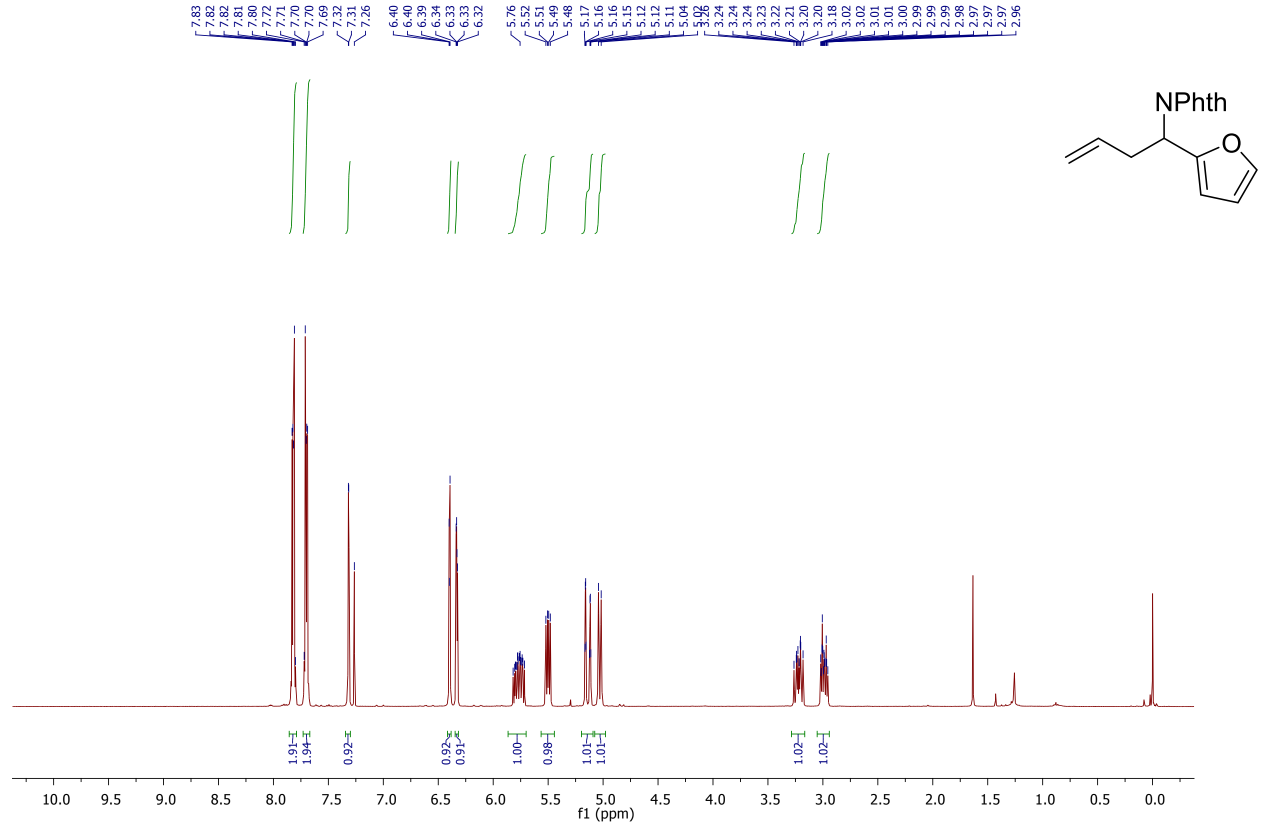

^{13}C{^{1}H} NMR (101 MHz, $CDCl_3$)

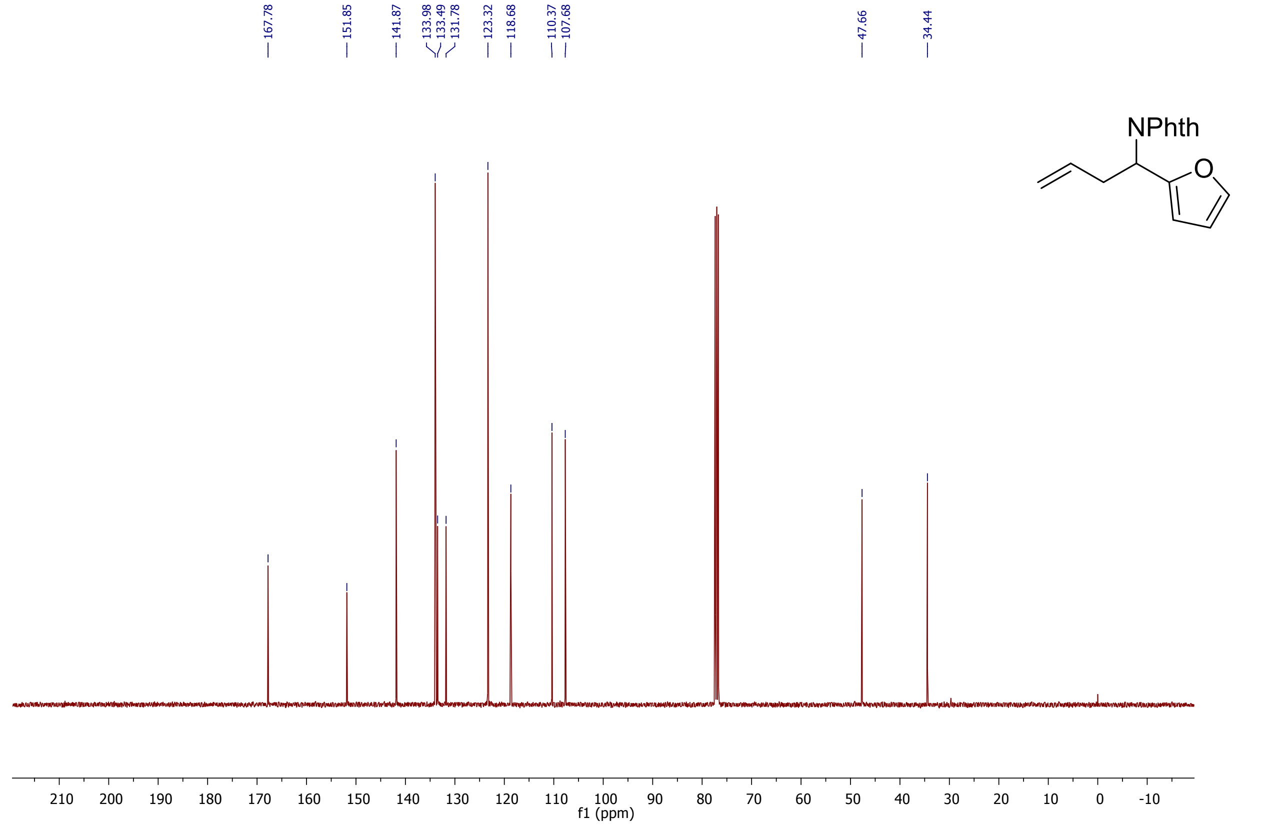

2-(1-(thiophen-2-yl)but-3-en-1-yl)isoindoline-1,3-dione (2n)

^{1}H NMR (400 MHz, $CDCl_3$)

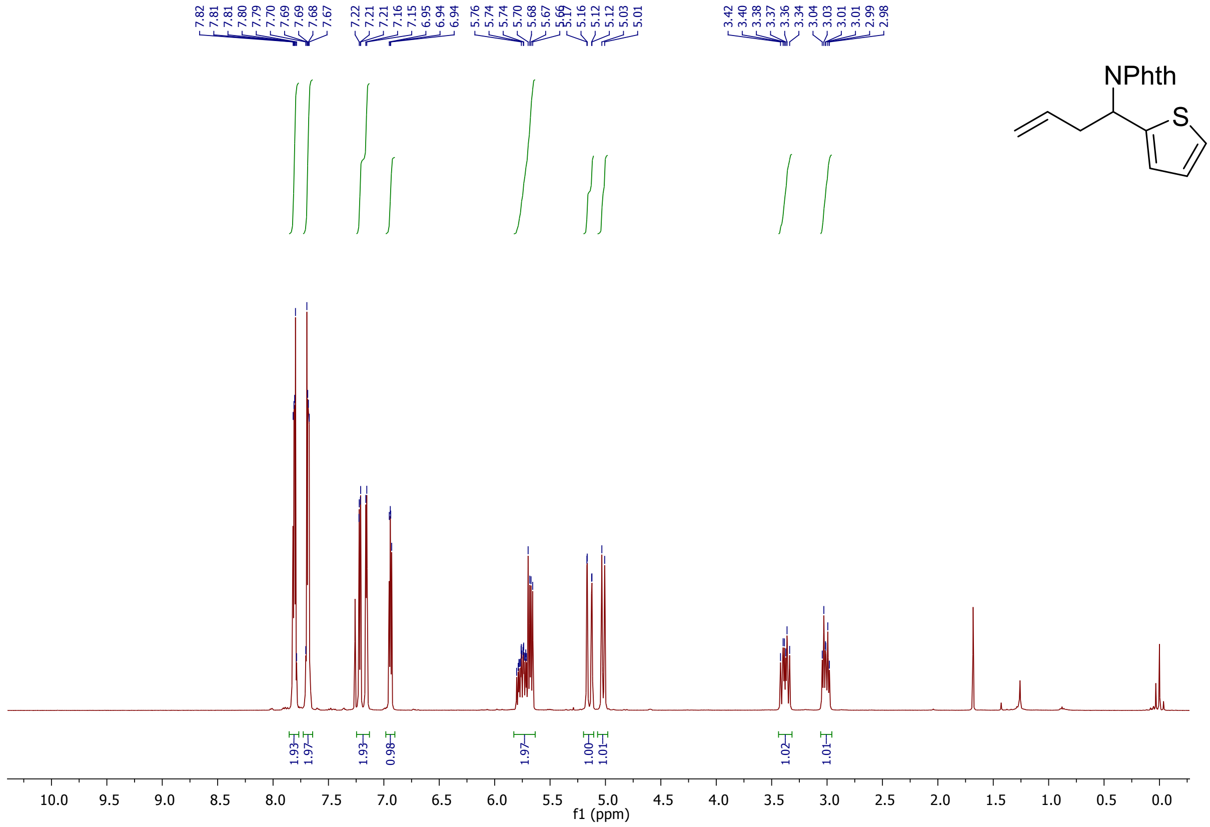

$^{13}C\{^{1}H\}$ NMR (101 MHz, $CDCl_3$)

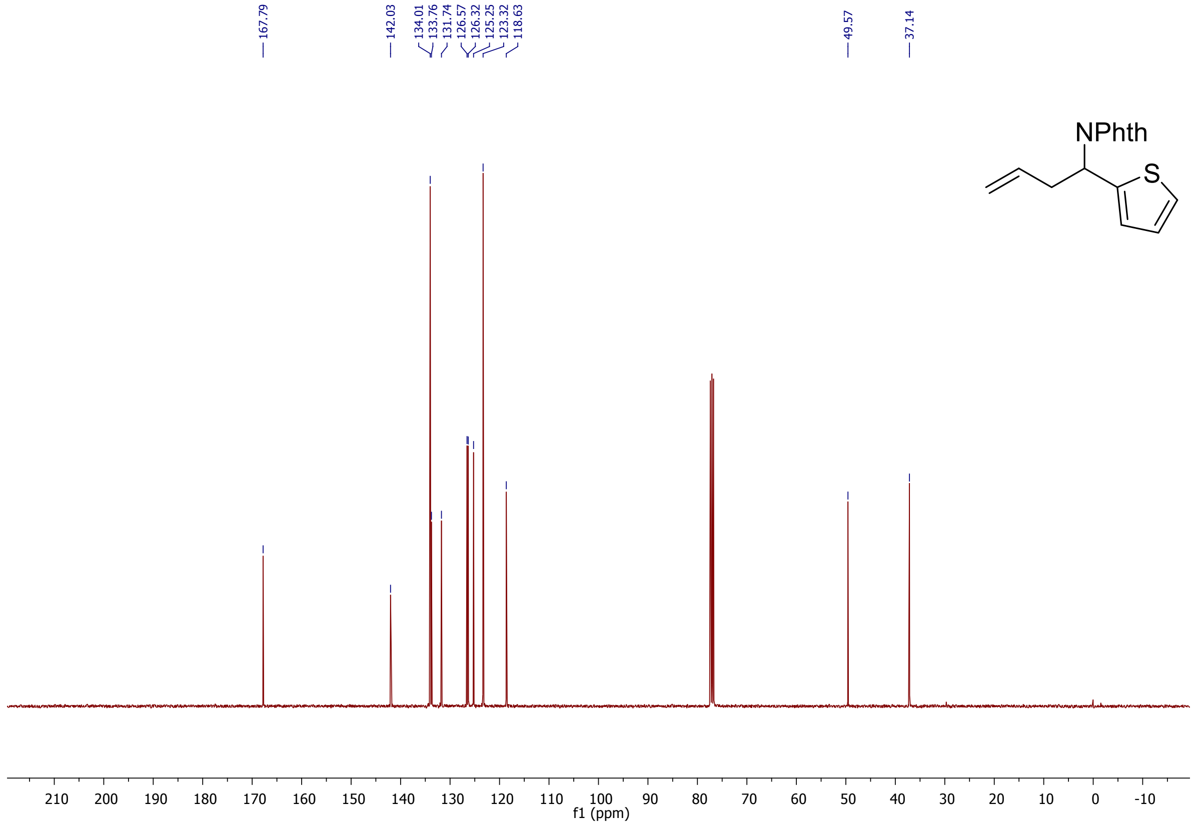

2-(1-cyclohexylbut-3-en-1-yl)isoindoline-1,3-dione (2o)

^{1}H NMR (400 MHz, $CDCl_3$)

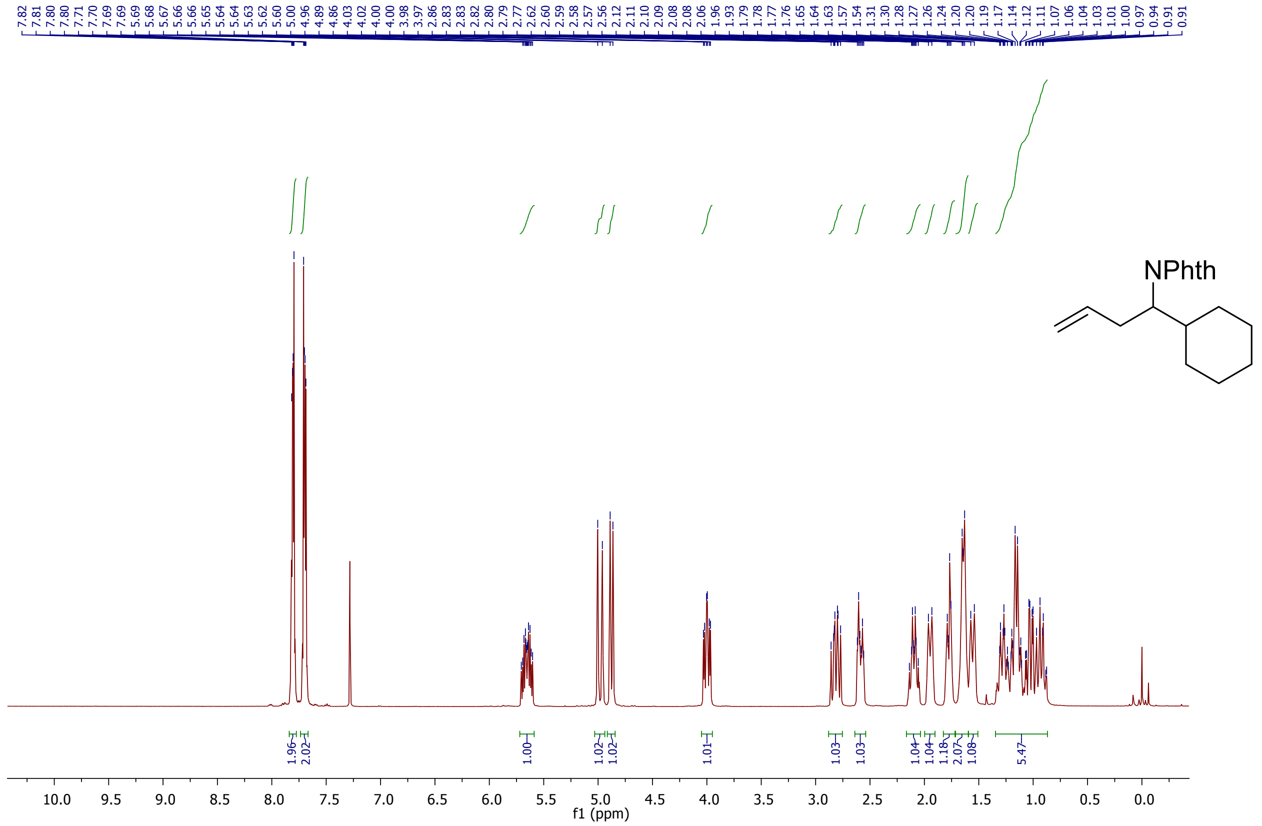

$^{13}C\{^{1}H\}$ NMR (101 MHz, $CDCl_3$)

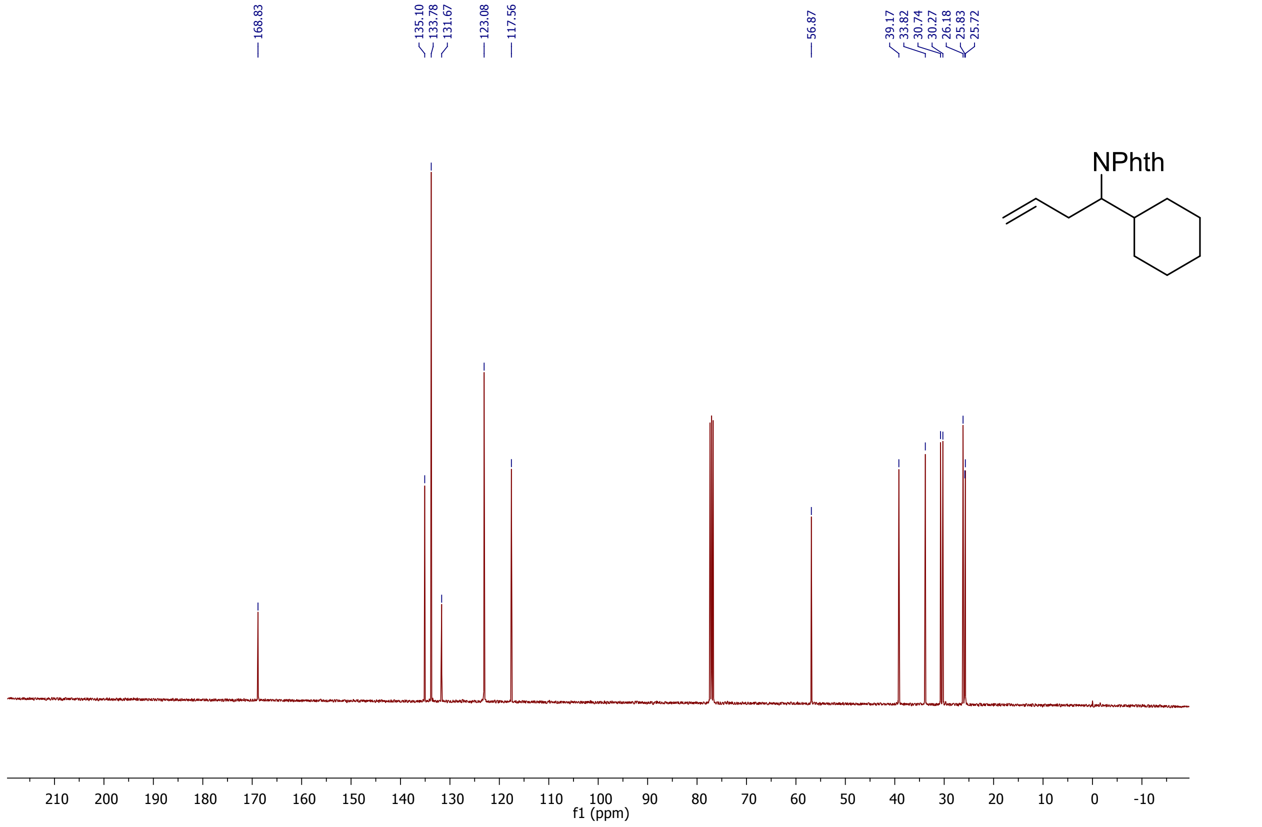

2-(phenyl(phenylthio)methyl)isoindoline-1,3-dione (3a)

^{1}H NMR (400 MHz, $CDCl_3$)

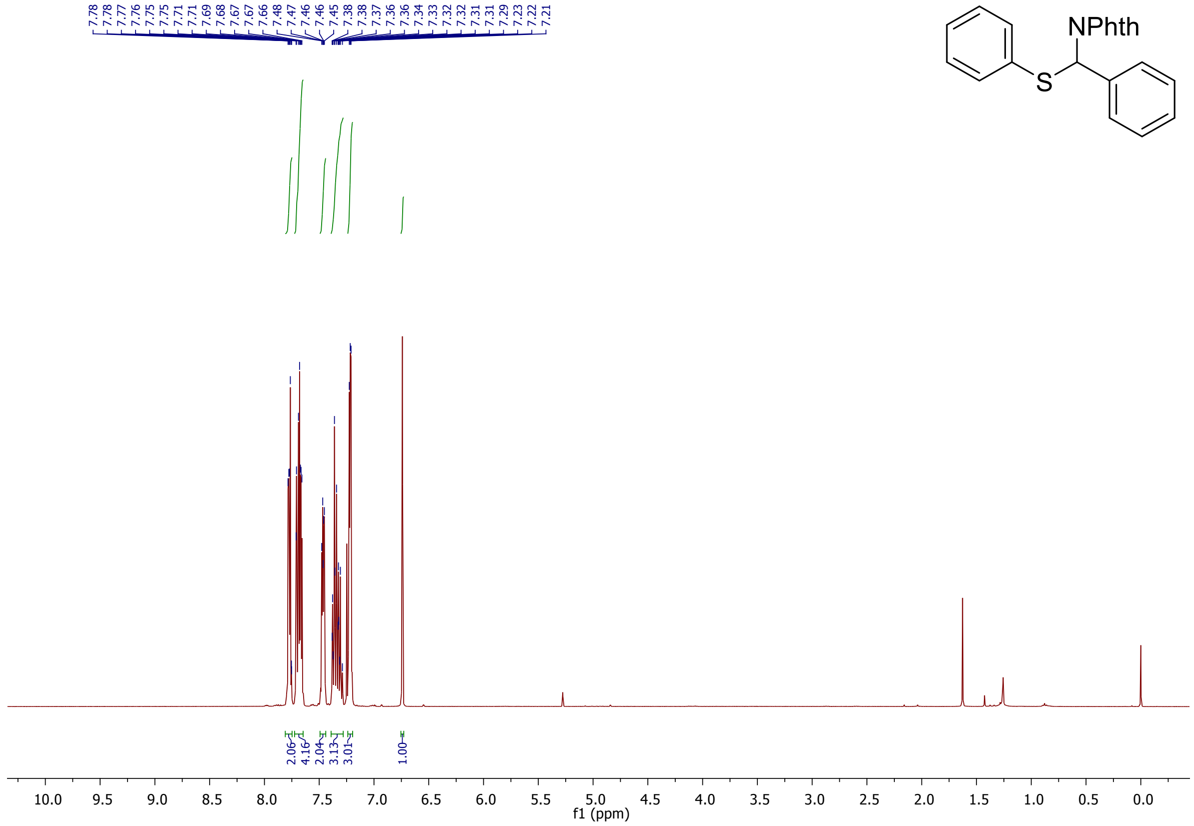

^{13}C{^{1}H} NMR (101 MHz, $CDCl_3$)

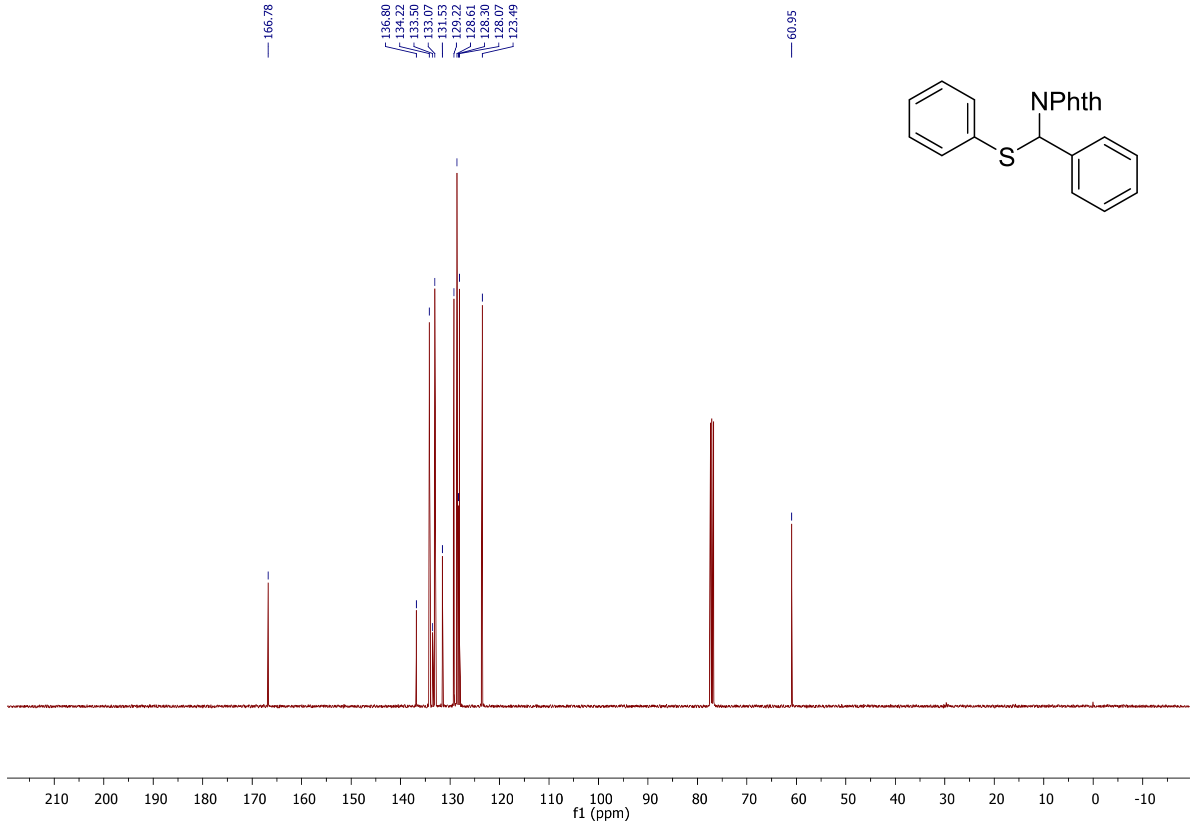

2-((3-bromo-2-fluorophenyl)(phenylthio)methyl)isoindoline-1,3-dione (3b)

^{1}H NMR (400 MHz, $CDCl_3$)

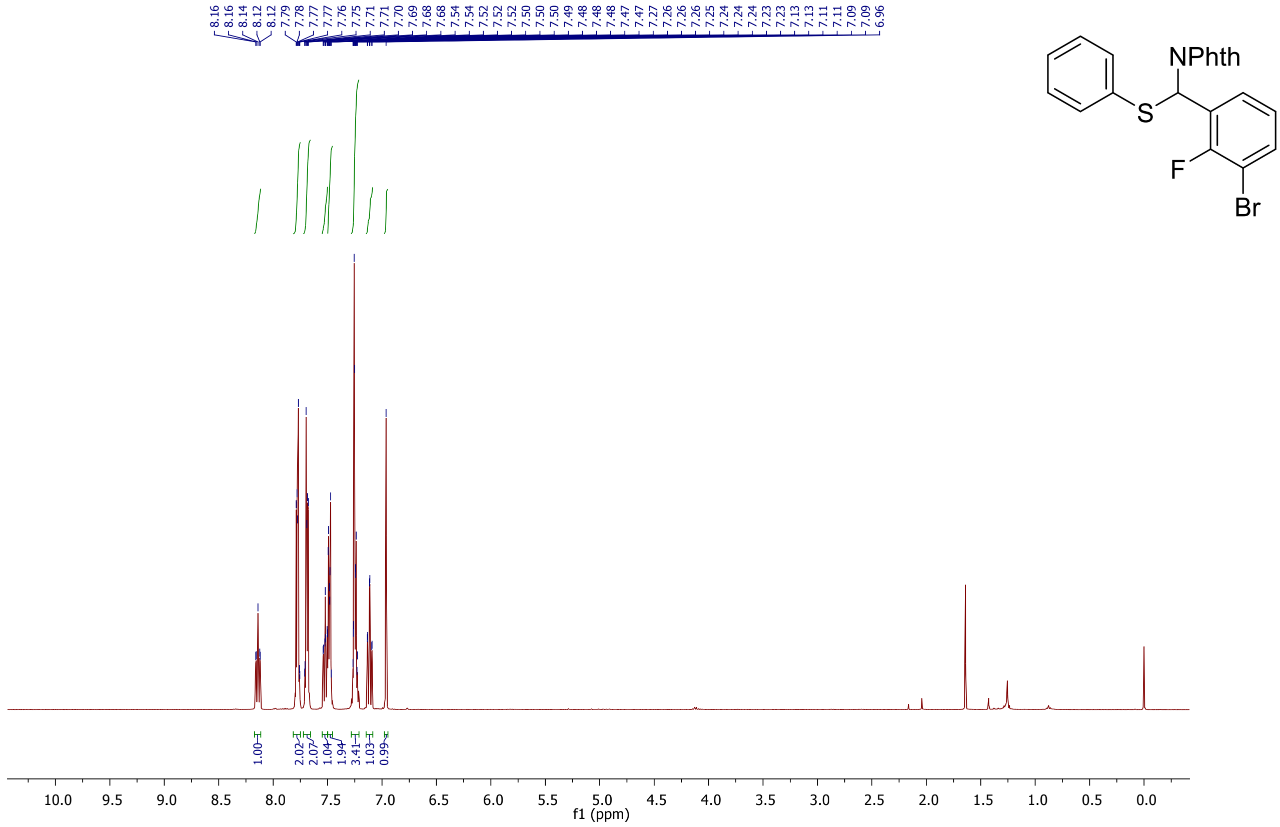

$^{13}C\{^{1}H\}$ NMR (101 MHz, $CDCl_3$)

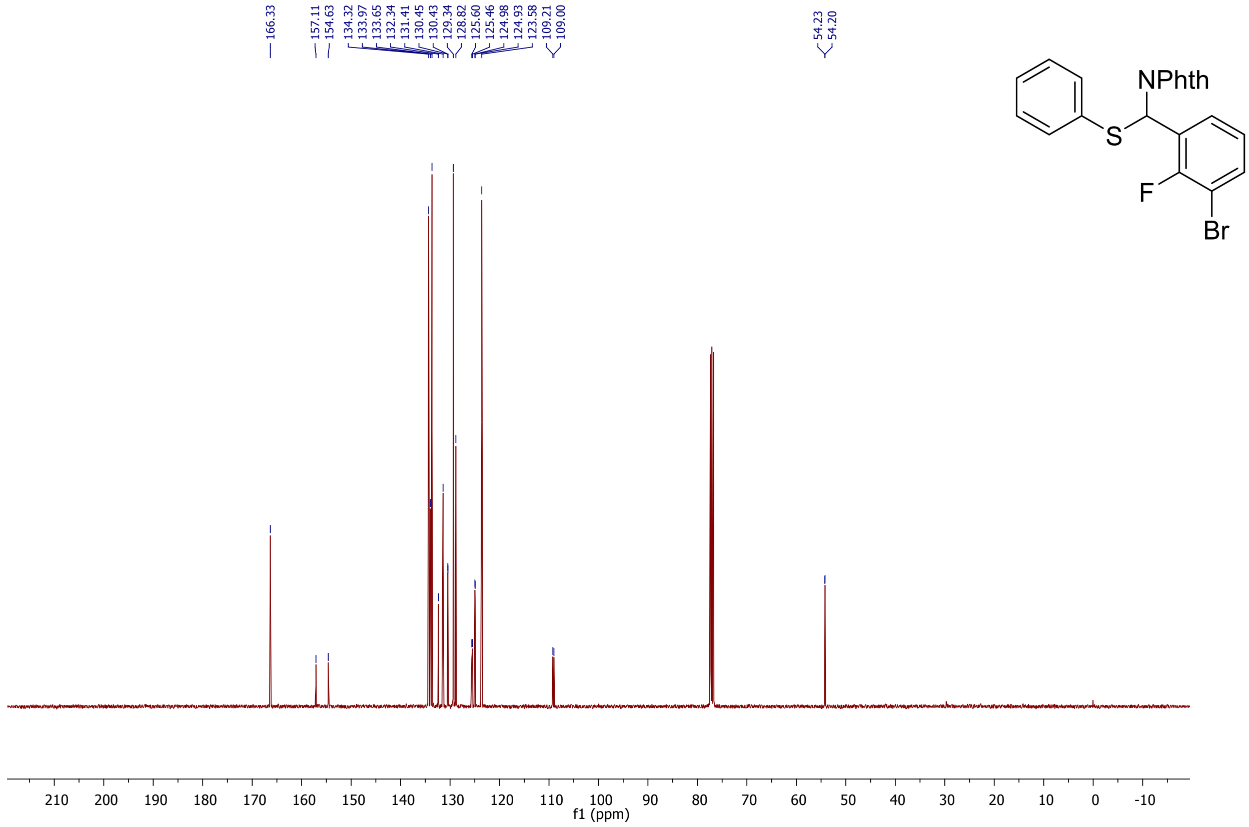

2-((4-nitrophenyl)(phenylthio)methyl)isoindoline-1,3-dione (3c)

^{1}H NMR (400 MHz, $CDCl_3$)

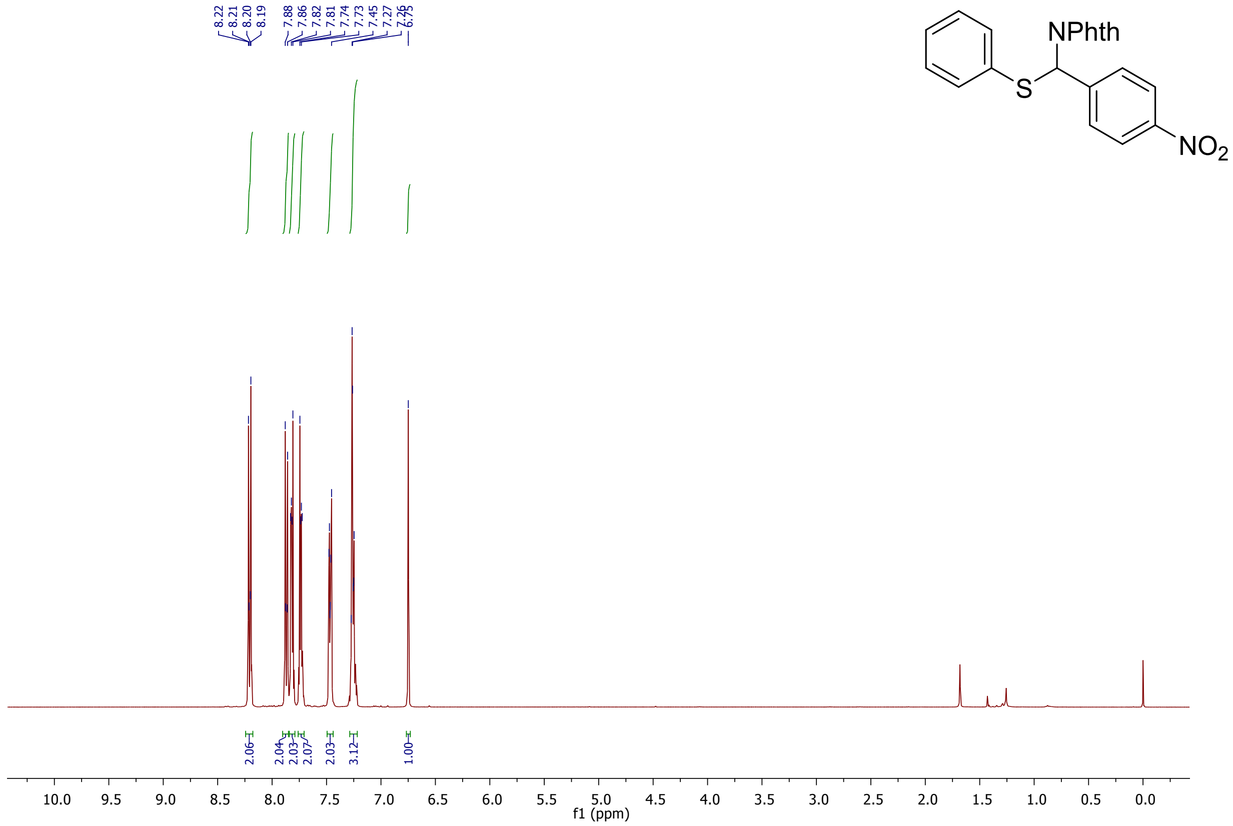

$^{13}C\{^{1}H\}$ NMR (101 MHz, $CDCl_3$)

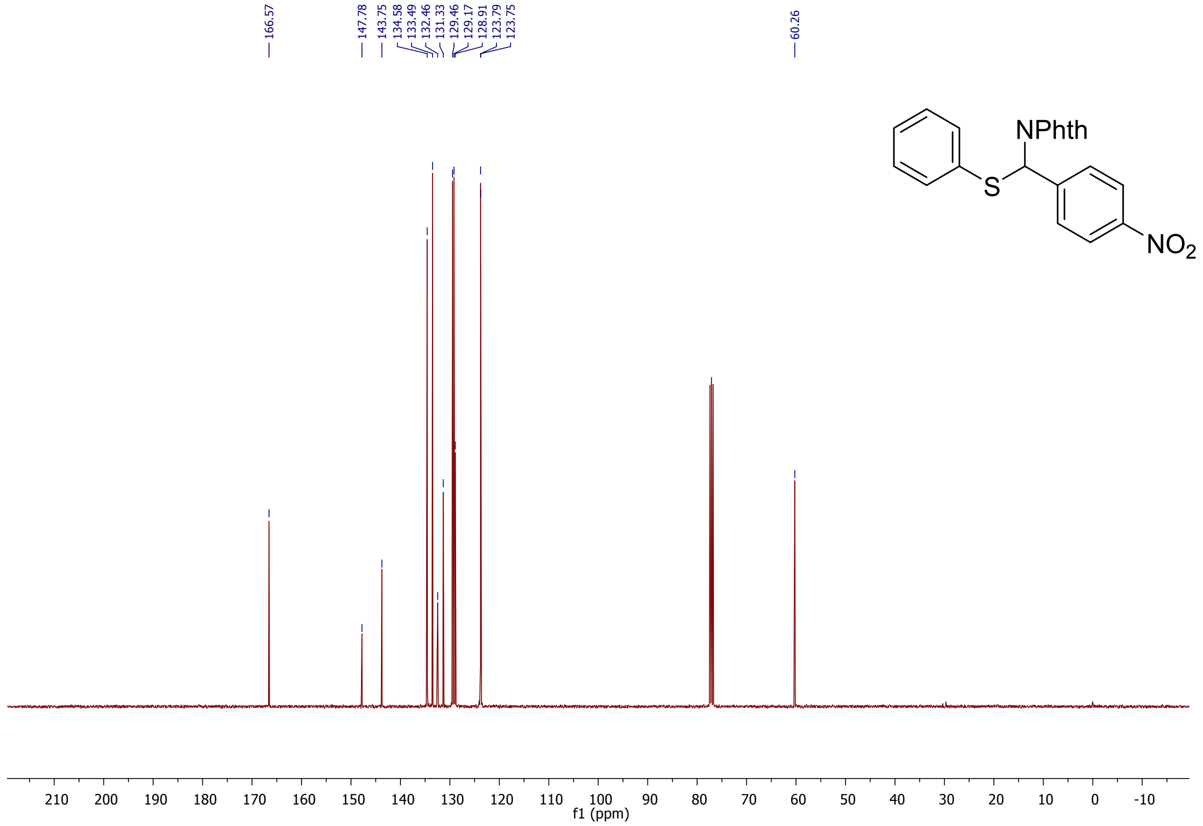

2-(((2-fluorophenyl)thio)(thiophen-2-yl)methyl)isoindoline-1,3-dione (3d)

^{1}H NMR (400 MHz, $CDCl_3$)

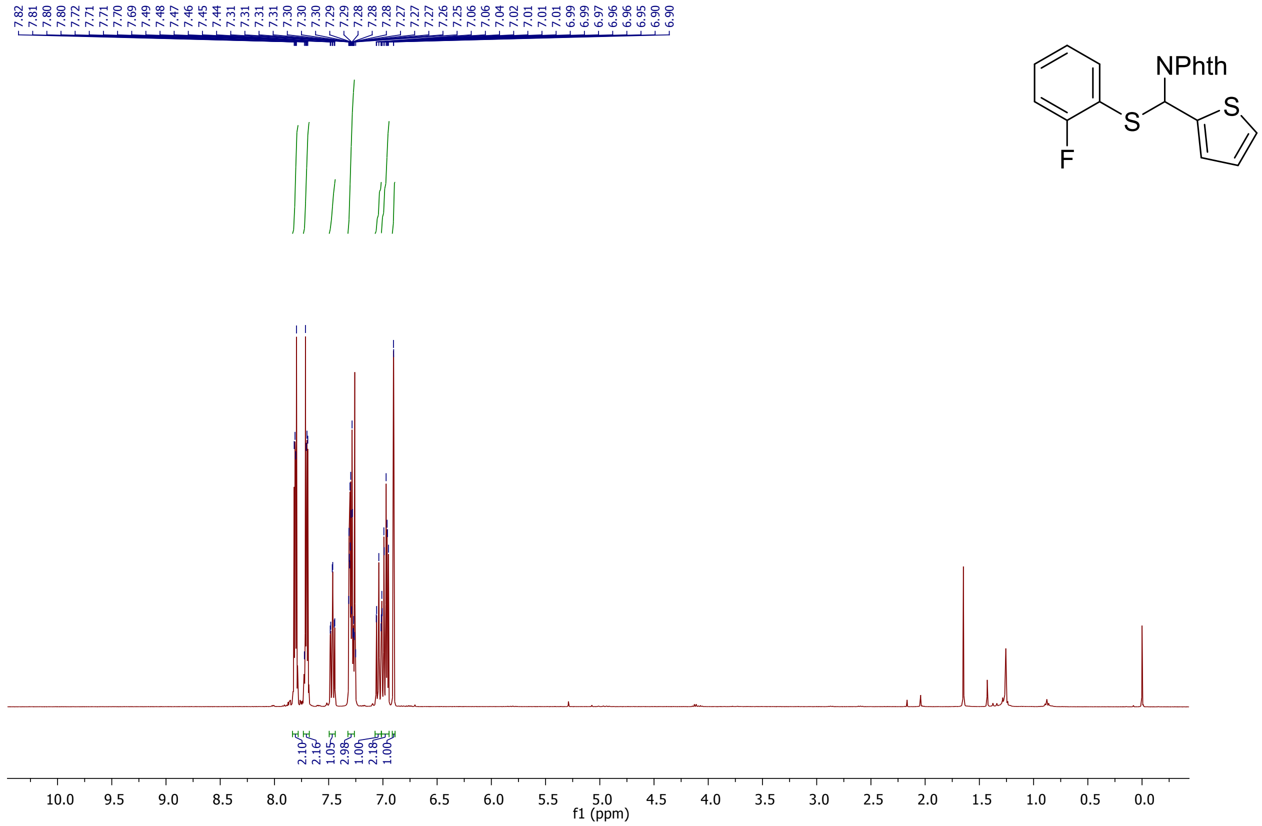

$^{13}C\{^{1}H\}$ NMR (101 MHz, $CDCl_3$)

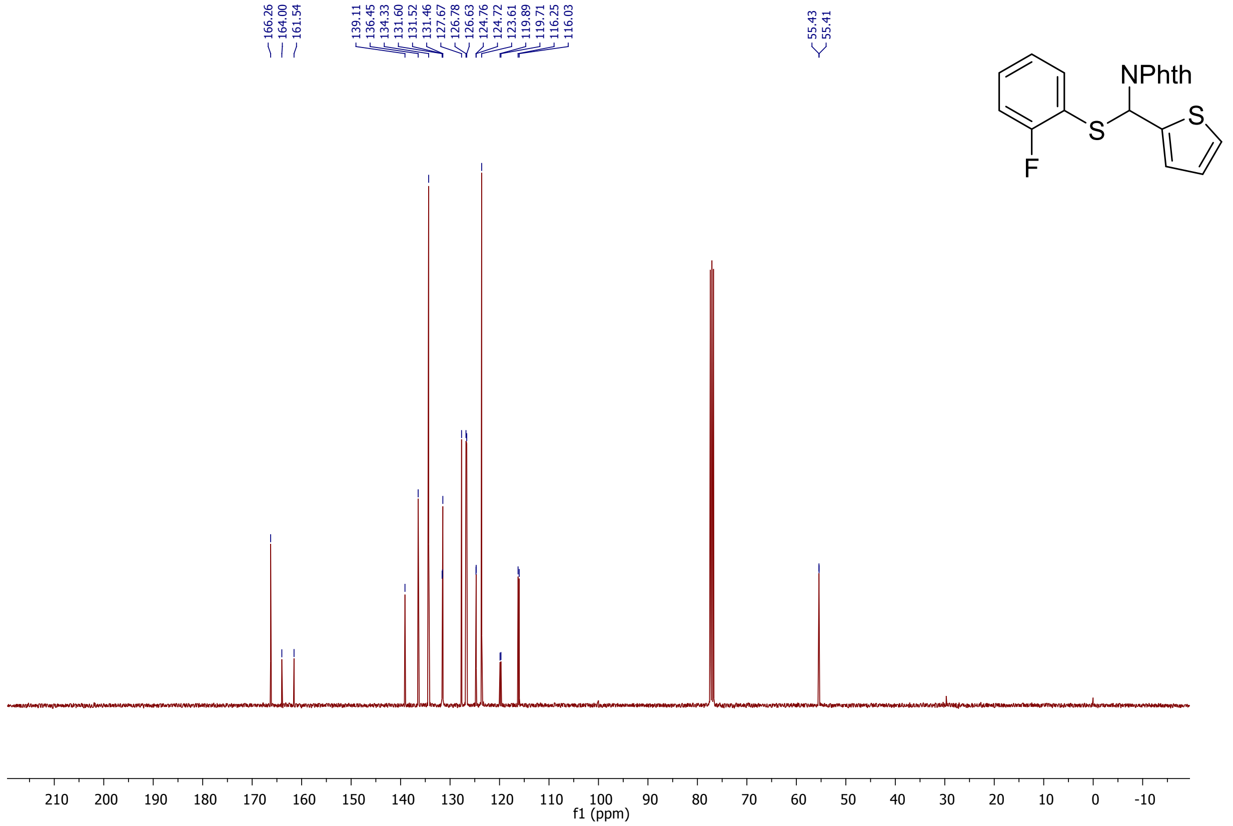

2-(cyclopropyl((4-methoxyphenyl)thio)methyl)isoindoline-1,3-dione (3e)

^{1}H NMR (400 MHz, $CDCl_3$)

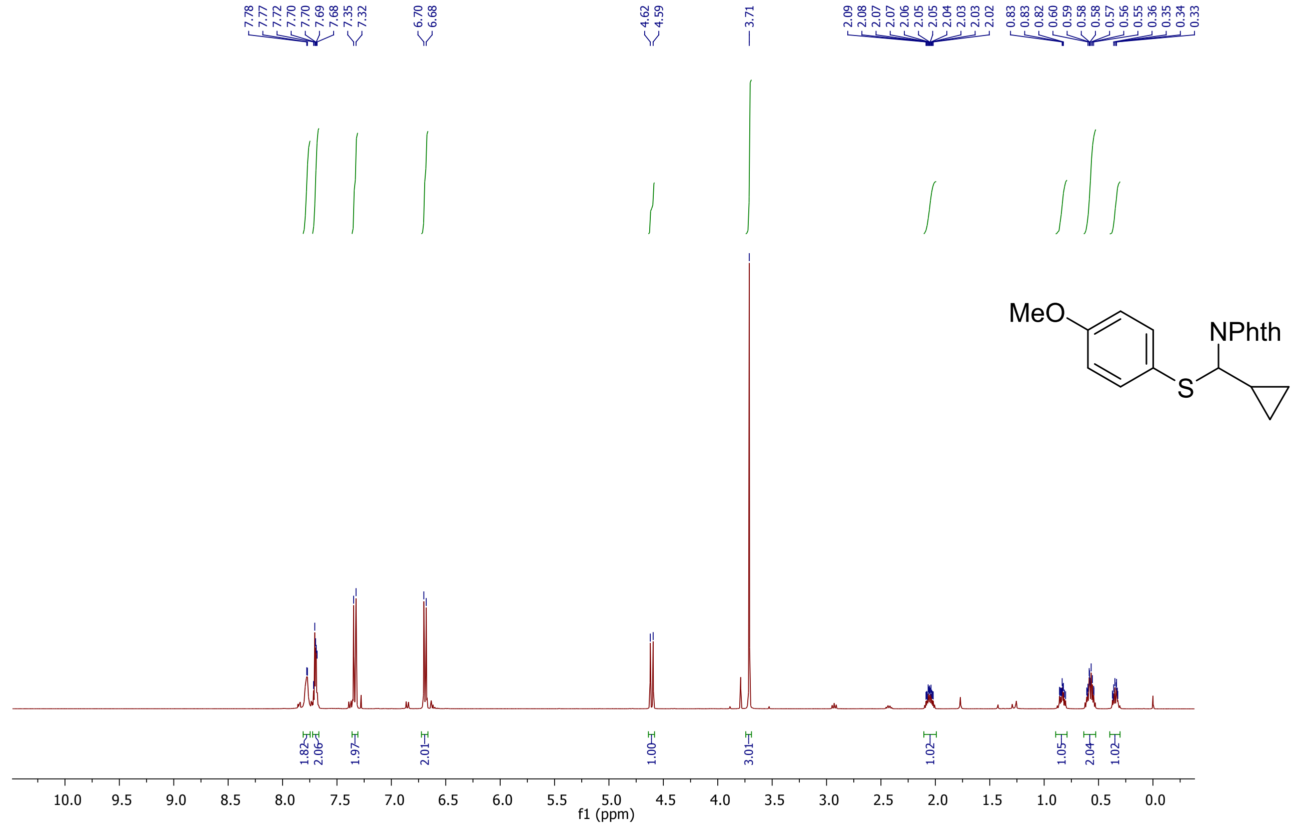

$^{13}C\{^{1}H\}$ NMR (101 MHz, $CDCl_3$)

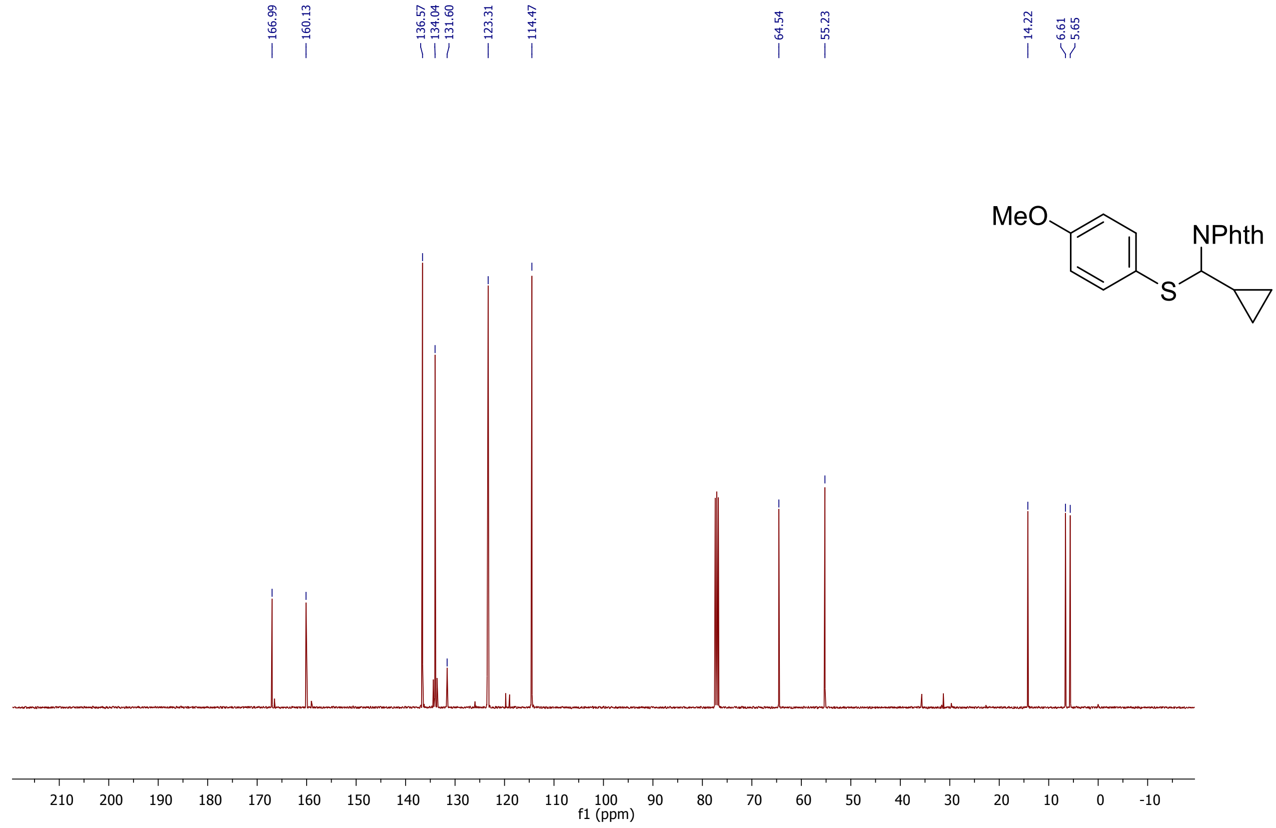

2-((ethylthio)(4-methoxyphenyl)methyl)isoindoline-1,3-dione (3f)

^{1}H NMR (400 MHz, $CDCl_3$)

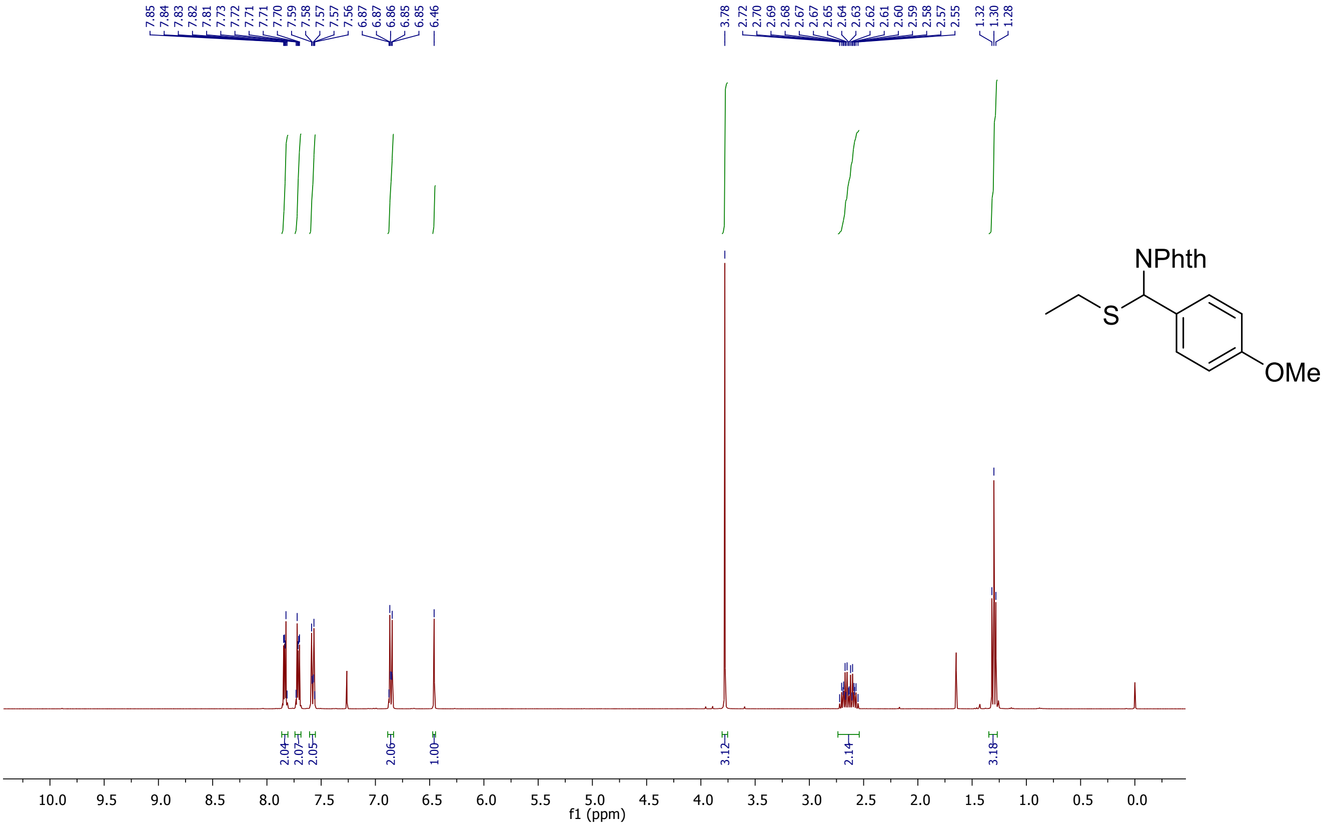

^{13}C{^{1}H} NMR (101 MHz, $CDCl_3$)

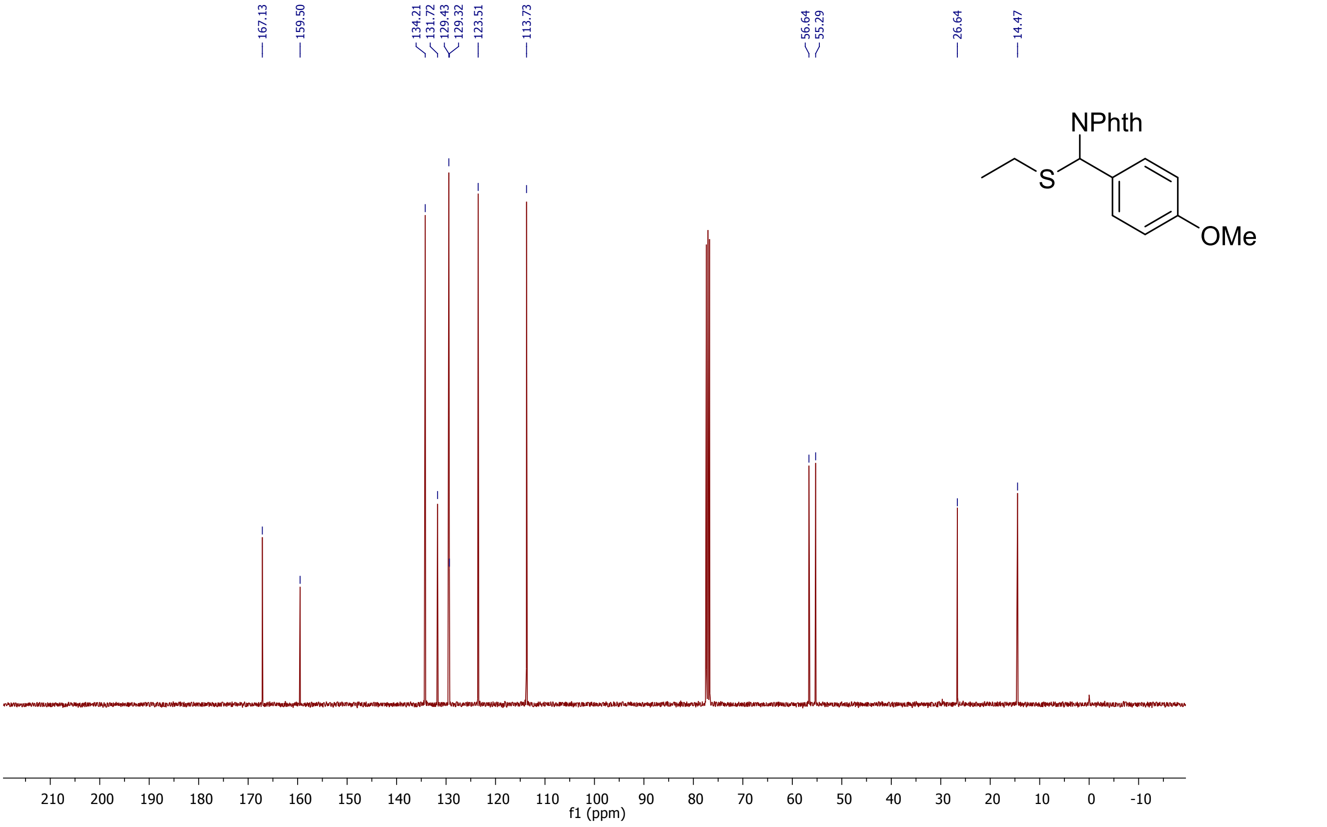

2-((5-bromo-1-methyl-1H-indol-3-yl)(phenyl)methyl)isoindoline-1,3-dione (3g)

^{1}H NMR (400 MHz, $CDCl_3$)

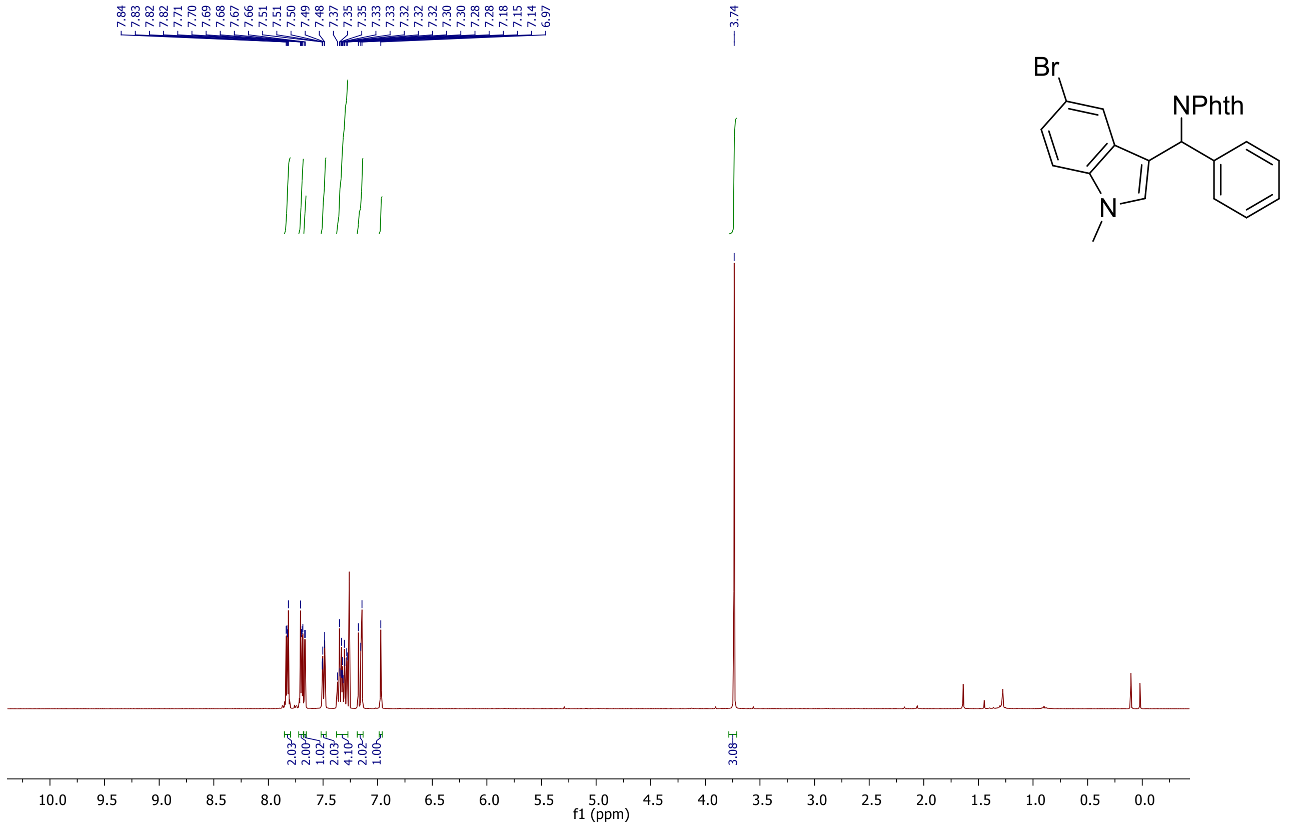

^{13}C{^{1}H} NMR (101 MHz, $CDCl_3$)

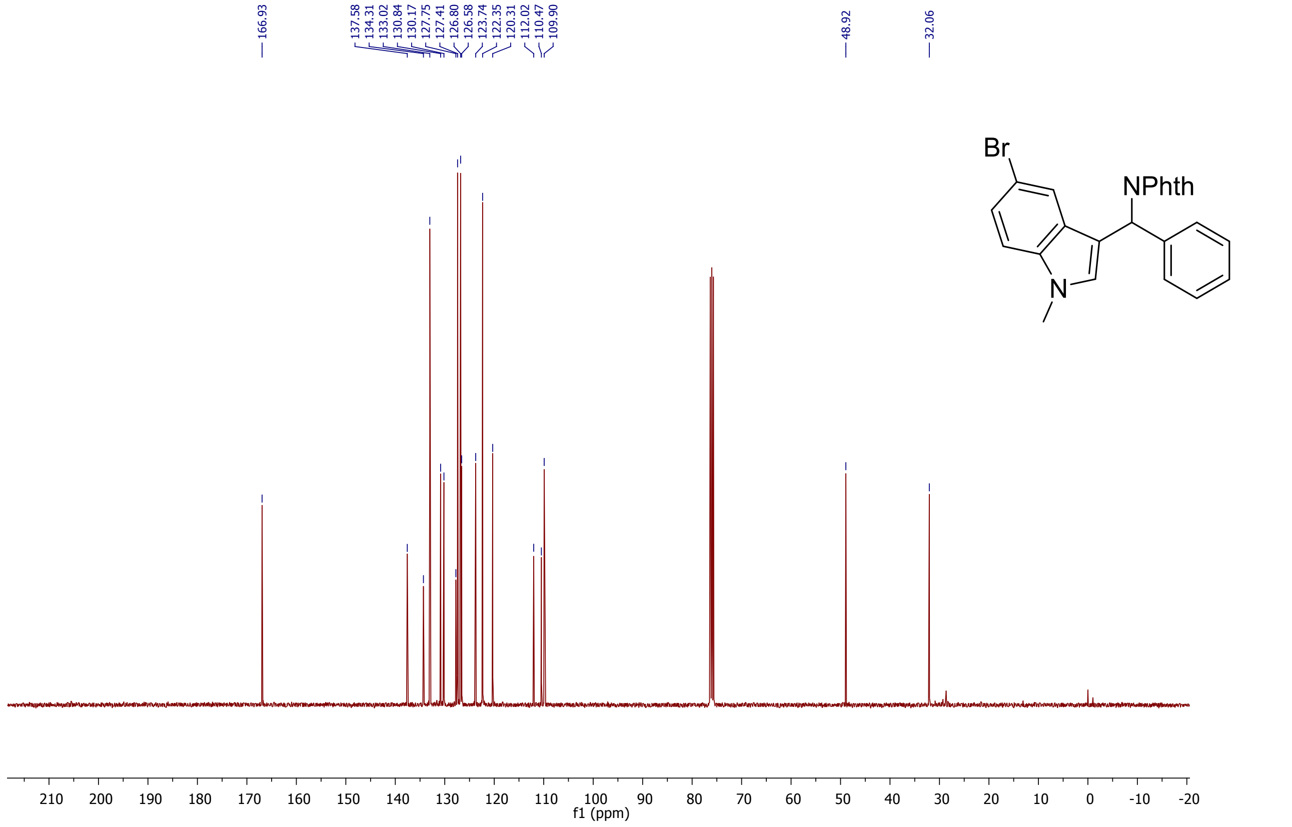

2-((5-bromo-1-methyl-1H-indol-3-yl)(2-methoxyphenyl)methyl)isoindoline-1,3-dione (3h)

^{1}H NMR (400 MHz, $CDCl_3$)

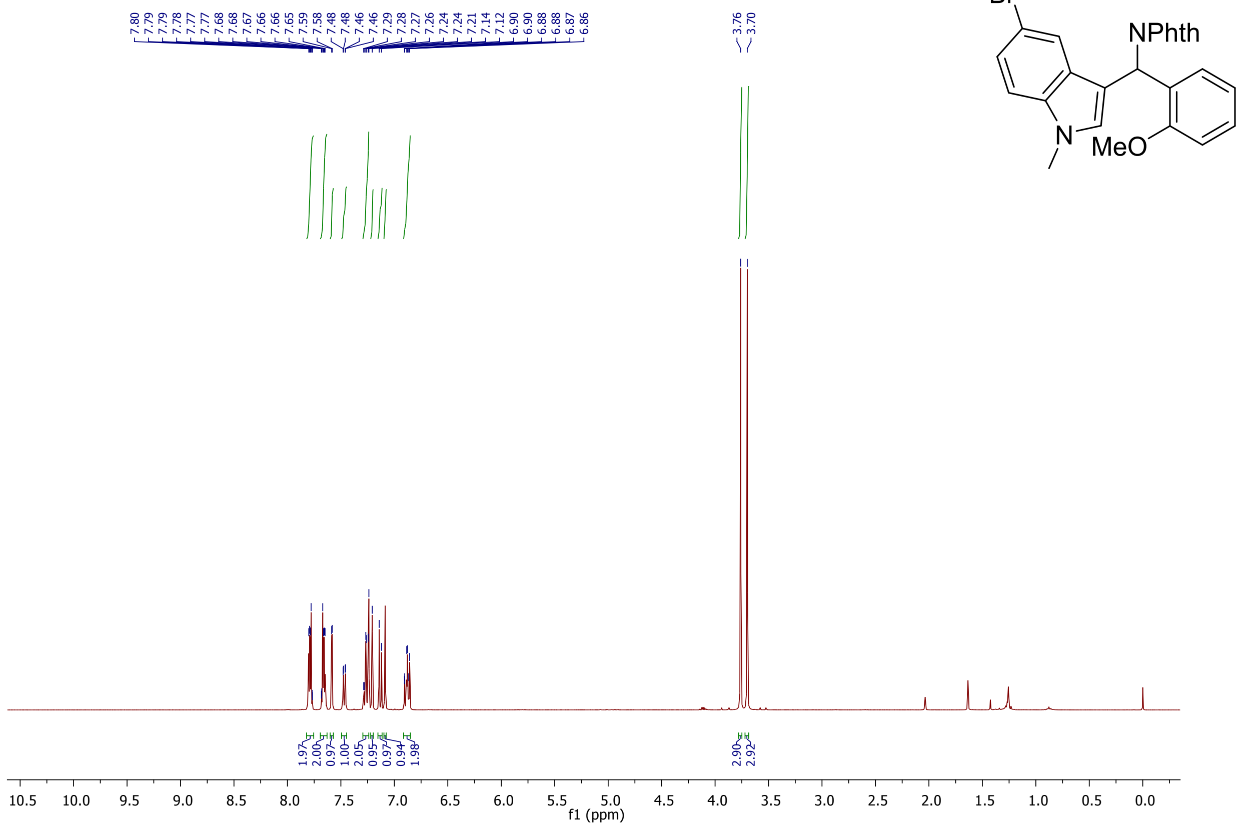

$^{13}C\{^1H\}$ NMR (101 MHz, $CDCl_3$)

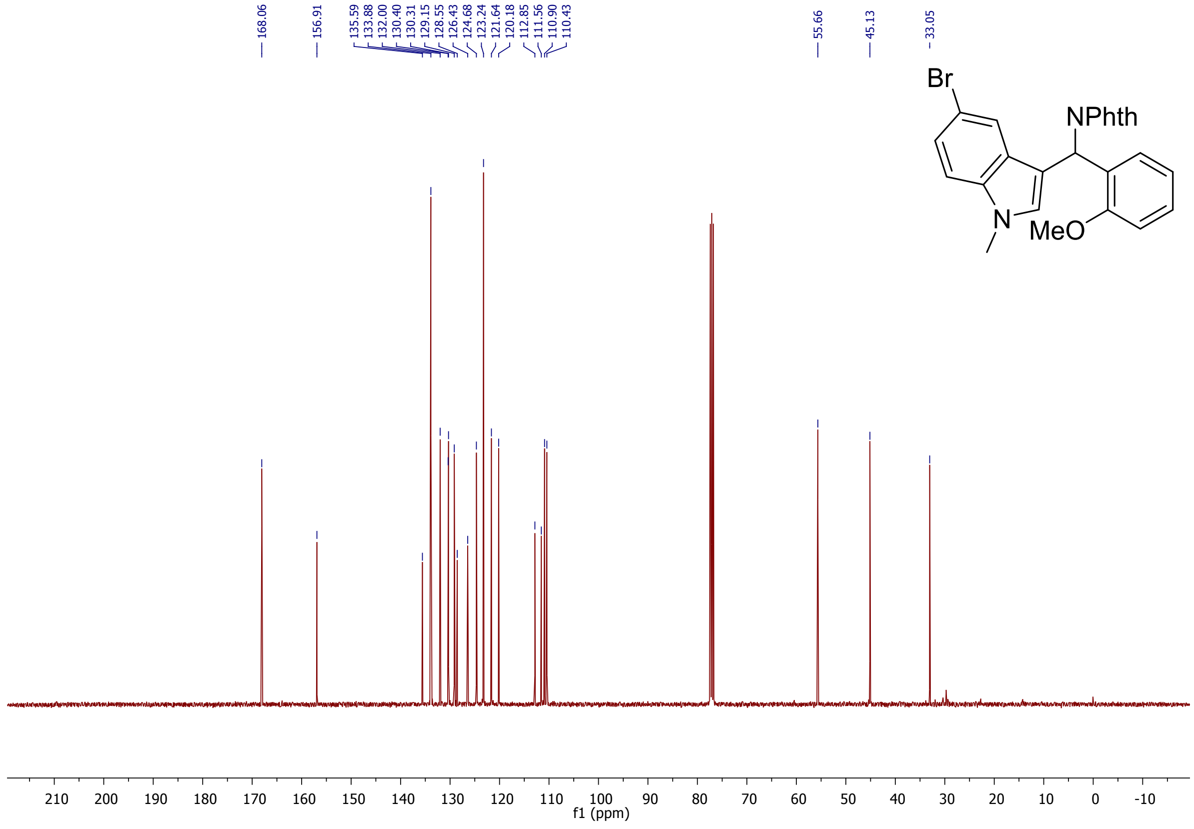

2-((5-bromo-1-methyl-1H-indol-3-yl)(furan-2-yl)methyl)isoindoline-1,3-dione (3i)

^{1}H NMR (400 MHz, $CDCl_3$)

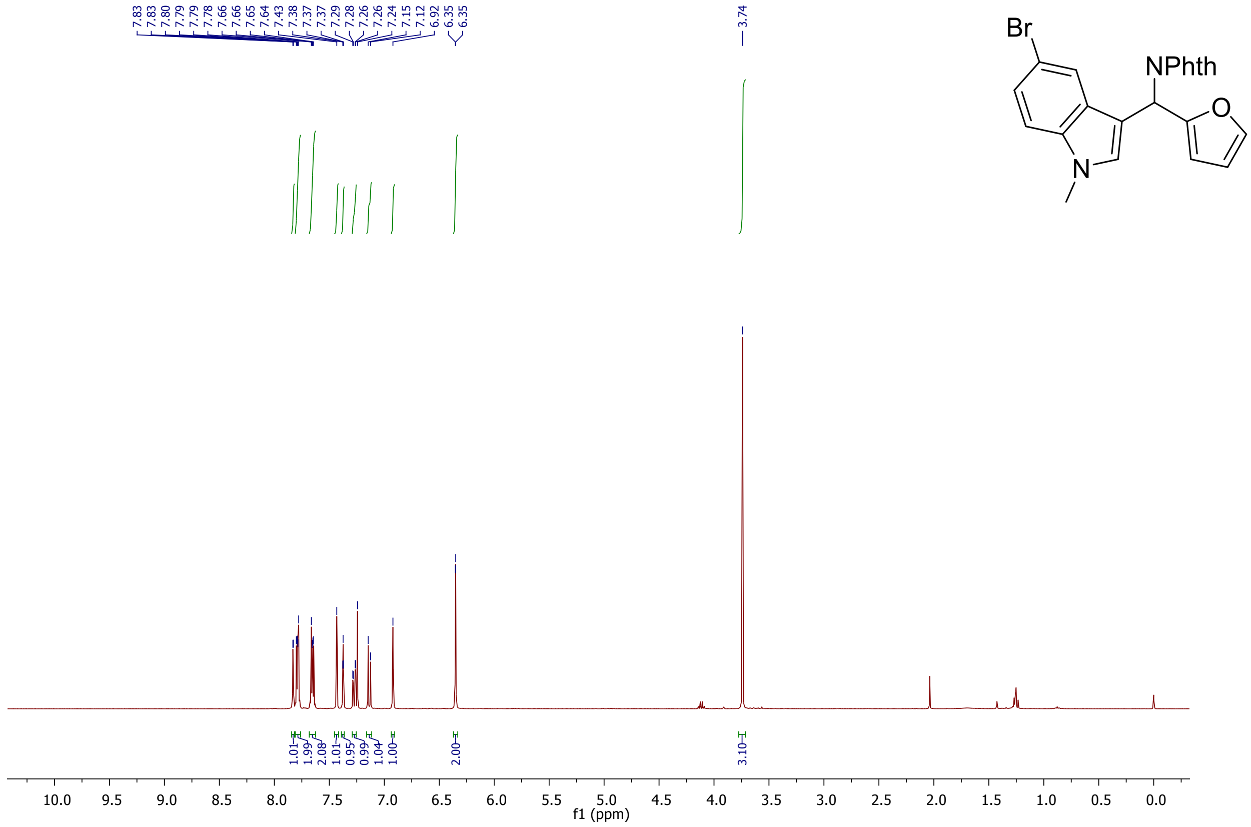

$^{13}C\{^{1}H\}$ NMR (101 MHz, $CDCl_3$)

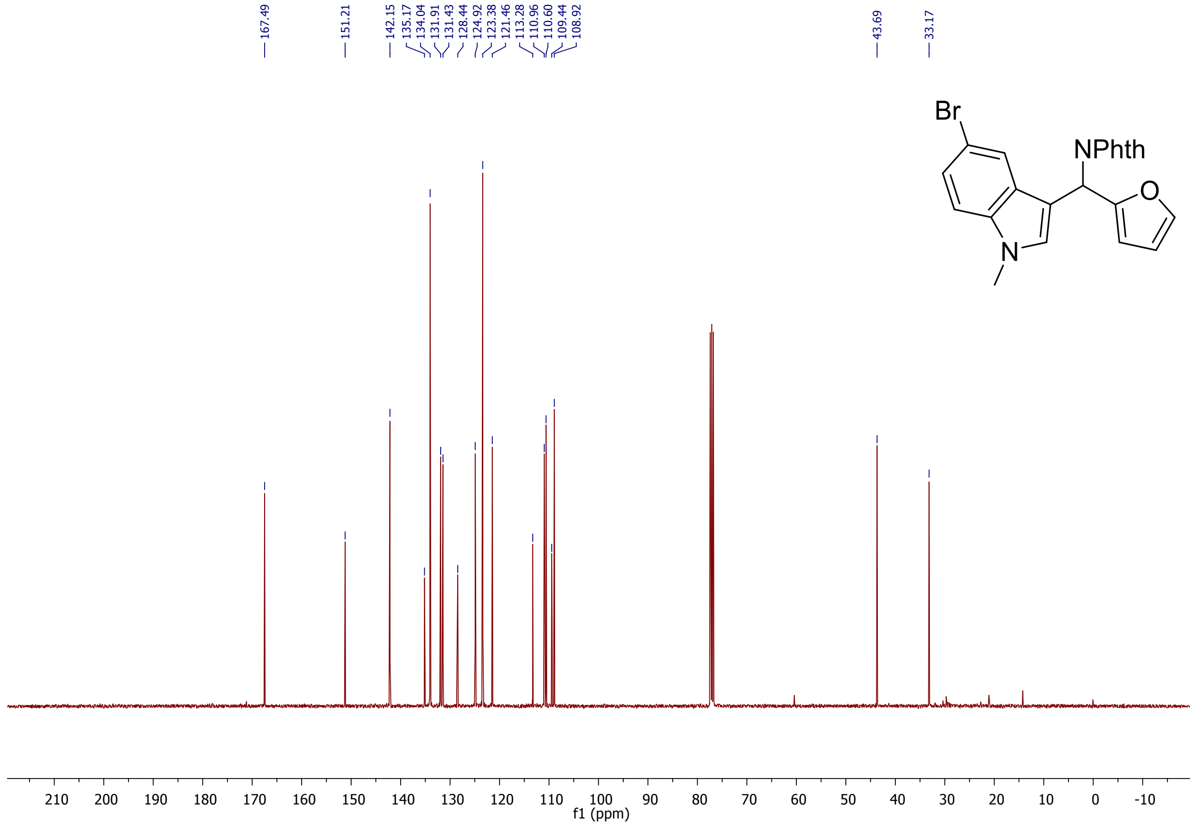

2-((3,5-bis(trifluoromethyl)phenyl)(5-bromo-1-methyl-1H-indol-3-yl)methyl) isoindoline-1,3-dione (3j)

^{1}H NMR (400 MHz, $CDCl_3$)

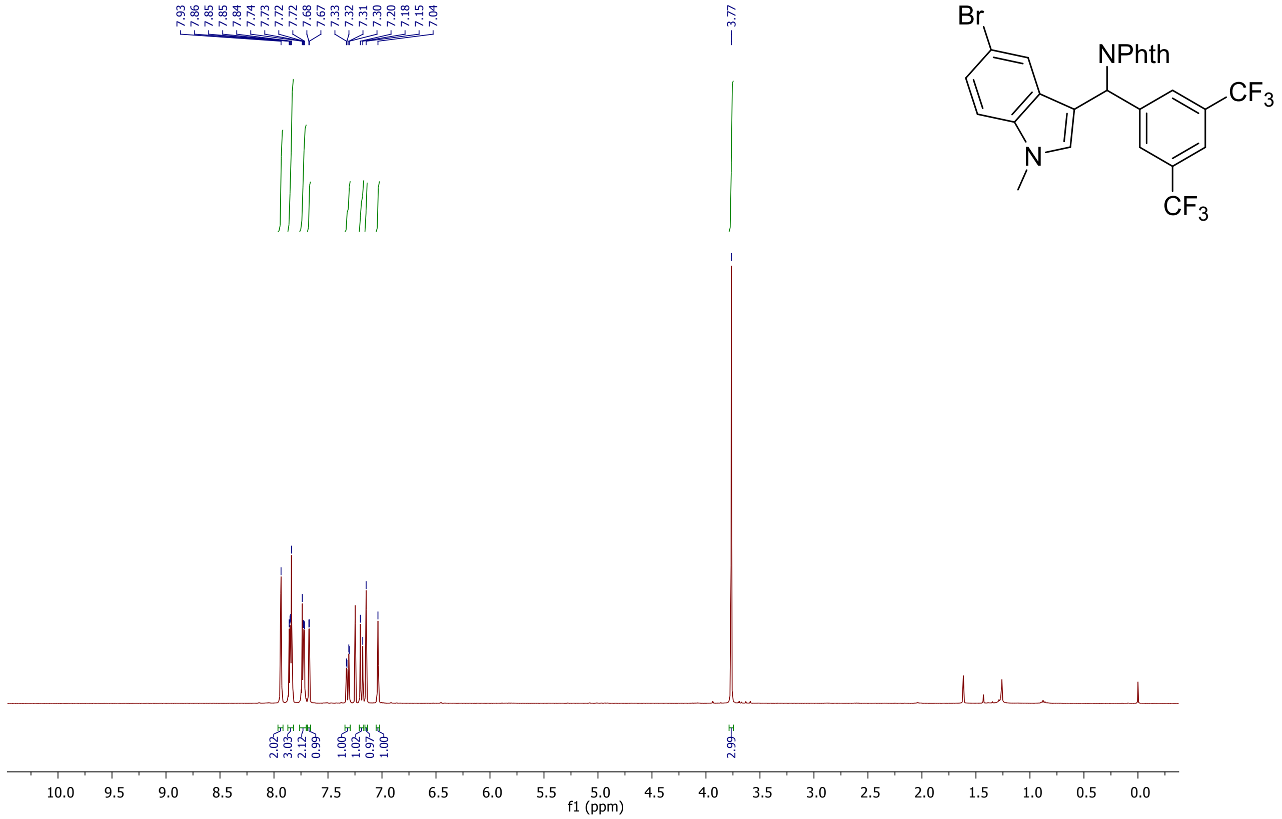

$^{13}C\{^{1}H\}$ NMR (101 MHz, $CDCl_3$)

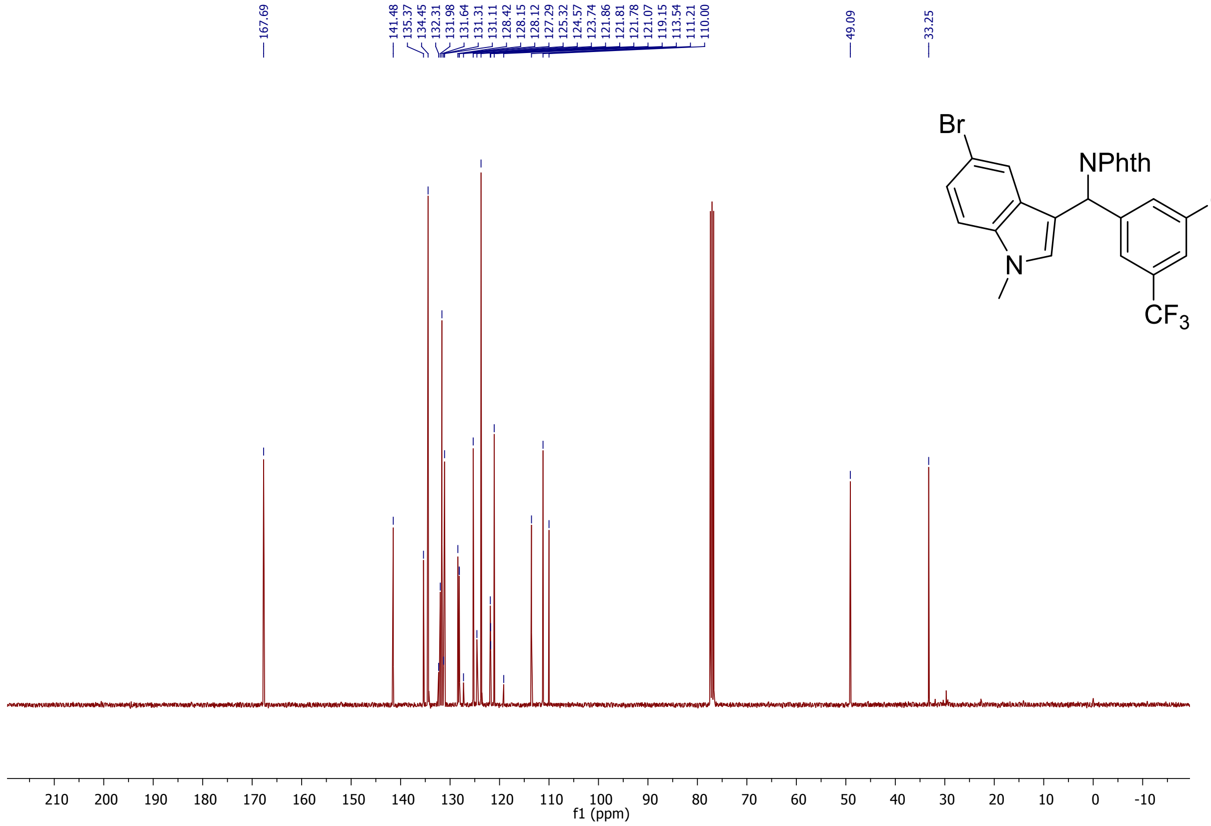

2-((5-bromo-1-methyl-1H-indol-3-yl)(cyclohexyl)methyl)isoindoline-1,3-dione (3k)

^{1}H NMR (400 MHz, CDCl$_3$)

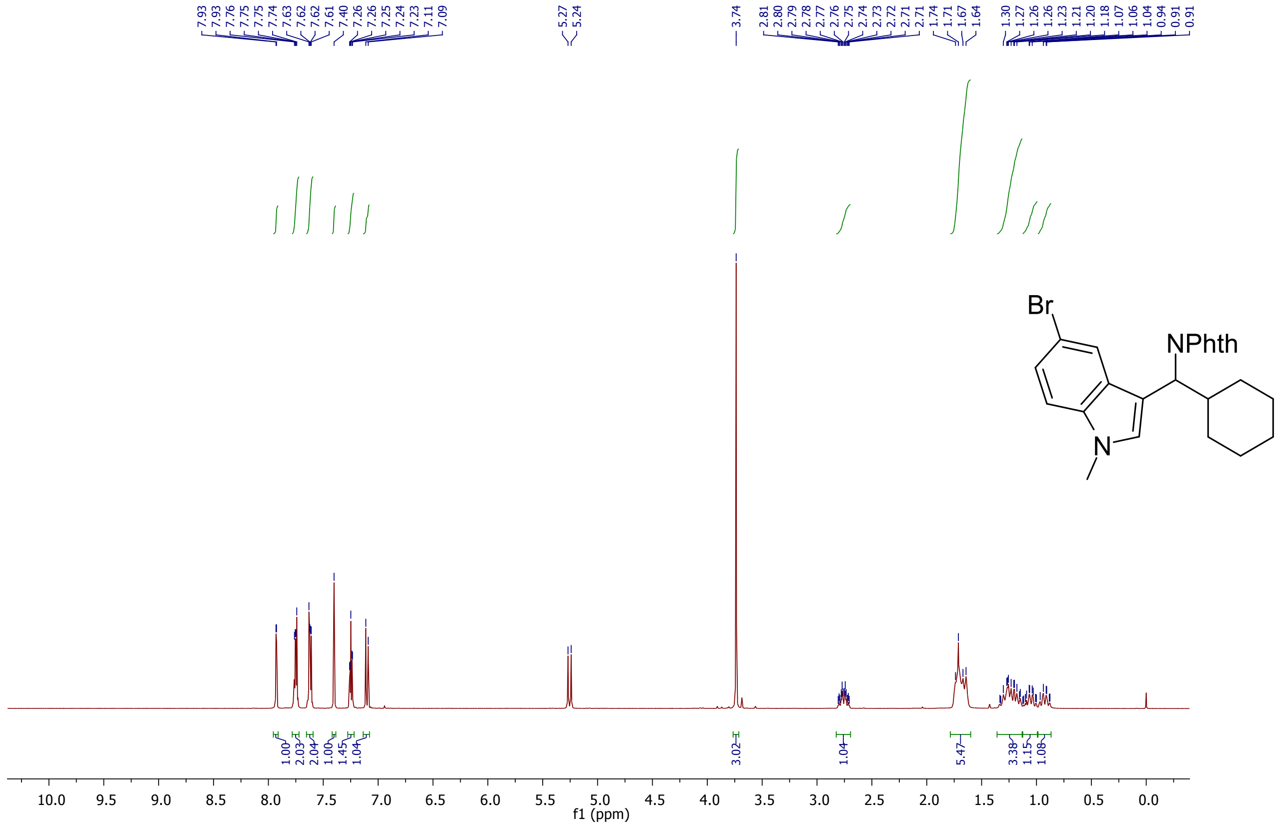

^{13}C{^{1}H} NMR (101 MHz, CDCl$_3$)

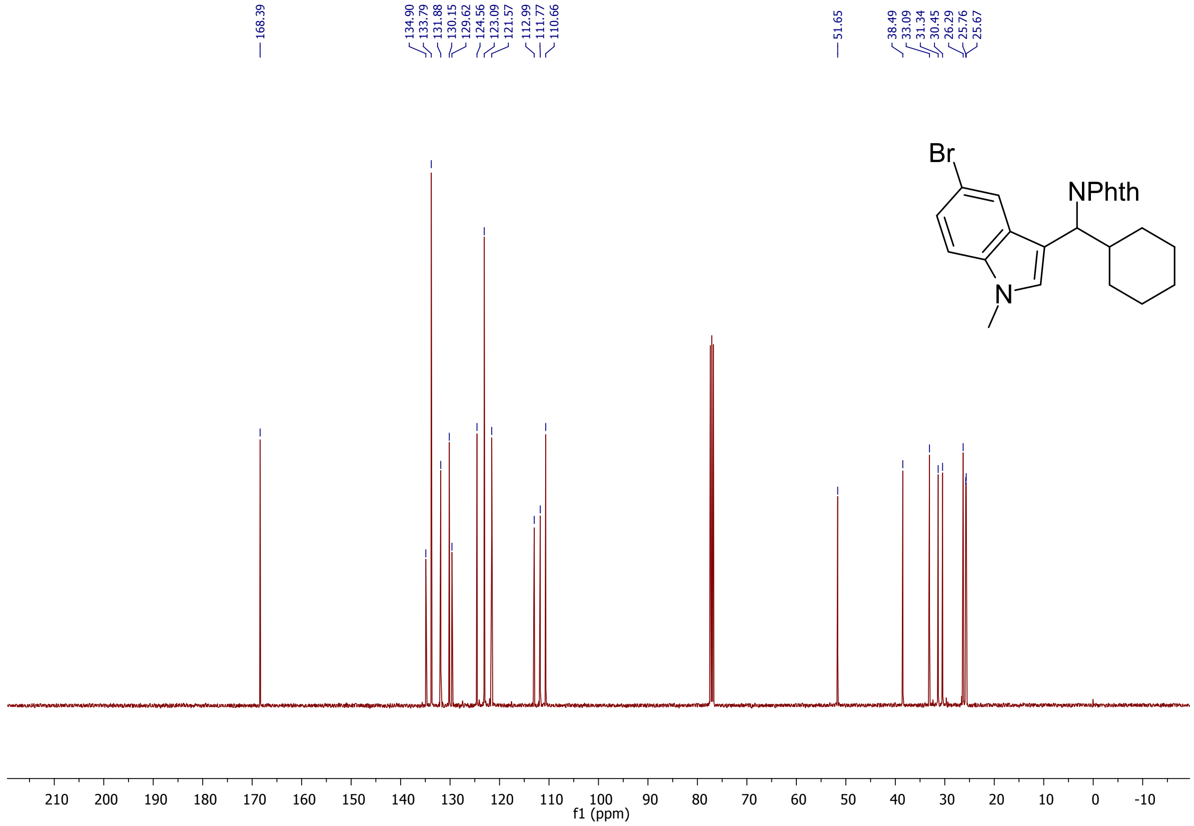

N-((1,3-dioxoisoindolin-2-yl)(phenyl)methyl)benzamide (3l)

^{1}H NMR (400 MHz, $CDCl_3$)

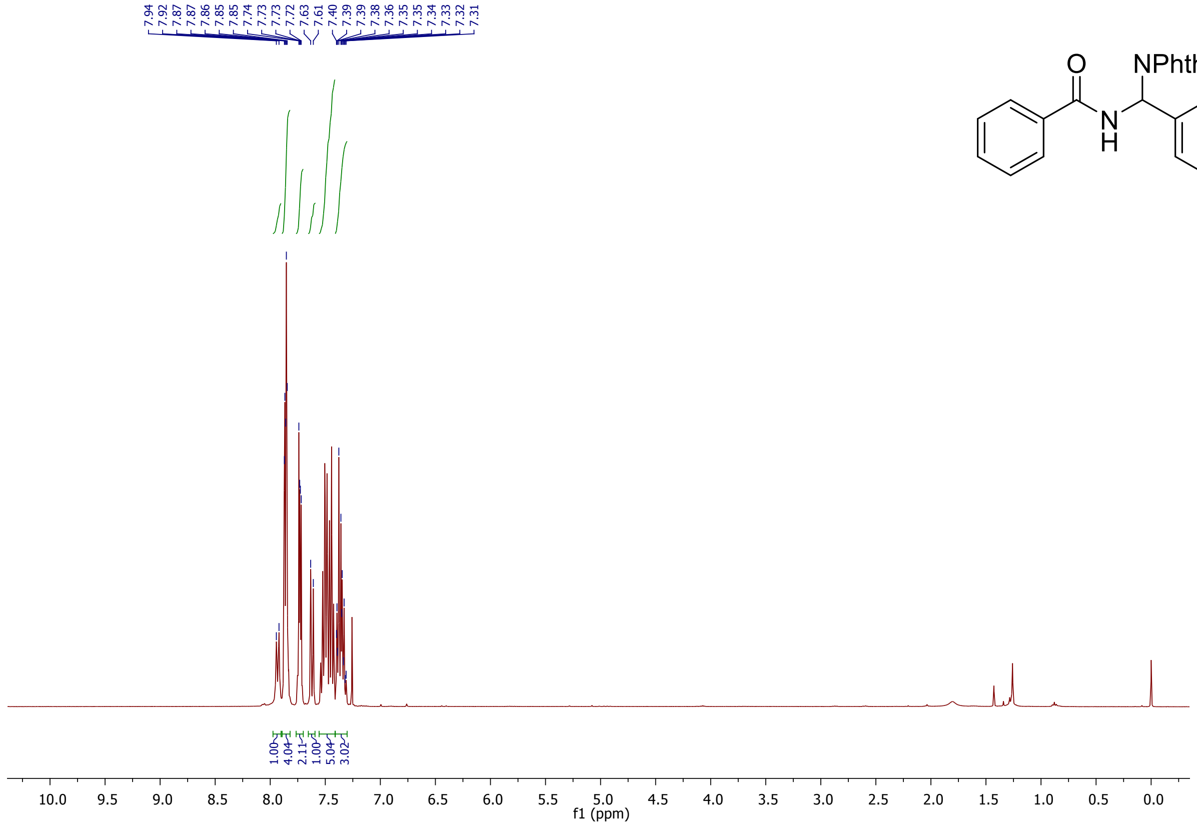

$^{13}C\{^{1}H\}$ NMR (101 MHz, $CDCl_3$)

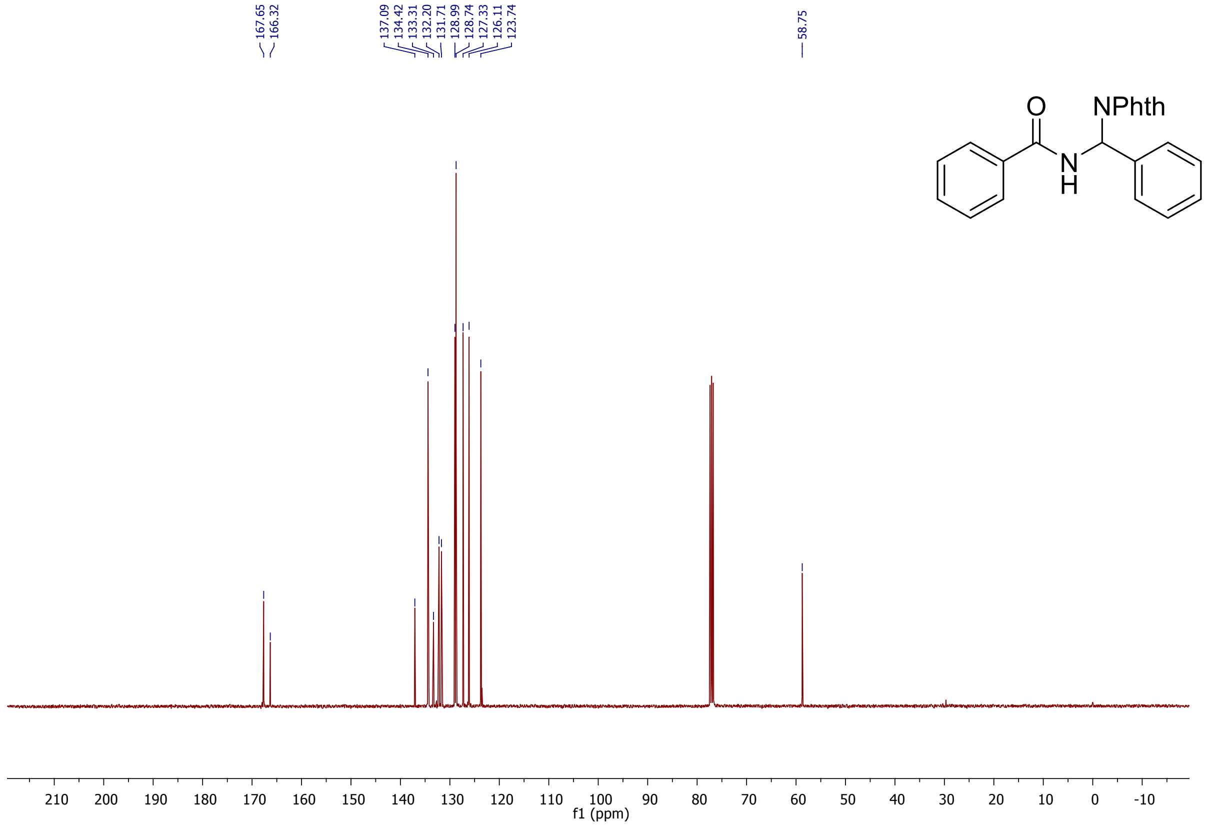

N-((3-chlorophenyl)(1,3-dioxoisoindolin-2-yl)methyl)-4-methoxybenzamide (3m)

^{1}H NMR (400 MHz, $CDCl_3$)

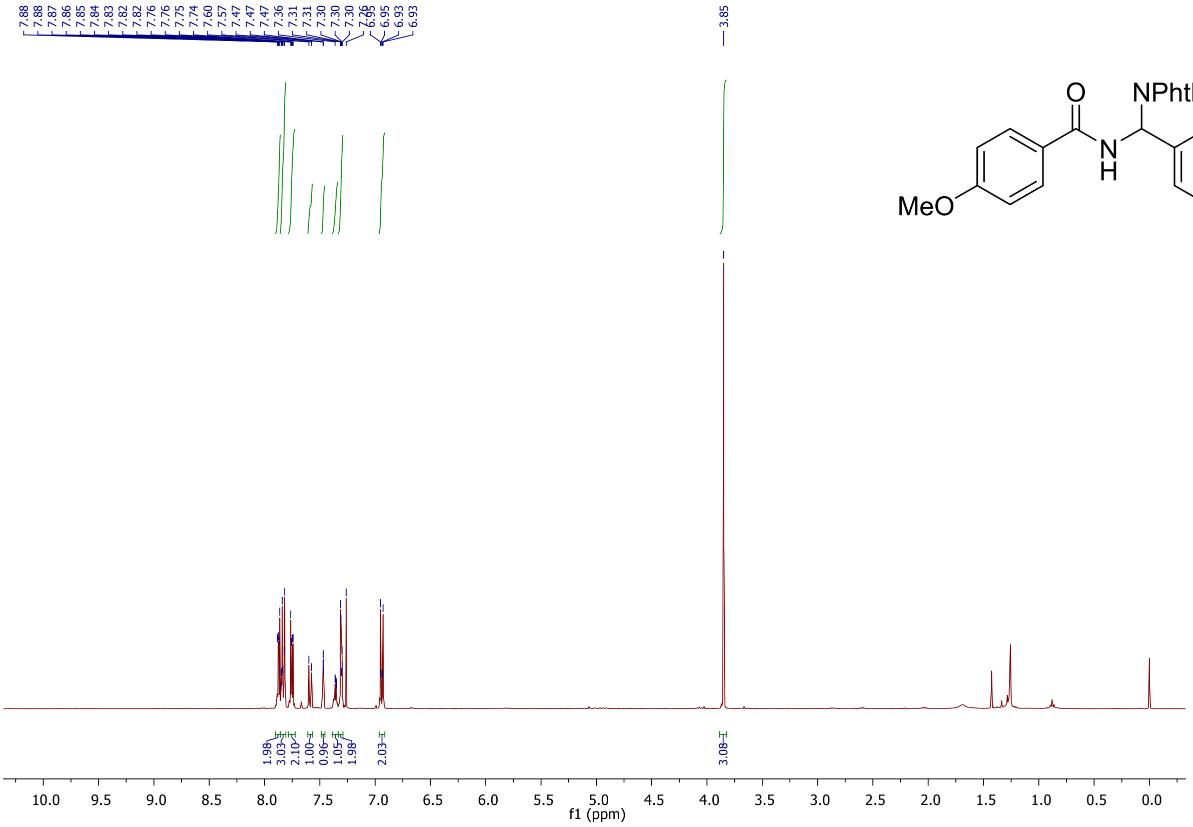

$^{13}C\{^{1}H\}$ NMR (101 MHz, $CDCl_3$)

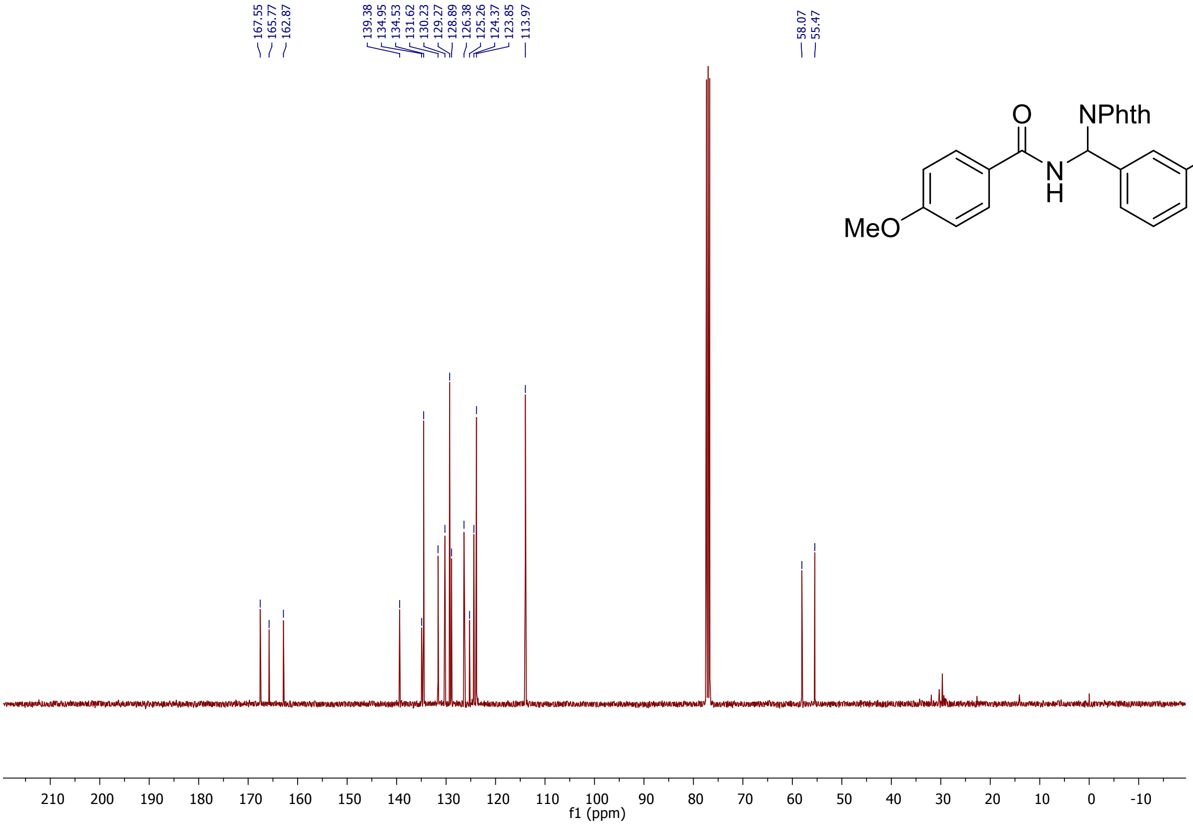

N-((1,3-dioxoisoindolin-2-yl)(naphthalen-2-yl)methyl)propionamide (3n)

^{1}H NMR (400 MHz, $CDCl_3$)

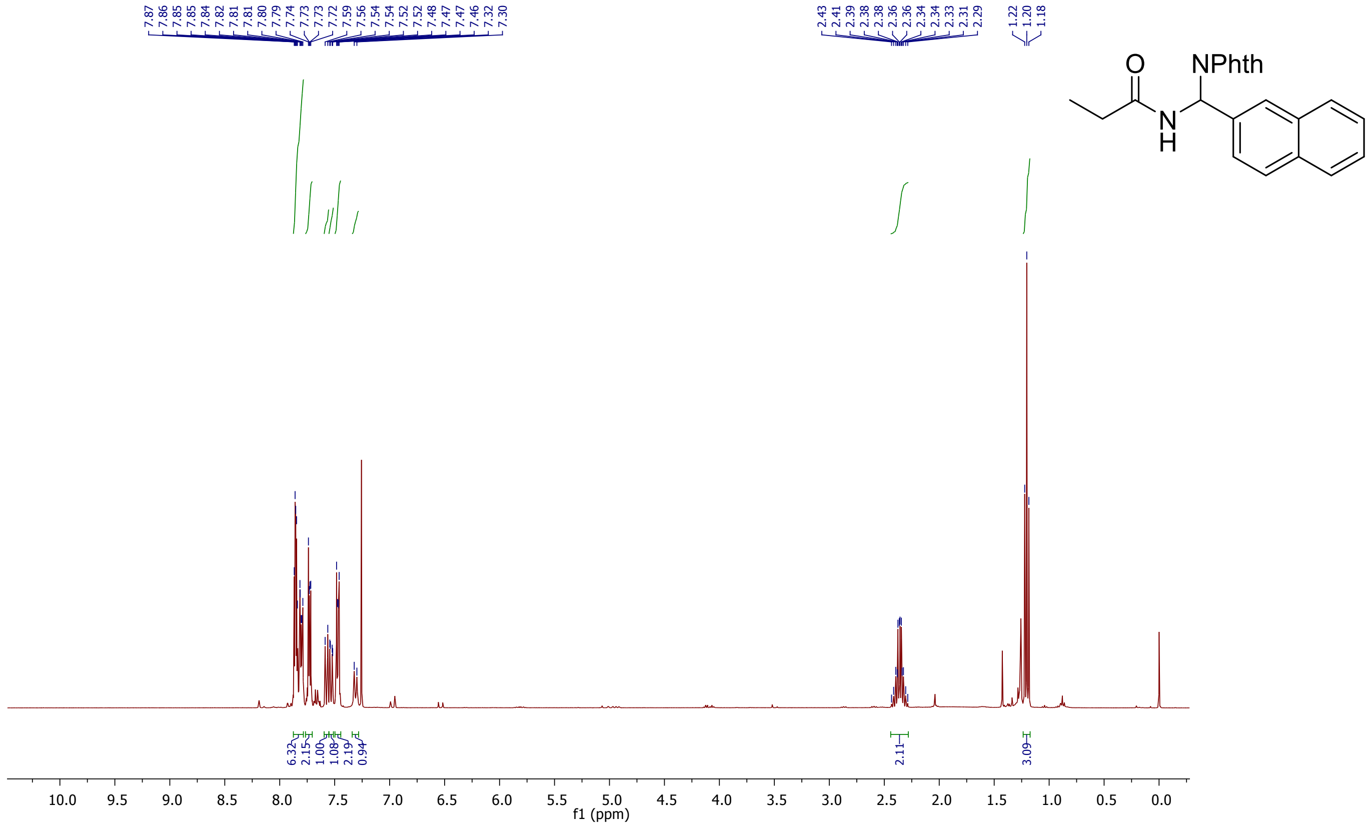

$^{13}C\{^{1}H\}$ NMR (101 MHz, $CDCl_3$)

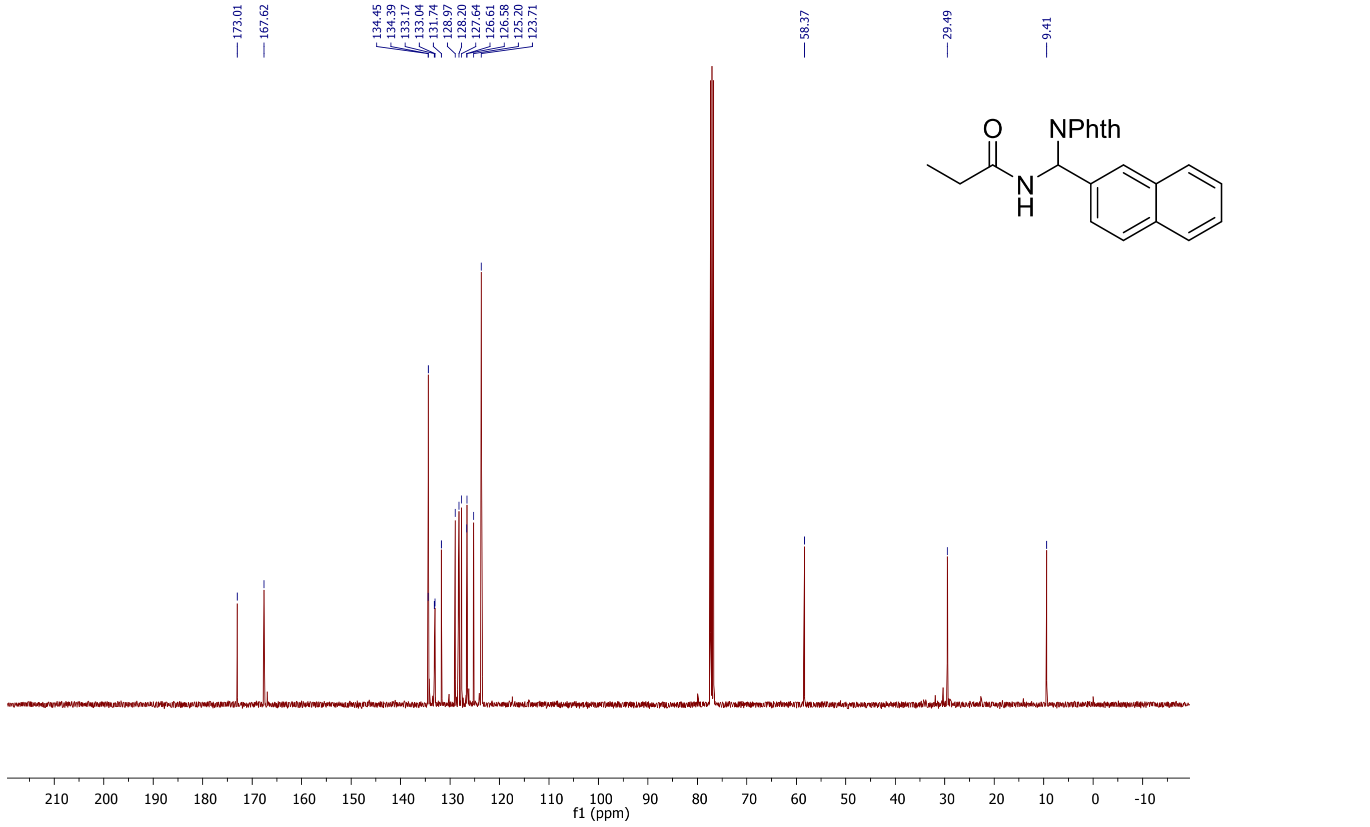

benzyl ((1,3-dioxoisoindolin-2-yl)(phenyl)methyl)carbamate (3o)

^{1}H NMR (400 MHz, $CDCl_3$)

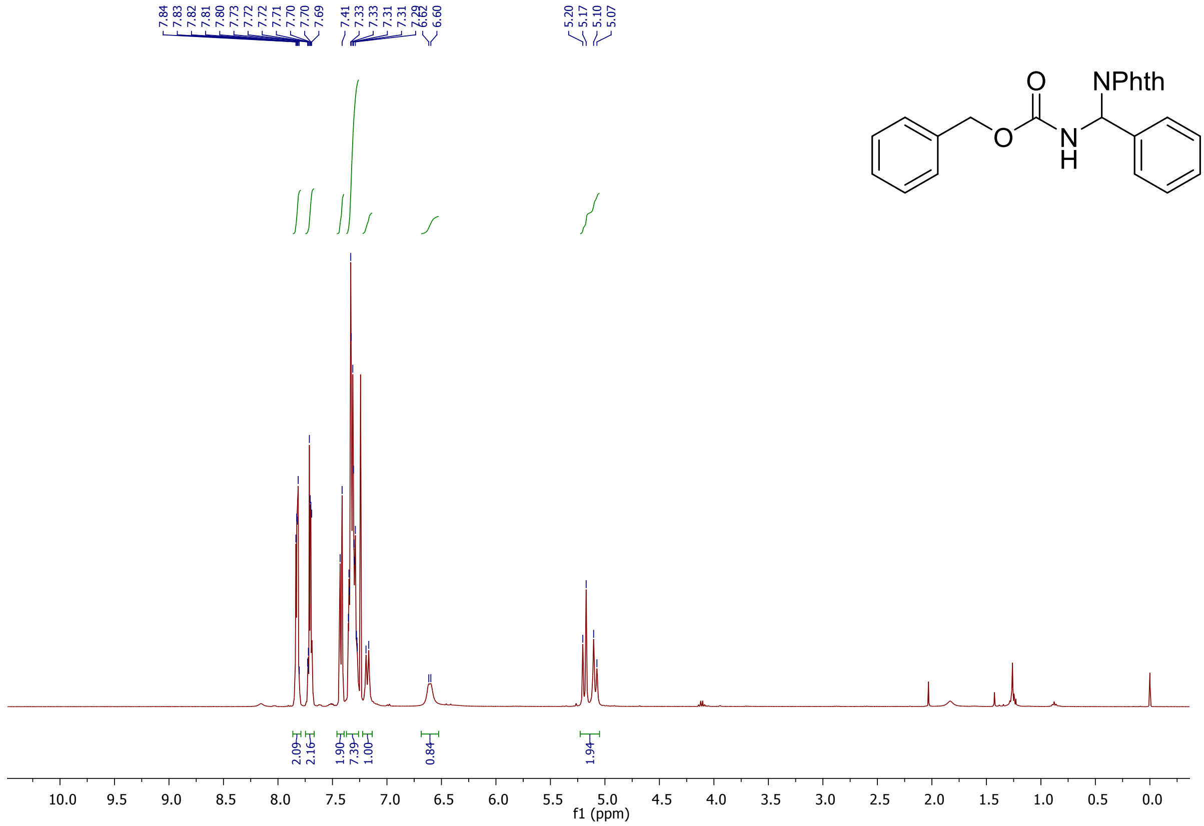

$^{13}C\{^{1}H\}$ NMR (101 MHz, $CDCl_3$)

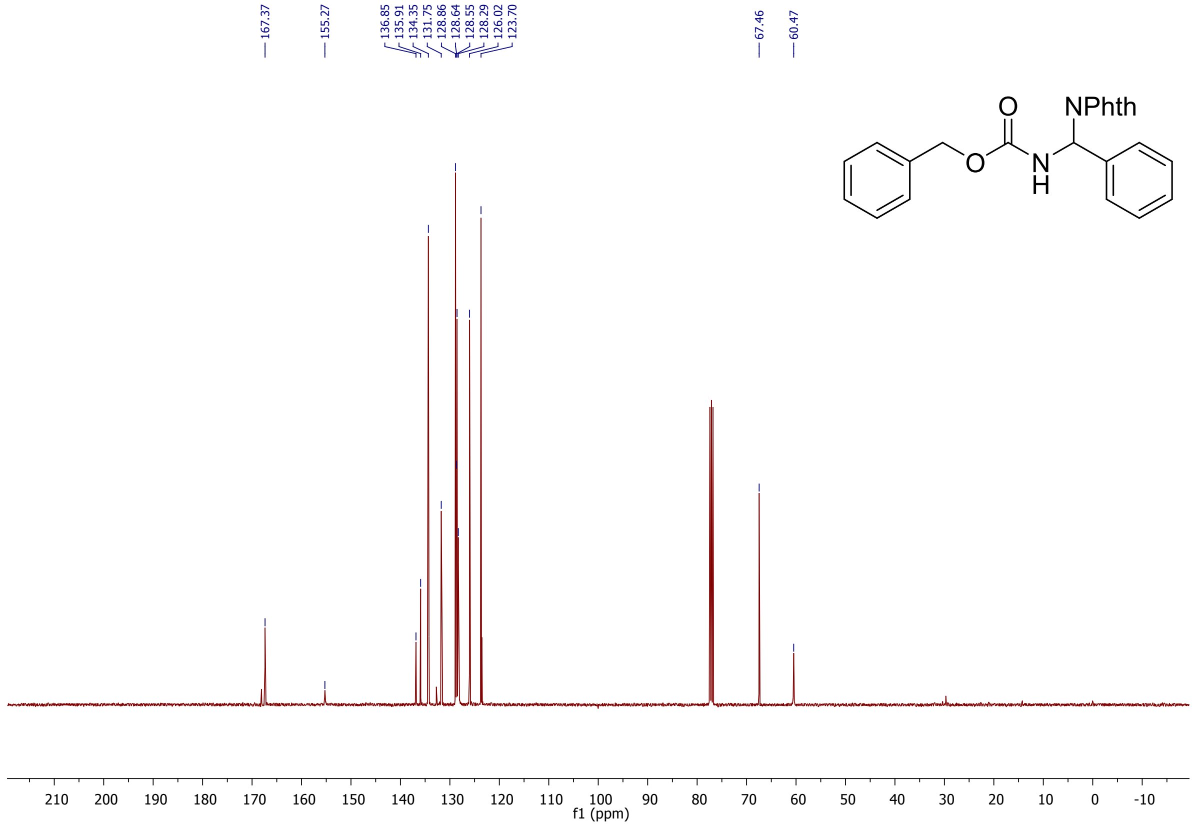

2-(1,3-dioxoisoindolin-2-yl)-2-phenylacetonitrile (4a)

^{1}H NMR (400 MHz, $CDCl_3$)

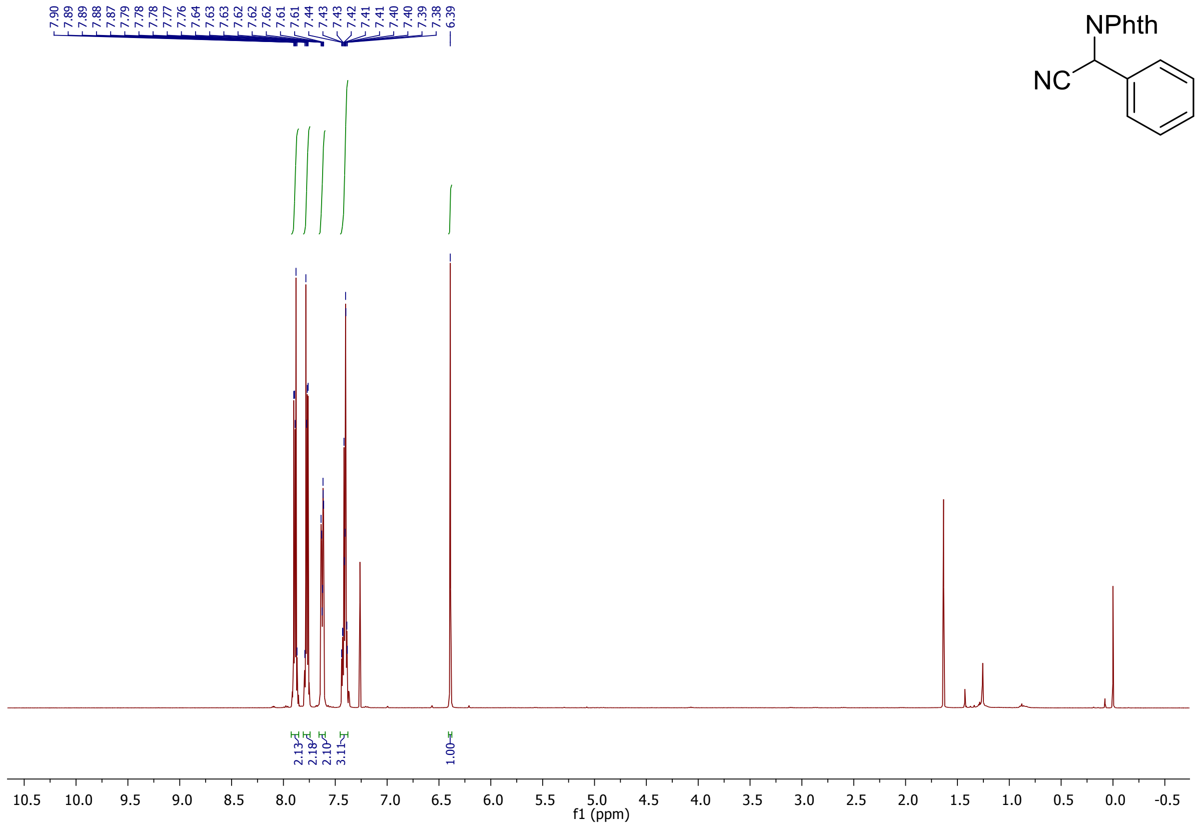

$^{13}C\{^{1}H\}$ NMR (101 MHz, $CDCl_3$)

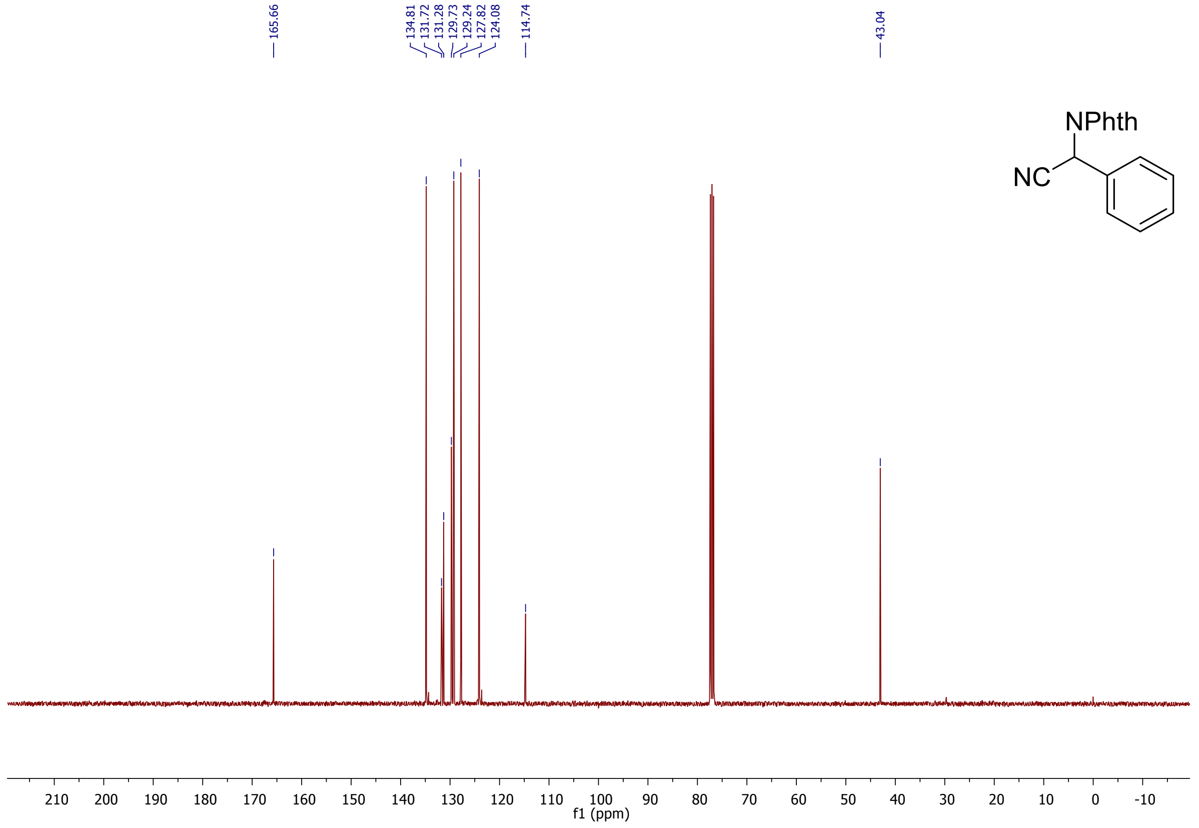

2-(1,3-dioxoisoindolin-2-yl)-2-(4-fluorophenyl)acetonitrile (4b)

^{1}H NMR (400 MHz, $CDCl_3$)

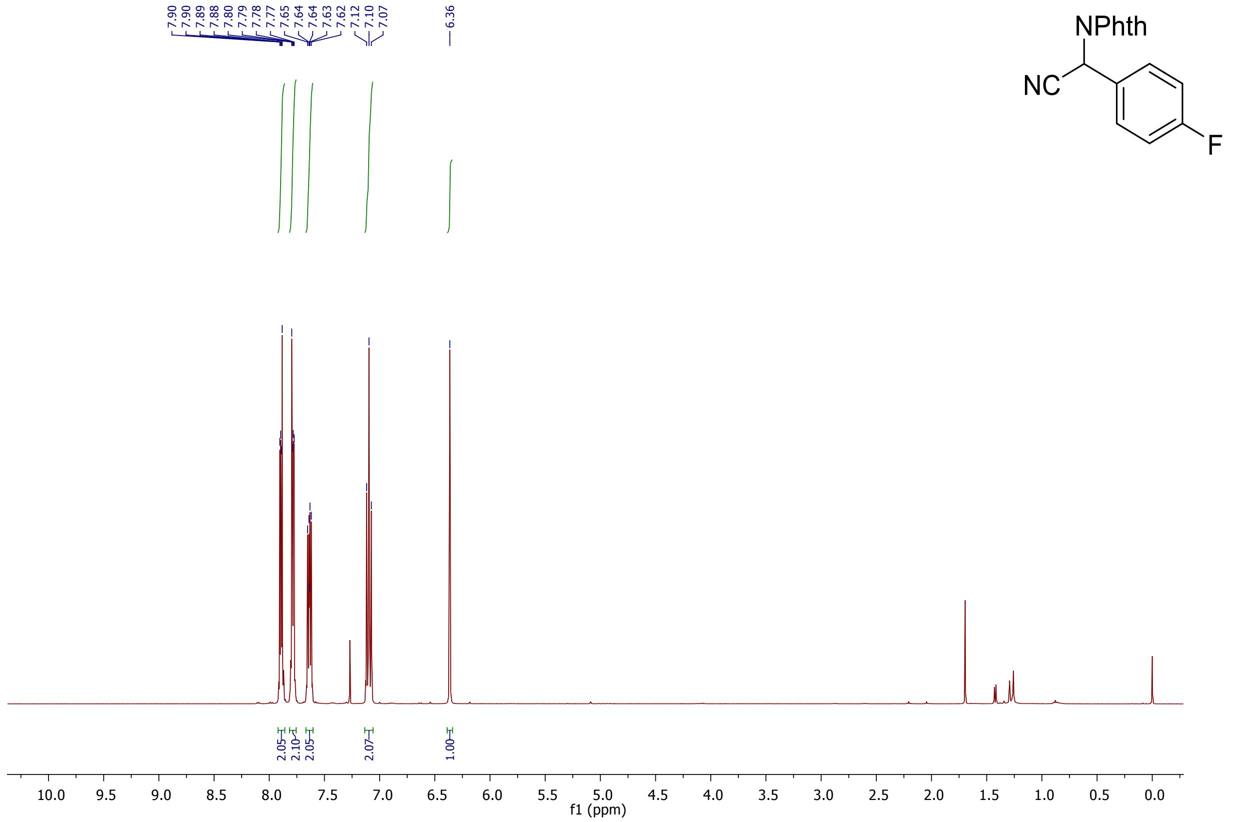

$^{13}C\{^{1}H\}$ NMR (101 MHz, $CDCl_3$)

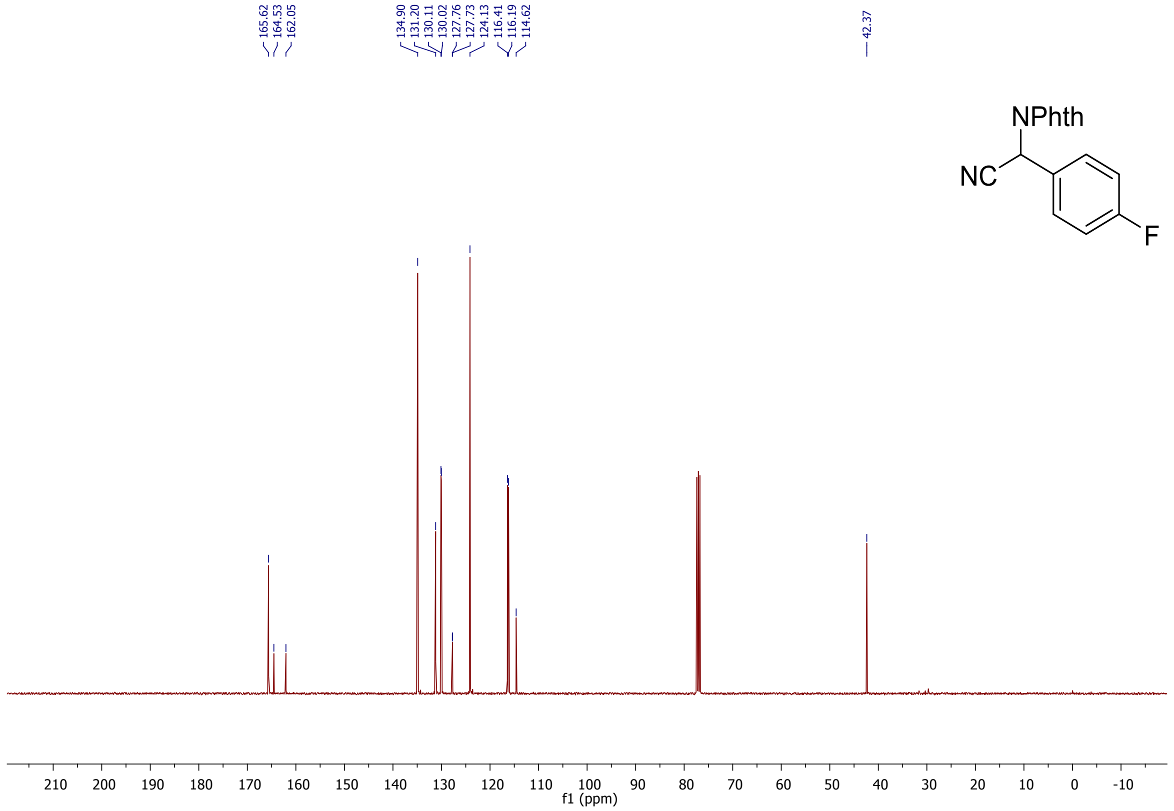

2-(3-chlorophenyl)-2-(1,3-dioxoisoindolin-2-yl)acetonitrile (4c)

^{1}H NMR (400 MHz, $CDCl_3$)

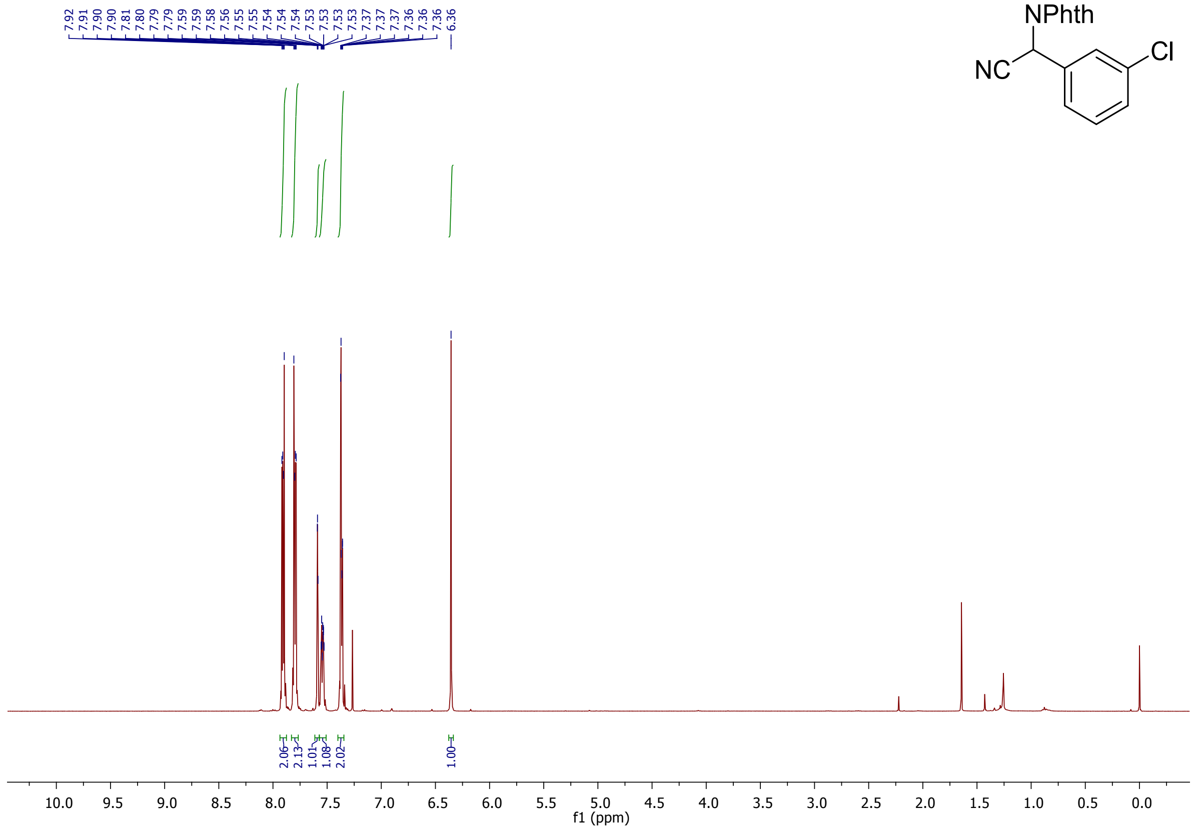

$^{13}C\{^{1}H\}$ NMR (101 MHz, $CDCl_3$)

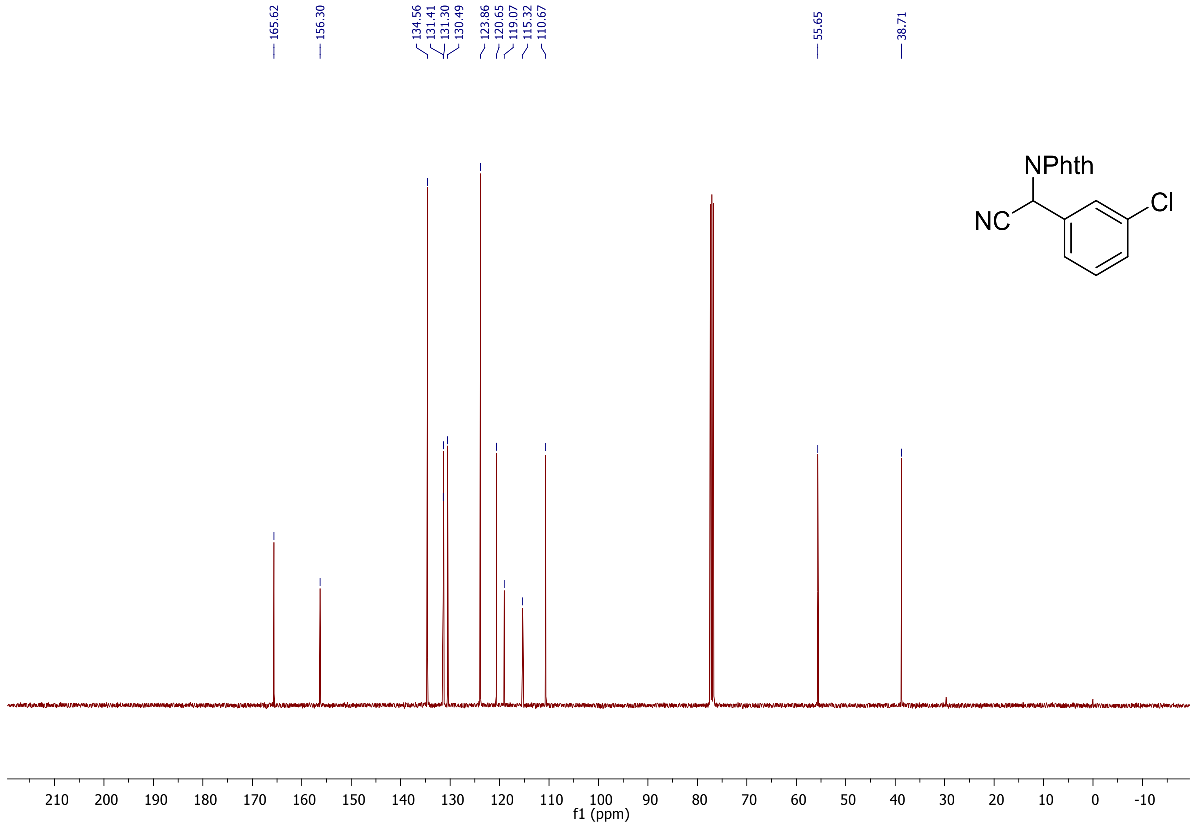

2-(2-bromophenyl)-2-(1,3-dioxoisoindolin-2-yl)acetonitrile (4d)

^{1}H NMR (400 MHz, $CDCl_3$)

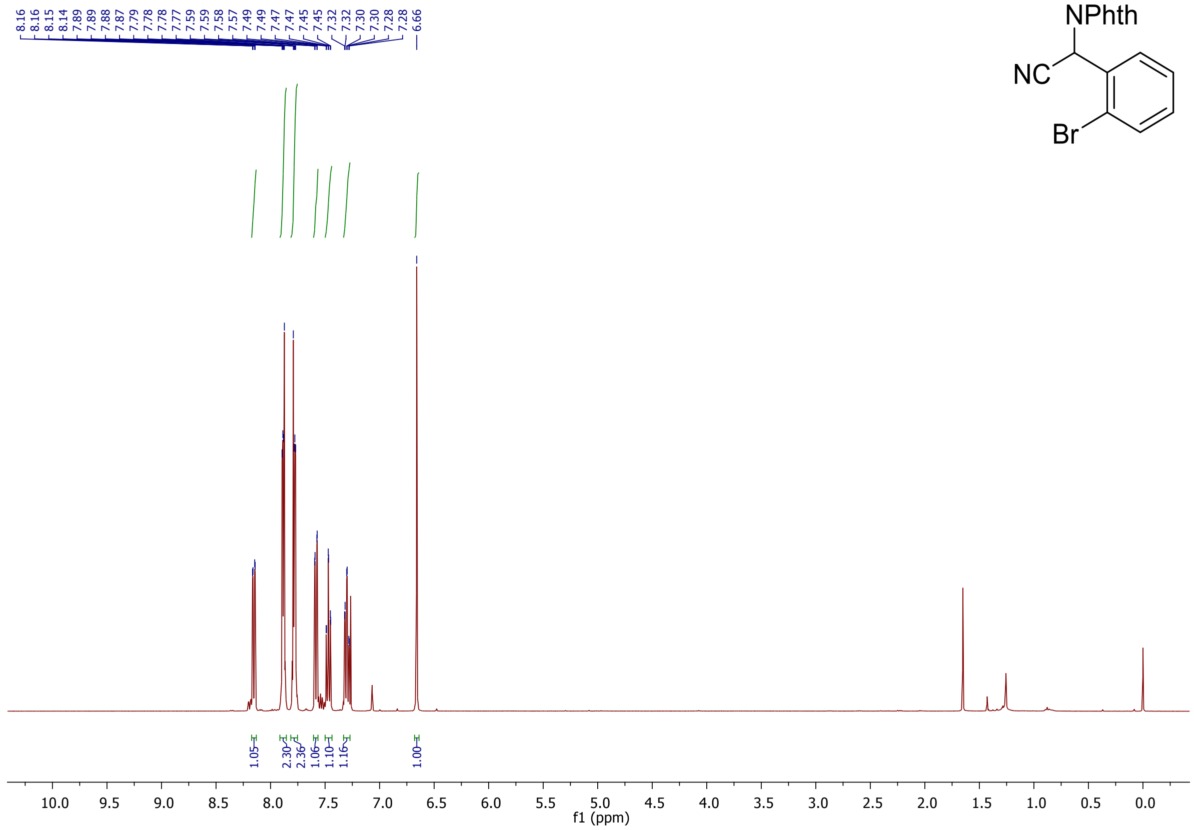

^{13}C{^{1}H} NMR (101 MHz, $CDCl_3$)

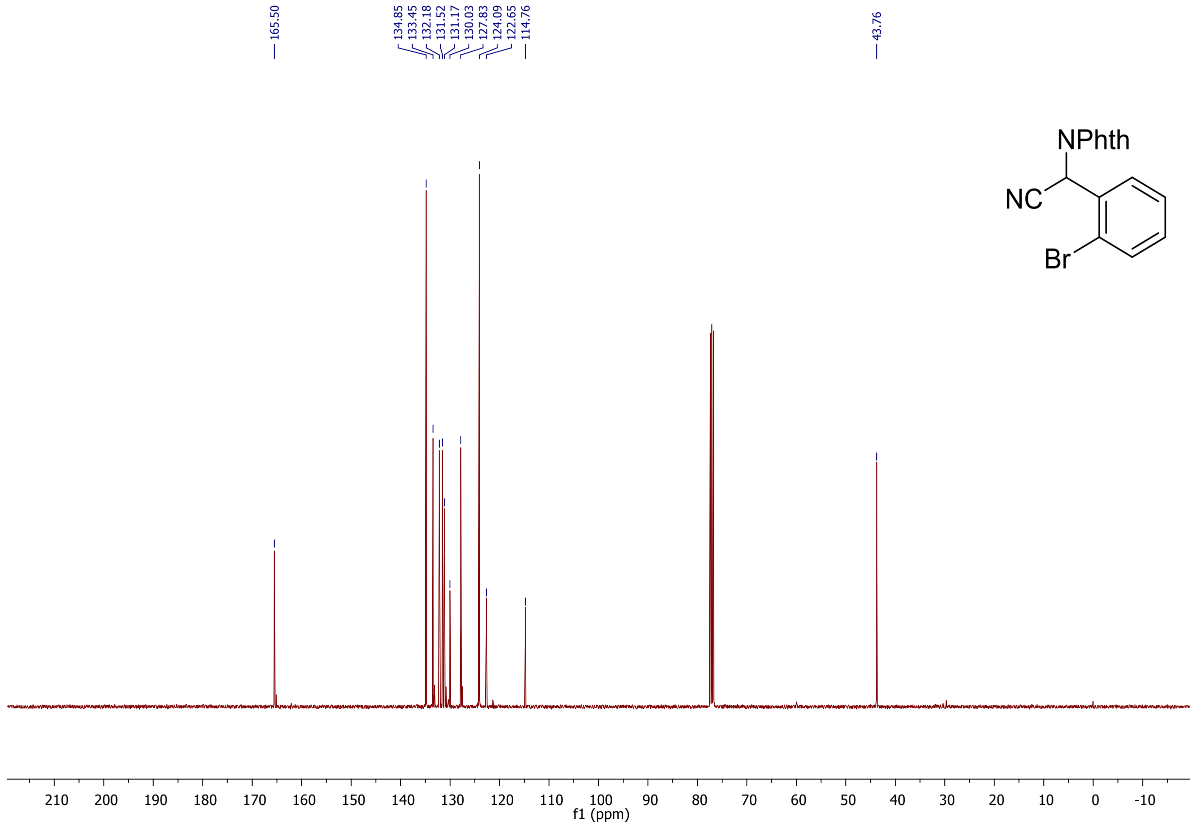

2-(3-bromo-2-fluorophenyl)-2-(1,3-dioxoisoindolin-2-yl)acetonitrile (4e)

^{1}H NMR (400 MHz, CDCl$_3$)

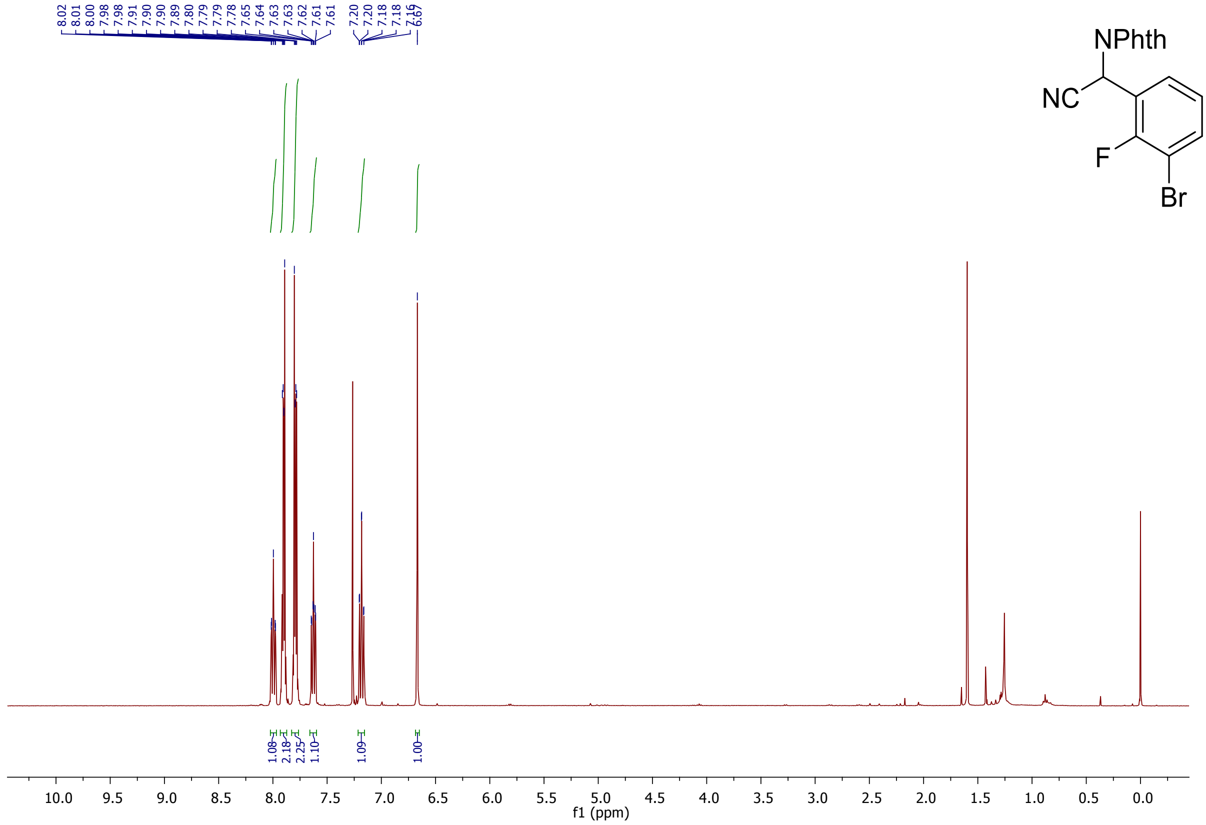

^{13}C{^{1}H} NMR (101 MHz, CDCl$_3$)

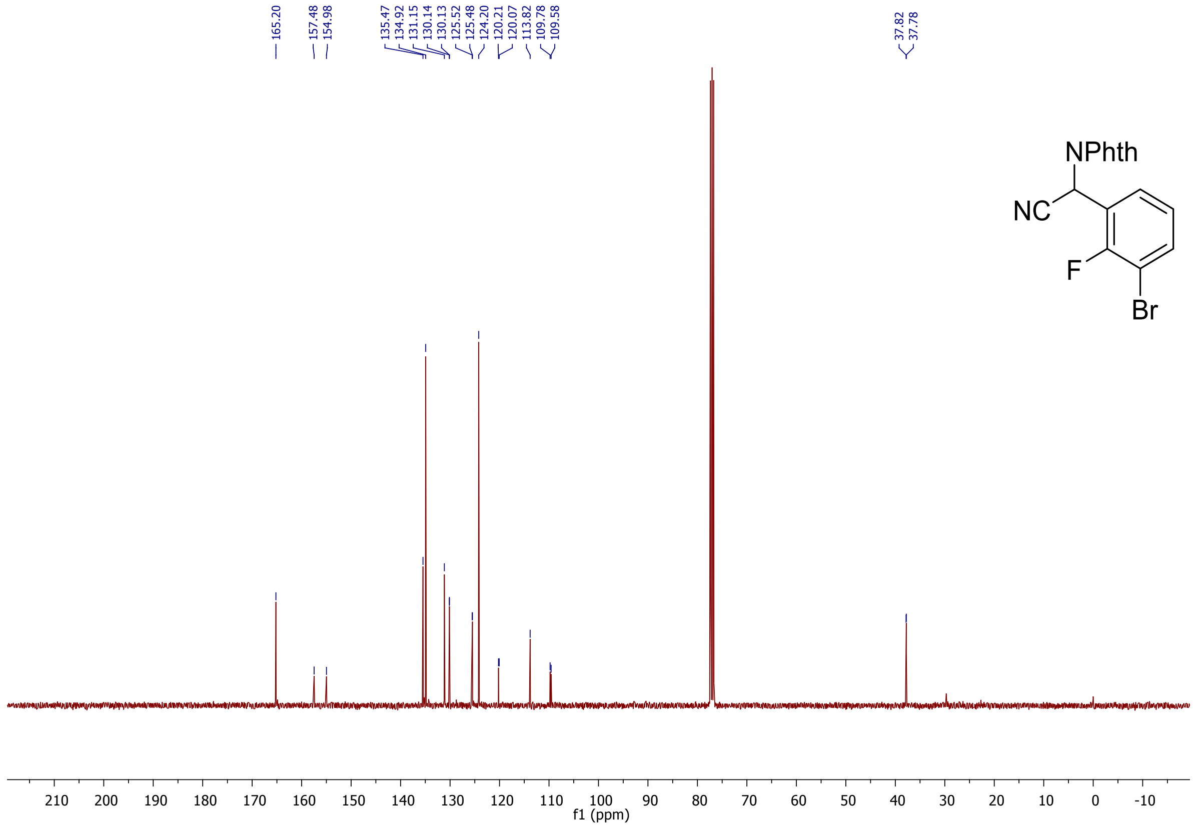

2-(1,3-dioxoisoindolin-2-yl)-2-(p-tolyl)acetonitrile (4f)

^{1}H NMR (400 MHz, $CDCl_3$)

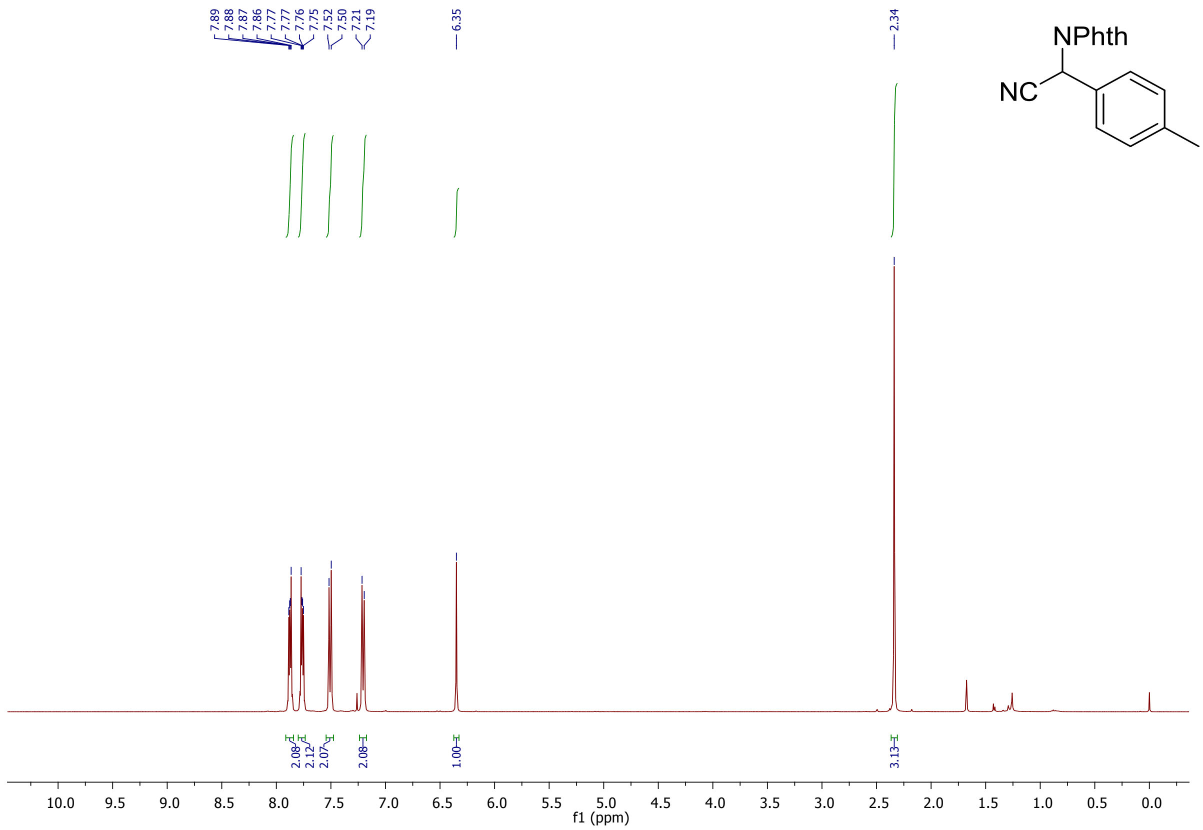

$^{13}C\{^{1}H\}$ NMR (101 MHz, $CDCl_3$)

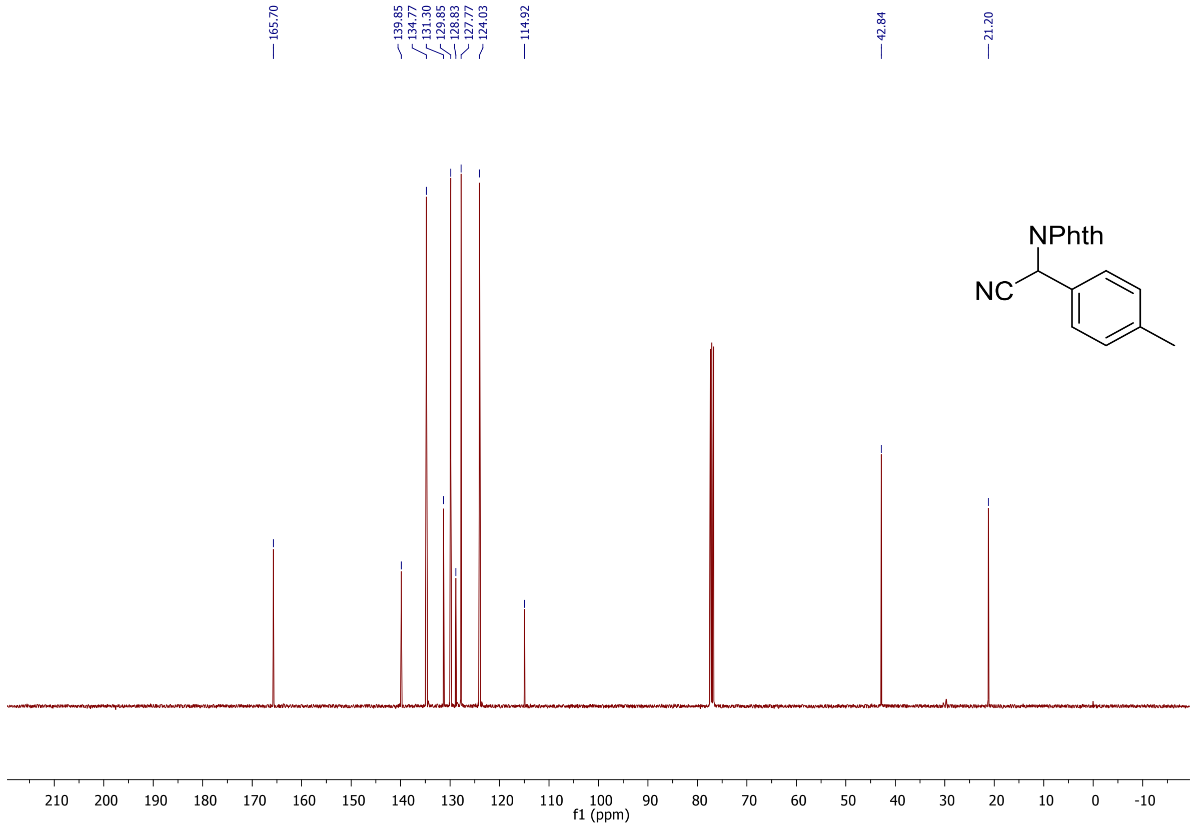

2-(1,3-dioxoisoindolin-2-yl)-2-(2-methoxyphenyl)acetonitrile (4g)

^{1}H NMR (400 MHz, $CDCl_3$)

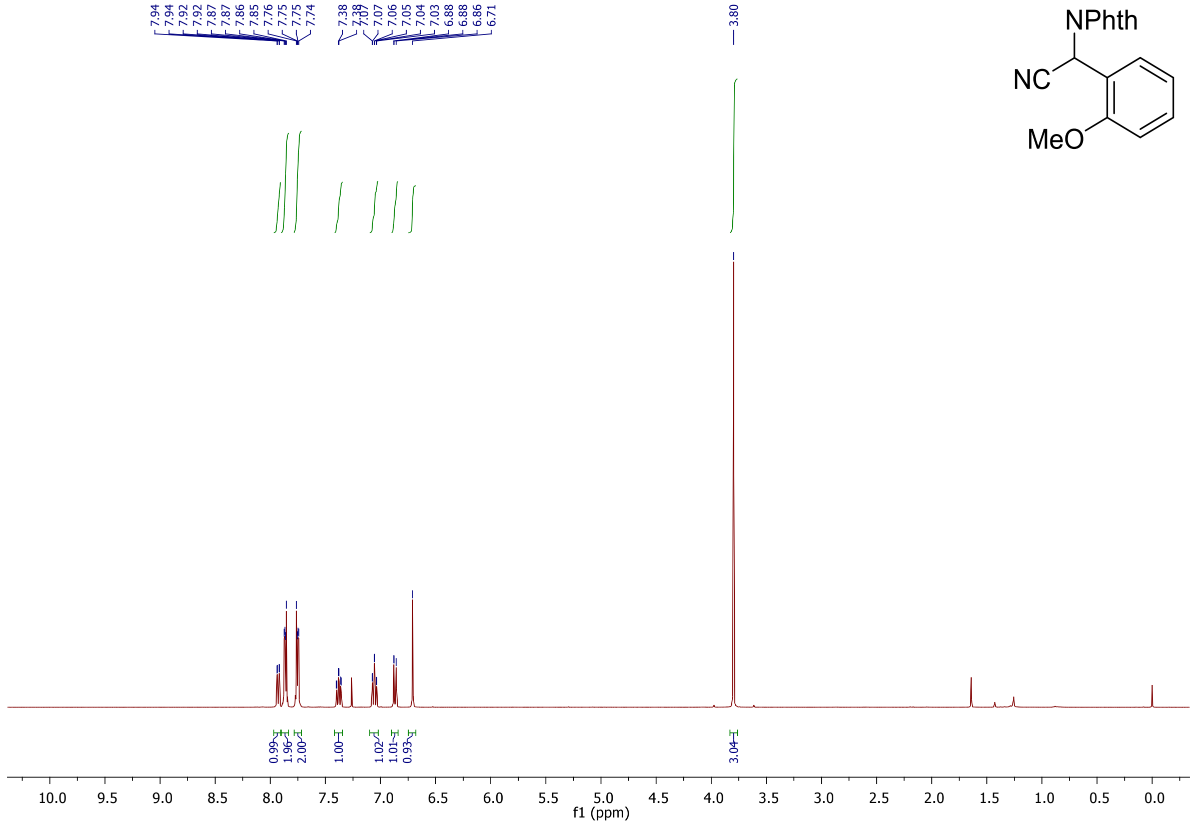

$^{13}C\{^{1}H\}$ NMR (101 MHz, $CDCl_3$)

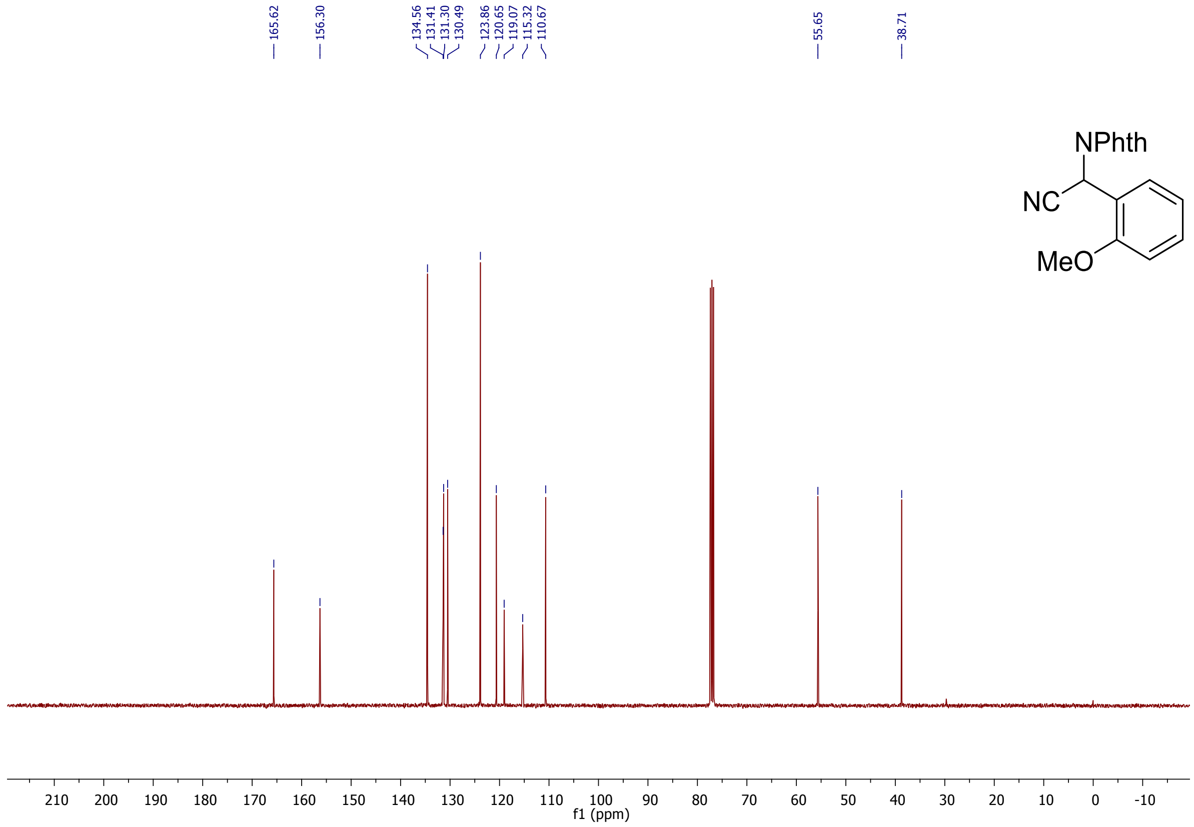

2-(1,3-dioxoisoindolin-2-yl)-2-(naphthalen-2-yl)acetonitrile (4h)

^{1}H NMR (400 MHz, $CDCl_3$)

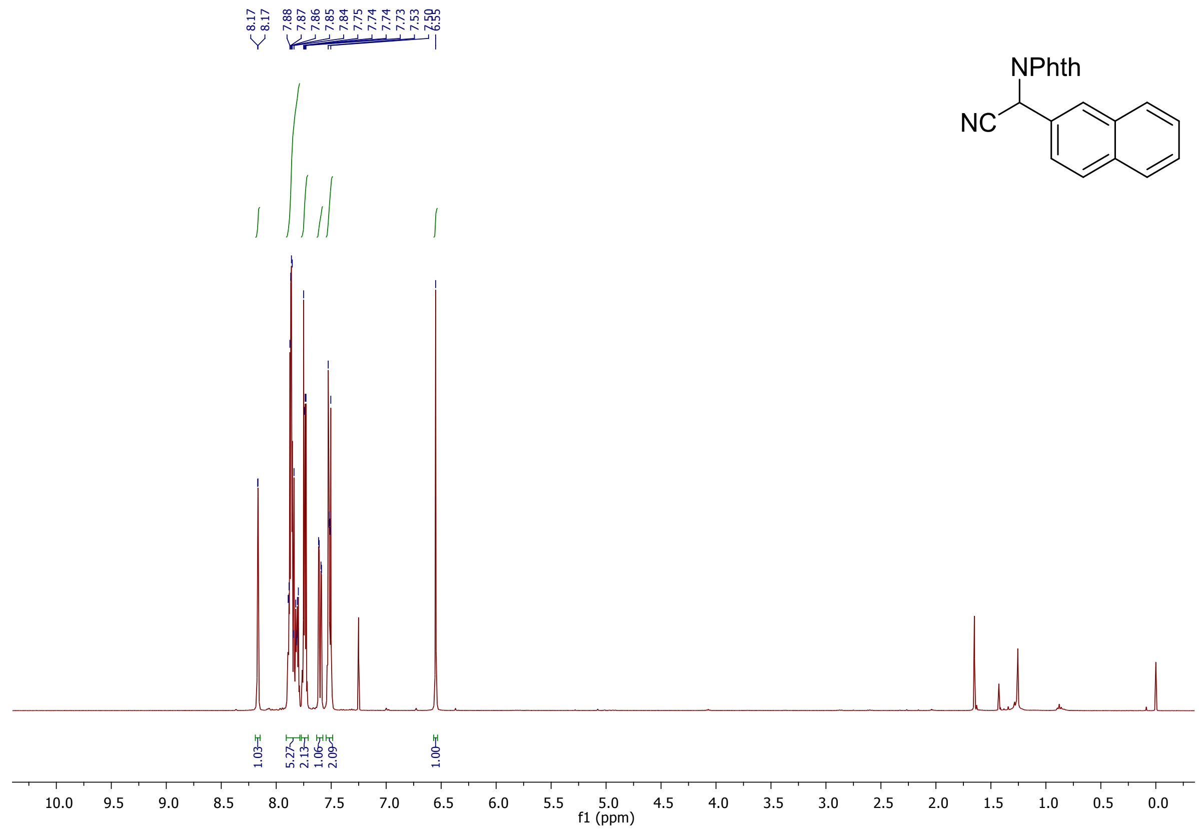

^{13}C{^{1}H} NMR (101 MHz, $CDCl_3$)

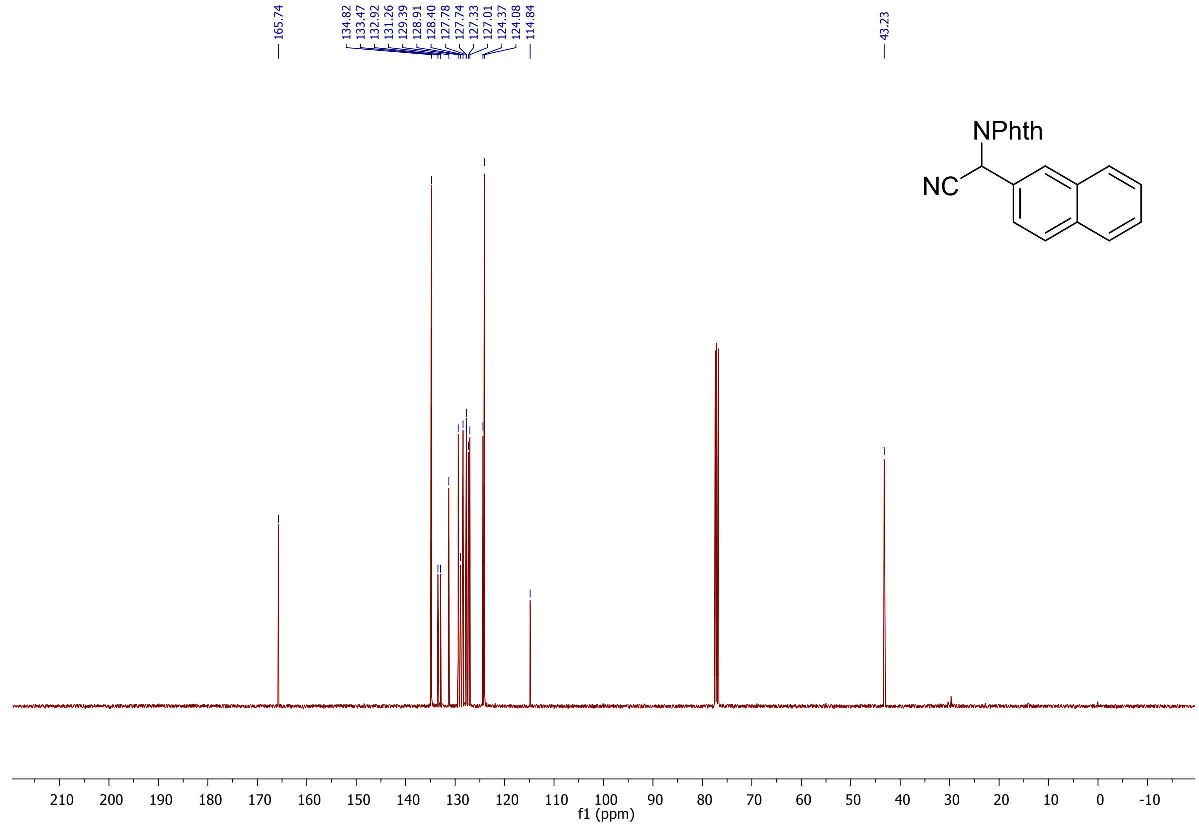

2-(1,3-dioxoisoindolin-2-yl)-2-(furan-2-yl)acetonitrile (4i)

^{1}H NMR (400 MHz, $CDCl_3$)

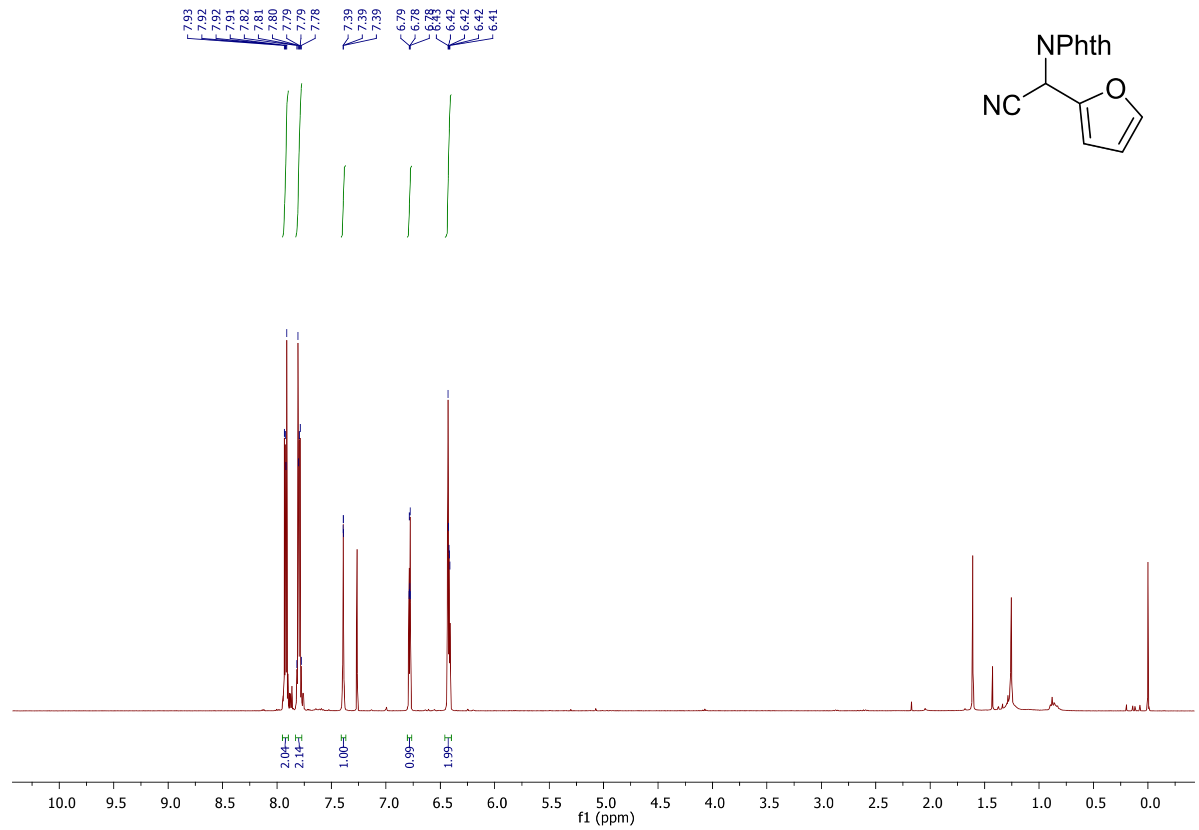

^{13}C{^{1}H} NMR (101 MHz, $CDCl_3$)

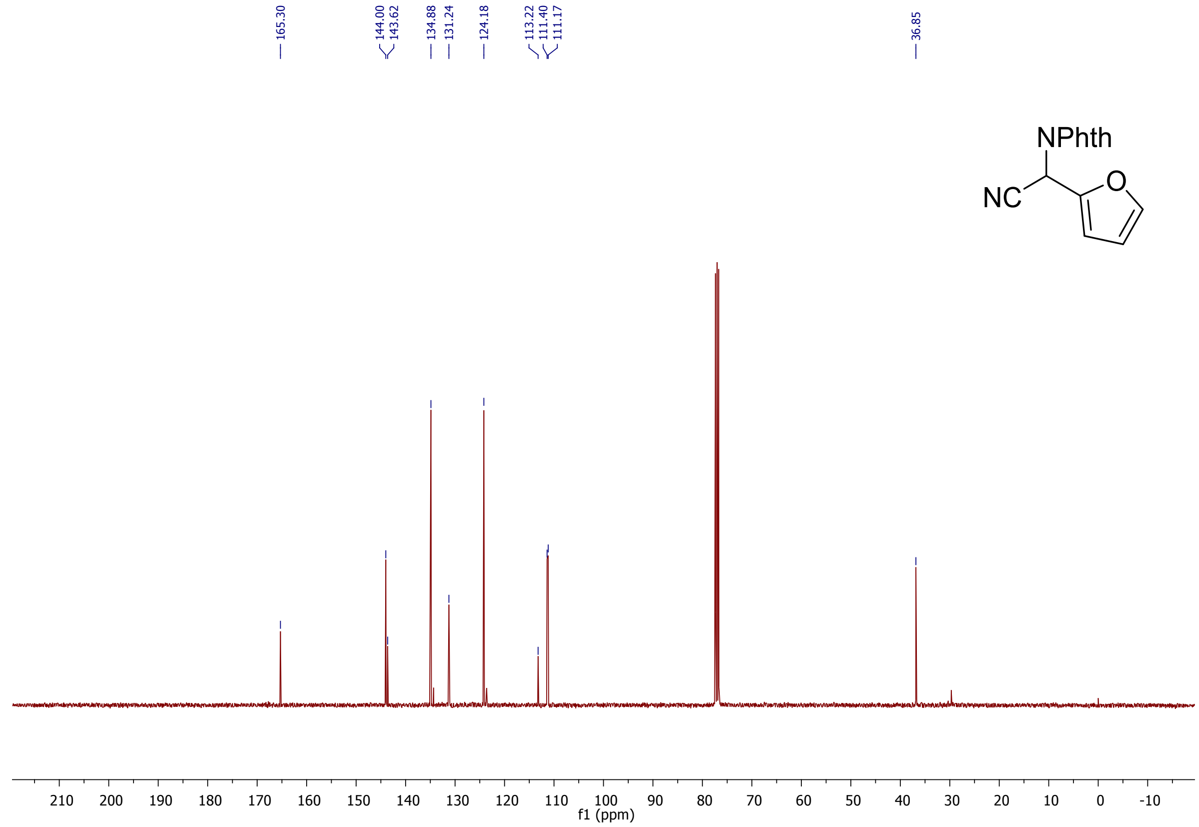

2-(1,3-dioxoisoindolin-2-yl)-2-(thiophen-2-yl)acetonitrile (4j)

^1H NMR (400 MHz, $CDCl_3$)

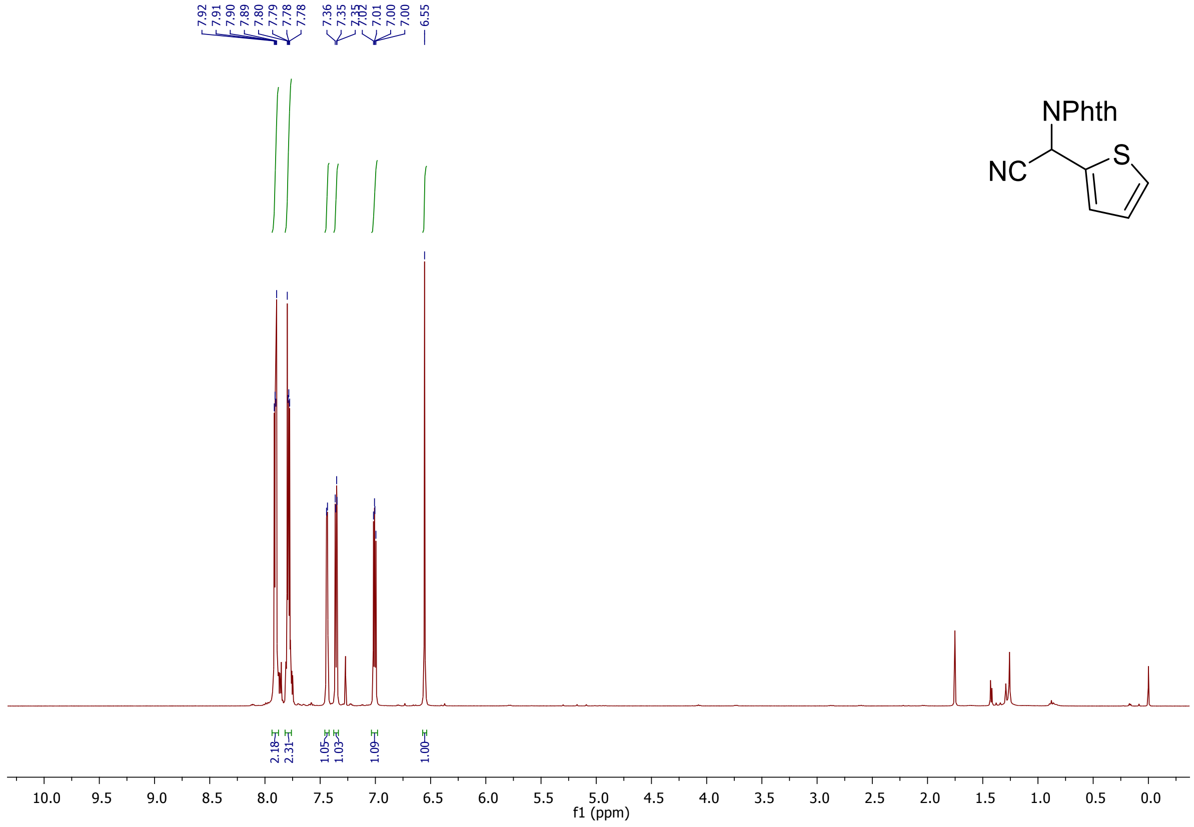

$^{13}C\{^1H\}$ NMR (101 MHz, $CDCl_3$)

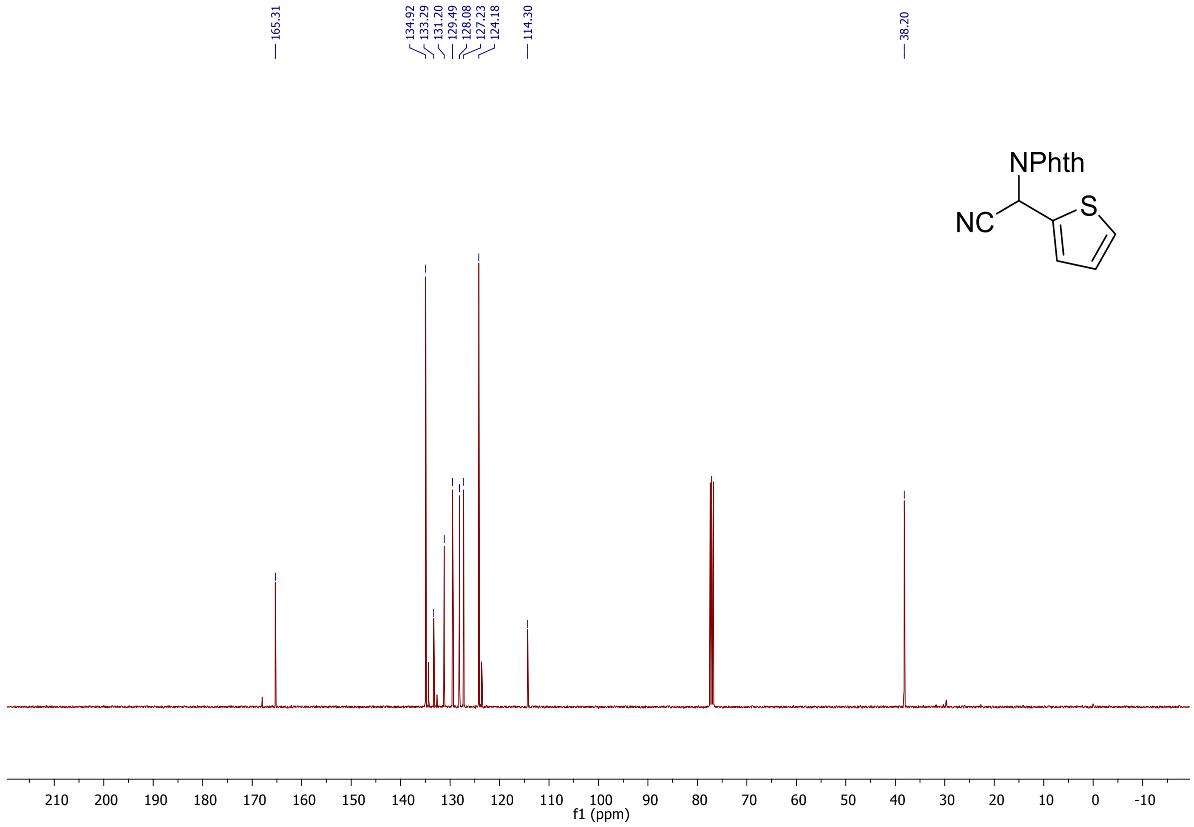

2-cyclohexyl-2-(1,3-dioxoisoindolin-2-yl)acetonitrile (4k)

^{1}H NMR (400 MHz, $CDCl_3$)

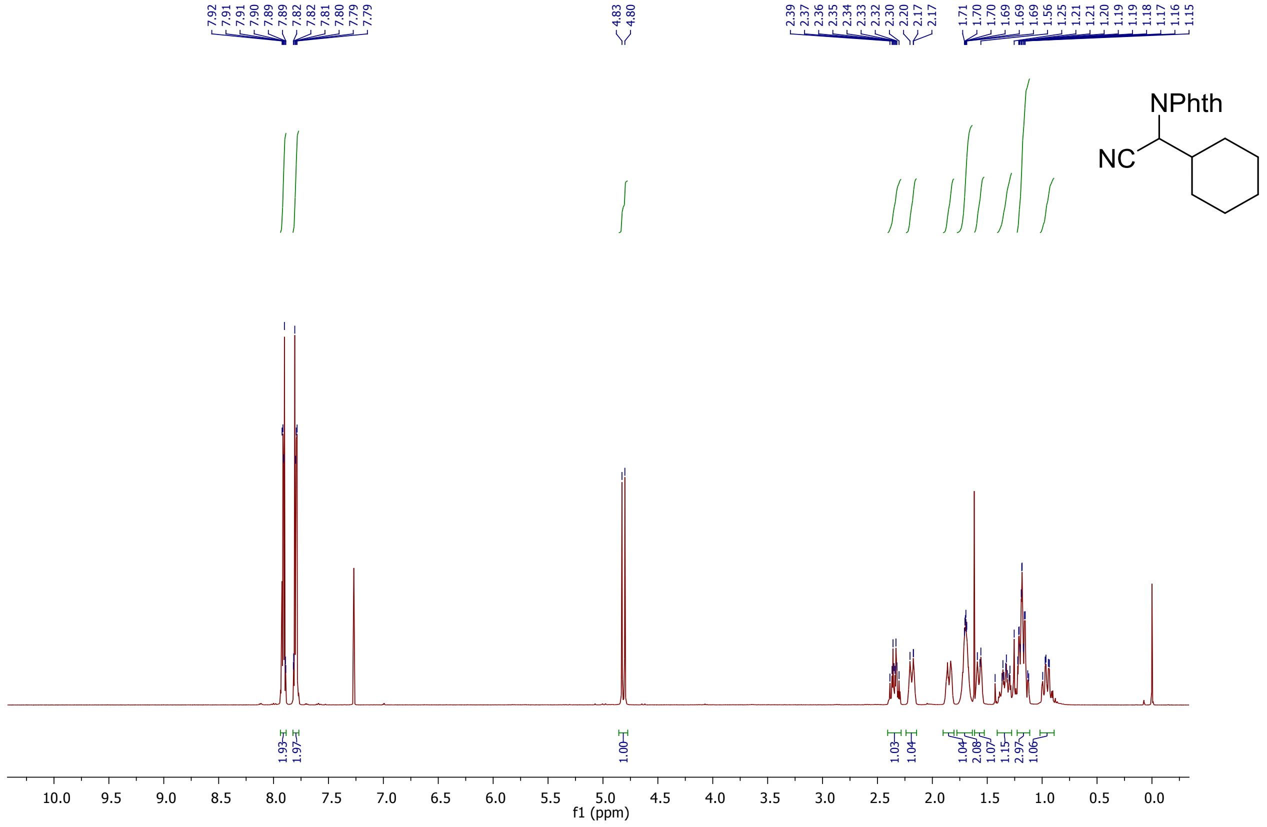

$^{13}C\{^{1}H\}$ NMR (101 MHz, $CDCl_3$)

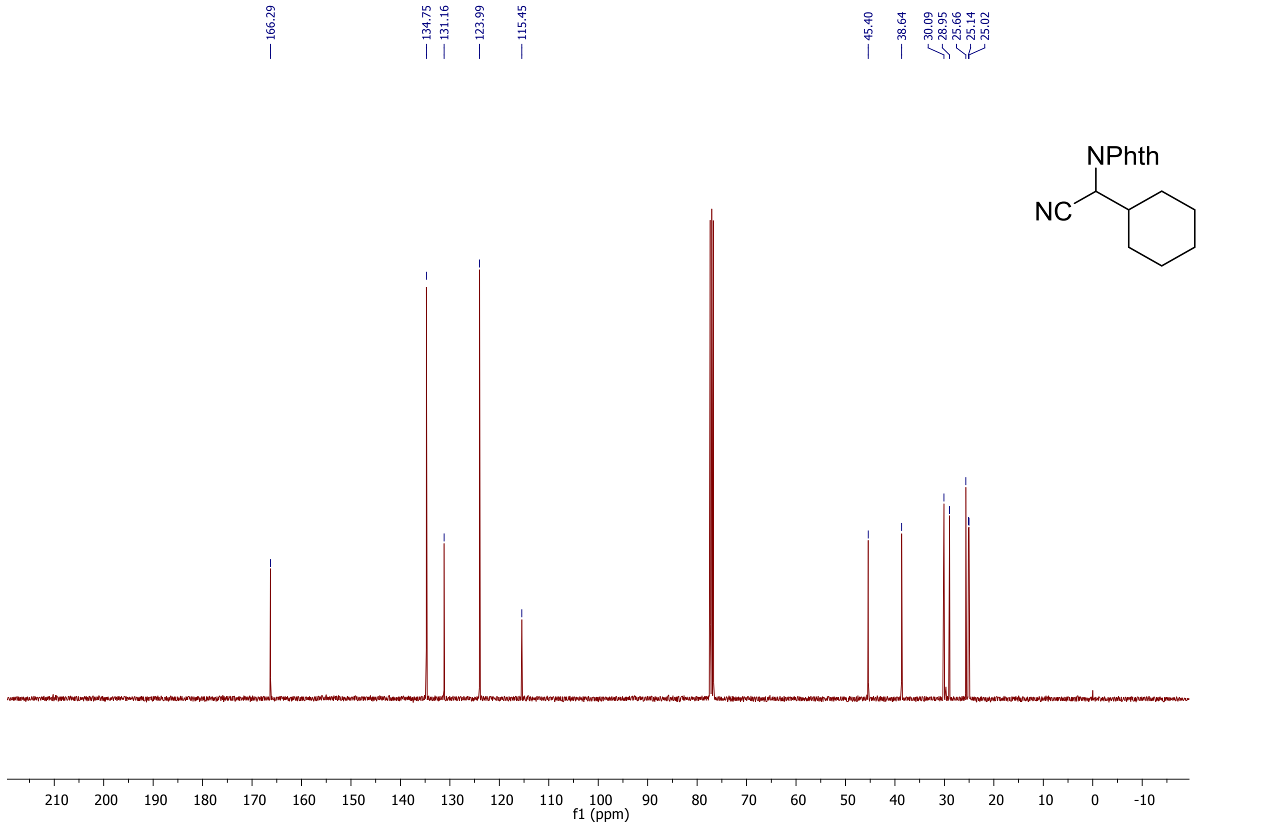

2-cyclopropyl-2-(1,3-dioxoisoindolin-2-yl)acetonitrile (4l)

^{1}H NMR (400 MHz, $CDCl_3$)

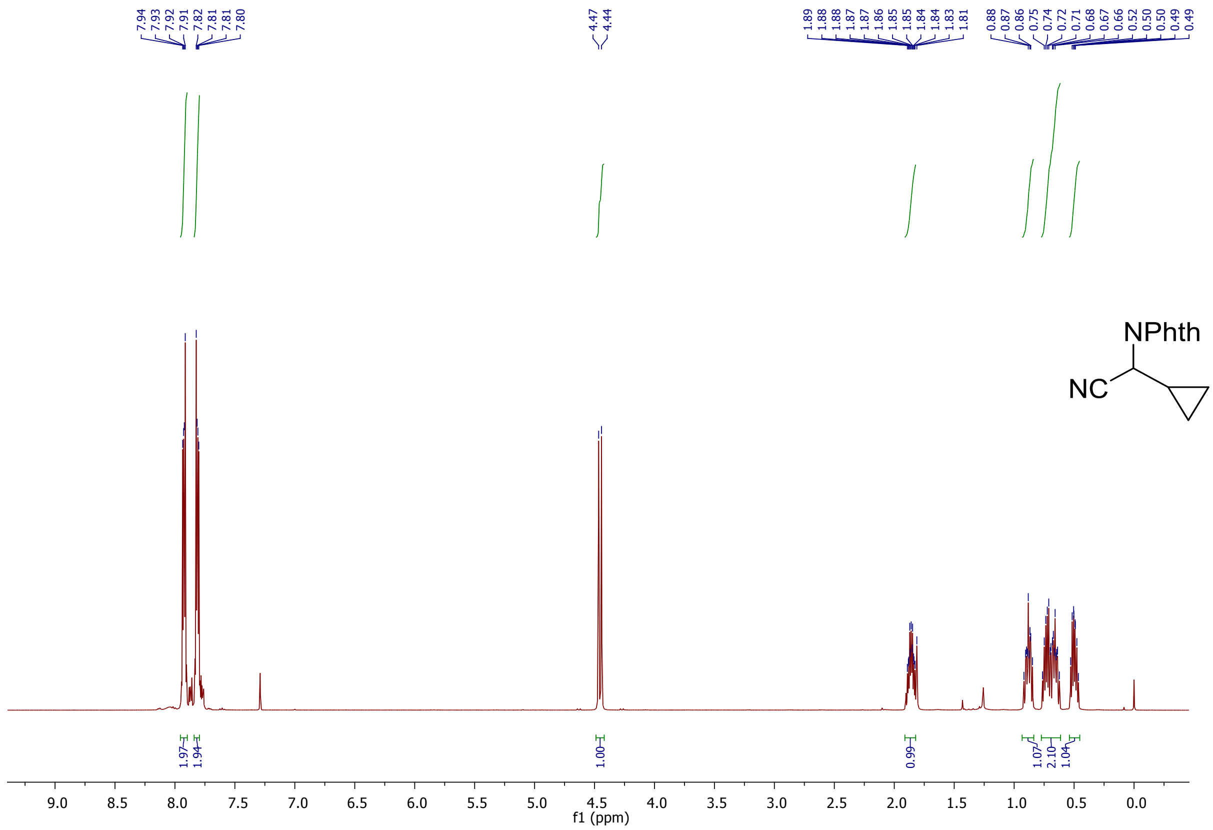

$^{13}C\{^{1}H\}$ NMR (101 MHz, $CDCl_3$)

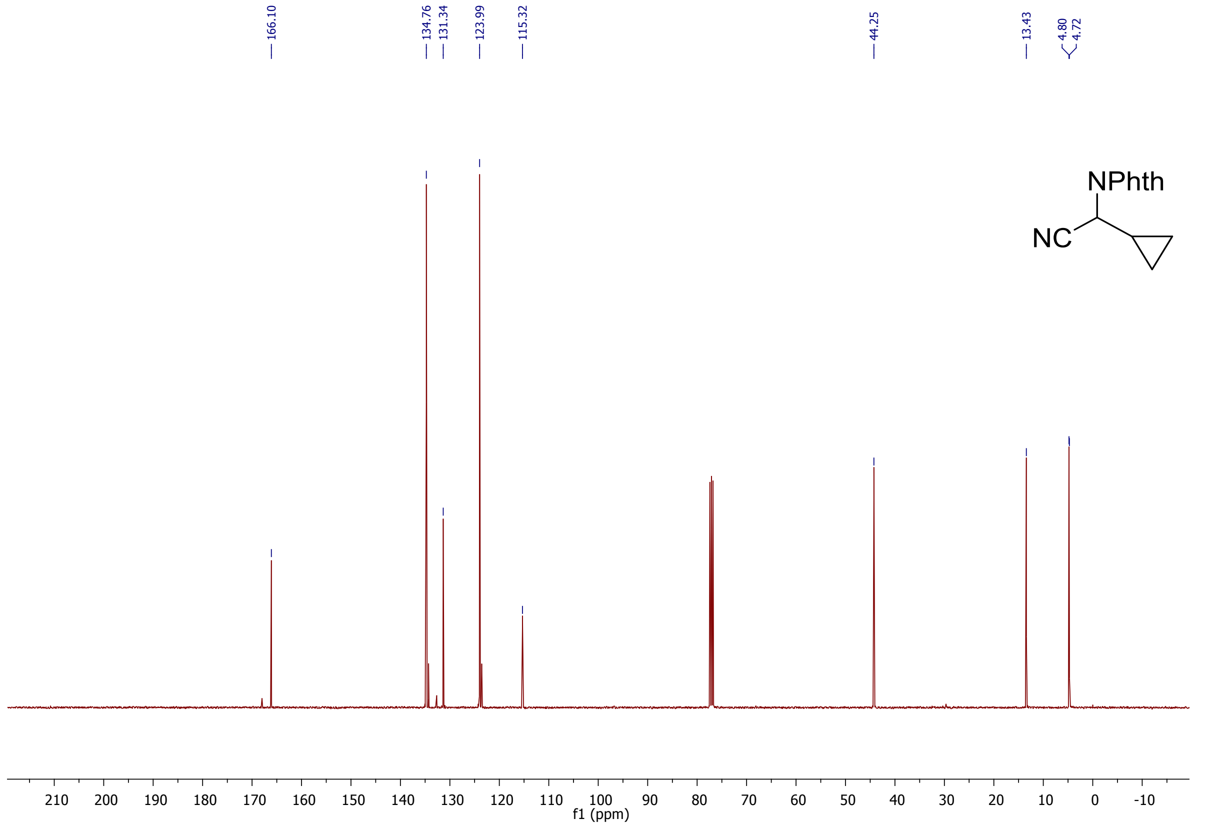

tert-butyl (1-phenylbut-3-en-1-yl)carbamate (5a)

^{1}H NMR (400 MHz, $CDCl_3$)

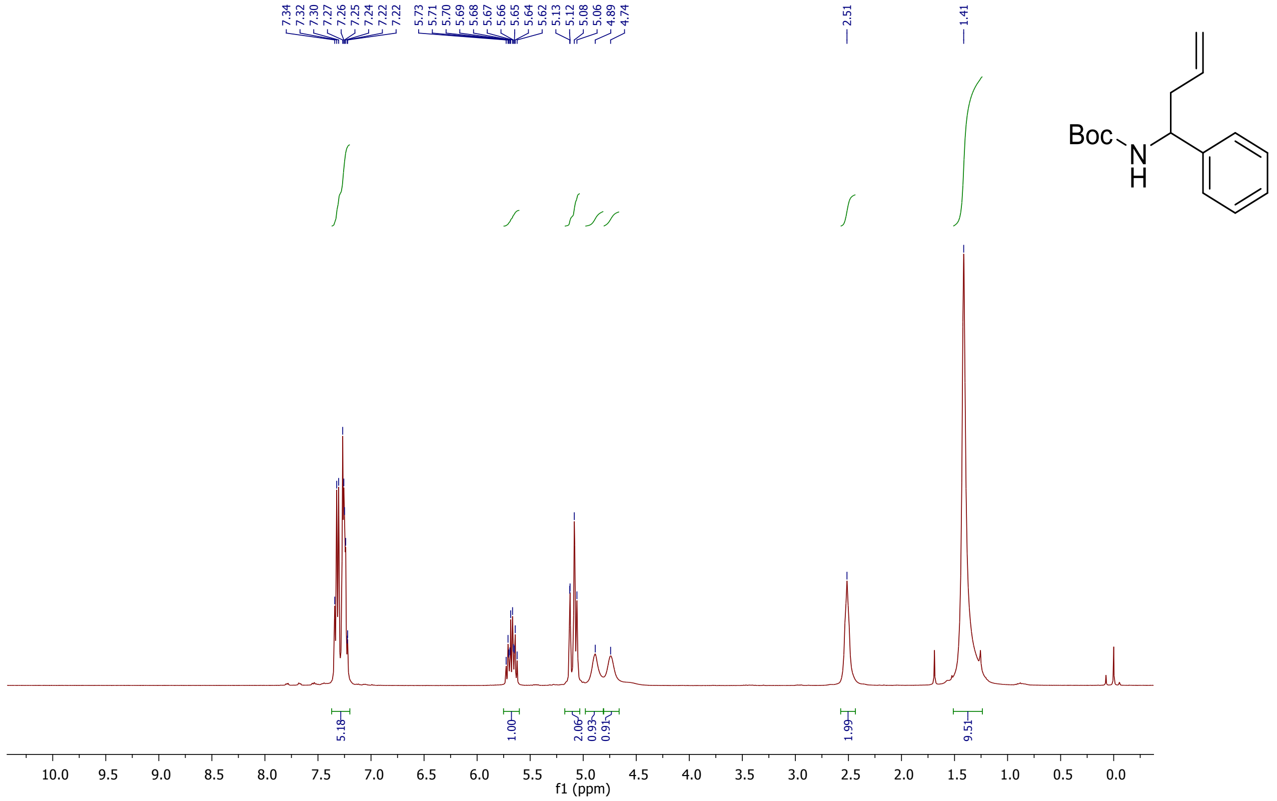

^{13}C{^{1}H} NMR (101 MHz, $CDCl_3$)

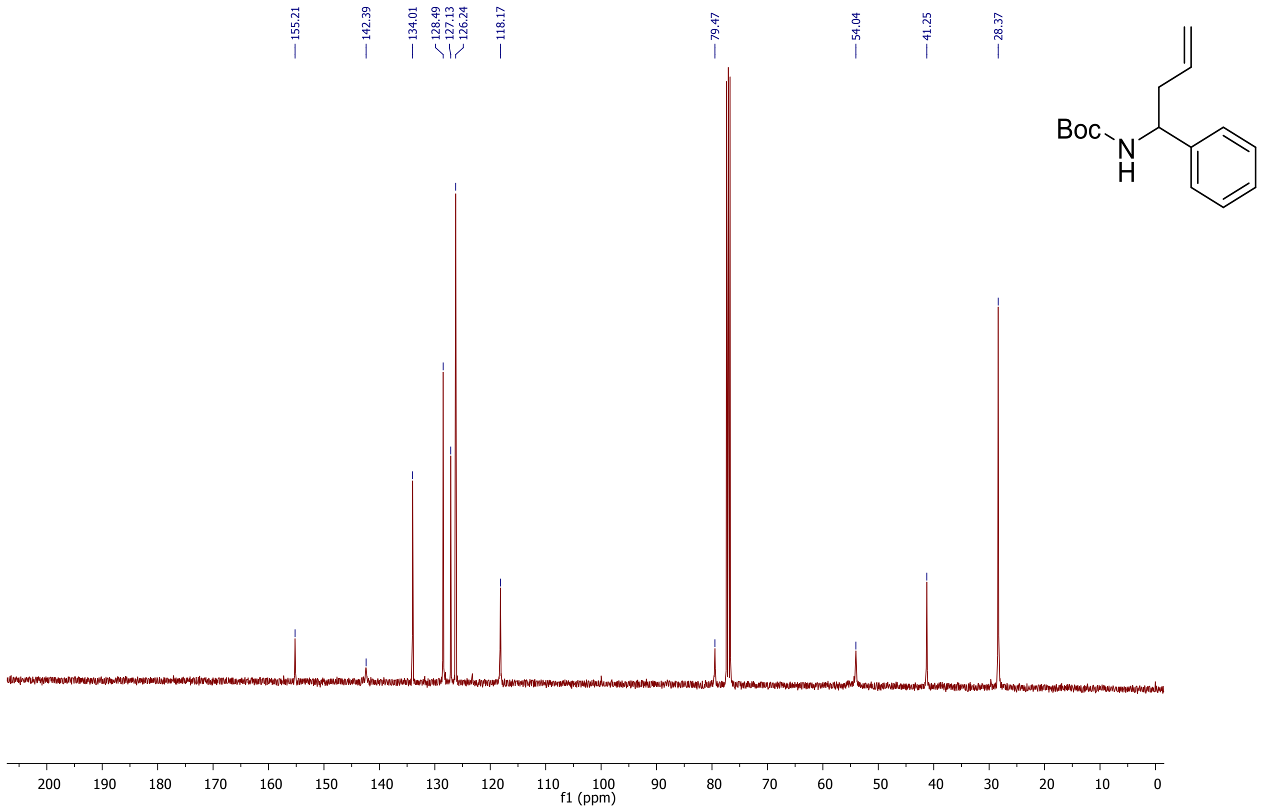

***tert*-butyl (1-(4-methoxyphenyl)but-3-en-1-yl)carbamate (5b)**

^{1}H NMR (400 MHz, $CDCl_3$)

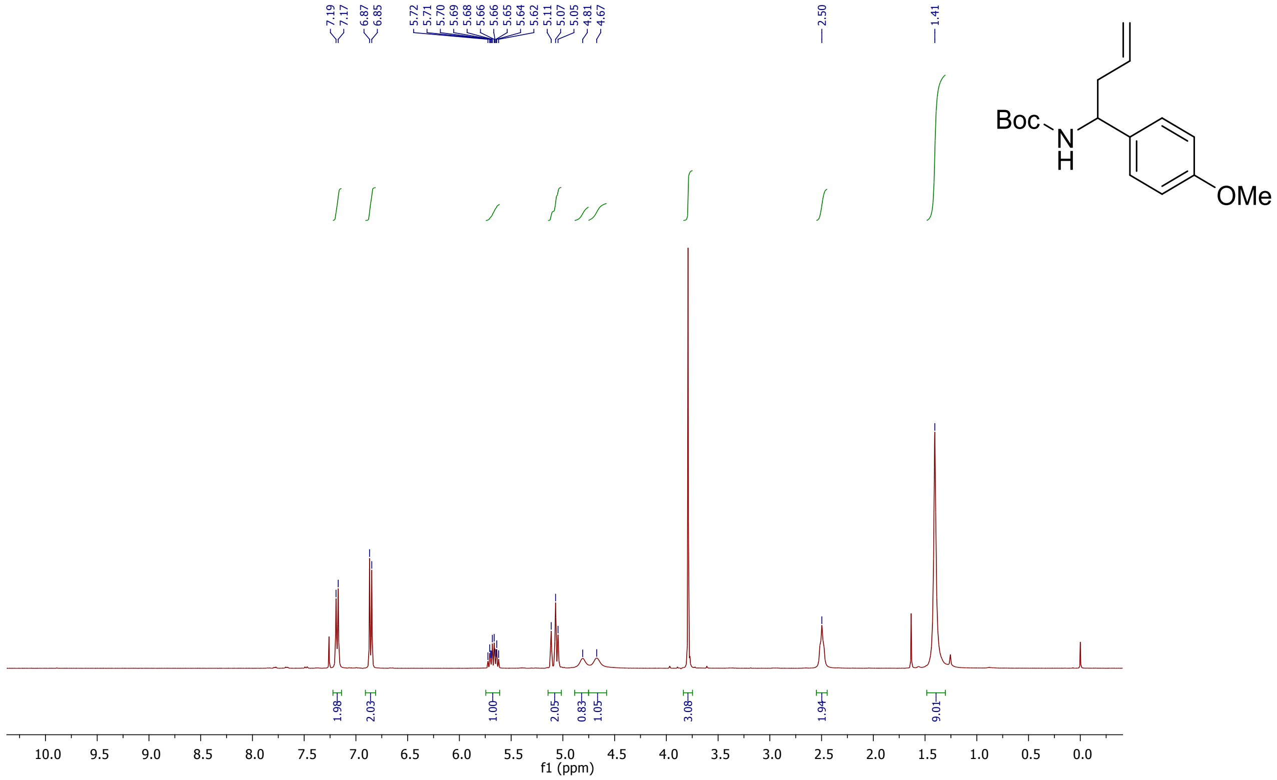

^{13}C{^{1}H} NMR (101 MHz, $CDCl_3$)

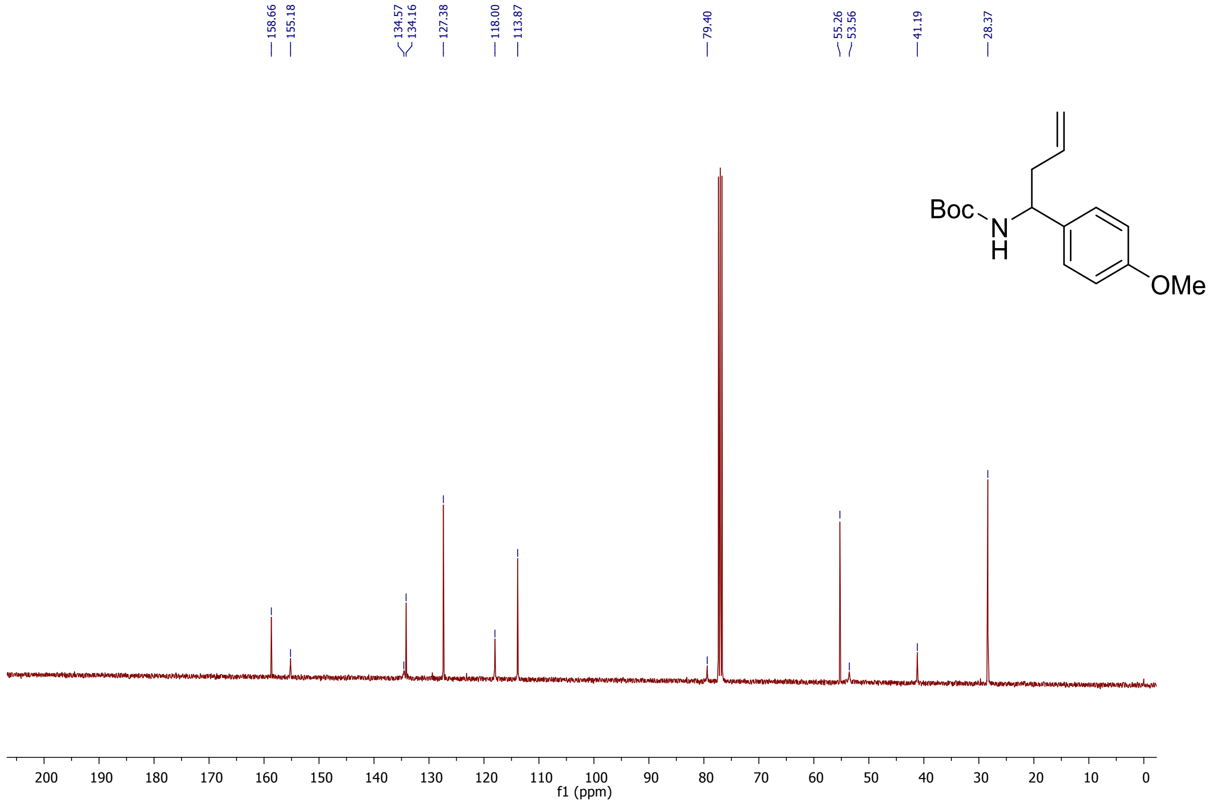

***tert*-butyl (1-(p-tolyl)but-3-en-1-yl)carbamate (5c)**

^{1}H NMR (400 MHz, $CDCl_3$)

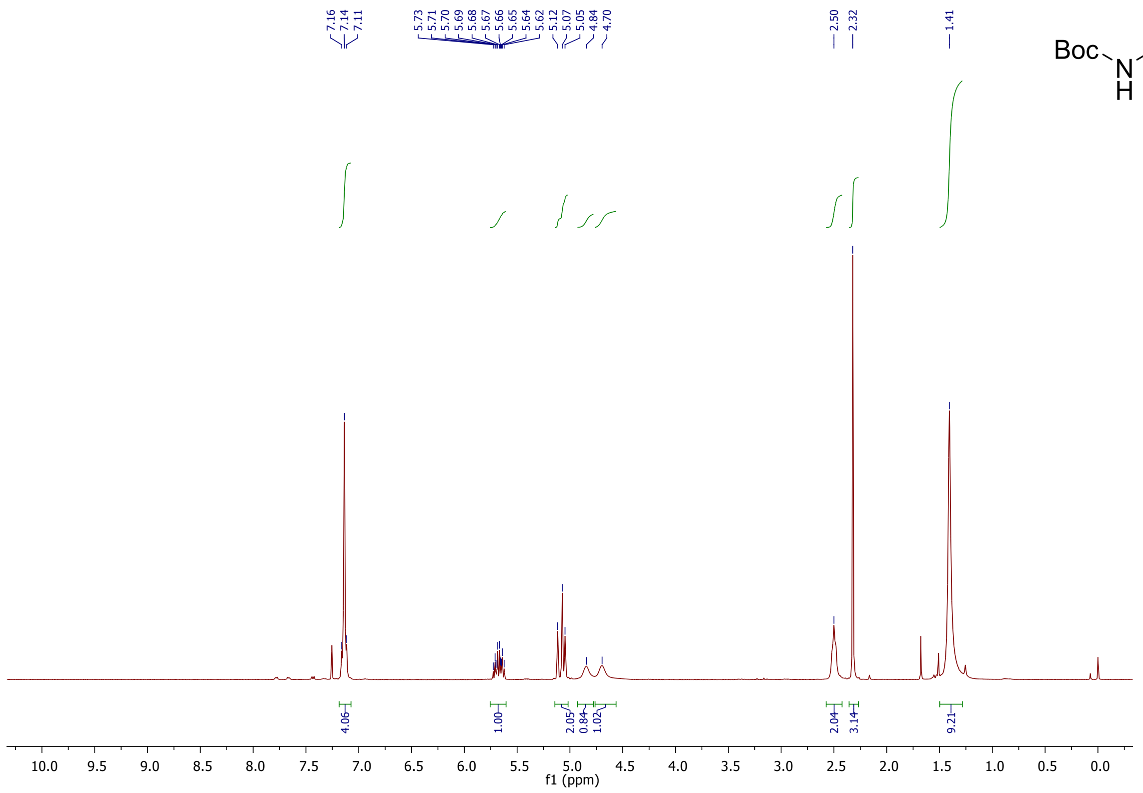

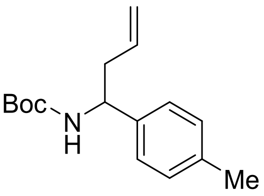

^{13}C{^{1}H} NMR (101 MHz, $CDCl_3$)

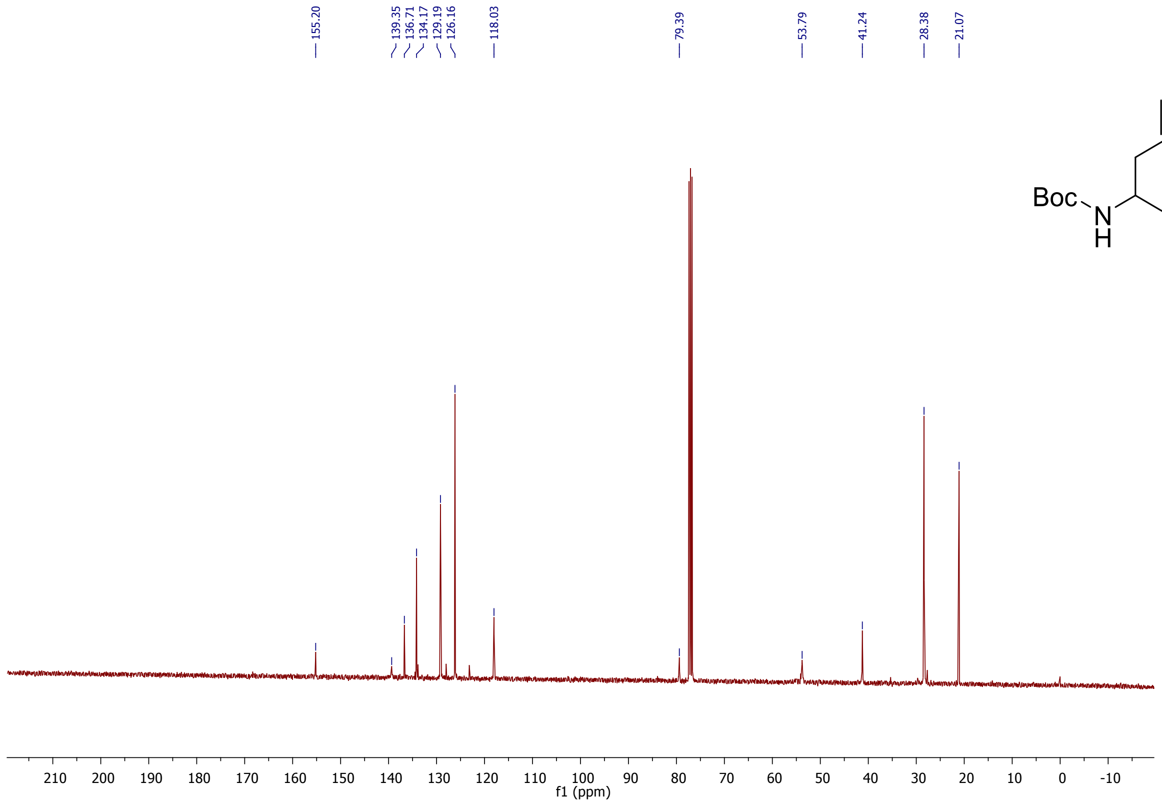

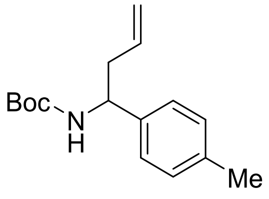

***tert*-butyl (1-(2-methoxyphenyl)but-3-en-1-yl)carbamate (5d)**

^{1}H NMR (400 MHz, $CDCl_3$)

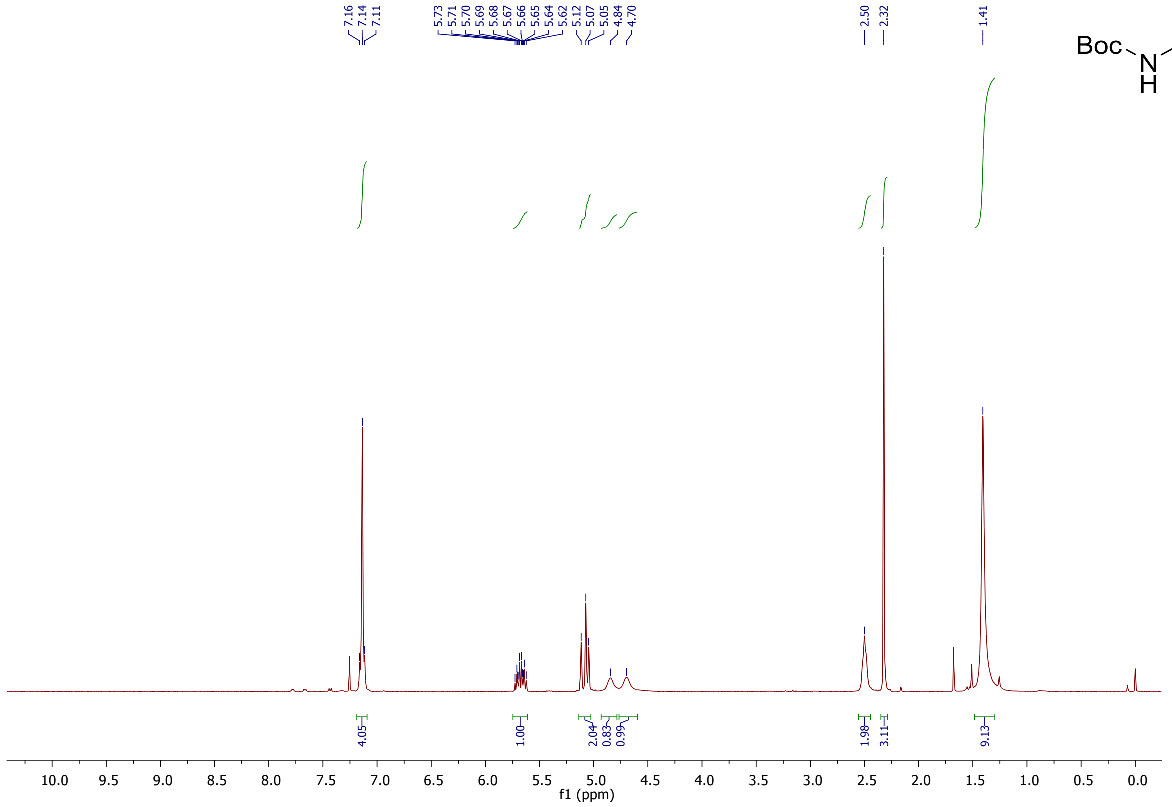

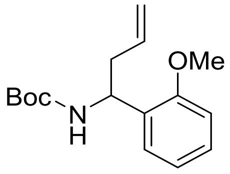

$^{13}C\{^{1}H\}$ NMR (101 MHz, $CDCl_3$)

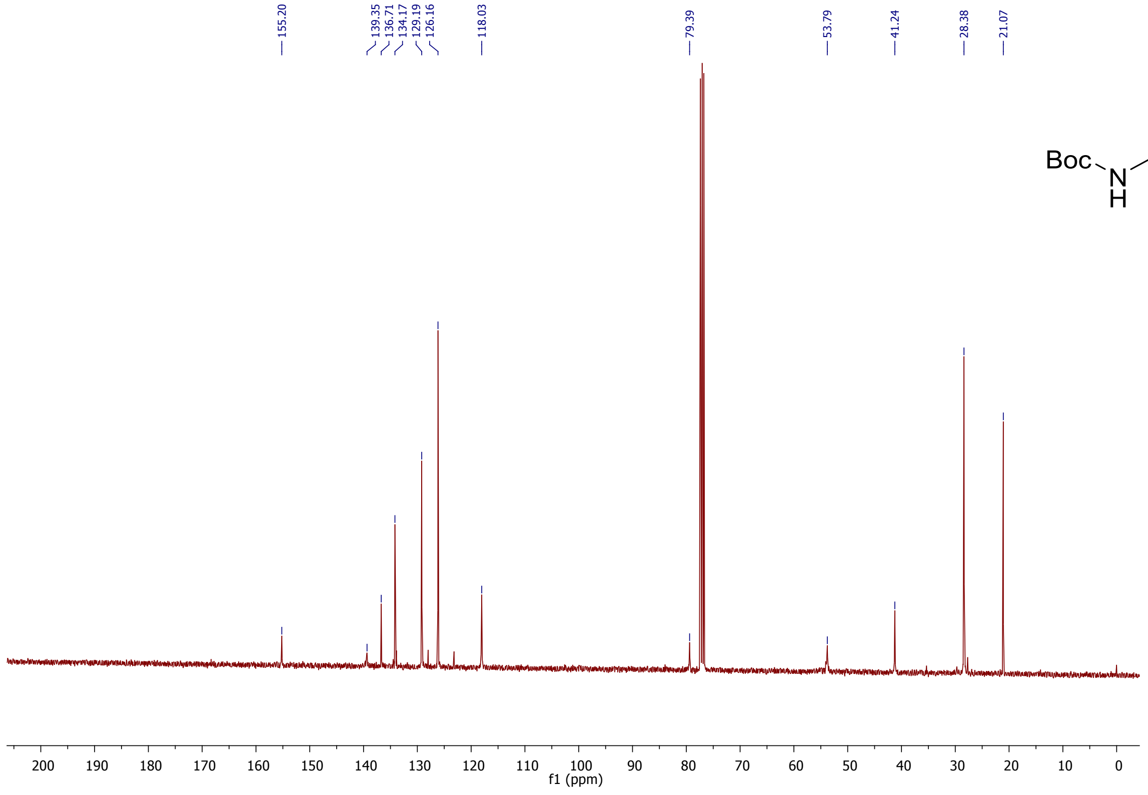

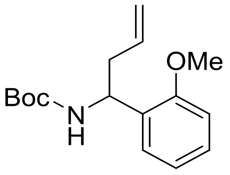

***tert*-butyl (1-(4-fluorophenyl)but-3-en-1-yl)carbamate (5e)**

^{1}H NMR (400 MHz, $CDCl_3$)

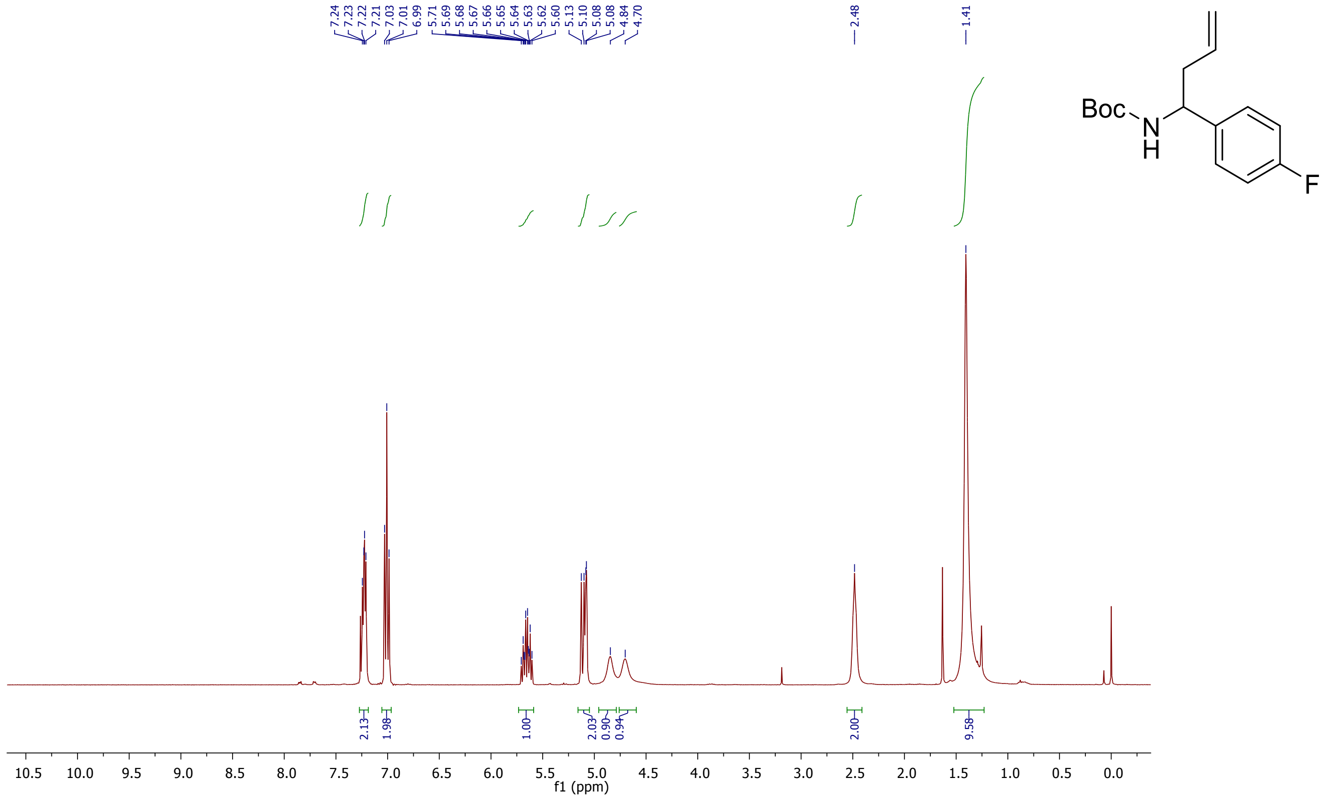

$^{13}C\{^{1}H\}$ NMR (101 MHz, $CDCl_3$)

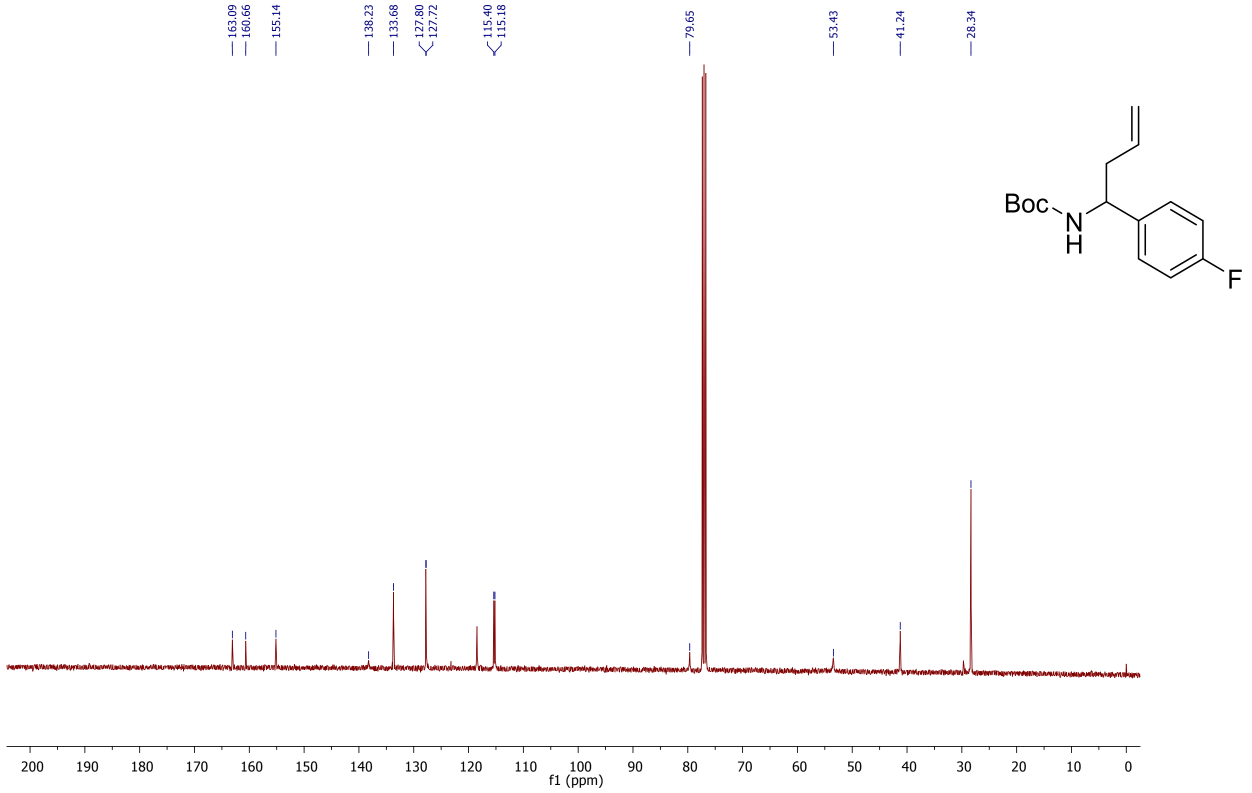

***tert*-butyl (1-(3-chlorophenyl)but-3-en-1-yl)carbamate (5f)**

^1H NMR (400 MHz, $CDCl_3$)

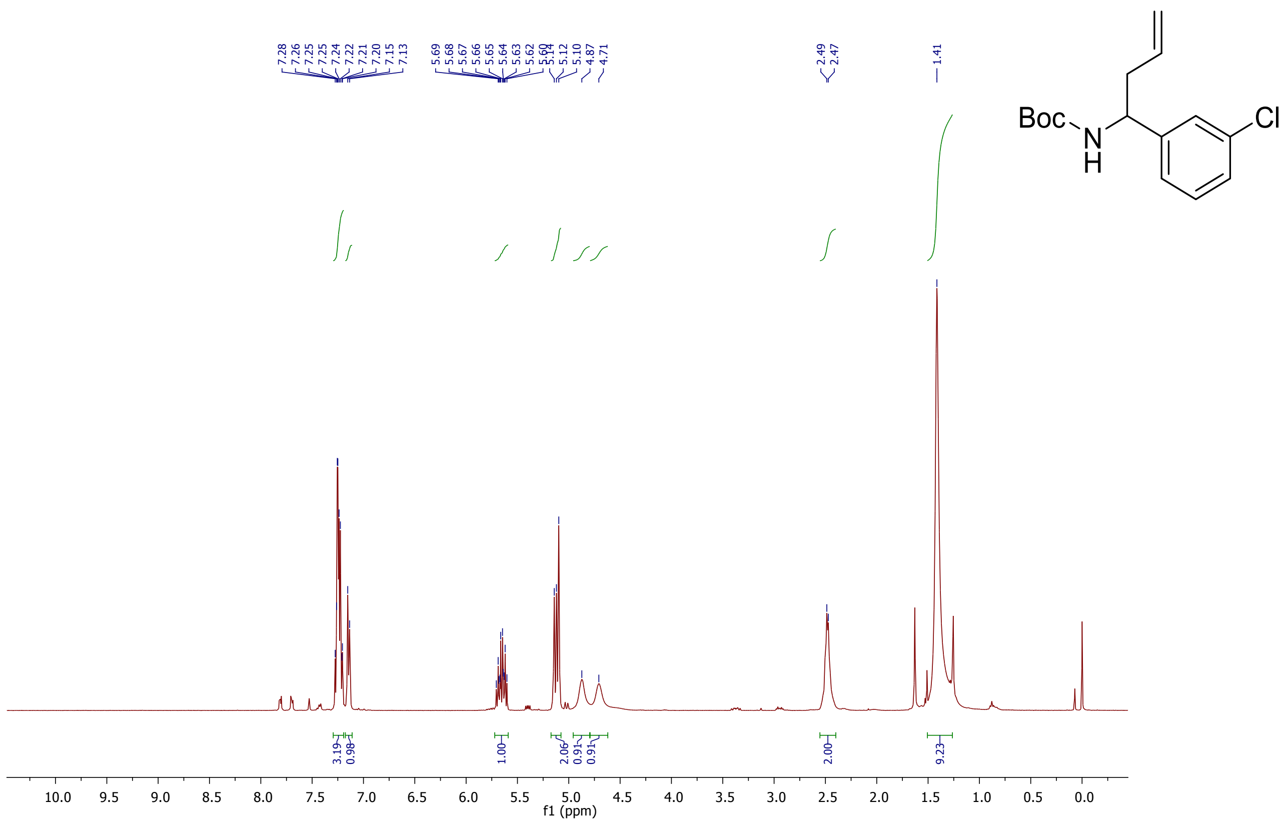

$^{13}C\{^1H\}$ NMR (101 MHz, $CDCl_3$)

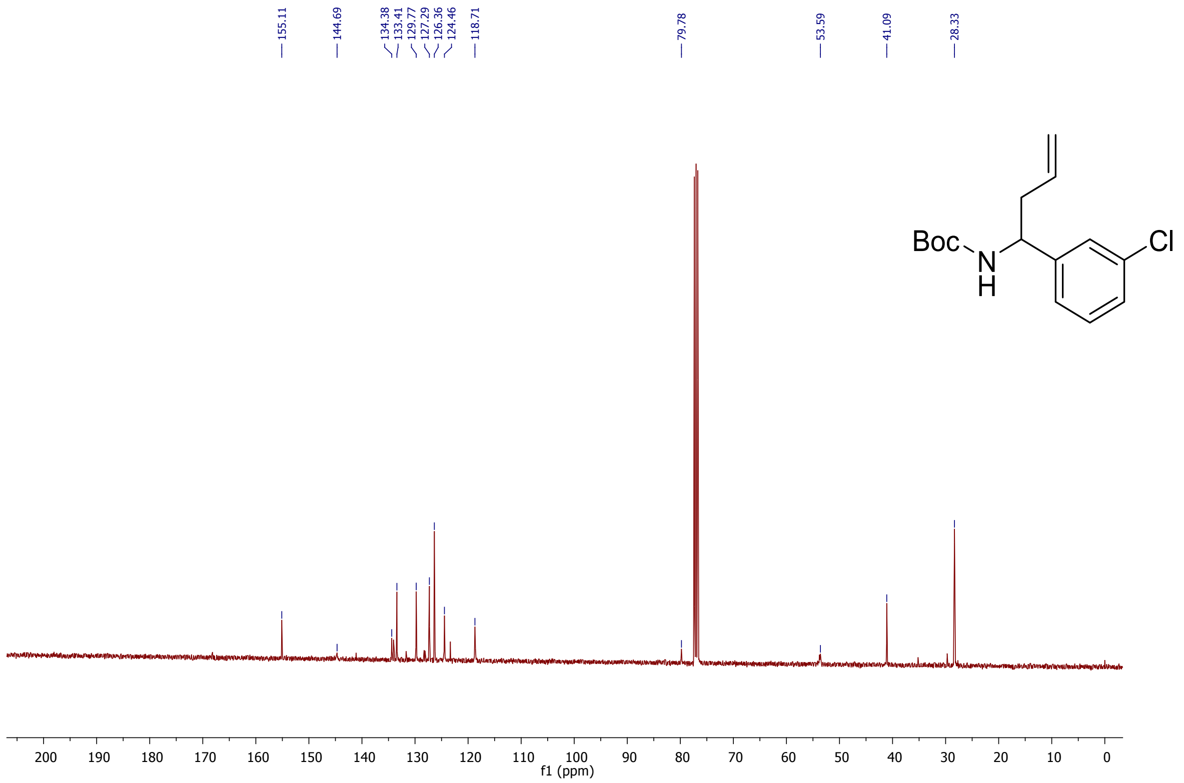

***tert*-butyl (1-(2-bromophenyl)but-3-en-1-yl)carbamate (5g)**

^{1}H NMR (400 MHz, $CDCl_3$)

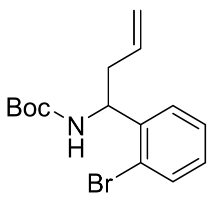

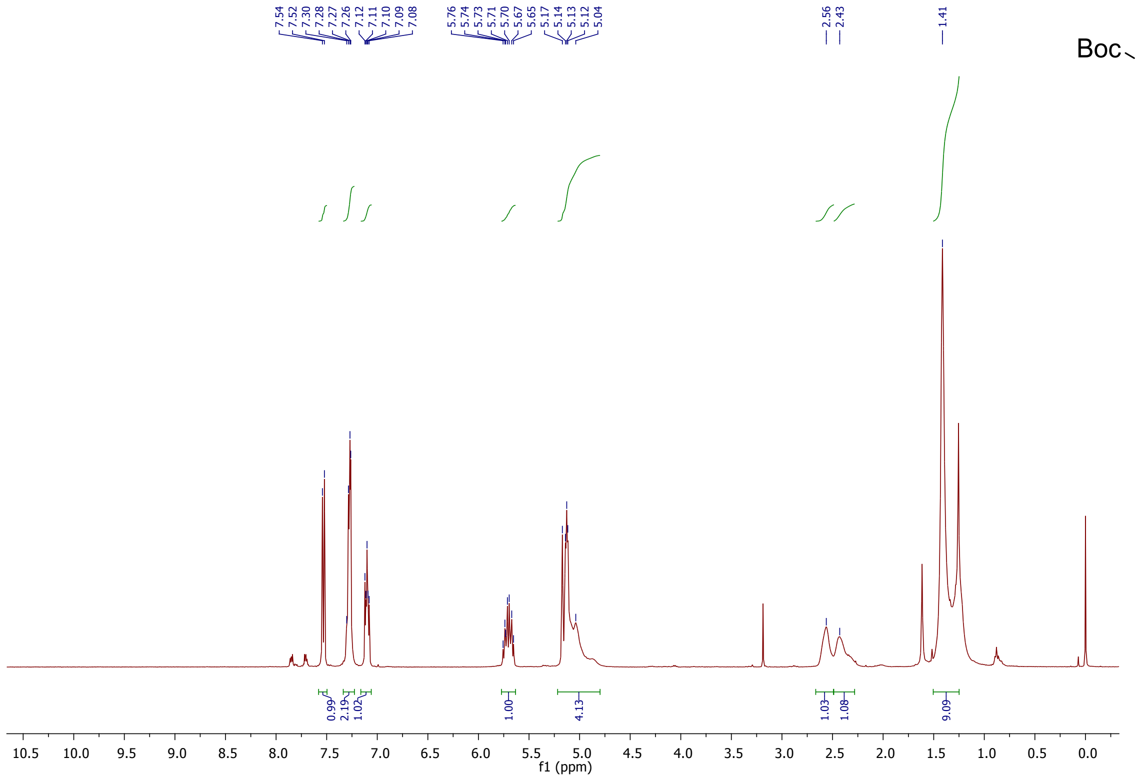

$^{13}C\{^1H\}$ NMR (101 MHz, $CDCl_3$)

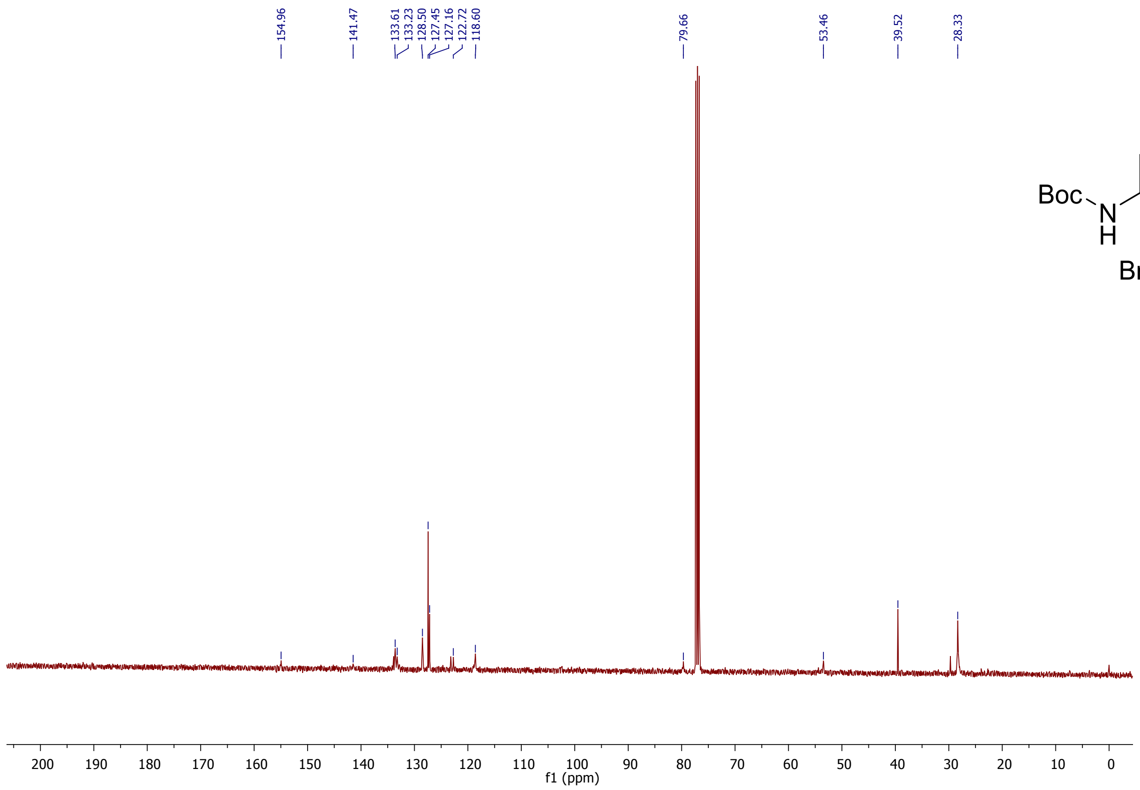

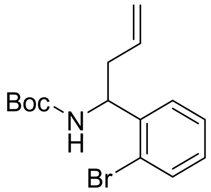

tert-butyl ((5-bromo-1-methyl-1H-indol-3-yl)(phenyl)methyl)carbamate (5h)

^{1}H NMR (400 MHz, $CDCl_3$)

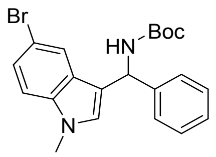

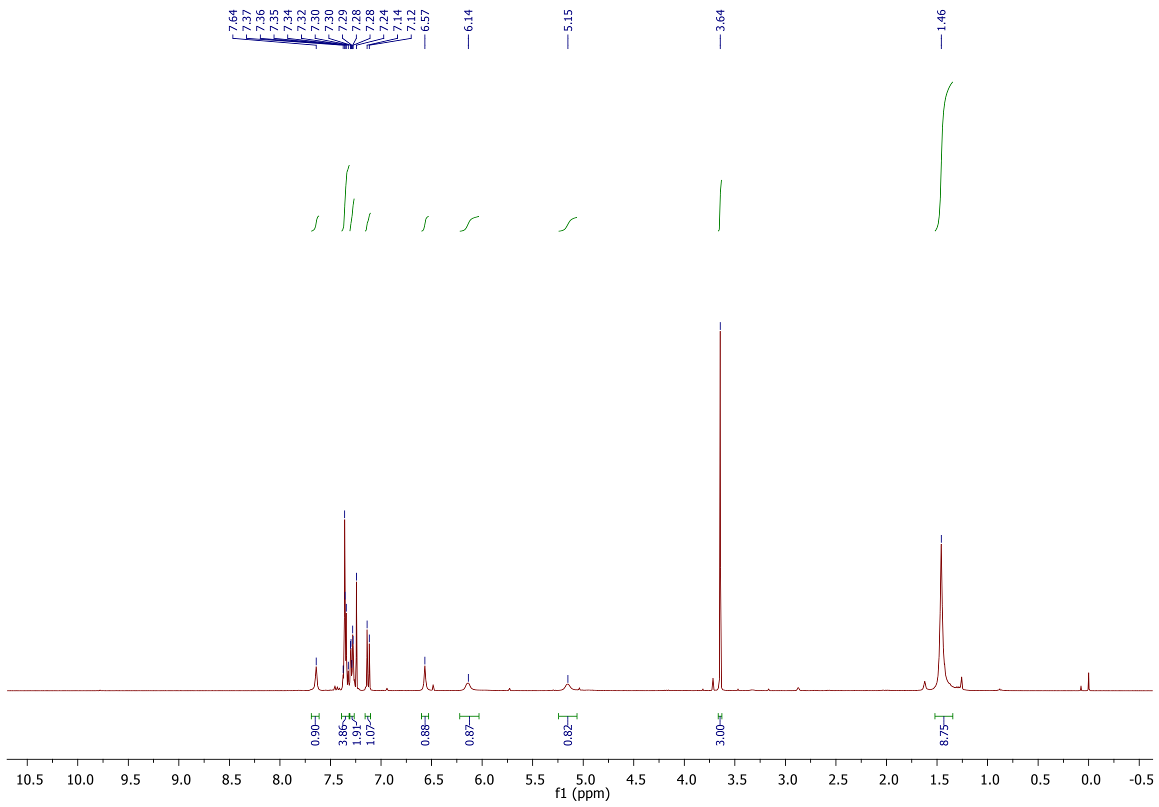

$^{13}C\{^{1}H\}$ NMR (101 MHz, $CDCl_3$)

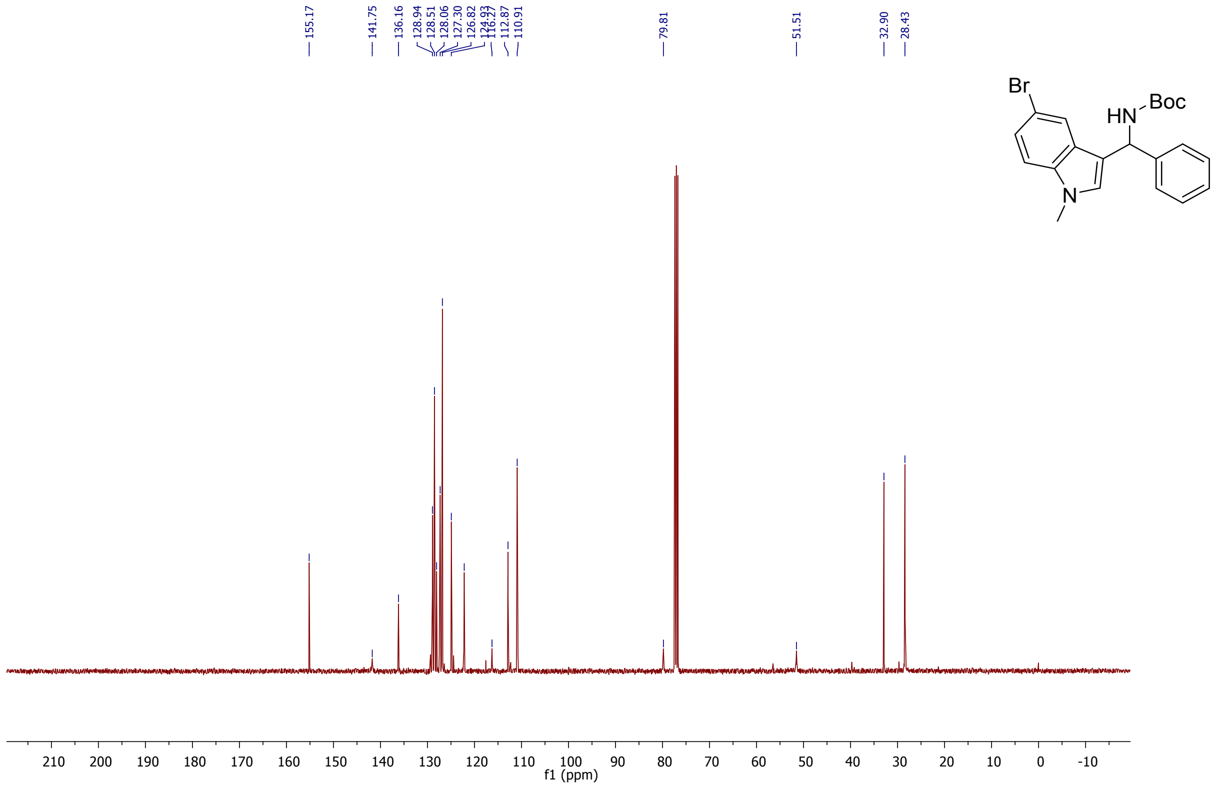

tert-butyl ((5-bromo-1-methyl-1H-indol-3-yl)(4-methoxyphenyl)methyl)carbamate (5i)

^{1}H NMR (400 MHz, $CDCl_3$)

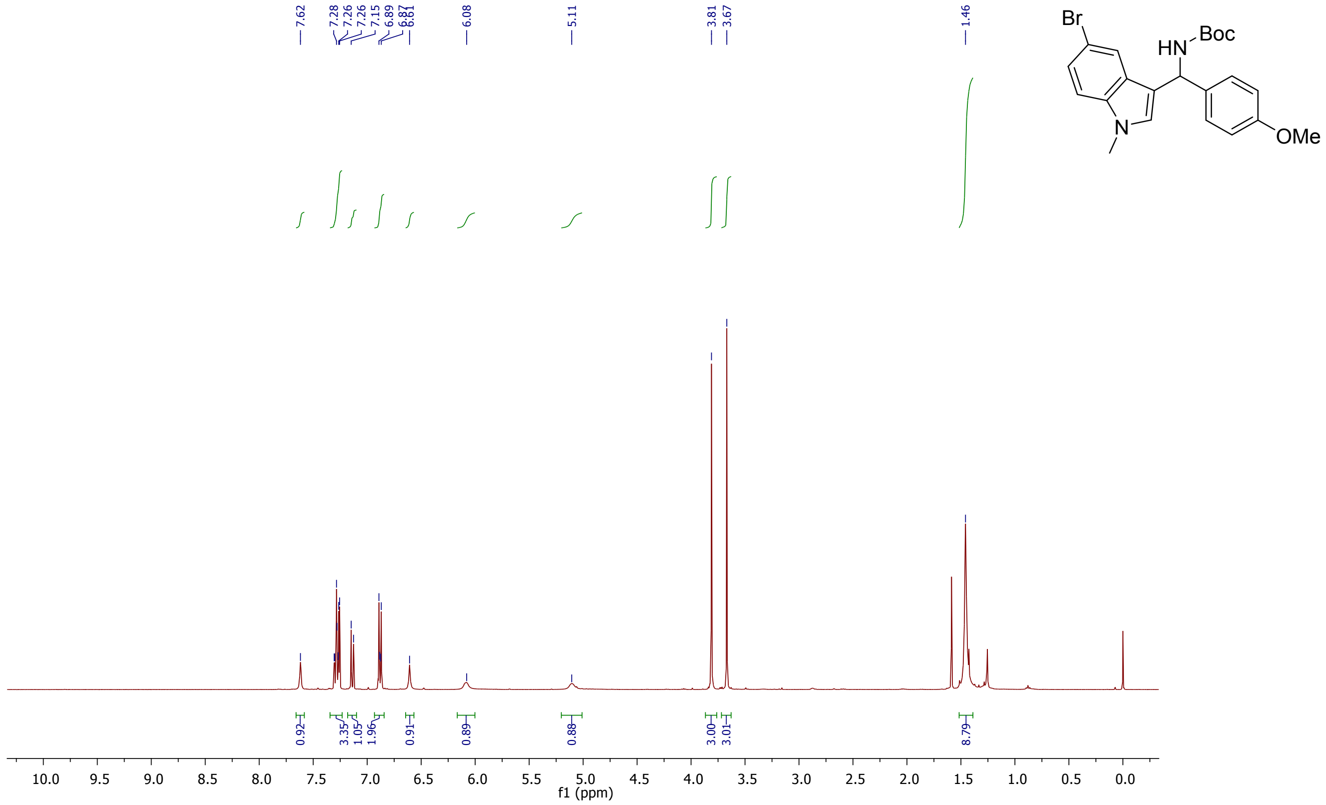

$^{13}C\{^{1}H\}$ NMR (101 MHz, $CDCl_3$)

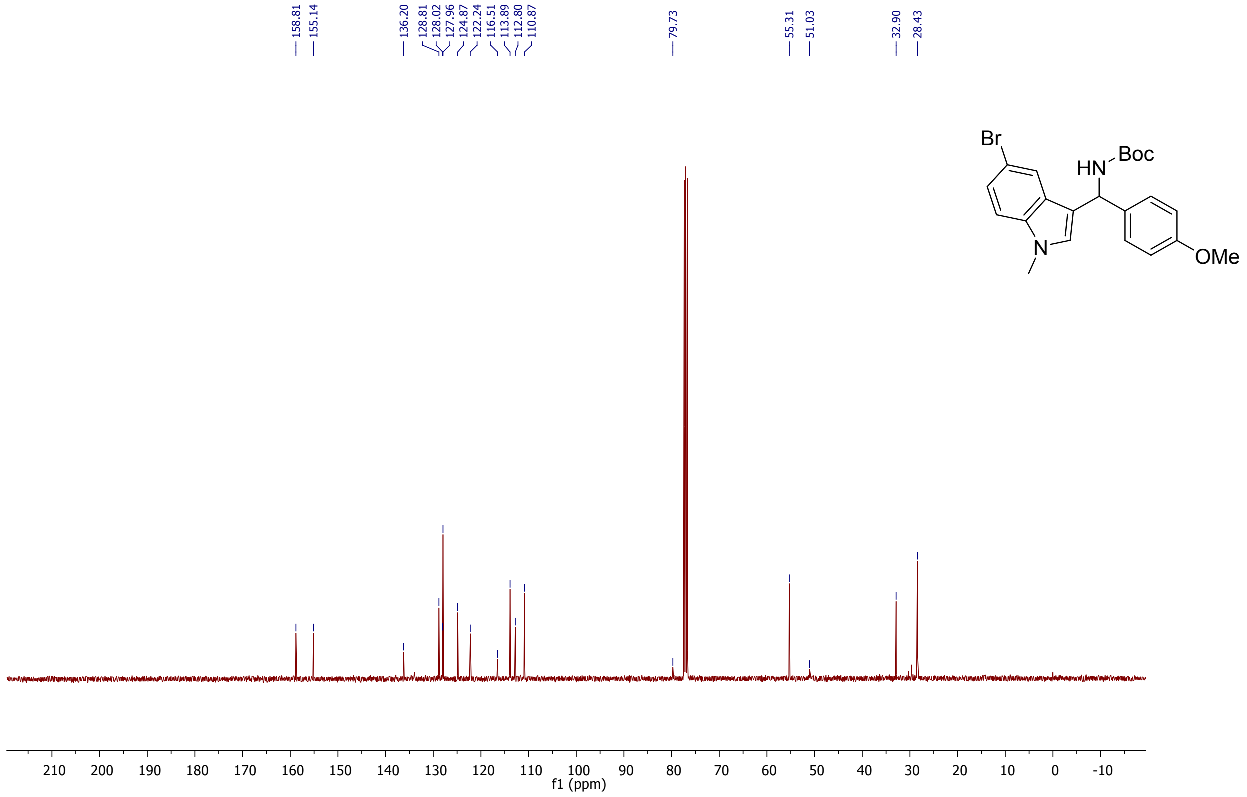

(9H-fluoren-9-yl)methyl (1-phenylbut-3-en-1-yl)carbamate (6a)

^{1}H NMR (400 MHz, $CDCl_3$)

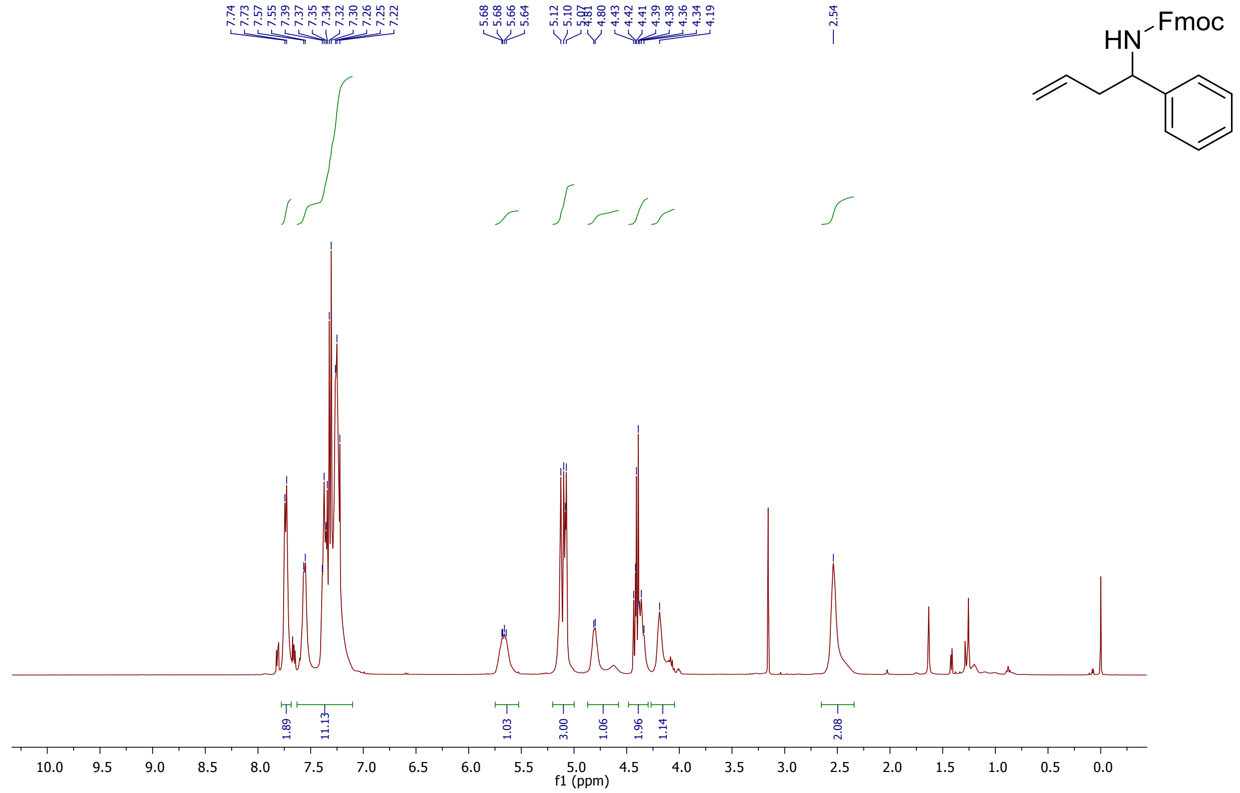

$^{13}C\{^{1}H\}$ NMR (101 MHz, $CDCl_3$)

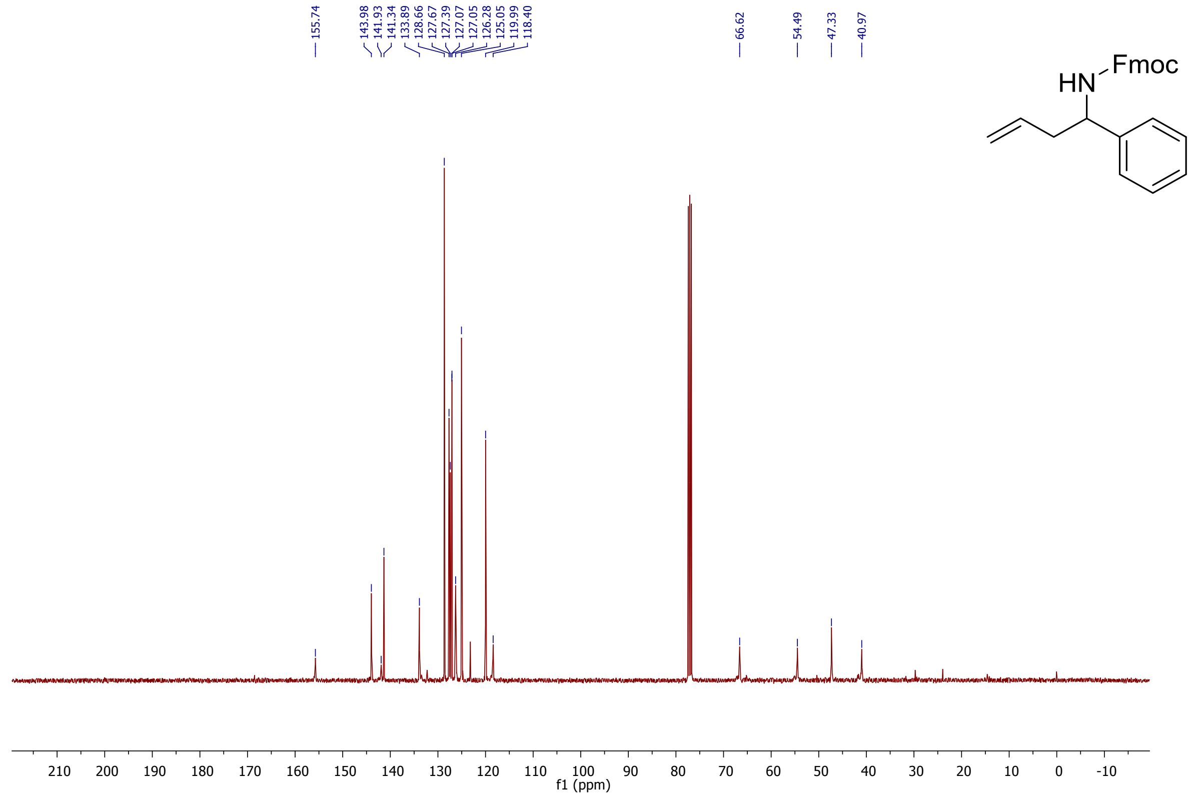

N-(1-phenylbut-3-en-1-yl)benzamide (6b)

^{1}H NMR (400 MHz, $CDCl_3$)

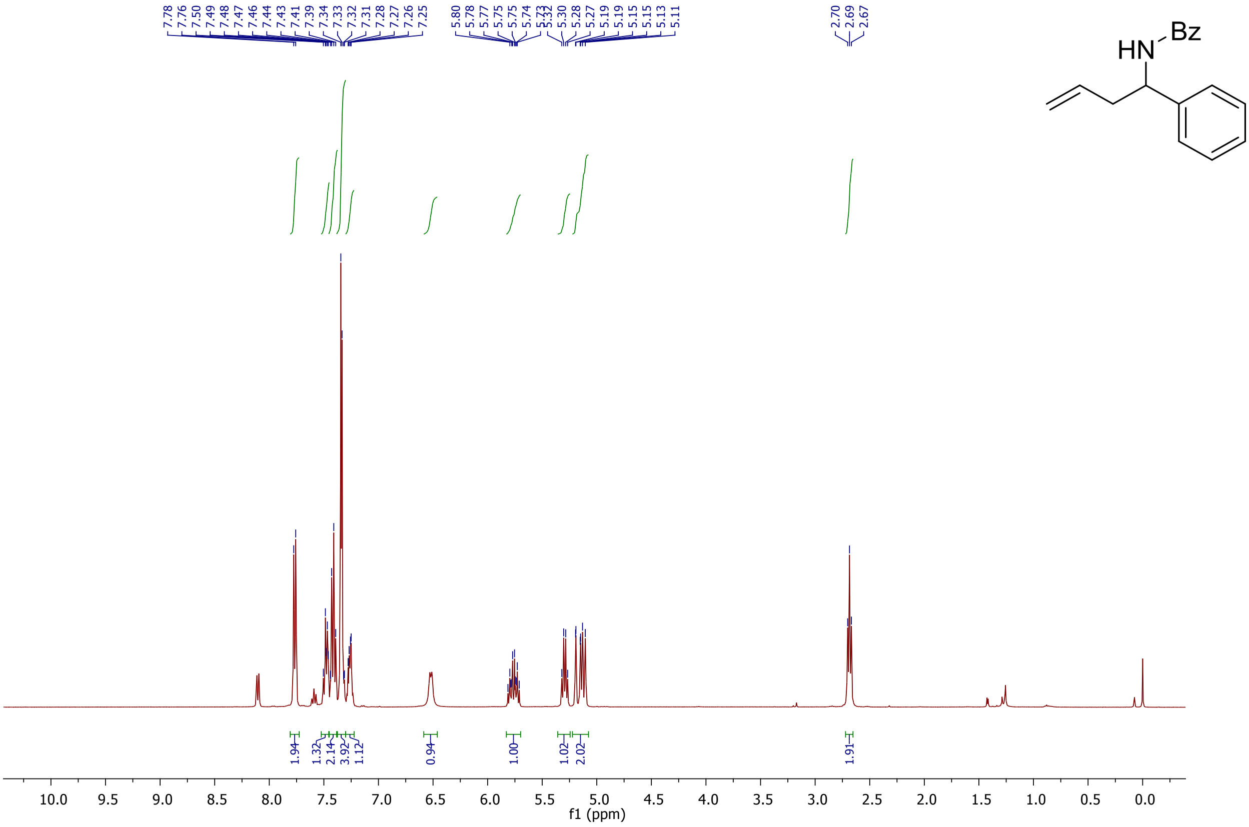

$^{13}C\{^{1}H\}$ NMR (101 MHz, $CDCl_3$)

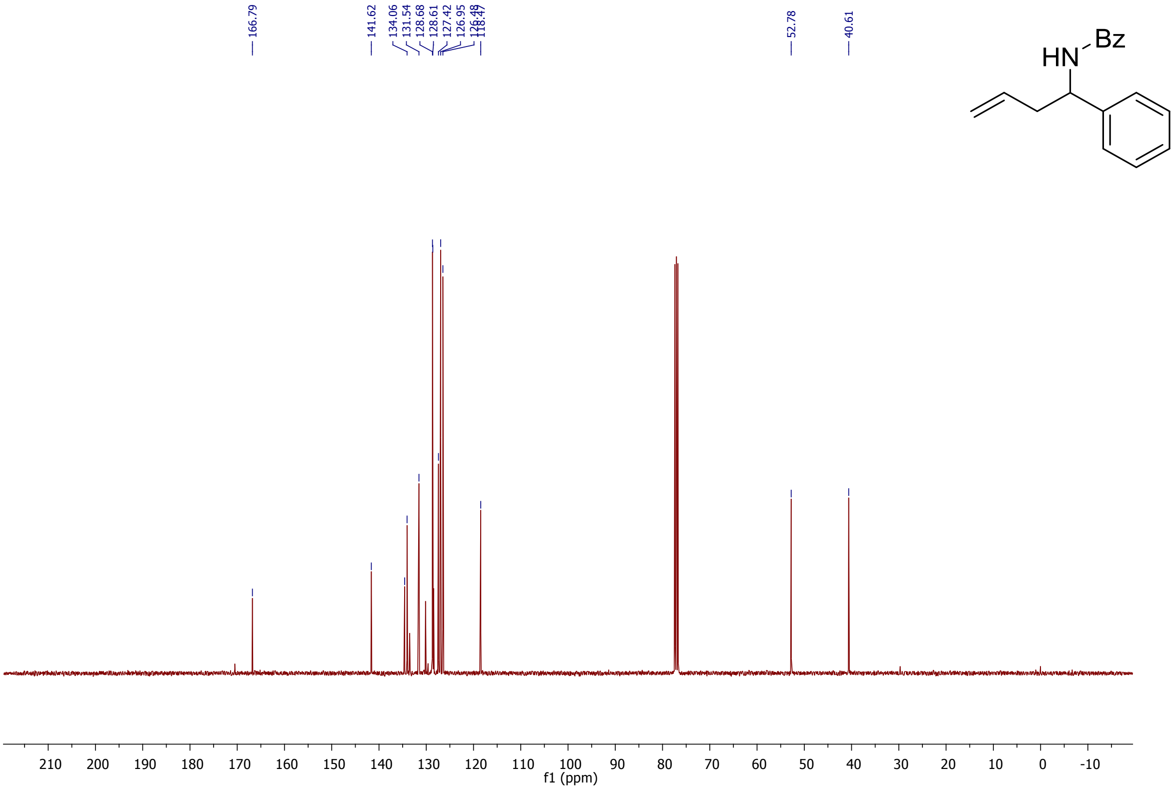

allyl (1-phenylbut-3-en-1-yl)carbamate (6c)

^{1}H NMR (400 MHz, $CDCl_3$)

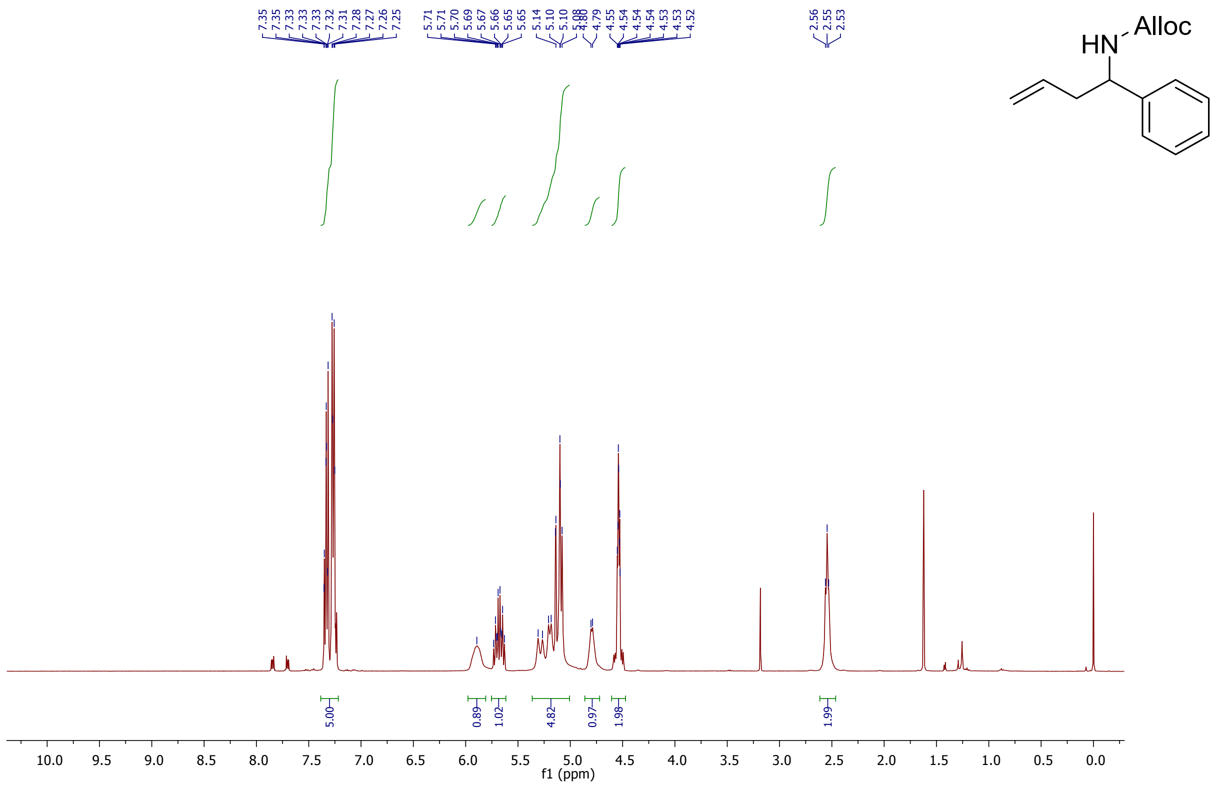

$^{13}C\{^1H\}$ NMR (101 MHz, $CDCl_3$)

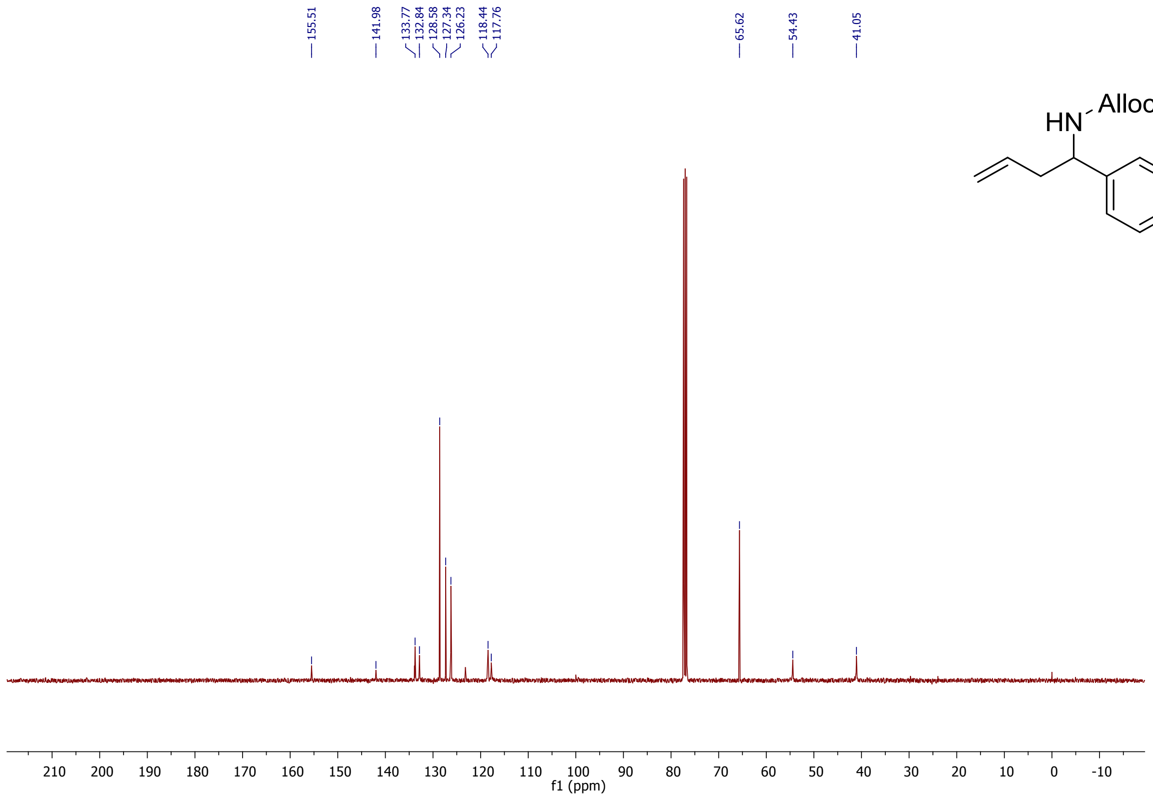

N-(1-(thiophen-2-yl)but-3-en-1-yl)acetamide (6d)

^1H NMR (400 MHz, $CDCl_3$)

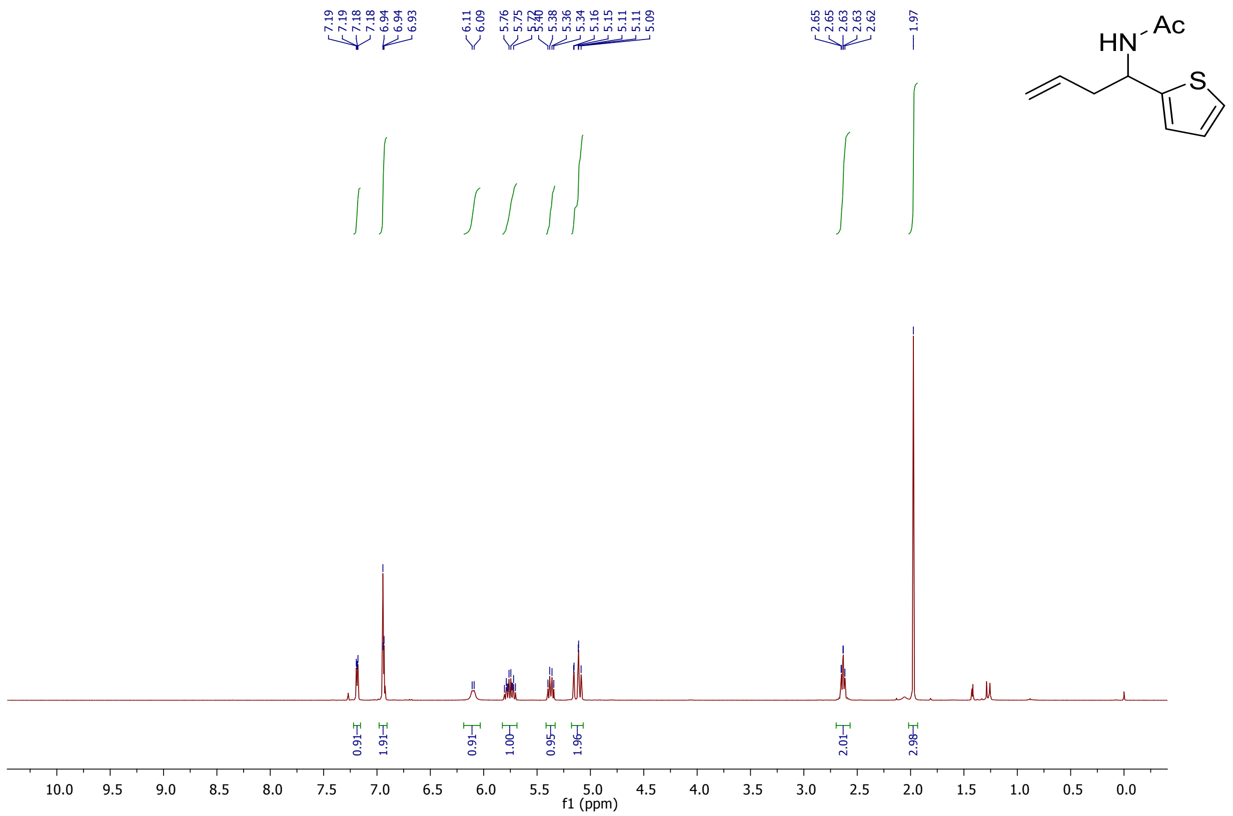

$^{13}C\{^1H\}$ NMR (101 MHz, $CDCl_3$)

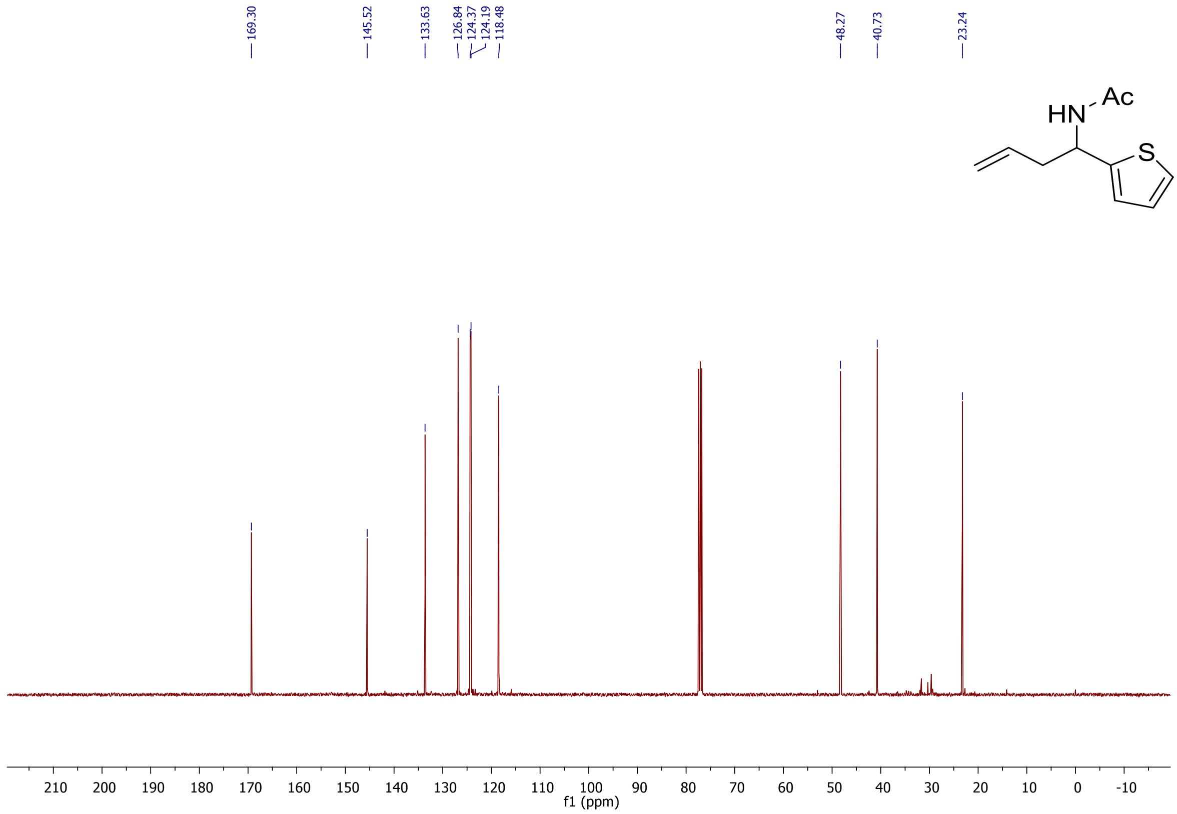

benzyl (1-(2-methoxyphenyl)but-3-en-1-yl)carbamate (6e)

^{1}H NMR (400 MHz, $CDCl_3$)

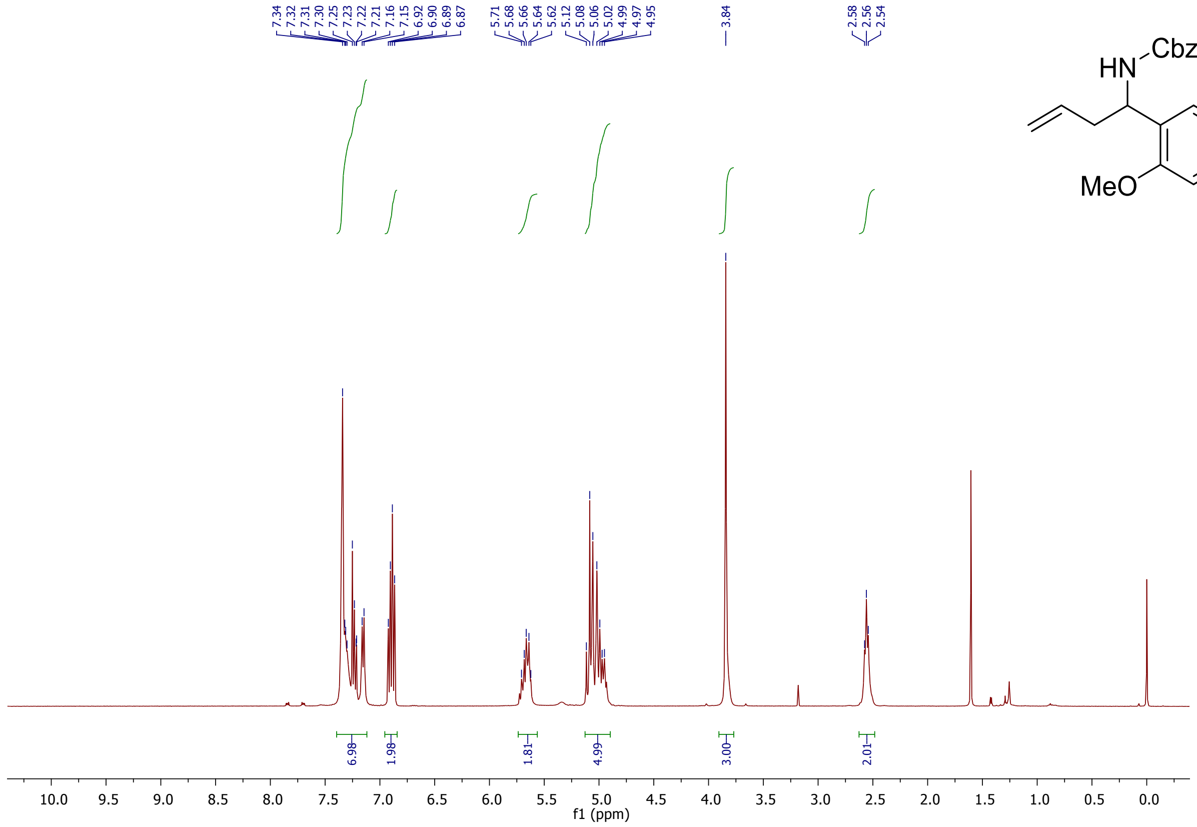

$^{13}C\{^{1}H\}$ NMR (101 MHz, $CDCl_3$)

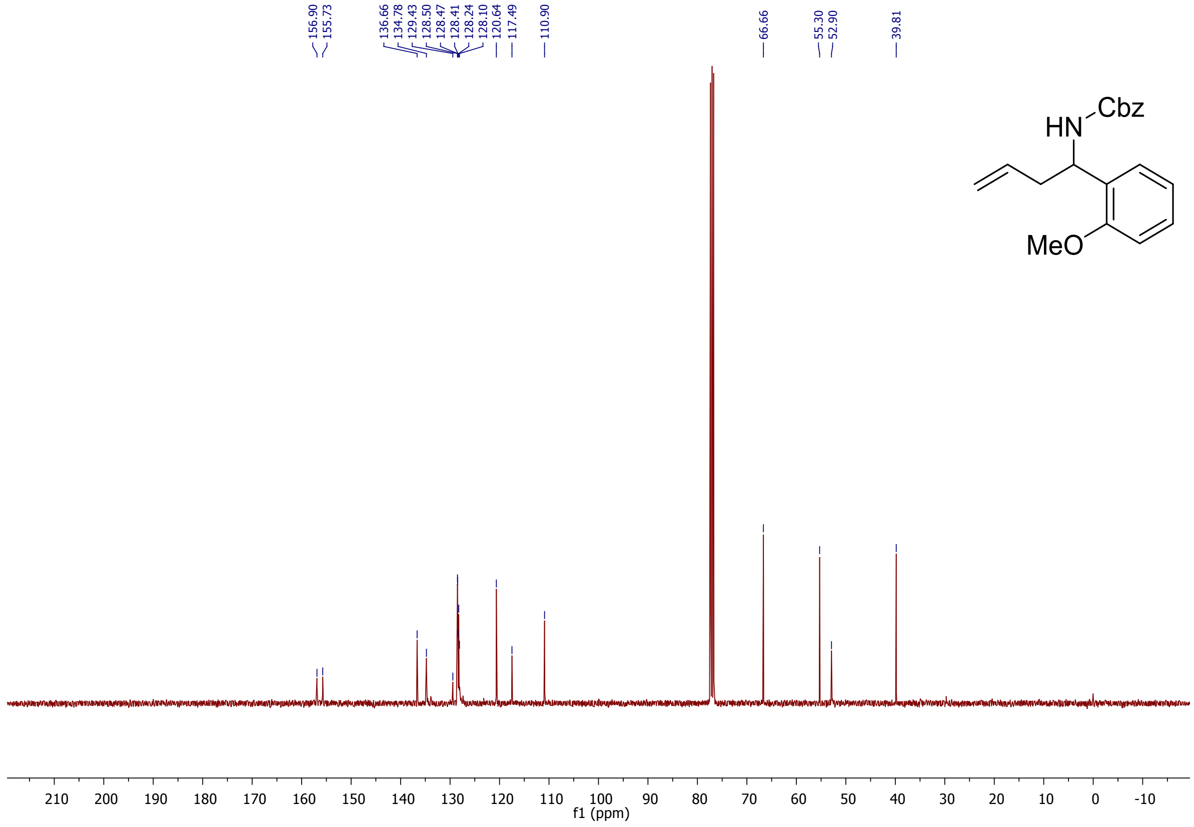

benzyl ((5-bromo-1-methyl-1H-indol-3-yl)(phenyl)methyl)carbamate (6f)

^{1}H NMR (400 MHz, $CDCl_3$)

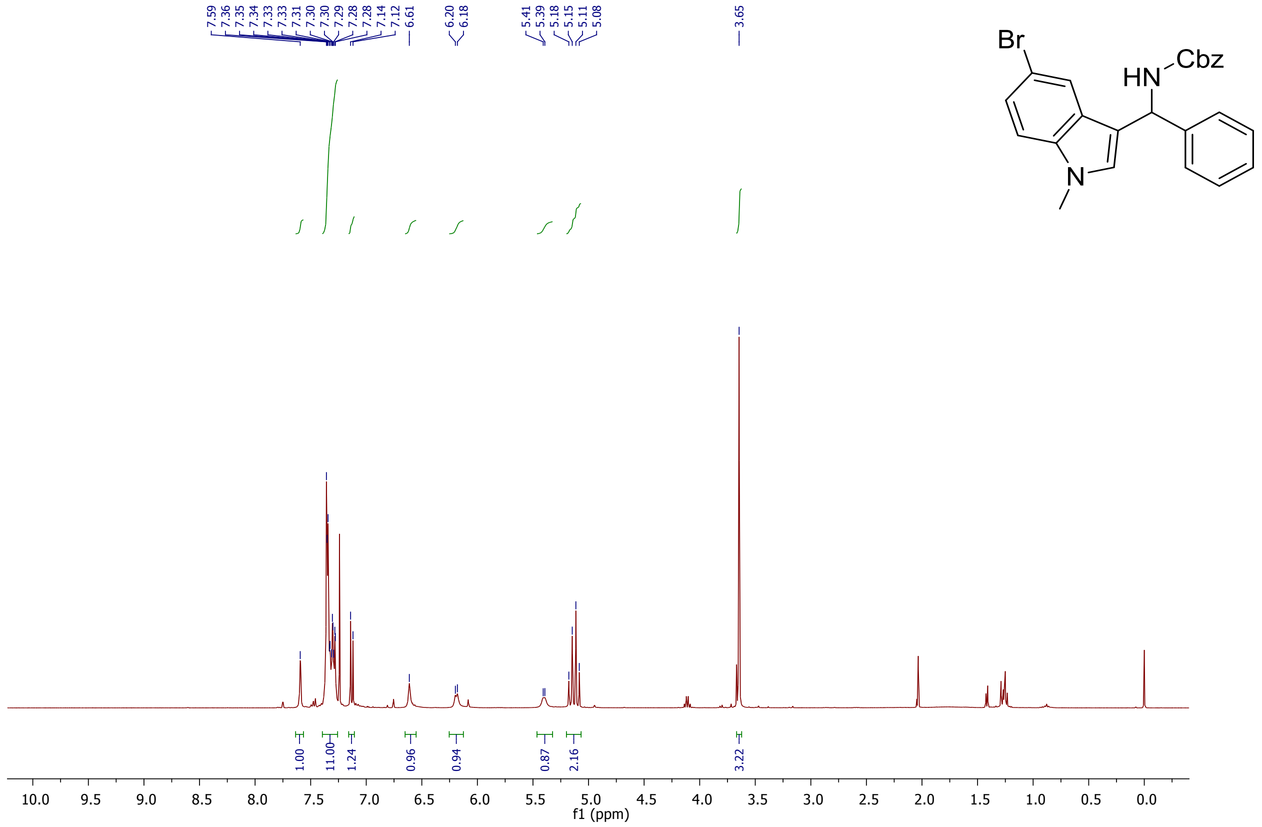

$^{13}C\{^{1}H\}$ NMR (101 MHz, $CDCl_3$)

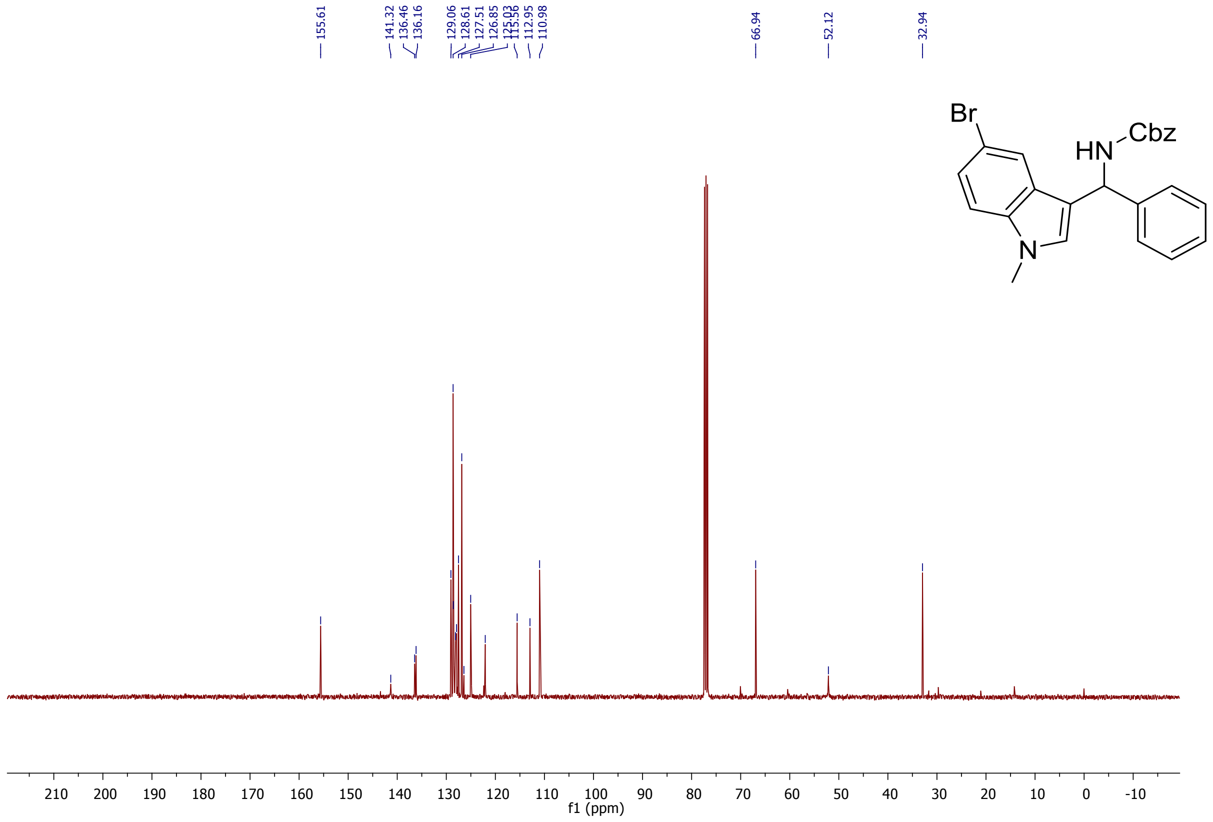

N-((5-bromo-1-methyl-1H-indol-3-yl)(phenyl)methyl)acetamide (6g)

^{1}H NMR (400 MHz, DMSO)

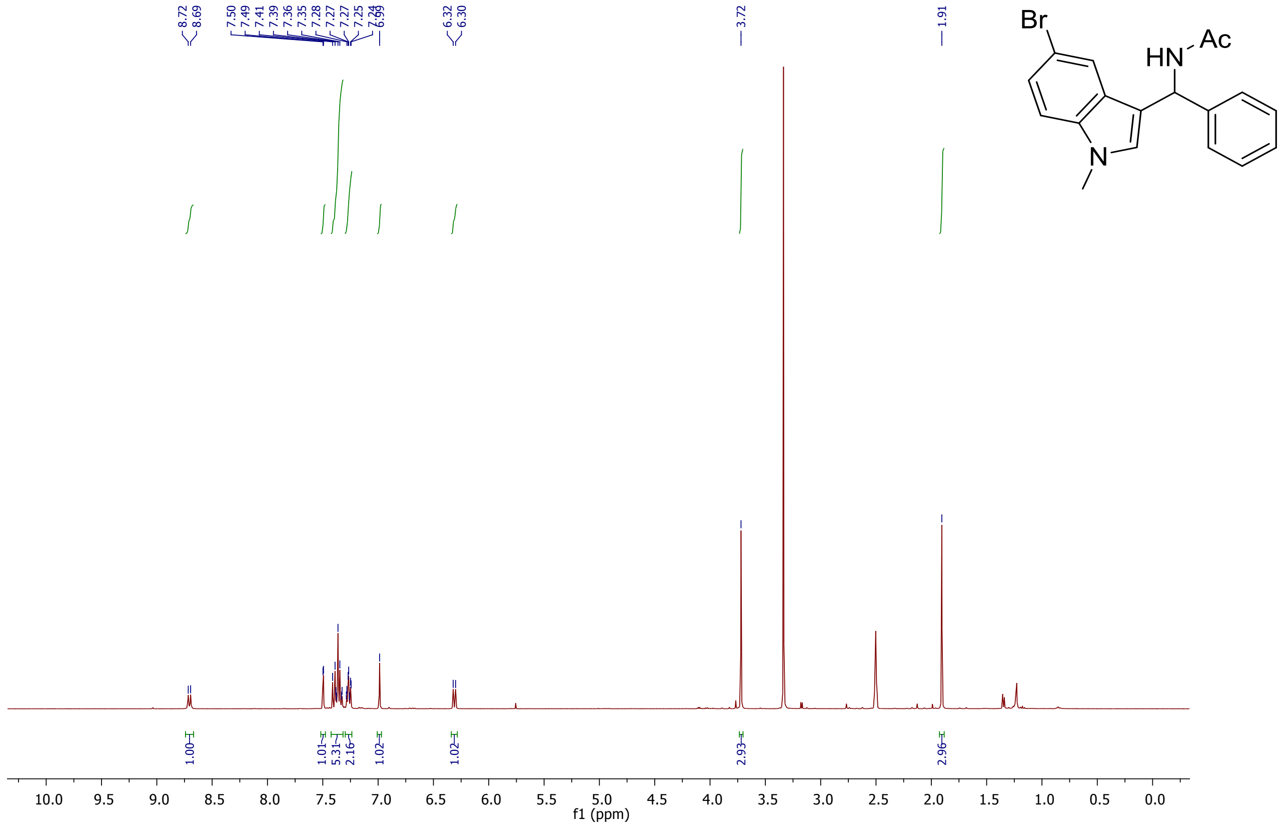

^{13}C{^{1}H} NMR (101 MHz, DMSO)

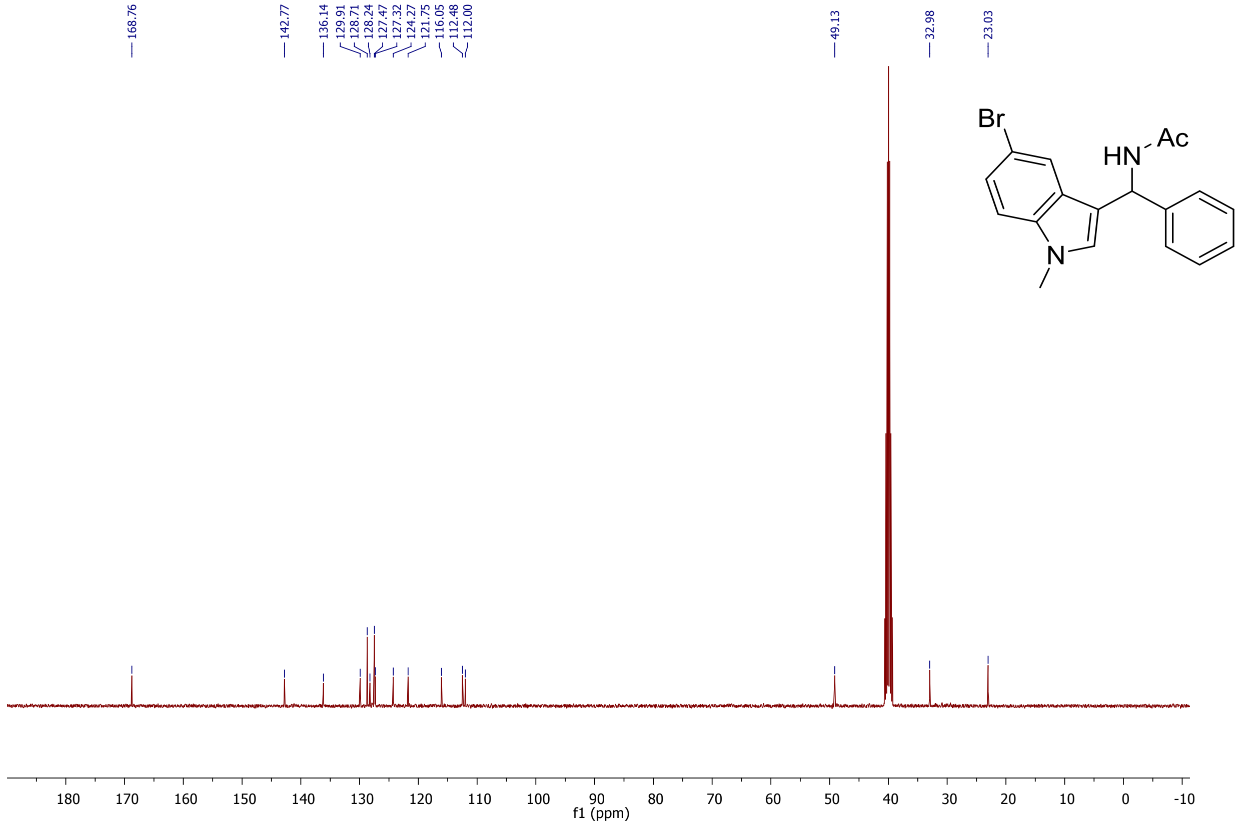

N-((5-bromo-1-methyl-1H-indol-3-yl)(phenyl)methyl)benzamide (6h)

^{1}H NMR (400 MHz, DMSO)

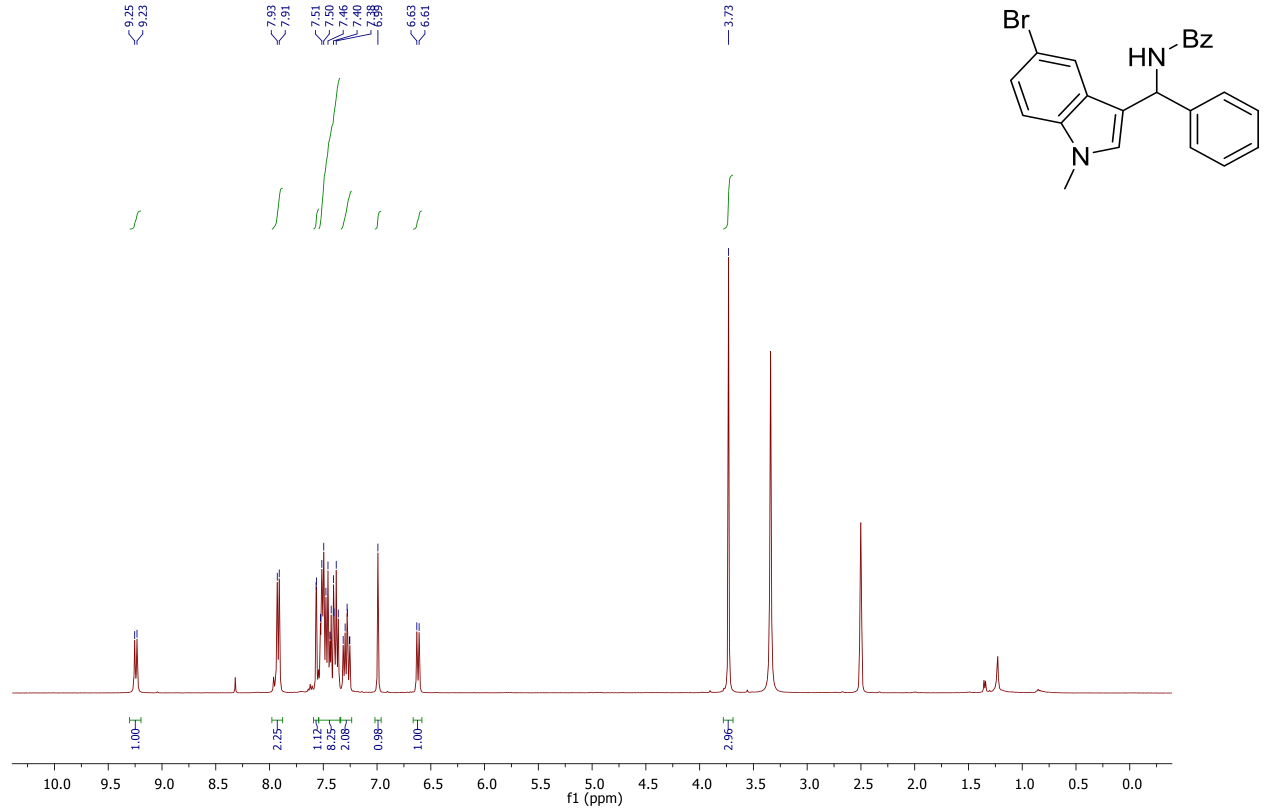

^{13}C{^{1}H} NMR (101 MHz, DMSO)

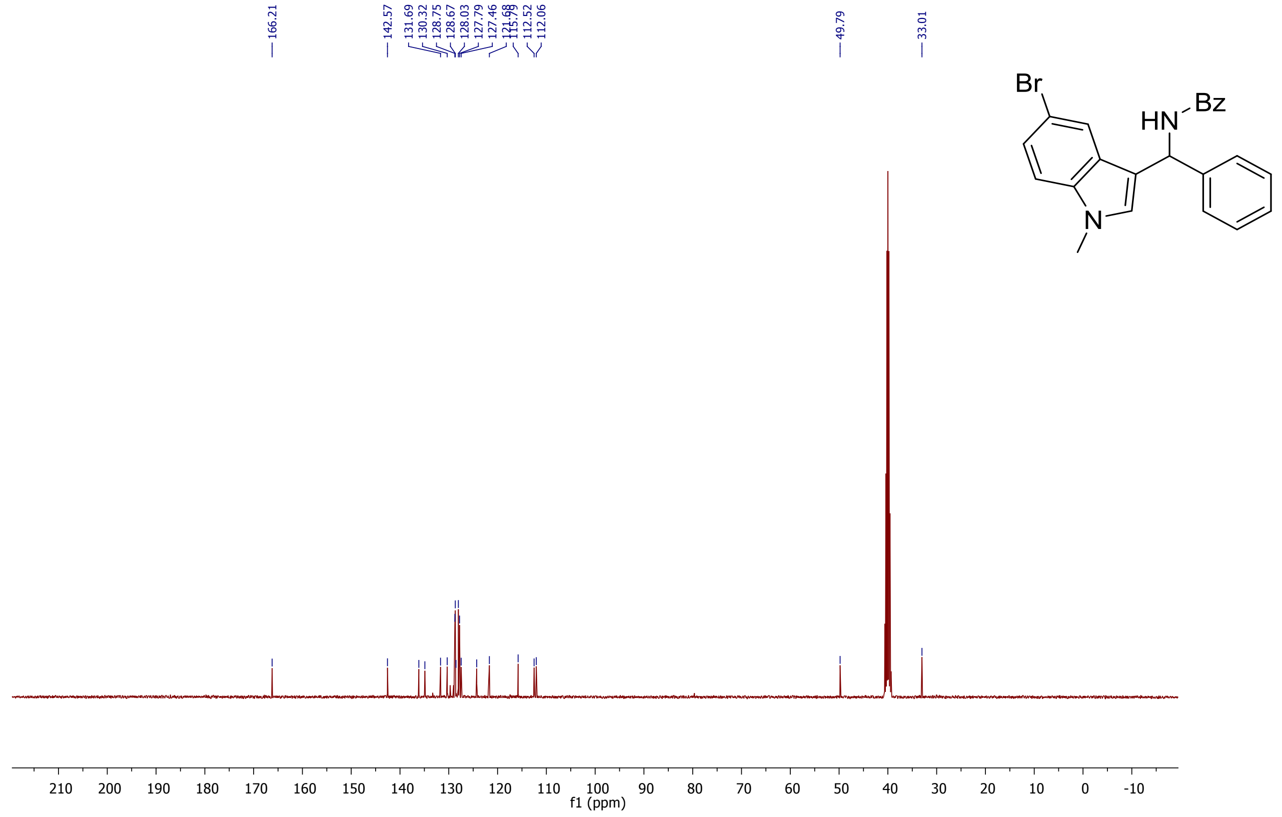

allyl ((5-bromo-1-methyl-1H-indol-3-yl)(phenyl)methyl)carbamate (6i)

^{1}H NMR (400 MHz, $CDCl_3$)

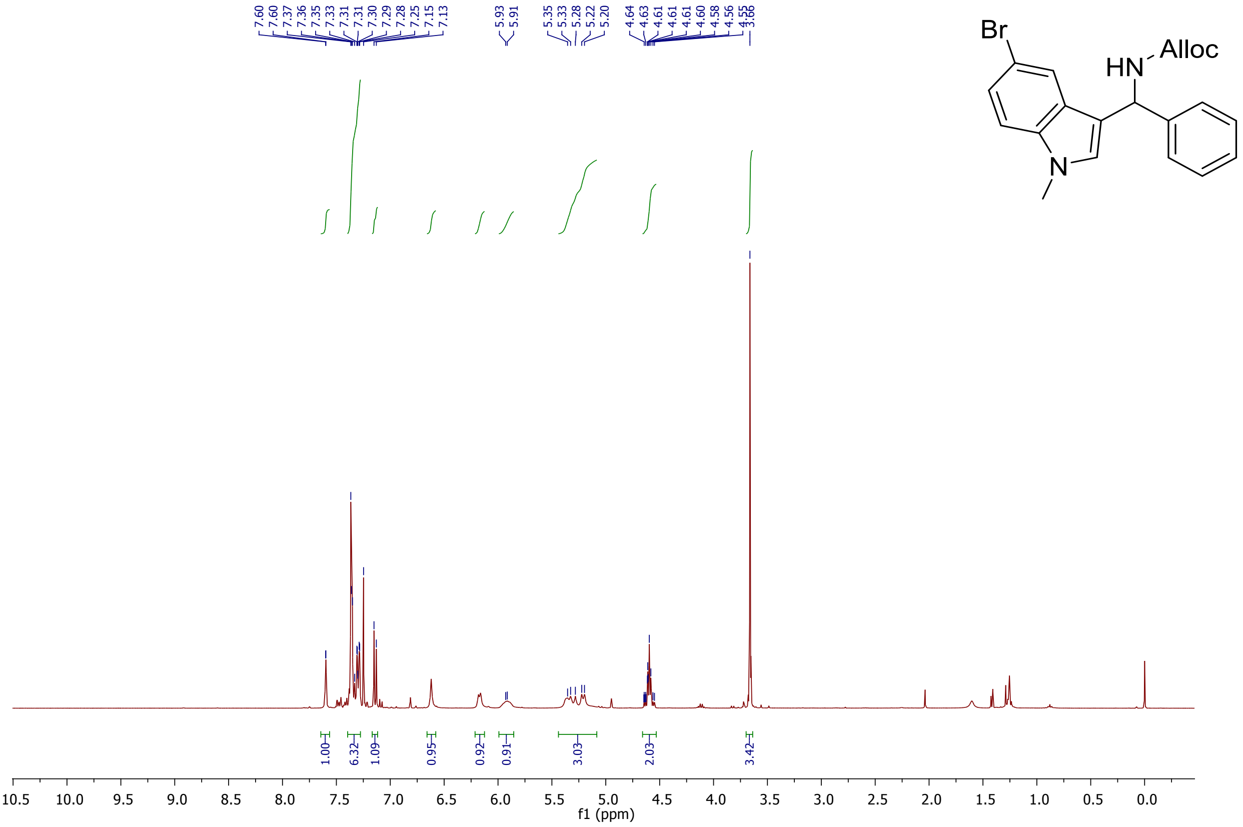

^{13}C{^{1}H} NMR (101 MHz, $CDCl_3$)

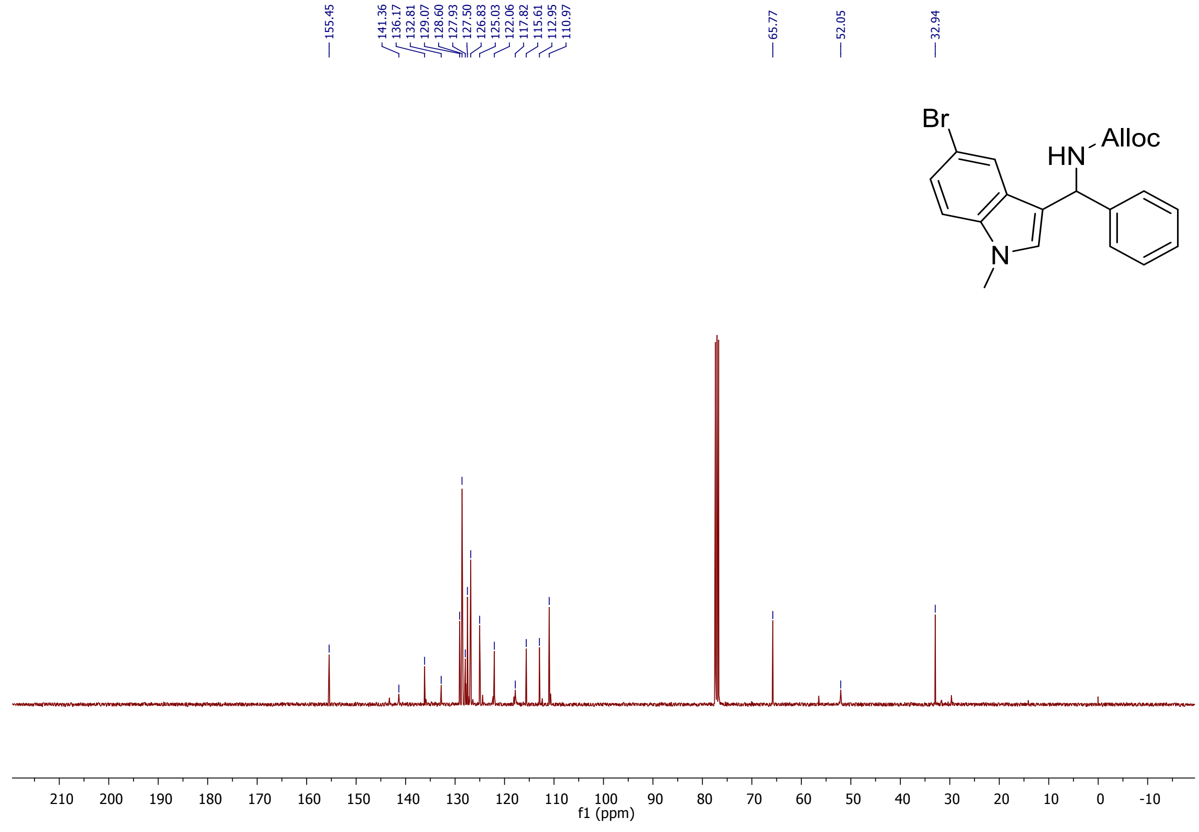

Supplement: Supplementary file 1 [file jo5c03185_si_001.pdf]
